# Chromosome 20q Amplification Regulates *in Vitro* Response to Kinesin-5 Inhibitor

**DOI:** 10.4137/cin.s609

**Published:** 2008-03-26

**Authors:** Aimee L. Jackson, Mao Mao, Sumire Kobayashi, Teresa Ward, Matthew Biery, Hongyue Dai, Steven R. Bartz, Peter S. Linsley

**Affiliations:** 1 Rosetta Inpharmatics LLC, a wholly-owned subsidiary of Merck and Co., Inc., Seattle, WA 98109; 2 Sirna Therapeutics, a wholly-owned subsidiary of Merck and Co., Inc., San Francisco, CA 94158

**Keywords:** chromosome20q, aurora a kinase, KSP, KIF11, RNAi

## Abstract

We identified gene expression signatures predicting responsiveness to a Kinesin-5 (KIF11) inhibitor (Kinesin-5i) in cultured colon tumor cell lines. Genes predicting resistance to Kinesin-5i were enriched for those from chromosome 20q, a region of frequent amplification in a number of tumor types. siRNAs targeting genes in this chromosomal region identified *AURKA*, *TPX2* and *MYBL2* as genes whose disruption enhances response to Kinesin-5i. Taken together, our results show functional interaction between these genes, and suggest that their overexpression is involved in resistance to Kinesin-5i. Furthermore, our results suggest that patients whose tumors overexpress *AURKA* due to amplification of 20q will more likely resist treatment with Kinesin-5 inhibitor, and that inactivation of AURKA may sensitize these patients to treatment.

## Introduction

The variable efficacy of chemotherapeutics among patients highlights the need to identify the factors that predict patient response. Many cancer patients will suffer adverse effects of chemotherapy with no effective response in the tumor. The window of opportunity for treatment of cancer patients can be limited as the patient’s condition deteriorates. The inability to predict the lack of response to therapy can therefore result in loss of valuable time with negative consequences for patient outcome. Genome-wide expression profiling offers the ability to identify patterns of gene expression that correlate with, and predict, responsiveness to cancer therapy(33, 34, 49, 71). We have used expression profiling to identify transcripts whose expression level correlates with cellular resistance to a small molecule inhibitor of the kinesin Kinesin-5 (KSP-1A(63, 64), hereafter referred to as Kinesin-5i).

Members of the kinesin family of microtubule motor proteins play exclusive and essential roles in mitotic spindle function and are potential targets for novel antimitotic cancer therapies. Kinesin-5 (kinesin spindle protein), also known as KIF11, KSP or HsEg5, is a kinesin that plays an essential role in the formation of a bipolar mitotic spindle and is required for cell cycle progression through mitosis(7, 24, 26, 32, 47, 51, 56). Multiple studies, including use of small molecule inhibitors or RNA interference, demonstrate that failure of Kinesin-5 function leads to cell cycle arrest in mitosis with a monopolar mitotic spindle(7, 32, 47, 72), eventually leading to apoptotic cell death or mitotic catastrophe. Kinesin-5 inhibitors are effective in cell lines resistant to Taxol(44), potentially providing a route to overcoming Taxol resistance in the clinic. In addition, Kinesin-5 is expressed only in actively dividing cells and functions exclusively in mitosis, so Kinesin-5 inhibitors may be able to avoid the side effects of Taxol and related tubulin-binding molecules, including peripheral neuropathy(52, 66). The therapeutic potential of Kinesin-5 inhibition has been evaluated through use of antisense oligonucleotides to reduce tumor growth in xenografts(35), and through tumor formation induced by overexpression of Kinesin-5 in transgenic animals(11). Given the potential for improved specificity and unique mechanism of action, Kinesin-5 inhibitors have recently entered clinical trials for cancer therapy. Here we have used expression profiling and RNA interference to identify genes whose expression predicts cellular responsiveness to a Kinesin-5 inhibitor. Furthermore, we have used RNA interference to determine which of the correlated genes are the drivers of resistance, and whose inhibition may sensitize patients to therapy with this inhibitor.

## Materials and Methods

### Cell culture and transfections

All cell lines were obtained from ATCC (Bethesda, MD). HCT-8, COLO320DM, COLO201, COLO205, SNU-C2B, and NCI-H716 were grown in RPMI, all other cell lines were grown in DMEM. In all cases, media were supplemented with 10%FBS and 100U/ml of penicillin and streptomycin. See Supplemental Table 1 for cell lines used in this study. Kinesin-5i was titrated (1:3 dilutions in DMSO) from a starting concentration of 2 uM. Taxol was titrated from a starting concentration of 723 nM. Cell viability was measured by Alamar blue reagent (BioSource International, Camarillo, CA) 72 hours after addition of Kinesin-5i or Taxol, and is reported as percent viability relative to mock-treated cells (DMSO only). EC_50_ values were determined using GraphPad Prism^®^ software as the dose of inhibitor providing a response 50% between maximum and minimum. For siRNA transfections, cells were transfected in 6-well plates using DhamaFect1 (Dharmacon, Lafayette, CO) and the indicated doses of siRNA duplex. Where not specified, the concentration of siRNA was 100 nM. Kinesin-5i was added 4 hours following siRNA transfection, and cell viability was measured by Alamar blue reagent 72 hours later.

### Microarray analysis

RNA from each individual cell line was hybridized against a reference pool containing RNA from 10 of the cell lines. Total RNA was purified by Qiagen RNeasy kit, and processed as described previously(27) for hybridization to Agilent microarrays containing oligonucleotides corresponding to approximately 21,000 human genes. Ratio hybridizations were performed with fluorescent label reversal to eliminate dye bias. Data shown are signature genes that display a difference in expression level (p < 0.01) in 3 cell lines relative to the reference pool. No cuts were placed on fold change in expression. Blue indicates decreased expression; magenta indicates increased expression; black indicates no change in expression. Data were analyzed using Rosetta Resolver^®^ and MatLab (Math-works) software. Transcript regulation was calculated as the error-weighted mean log_10_ ratio for each transcript across the fluor-reversed pair. Microarray data has been deposited at the NCBI Gene Expression Omnibus, GSE 7969.

### Determining AURKA and TPX2 mRNA levels

Total RNA was harvested from exponentially growing cells using the RNeasy Mini kit (#74104 Qiagen, Carlsbad, CA). Reverse transcriptase reactions were performed using the High Capacity cDNA Archive Kit (#4322171 Applied Biosystems, Foster City, CA). Quantitative PCR was performed with TaqMan Universal PCR Master Mix (#43108157, Applied Biosystems) on the 7900HT Sequence Detection System (Applied Biosystems). The GUSB endogenous control, AURKA and TPX2 primer/probe sets were Applied Biosystems #4310888E, Hs0026921_m1, and Hs00201616_m1 respectively.

### Determining AURKA protein levels

Protein lysates were harvested 48 hours post-transfection, and were run on 4%–12% Bis-Tris Gels with MOPS Running Buffer. Gels were transferred to nitrocellulose membranes and probed with mouse monoclonal antibody to AURKA (1:1000 dilution, ab13824, Abcam) or with rabbit polyclonal antibody to Actin (1:10000 dilution, ab8227, Abcam).

### Determining AURKA and TPX2 DNA copy number

DNA was isolated from cell lines using the DNeasy minid kit (#69504, Qiagen). Primer/probes were designed byApplied BiosystemsAssays by Design to introns of *AURKA* and *TPX2* and the endogenous controls *B2M*, *GUSB*, and *GAPD*. Quantitative PCR was performed with TaqMan Universal PCR Master Mix (#43108157, Applied Biosystems). Blood genomic DNA (#636401, Clontech, Mountain View, CA) was used to calibrate the expected diploid delta CTs between *AURKA* and *TPX2* and the endogenous controls so that the ploidy of the tumor cell lines could be determined. The ploidy given is an average of the ploidy determined using the delta CTs between *AURKA* or *TPX2* and each of the 3 endogenous controls.

### siRNA screens

HeLa cells were plated at 600 cells/well in 384-well plates. Cells were transfected with 100 nM each siRNA pool using DharmaFect1 (Dharmacon, Lafayette, CO), and cell viability was measured by Alamar blue (BioSource International, Camarillo, CA) assay 72 hours post-transfection. Each transfection was performed in duplicate. Viability for each siRNA pool was calculated as percent of viability for control siRNA targeting luciferase. Genes sensitizing to Kinesin-5i were selected based on viability of 2 SD from the mean of the population calculated per plate. Data was analyzed using Rosetta iLiminator^®^ software.

### Monoaster analysis and flow cytometry

Cells were plated at 30,000 per well in 24-well plate in DMEM/10%FBS and transfected with the 10 nM siRNAs using Oligofectamine (Invitrogen). Four hours post-transfection, cells were treated with the indicated doses of Kinesin-5i for 24 hours. For monoaster analysis, wells were aspirated and washed once with TBST before exposure to mouse anti-alpha tubulin antibody (SIGMA, #T-9026) at 1:500 and goat anti-mouse Alexa 488-labeled secondary antibody (Molecular Probes, #A21121) at 1:200 in TBST + 5 mg/ml BSA for 4 hours at room temperature. Cells were washed with TBST + Hoechst stain (10 μg/ml), 2 × 10 minutes, followed by a 10 minute wash in TBST without stain. Microphotographs were acquired using a 20X objective on a Leica DMIL inverted fluorescence microscope. For flow cytometry, cell wells were aspirated, washed, and trypsinized. The reserved aspirant, wash, and trypsin cell suspension were combined and pelleted. Cells were resuspended in 1X PBS and ethanol-fixed prior to propidium iodide staining and RNAse treatment for 20 minutes at 37 °C. Flow cytometry was conducted on a Becton-Dickinson FACSCalibur cytometer, followed by analysis using FlowJo (Treestar).

## Results

**Figure 1:**
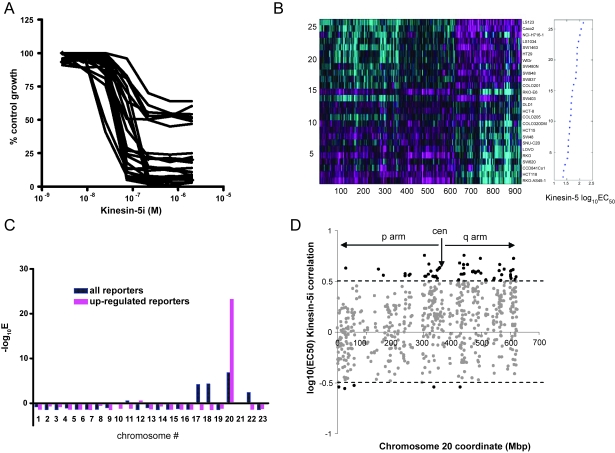
Identification of reporter genes correlating with *in vitro* response to Kinesin-5i (**A**) 26 colon cancer cell lines were tested *in vitro* for responsiveness to Kinesin-5 inhibitor using a 72-hour Alamar blue assay. (**B**) RNA from each individual cell line was profiled against a reference pool corresponding to RNA from 10 of the colon cancer cell lines. Shown is a heat-map analysis of cell lines rank-ordered according to log_10_EC_50_ for Kinesin-5i response (Y axis) and clustered (X axis) with genes correlated (> 0.5 or < − 0.5) with log_10_EC_50_ for Kinesin-5i. The cell lines are ranked from most resistant (top) to most sensitive (bottom) according to log_10_(EC_50_). Pink indicates genes that are more highly expressed in a single cell line compared to the reference pool. Blue indicates genes that are expressed at a lower level in a single cell line compared to the reference pool. Microarray data has been deposited at the NCBI Gene Expression Omnibus, GSE 7969. (**C**) All the Kinesin-5i reporters or just the positive reporters were evaluated for chromosomal distribution. The enrichment of reporter genes on each chromosome was calculated relative to a background set composed of all genes for that chromosome that were present on the microarray. Shown is the –log_10_(e-value) for enrichment of reporter genes (p-value with Bonferroni correction). (**D**) The chromosomal location (x-axis) for each gene from chromosome 20 present on the microarray was compared to the correlation of that gene (x-axis) with log_10_(EC_50_) in the colon tumor lines. Genes with a correlation > 0.5 or < − 0.5 are indicated in black. The location of the centromere (cen) for chromosome 20 is indicated. The p arm of the chromosome is to the left of the centromere, the q arm is to the right of the centromere.

We utilized cancer cell lines to identify constitutive gene expression signatures that correlate with *in vitro* response to a Kinesin-5 inhibitor, KSP-1A(63, 64), referred to here as Kinesin-5i. The derivation of this compound, (1S)-1-{[(2S)-4-(2,5-difluorophenyl)-2-phenyl-2,5-dihydro-1H-pyrrol-1-yl]carbonyl}-2-methylpropylamine, has previously been described (see compound 17 in(18)). We measured the efficacy of this inhibitor in a panel of 26 colorectal cancer cell lines. Colorectal cancer was chosen as the initial model to test this approach, due to the fact that G2 checkpoint defects, found in the majority of colorectal cancers(9), are synthetically lethal with loss of function of *CIN8*, the *Kinesin-5* homolog in *S. cerevisiae*(19). A recent report further supports the idea that the status of the G2 checkpoint in human cancer cells contributes to the cellular response to Kinesin-5 inhibitor(64). We analyzed 26 colorectal cancer cell lines with doubling time less than 48 hours for dose response to Kinesin-5i, using seven-point drug titration curves. Three days after addition of the drug, cell survival was measured by Alamar Blue assay, and reported relative to control (DMSO treated) cells. The cell lines segregated into two clearly distinguishable populations differing in sensitivity to Kinesin-5i (Fig. 1A). These populations differed in endpoint response to the inhibitor, as well as EC_50_, which ranged from 22 nM in the most sensitive line (RKO-AS45-1) to 137 nM in the most resistant line (LS123, see Supplemental Table 1 for all EC_50_ values). Cell lines also segregated according to colon cancer type, with the microsatellite instability (MIN) type colon cancers generally showing sensitivity to Kinesin-5i, and the chromosome instability (CIN) type colon cancer lines showing resistance to the inhibitor. These two distinct types of colon cancers arise from distinct molecular mechanisms(42,55). Our data suggest that these different tumorigenic mechanisms correlate with responsiveness to Kinesin-5i, and that MIN colon tumors will likely respond more favorably in the clinic to this Kinesin-5 inhibitor.

We subsequently used microarray profiling to identify basal (pre-existing) gene expression patterns that correlated with cellular response to Kinesin-5i. RNA from each individual cell line was compared to a reference pool containing RNA from a subset of the samples (N = 10). This approach enabled the identification of transcripts whose expression has significant variation from the reference population. It is important to note that the cell lines were not treated with Kinesin-5i prior to expression profiling, because we wanted to identify transcript expression patterns predictive of response, rather than genes whose expression changes in response to the inhibitor. Transcripts whose expression differed from the reference population (p < 0.01) in 3 or more cell lines were selected for further analysis. The cell lines were rank-ordered according to the log_10_(EC_50_) calculated from the *in vitro* growth assays, and expression signatures correlating with this parameter were identified using a threshold of >0.5 or < − 0.5 correlation. Using these criteria, 932 transcripts were identified using 26 cell lines. The expression of these transcripts in each of the cell lines in the panel is shown in Figure 1B. The cell lines are ranked from most resistant (top) to most sensitive (bottom) according to log_10_(EC_50_). Therefore, transcripts more highly expressed (pink) in cell lines in the top portion of the panel are positively correlated with resistance to Kinesin-5i.

The performance of the reporters identified through this process was evaluated through the leave-one-out validation procedure. Namely, each time we left out cell line(s) derived from the same patient, and used the remaining cell lines to identify the reporters and to construct the prediction model for log_10_(EC_50_). The prediction model was simply a linear fit between the average expression of correlated (or anti-correlated) reporters and the log_10_(EC_50_) . The left-out sample(s) were predicted using this linear model. The process was repeated 26 times until each sample was left out once. The advantage of this approach is that all of the data can be used for training, and none has to be held back for a separate test set. This is especially useful when the sample size is limited, and avoids the possible bias introduced by relying on any one particular division into test and training components. By this leave-one-out process, we found that the baseline expression of negatively-correlated reporter transcripts predicted the log_10_(EC_50_) for Kinesin-5i response with a correlation of 0.56 and p-value = 1.6e^−3^. The baseline expression of positively-correlated reporter transcripts predicted the log_10_(EC_50_) for Kinesin-5i response with a correlation of 0.68 and p-value = 2.6e^−5^. Among 932 genes identified using all 26 cell lines, 304 genes were positively correlated (were more highly expressed in the resistant cell lines), and 628 genes were negatively correlated (were expressed at lower levels in the resistant lines).

Log_10_(EC_50_) reporter genes were analyzed for chromosomal localization. Negatively correlated reporters showed enrichment for genes located on chromosomes 17, 18, and 22 (e-values < 1e^−10^, 2e^−7^, and 2e^−5^ respectively, with Bonferroni correction). Positively correlated reporters showed a significant enrichment for genes located on chromosome 20 (e-value < 10^−23^ with Bonferroni correction, Fig. 1C). There was no enrichment for other chromosomes among the positive reporters. Furthermore, reporter genes from chromosome 20 had much of the predictive power of the entire set of positively correlated reporters, demonstrating that one or more genes harbored on chromosome 20 are implicated in resistance to Kinesin-5i. Neither the positively nor negatively correlated Kinesin-5i reporter genes predicted response to Taxol (correlation = 0.01, p = 4.8e^−1^ for chromosome 20 reporters, not shown). Response to Taxol was instead dominated by the expression level of *ABCB1*, also known as *MDR1* (multi-drug resistance). *ABCB1* expression predicts response to Taxol, but does not predict response to Kinesin-5i (Supplemental Fig. 1). Cell lines were also tested for response to nocodazole and camptothecin (data not shown). Genes whose expression correlated with Kinesin-5i EC_50_ predicted *in vitro* responsiveness to this inhibitor, but did not predict response to any of the other drugs tested. In contrast, genes whose expression correlated with final cell killing by Kinesin-5i were predictive of response to all of the drugs tested. There was good overlap among the genes correlated with endpoint response to all of the drugs tested. Thus, endpoint cell killing was more reflective of general drug response while EC_50_ was more reflective of response to the particular drug under study. Since the genes whose expression correlates with Kinesin-5i EC_50_ appear to be selective for responsiveness to this inhibitor, these reporters might therefore play a direct role in Kinesin-5 function. Given the considerable enrichment for Kinesin-5i resistance reporters on chromosome 20, we focused on the chromosome 20 reporters for further analysis.

The chromosomal coordinate (Fig. 1D, x-axis) of each gene from chromosome 20 present on the microarray was compared to the correlation of that gene’s expression with log_10_(EC_50_) in the colon tumor lines (Fig. 1D, y-axis). Genes whose expression displayed a correlation of > 0.5 or < −0.5 with log_10_(EC_50_) for Kinesin-5i were enriched for those on the q arm of chromosome 20 (Fig. 1D, p-value = 8e^−17^, see Supplemental Table 3 for data). Thus, genes whose expression correlated with resistance to Kinesin-5i were clustered on chromosome 20q. Chromosome 20q is frequently amplified in colon, breast, and ovarian cancers(25,39,48,61,69,70) and cancer cell lines(17), and has been implicated in metastasis and poor prognosis(2,36,62). Chromosomal amplification is the only known mechanism to explain coordinate over-expression of genes mapping to an entire chromosomal arm. Among these will be dominant oncogenes that provide a survival advantage to tumors.

**Figure 2:**
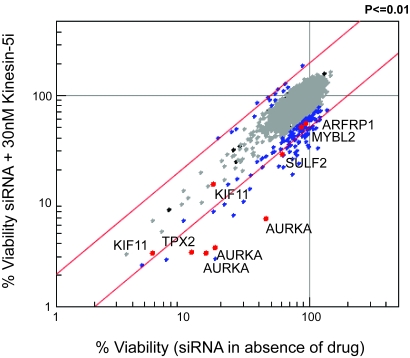
Silencing of several genes on chromosome 20q sensitizes HeLa cells to Kinesin-5 inhibitor HeLa cells were transfected with siRNA pools (3 siRNAs per gene) to each of ~3500 individual genes, including 378 genes on chromosome 20q, in the presence (Y-axis) or absence (X-axis) of 30 nM Kinesin-5i. Cell survival was measured 72-hours post transfection by Alamar blue assay. Each dot indicates survival of cells transfected with siRNAs targeting a single gene. Blue dots indicates genes whose silencing affects viability in response to Kinesin-5i (2SD from the mean of the population). Red dots indicate Kinesin-5i enhancers composed of siRNA pools targeting genes on chromosome 20q.

To functionally test for the driver(s) of Kinesin-5i resistance, we screened for siRNAs that sensitize cells to growth inhibition by a sublethal dose (~EC_20_) of this inhibitor. HeLa cells were selected for this screen because they are readily transfectable with siRNAs, and preliminary experiments in this cell line demonstrated the ability of *KINESIN-5* and *AURKA* siRNAs to enhance the phenotype of Kinesin-5i. The colon cancer cell lines identified in this study as resistant to Kinesin-5i, which would be the natural choice for such a screen, have proven difficult to transfect with siRNAs in high-throughput format for the purpose of a screen. HeLa cells were transfected with a siRNA library targeting ~3,500 genes, including all 378 genes on chromosome 20q. Each gene was represented by a pool of 3 siRNAs. Cell viability was measured 72 hours following addition of 30 nM Kinesin-5i. Genes whose silencing sensitized HeLa cells to the lethal effects of Kinesin-5i would show reduced viability in the presence of Kinesin-5i (y-axis) relative to the absence of Kinesin-5i (x-axis), and therefore would fall into the lower right quadrant of the correlation plot in Figure 2. Three independent screens were performed to identify genes whose silencing enhanced the lethal effect of Kinesin-5i. The results from a representative experiment are shown in Figure 2 (see Supplemental Table 4 for data).

Fifty-one genes were identified for which target silencing enhanced cell killing by Kinesin-5i (2 standard deviations from the mean in any 2 of the 3 screens, Fig. 2). This set of 51 genes displays no significant functional annotation as determined by GO Biological Process, although individual genes such as *KINESIN-5* (the target of Kinesin-5i), additional mitotic kinesins (*KIF13B, KIF15*), and a mitotic regulator (*PLK4*), are consistent with the mitotic function of Kinesin-5i (see Supplemental Table 2 for full list of genes). Also among these genes was *AURKA*, for which 3 independent siRNA pools enhanced the Kinesin-5i phenotype. Only four other genes from chromosome 20q were identified as genes whose silencing enhanced the Kinesin-5i phenotype, *SULF2*, *TPX2, MYBL2,* and *ARFRP1. TPX2*, *AURKA*, and *KINESIN-5* function in the same pathway(4,16,22), and silencing of *TPX2* or *AURKA* sensitizes cells to the lethal effects of Kinesin-5i similarly to silencing of *KINESIN-5* itself.

**Figure 3:**
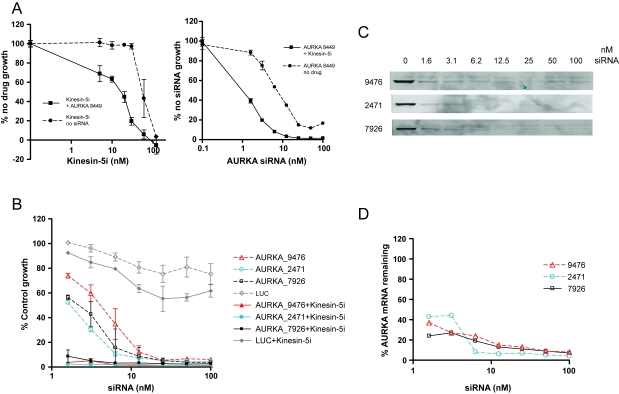
Silencing of *AURKA* sensitizes HeLa cells to Kinesin-5 inhibitor (**A**) Comparison of Kinesin-5i titration (left panel) to siRNA titration (right panel) for growth inhibition. Left panel: Kinesin-5i was titrated in the absence of siRNA (dotted line) or the presence of 100 nM AURKA siRNA (solid line). Right panel: AURKA siRNA was titrated in the absence of Kinesin-5i (dotted line) or the presence of 10 nM Kinesin-5i (solid line). Cell survival was measured 72-hours post transfection by Alamar blue assay. (**B**) 3 siRNAs from a single pool targeting *AURKA* or negative control luciferase were transfected into HeLa cells at the indicated doses. Cells were then grown in the absence (dotted lines) or presence (solid lines) of 10 nM Kinesin-5i. Cell survival was measured 72-hours post transfection by Alamar blue assay. Silencing of AURKA protein (**C**) and mRNA (**D**) following transfection of HeLa cells with the indicated concentrations of siRNA. Protein was harvested 48 h post-transfection. RNA was harvested 24 h post-transfection.

To confirm that target silencing for these 5 chromosome 20q genes enhances the phenotype of Kinesin-5i, and to conform to best practices for siRNA validation(13), the pools were deconvoluted to determine the ability of each individual siRNA to enhance the lethal effect of Kinesin-5i. For these follow-up assays, we decided that dose-titration curves would be more informative than single-point assays. We initially tested 2 dose-titration methods to investigate the impact of gene silencing on growth inhibition in combination with Kinesin-5i. We initially tested a constant concentration of a single siRNA while titrating Kinesin-5i. An AURKA siRNA did shift the dose-response of Kinesin-5i (~5-fold reduction in EC_50_, Fig. 3A, left panel). We also tested a constant concentration of Kinesin-5i with a titration of the siRNA to modulate the amount of target gene silencing. Kinesin-5i shifted the dose-response of AURKA siRNA (~10-fold reduction in EC_50_, Fig. 3A, right panel). The 2 methods yielded similar results showing that the combination of siRNA with drug produced more growth inhibition than either treatment alone. However, the siRNA titration provided more points in the linear range of the curve. We therefore opted to perform our siRNA hit confirmation with this method.

**Figure 4:**
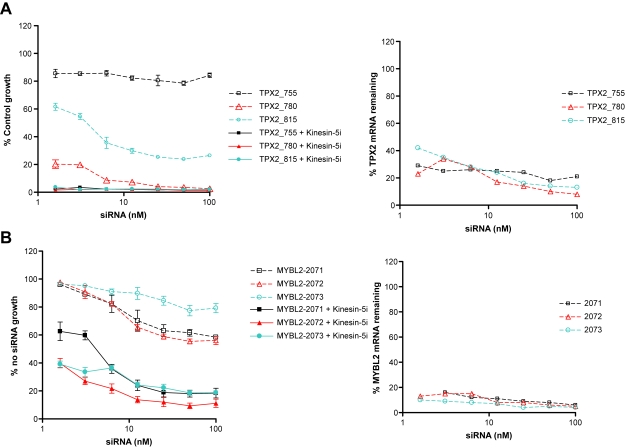
Silencing of TPX2 and *MYBL2* sensitize HeLa cells to Kinesin-5 inhibitor siRNAs targeting (**A**) *TPX2* or (**B**) *MYBL2* were transfected into HeLa cells at the indicated doses. Cells were then grown in the absence (dotted lines) or presence (solid lines) of 25 nM Kinesin-5i. Cell survival was measured 72-hours post transfection by Alamar blue assay. Right panels: Percent target mRNA remaining following transfection of HeLa cells with the indicated concentrations of siRNAs.

In the absence of Kinesin-5i, 3 additional siR-NAs (representing one pool) targeting *AURKA* produced some reduction in cell viability (Fig. 3B). The addition of Kinesin-5i shifted the dose response curve for the *AURKA* siRNAs 5-10-fold to the left (Fig. 3B). The levels of AURKA mRNA silencing and protein silencing were also measured at each dose of the siRNAs. All 3 siRNAs showed similar dose-dependent reduction in protein and mRNA levels (Fig. 3C, D, respectively). At the lowest doses of siRNA, there was still detectable AURKA mRNA and protein. Doses of siRNA greater than 12.5 nM resulted in maximal decrease of AURKA mRNA (~90%) and protein. These doses of siRNA also resulted in maximal growth inhibition, suggesting that the growth inhibition was due to AURKA disruption. Addition of Kinesin-5i caused a shift in dose-response for all 3 *TPX2* siRNAs (Fig. 4A). Although one siRNA was toxic, producing 80% reduction in cell growth, there was additional lethality upon addition of Kinesin-5i. Thus, Kinesin-5i enhances the effects of *AURKA* and *TPX2* siRNAs on cell growth. The impact of the siRNAs on silencing of the target protein is important for interpreting differences in phenotype, but unfortunately we were unable to identify TPX2 antibodies of sufficient specificity and sensitivity to measure TPX2 protein silencing. Since the concentration of Kinesin-5i used in these experiments did not affect cell growth on its own, the effect of Kinesin-5i on *AURKA* and *TPX2* siRNA activity is greater than additive.

*SULF2* has no known link to *AURKA* or *KINESIN-5*. Thus, either we have identified a novel function for *SULF2*, or this represents an off-target effect. Two separate detection methods, qPCR and microarray, indicate that the *SULF2* transcript is not expressed in HeLa cells (detection is below background). In addition, 1 of the siRNAs in the *SULF2* pool has a seed region that is complementary to the *AURKA* transcript sequence. siRNA seed region sequence complementarity has been implicated in silencing of unintended transcripts(28,29). The *SULF2* siRNA with seed region complementarity to *AURKA*, as well as the *SULF2* pool, silences the *AURKA* transcript by 70%–80%, similar to the extent of silencing by the *AURKA* siRNAs (Supplemental Fig. 2). Six additional *SULF2* siRNAs failed to sensitize HeLa cells to Kinesin-5i. Therefore, the enhancement of Kinesin-5i lethality by the *SULF2* siRNA pool is likely an off-target effect of silencing *AURKA*.

We identified *ARFRP1* as a positive reporter for Kinesin-5i resistance by expression profiling and as a gene whose silencing enhances Kinesin-5i lethality. Deconvolution of the *ARFRP1* pool revealed that only 1 of the 3 individual siRNAs sensitized HeLa to Kinesin-5i, and this 1 siRNA only silenced the *ARFRP1* transcript by 40% (Data not shown). The other 2 siRNAs silenced the target by 70%–80%, but did not sensitize to Kinesin-5i. Thus, *ARFRP1* is an off-target hit. Although *ARFRP1* expression is a reporter of Kinesin-5i responsiveness, silencing of this gene does not sensitize cells to Kinesin-5i. Therefore, *ARFRP1* is likely a bystander of chromosome 20q amplification rather than a driver gene.

*MYBL2* (*BMYB*) is myeloblastosis oncogene-like 2, a transcription factor whose expression is regulated at the G1/S border of the cell cycle, and is involved in the regulation of apoptosis, cell division and cell differentiation(30,54). All 3 *MYBL2* siRNAs silenced the target by ≥ 90% at all doses, and all 3 sensitized HeLa cells to Kinesin-5i (Fig. 4B), confirming that *MYBL2* silencing enhances cell killing by Kinesin-5i. Despite testing several MYBL2 antibodies, we were unable to identify an antibody with sufficient specificity and sensitivity to measure silencing of MYBL2 protein. A role for *MYBL2* in the function of Kinesin-5i is currently uncharacterized. However, the demonstration that all individual siRNAs tested for this gene sensitized HeLa cells to the lethal effects of Kinesin-5i suggests a functional role for *MYBL2* in response to this inhibitor and a possible role of *MYBL2* in the Kinesin-5 pathway. Of 387 genes on chromosome 20q tested, 3 genes were confirmed to enhance the effect of Kinesin-5i upon target silencing, and 2 of these, *AURKA* and *TPX2*, function in the Kinesin-5 pathway.

For chromosome 20q amplifications, a likely candidate gene for driving tumorigenesis is *AURKA*(14), also known as *STK6*, *STK15*, or *BTAK. AURKA* DNA amplification is correlated with overexpression of its transcript in cancers and cell lines(6, 57), suggesting that *AURKA* is a target of chromosome 20q13 amplification. Furthermore, Kinesin-5 is a substrate of AURKA *in vitro*(21, 22), suggesting a possible functional consequence of *AURKA* amplification on Kinesin-5 function. *AURKA* was not among the reporter genes derived by expression profiling using the criteria described above, but did show a correlation of 0.42 with Kinesin-5i response. Therefore, the expression of *AURKA* correlated with Kinesin-5i responsiveness, but the correlation fell just below our threshold of 0.5. The decreased correlation of *AURKA* could occur if the microarray probe for this transcript reports expression level with a compressed dynamic range. To determine by another method whether *AURKA* amplification is correlated with resistance to Kinesin-5i, we measured *AURKA* DNA and mRNA copy number in a subset (n = 17) of the colon tumor cell lines by PCR. *AURKA* DNA and mRNA levels were correlated in the colon lines (r = 0.66, Supplemental Fig. 3A). *AURKA* DNA copy number (r = 0.6, Supplemental Fig. 3B) and mRNA level (r = 0.65, Supplemental Fig. 3C) were each correlated with Kinesin-5i EC_50_. *AURKA* mRNA levels showed 2-to-5-fold increased expression in the resistant cell lines. Another gene on chromosome 20q, *TPX2*, activates AURKA(15, 16, 65), in part through promotion of AURKA autophosphorylation (4), and targets AURKA to the microtubules proximal to the spindle pole(38). The mRNA level of *TPX2* was also correlated with Kinesin-5i EC_50_ (r = 0.4, data not shown), and the mRNA level is increased approximately 2-fold in the resistant lines. In contrast, Kinesin-5 mRNA levels were consistent across cell lines, and were not correlated with Kinesin-5i EC_50_ (Data not shown). Given their role in the Kinesin-5 pathway, amplification and/or overexpression of *AURKA* and *TPX2* could affect cellular response to Kinesin-5i through a direct or indirect impact on Kinesin-5 function. The finding that *AURKA* and *TPX2* transcript levels correlate with Kinesin-5i response is not sufficient to prove a causal role for these genes, but this finding, together with the demonstration that silencing of these transcripts enhances the lethal effect of Kinesin-5i, suggests a pivotal role for these genes in Kinesin-5i resistance.

**Figure 5:**
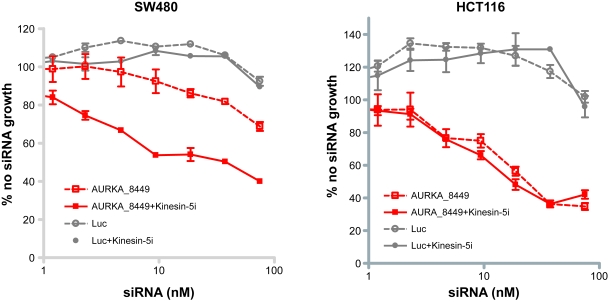
Silencing of *AURKA* enhances cell killing by Kinesin-5 inhibitor in a resistant colon cancer cell line siRNAs targeting *AURKA* or negative control luciferase were transfected into SW480 or HCT116 colon cancer cells at the indicated doses. Cells were then grown in the absence (dotted lines) or presence (solid lines) of Kinesin-5i. Cell survival was measured by Alamar blue assay 72 hours post-transfection.

Subsequently, we tested the ability of *AURKA* siRNAs to sensitize SW480 cells (relatively high levels of *AURKA*, resistant to Kinesin-5i) and HCT116 cells (low levels of *AURKA*, sensitive to Kinesin-5i) to Kinesin-5i. siRNA targeting *AURKA* or negative control luciferase were titrated in each of the cell lines, followed by addition of an EC_10_ concentration of Kinesin-5i. Silencing of *AURKA* had a slight effect on cell viability in SW480 cells, but this effect was increased upon addition of Kinesin-5i (~2-fold increase in percent growth inhibition across the dose-response, Fig. 5). The curves for growth inhibition in the presence and absence of Kinesin-5i are parallel but different. The addition of Kinesin-5i did not enhance cell killing in combination with silencing of luciferase. Since Kinesin-5i did not affect SW480 cells transfected with a control siRNA, the increased cell killing in cells transfected with the *AURKA* siRNA is greater than additive. We have also observed sensitization by *TPX2* siRNAs in SW480 cells (data not shown). Thus, genes identified as Kinesin-5i enhancers in HeLa cells also enhanced Kinesin-5i efficacy in a resistant colon cancer cell line. Silencing of *AURKA* alone decreased cell viability in HCT116 cells, but this effect was not enhanced by addition of Kinesin-5i (Fig. 5). One interpretation of these results is that HCT116 cells are already sensitive to Kinesin-5i due to low levels of *AURKA*, such that further silencing of *AURKA* has no impact on cellular response to the inhibitor.

**Figure 6:**
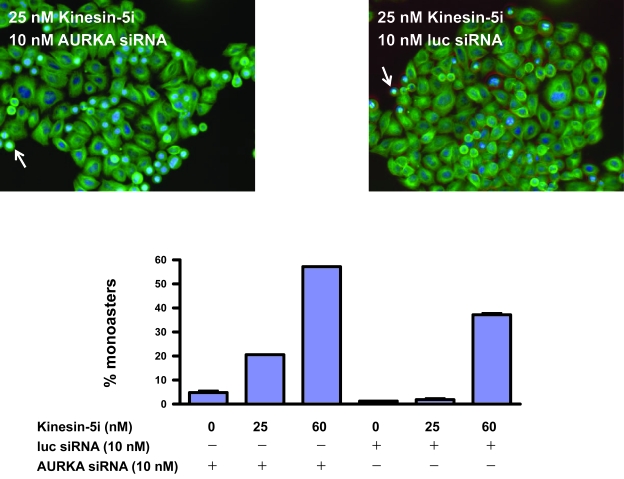
Silencing of *AURKA* increases monoaster formation in response to Kinesin-5i HeLa cells were transfected with 10 nM siRNA targeting AURKA, or negative control siRNA targeting luciferase. Four hours post-transfection, cells were treated with the indicated doses of Kinesin-5i for an additional 24 hours. Cells were treated for detection of alpha tubulin (anti-tubulin antibody, green) and for DNA (Hoescht, blue). Monoasters are detectable as bright blue features. In each panel, an example of a monoaster is indicated with an arrow.

To demonstrate that *AURKA* disruption directly impacts the effect of Kinesin-5i, we measured the effect of *AURKA* silencing on the formation of monoasters, a characteristic of Kinesin-5 disruption(32, 47). Silencing of *AURKA* alone produced monoasters in 5% of HeLa cells (Fig. 6). Addition of 25 nM Kinesin-5i increased the frequency of monoasters 4-fold (20% of cells), while the same concentration of Kinesin-5i did not increase the frequency of monoasters in cells transfected with a control siRNA targeting luciferase. Thus, the increase in monoaster formation by *AURKA* silencing in Kinesin-5i-treated cells is greater than additive. A higher concentration of Kinesin-5i produced monoasters even in the control cells (40% of cells), but the frequency was still greater in cells with *AURKA* disruption (60% of cells). Consistent with an impact on the spindle formation function of Kinesin-5, *AURKA* silencing caused an increase in the percentage of cells in the G2/M phase of the cell cycle (Supplemental Fig. 4). Thus, silencing of *AURKA* interferes directly with Kinesin-5 function in spindle formation and subsequent cell cycle progression.

## Discussion

Early attempts to predict patient response to chemotherapy on the basis of genetic information have focused on one or a few individual genes (*MDR*, *TP53*, *TS*, *EGFR*, etc). In contrast, we have used two unbiased approaches, siRNA screening and genome-wide expression profiling, to investigate the genetic basis of cellular response to the chemotherapeutic agent Kinesin-5i. Our data demonstrate that transcripts whose expression correlates with Kinesin-5i resistance are enriched for those localized to chromosome 20q. Thus, expression of one or more genes on chromosome 20q determines resistance to Kinesin-5i. Predictive approaches to cell line chemosensitivity through gene expression-based classifiers have previously been reported(3,5,45,59,71). In the current study we expand upon this correlative type of analysis to provide evidence that a subset of the predictive transcripts is functionally involved in the cellular response to Kinesin-5i. The demonstration here that of 378 genes on chromosome 20q targeted by siRNAs, only *AURKA, TPX2, and MYBL2* sensitized cells to Kinesin-5i, implicates one or more of these genes as the drivers for resistance to this inhibitor.

AURKA is a ser/thr protein kinase that phosphorylates Kinesin-5 in *Xenopus*(22). *AURKA* is an oncogene(14), is amplified in cancer cell lines and primary tumors(6), and is overexpressed in poor prognosis breast cancer patients(12,67,68). Furthermore, increased expression of *AURKA* correlates with the level of amplification in breast cancer cell lines and colorectal cancers(6,57). TPX2 binds to AURKA and stimulates its auto-activation(4,16). Located on chromosome 20q11, *TPX2* is amplified in giant-cell tumor of the bone(58), and is overexpressed in squamous cell lung cancer(43), neuroblastoma(37), poor prognosis breast cancer(53,67) and endometrial cancer, where its expression level is correlated with stage, grade, and myometrial invasion(8). *MYBL2* is amplified in breast cancers(23,41) and breast cancer cell lines(17), as well as in colorectal tumors of the chromosomal instability type(40). Although chromosomal amplifications are common in cancer, only a minority of genes residing within the amplicon show increased expression(50). This suggests that rare “target” or “driver” genes provide the selective advantage of chromosomal amplifications. For resistance to Kinesin-5i, *AURKA* and *TPX2* fulfill both criteria for defining a target gene for amplification: the putative target gene is located within the core of the amplification region, and amplification leads to over-expression of the gene(70). This suggests that *AURKA* and *TPX2* are strong candidates for the target of chromosome 20q amplification, and play critical causal roles in cancer development. A correlation between amplification and expression of *MYBL2* has not yet been tested, so we cannot yet conclude whether *MYBL2* is a driver gene based on this type of analysis.

We have used transcript expression profiling of cell lines to demonstrate that Kinesin-5i and Taxol have distinct responder populations. While amplification of *AURKA* is linked to resistance to both Kinesin-5i and Taxol, global gene expression identified distinct transcript signatures correlated with resistance (high EC_50_) to these two chemotherapeutics. Resistance to Kinesin-5i was dominated by amplification of chromosome 20q, while resistance to Taxol was dominated by overexpression of the multi-drug resistance gene (*MDR1*). The Kinesin-5i reporter signature was not able to predict response to Taxol, nor was expression of *MDR1* able to predict response to Kinesin-5i. Thus, global expression profiling can identify complex signatures of transcripts whose coordinate regulation is uniquely predictive of cellular response, and therefore define responder populations, for an individual drug. This ability to define patient populations according to likelihood of response could have profound effects on the outcome of clinical trials and on patient outcome.

We demonstrate that *AURKA* and *TPX2* are frequently amplified in cell lines from colon cancer of the chromosome instability (CIN) phenotype. The amplification of *AURKA* and *TPX2* in these cell lines is correlated with resistance to Kinesin-5i. *AURKA* is amplified in colon cancers(6,57) and is associated with the degree of aneuploidy(14), and *AURKA* mRNA expression is increased in sporadic colon cancers with CIN relative to those with-out(20). Amplification of the specific region on chromosome 20q that encompasses *AURKA* occurs in ~90% of CIN-positive colon cancers(31). *MYBL2* has also been reported to be amplified preferentially in CIN-type versus MIN-type (microsatellite instability) colon cancers(40). siRNA-induced silencing of *MYBL2*, *AURKA*, and *TPX2* each sensitized cells to Kinesin-5i, demonstrating that expression of these genes is linked to Kinesin-5i resistance. Carter et al.(10) recently reported an expression signature of chromosomal instability derived by correlating gene expression levels to the level of functional aneuploidy in a diverse set of tumors. Net overexpression of this signature was predictive of poor clinical outcome in several cancer types. The top-ranking genes in the signature included *TPX2* and *AURKA*, further strengthening the finding that amplification and/or overexpression of *TPX2* and *AURKA* are associated with poor clinical outcome. Overexpression of *AURKA* promotes CIN and has been implicated in resistance to other agents that impact on the spindle checkpoint, such as taxanes, by overriding the mitotic spindle assembly checkpoint(1). Consistent with these findings, Phase I/II studies in colorectal cancer, of which approximately 85% are of the CIN type, have failed to demonstrate a clinical benefit following treatment with taxanes (reviewed in(60)). If this failure is due to overexpression of *AURKA*, our data suggest that these patients would also fail to respond to Kinesin-5i. The patients in these trials were not assessed for the CIN phenotype or chromosome 20q amplification. A subset of the colon cancer patient population whose tumors do not display CIN may have responded to the taxane therapy, but this response would be masked by the CIN-positive cohort. Likewise, patients whose tumors do not display CIN or chromosome 20q amplification would be good candidates for response to Kinesin-5i therapy. In the case of colon cancer, this would be the MIN-type tumors, representing ~15% of the patient population. Measurement of *AURKA*, *TPX2*, and *MYBL2* expression in clinical biopsies could potentially distinguish the responder patient cohort (low expression) from the non-responder patient cohort (high expression) for Kinesin-5i therapy.

An additional conclusion from our results is that inhibition of AURKA function could enhance the effect of Kinesin-5i. Our demonstration of growth inhibitory activity of *AURKA* siRNAs suggests that this gene is essential for tumor cell growth and supports investigation of *AURKA* as an anti-tumor target. *AURKA* is a target of small molecule inhibitors under development for cancer therapy by a number of pharmaceutical companies (reviewed in(46)). The interactions between siR-NAs targeting *AURKA* and Kinesin-5i suggest that combination therapy with these compounds might be more effective than therapy with either compound alone, and could help to overcome tumor resistance to either single therapy.

Screening tumor samples for *AURKA*, *TPX2*, and *MYBL2* expression is feasible, and could be incorporated into design of clinical trials for Kinesin-5i response. Prediction of patient response to some therapies will undoubtedly be more complex than measurement of one or a few genetic determinants. The combined expression profiling and RNAi enhancer screen methodology described here can identify a subset of candidate markers implicated in responsiveness to a given chemotherapeutic. Our results indicate that this methodology reveals reporter genes specific for the therapeutic agent, providing a unique opportunity to identify a specific responder population. The combined measurement in clinical samples of the genes identified through this type of analysis will improve the ability to predict patient response, and move us one step closer to individualizing patient treatment.

## Supplementary Material

Supplemental Figure 1. *ABCB1* expression correlates with response to Taxol, but not Kinesin-5i The log_10_ (ratio) expression level of the ABCB1 transcript was determined by microarray for each colon cancer cell line, and was compared to the log_10_(EC50) for either Taxol (left panel) or Kinesin-5i (right panel).

Supplemental Figure 2. SULF2 siRNA alignment with *AURKA* The sequences for 3 *SULF2* siRNAs were aligned by FASTA with the sequence for *AURKA*. Nucleotides of identity between the siRNA sense strand (passenger strand) and the *AURKA* transcript are highlighted in green. The sequence of the siRNA complementary to the seed region is indicated with a black line. The ability of each *SULF2* siRNA to silence *AURKA* or to enhance cell lethality by Kinesin-5i is indicated.

Supplemental Figure 3. Correlation of *AURKA* mRNA and DNA copy number with Kinesin-5i EC50 (**A**) Correlation of *AURKA* DNA copy number with Kinesin-5i EC_50_. (**B**) Correlation of *AURKA* mRNA levels with *AURKA* DNA amplification. (**C**) Correlation of *AURKA* mRNA copy number with Kinesin-5i EC_50_. Red dots indicate HeLa cells.

Supplemental Figure 4. Silencing of *AURKA* increases G2/M cell cycle arrest in response to Kinesin-5i HeLa cells were transfected with 10 nM siRNA targeting *AURKA*, or negative control siRNA targeting luciferase. Four hours post-transfection, cells were treated with the indicated doses of Kinesin-5i for an additional 24 hours. Cells were fixed and stained with propidium iodide for analysis of DNA content. The fraction of cells in each phase of the cell cycle is indicated.

Supplementary Table 1

Supplementary Table 2

Supplementary Table 3

Supplementary Table 4
