# Supplementary figures and images for: Chromosome 20q Amplification Regulates in Vitro Response to Kinesin-5 Inhibitor
Source: Cancer Inform. 2008 Mar 26;6:147–64. doi: 10.4137/cin.s609 (PMC2621078; doi:10.4137/cin.s609)

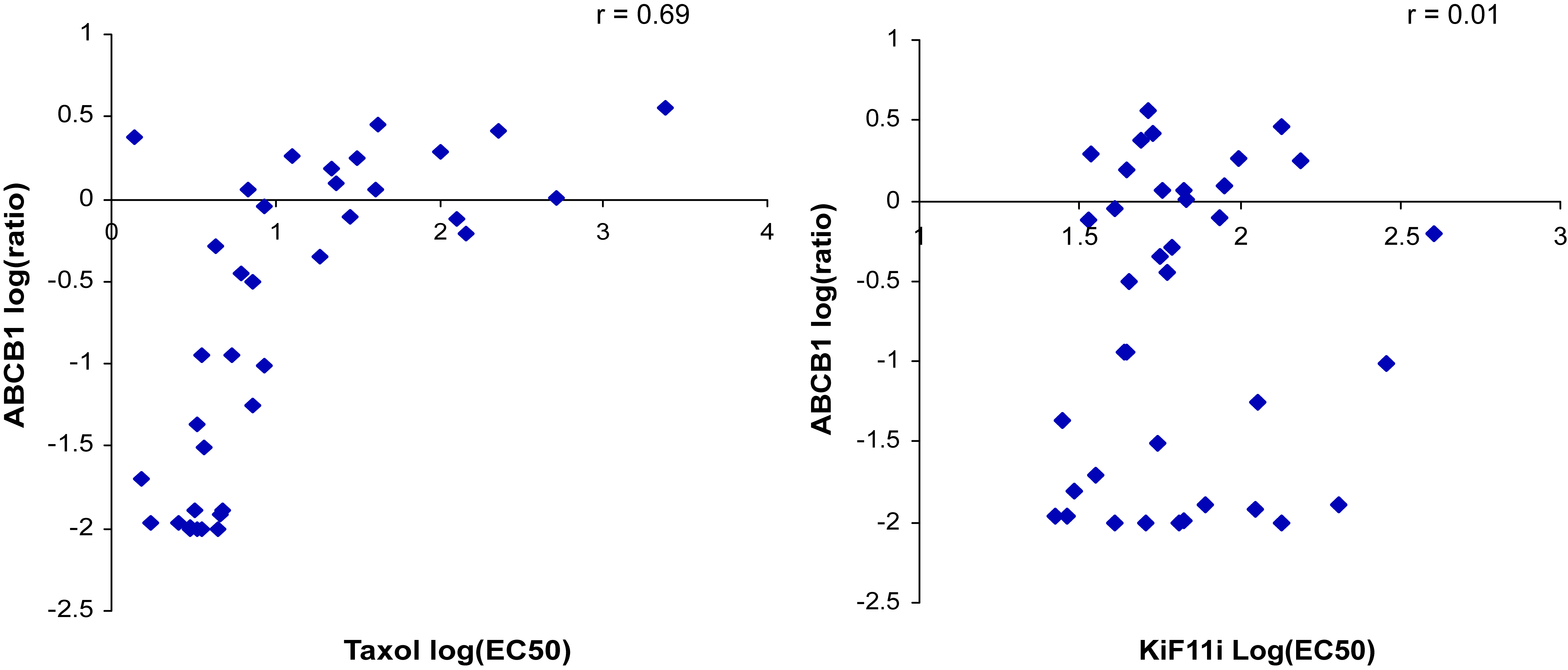

Supplement: Supplemental Figure 1. — The log10 (ratio) expression level of the ABCB1 transcript was determined by microarray for each colon cancer cell line, and was compared to the log10(EC50) for either Taxol (left panel) or Kinesin-5i (right panel). [file cin-6-0147-s1.tif]

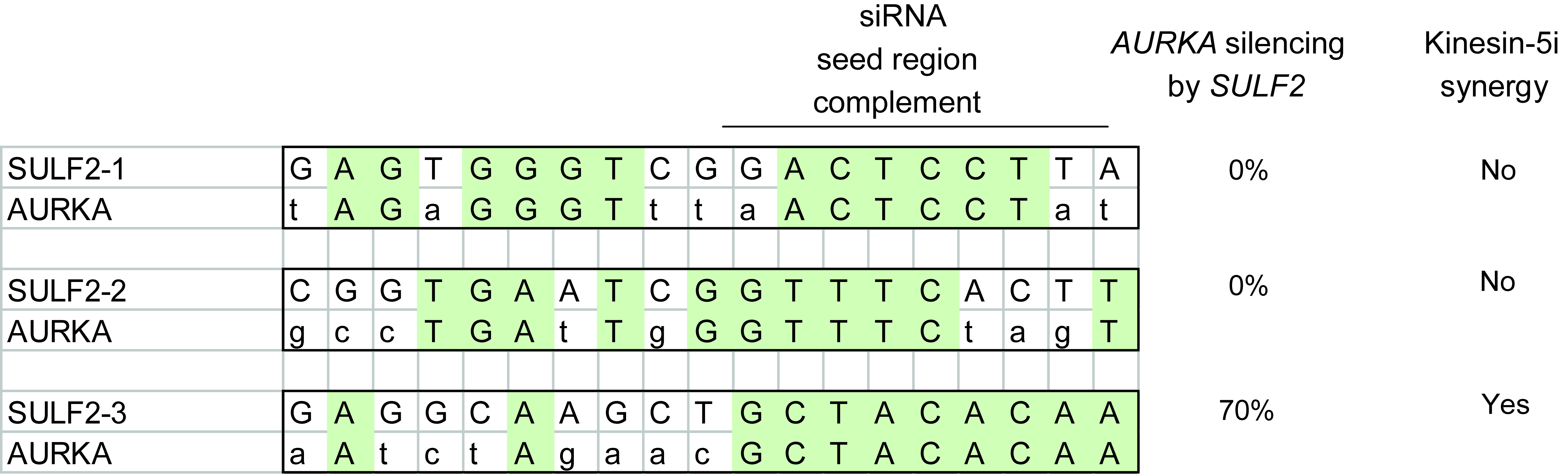

Supplement: Supplemental Figure 2. — The sequences for 3 SULF2 siRNAs were aligned by FASTA with the sequence for AURKA. Nucleotides of identity between the siRNA sense strand (passenger strand) and the AURKA transcript are highlighted in green. The sequence of the siRNA complementary to the seed region is indicated with a black line. The ability of each SULF2 siRNA to silence AURKA or to enhance cell lethality by Kinesin-5i is indicated. [file cin-6-0147-s2.tif]
