# Supplementary material for: Chromosome 20q Amplification Regulates in Vitro Response to Kinesin-5 Inhibitor
Source: Cancer Inform. 2008 Mar 26;6:147–64. doi: 10.4137/cin.s609 (PMC2621078; doi:10.4137/cin.s609)
Supplement: Supplementary Table 1 [file cin-6-0147-s5.doc]

**Supplemental Table 1. Cell lines used in this study.**

Cell line Kinesin-5i EC_50_ (nM) MIN/CIN status Reference pool

LS123 138 HCT116

CaCo2 111 CIN^a^ DLD1

NCI-H716-1 100 LS1034

LS1034 89 CIN^b^ SW620

SW1463 79 SW837

HT29 77 CIN^a^ SW480

WiDr 77 CIN SW48

SW480 76 CIN^a^ HCT-15

SW948 71 LS174T

SW837 69 CIN^a^ HT29

COLO201 58

RKO-E6 52

SW403 49

DLD1 47 MIN^a^

HCT-8 45

COLO205 45

COLO320DM 41

HCT-15 41 MIN^b^

HeLa 40

SW48 38 MIN^a^

SNU-C2B 36

LoVo 36 MIN^b^

RKO 35 MIN^a^

SW620 33

CCD841CoTr 27

HCT116 23 MIN^b^

RKO-AS45-1 22

^a^Cahill, DP, et al., 1998., ^b^Gayet, J, et al., 2001.
