# Supplementary material for: Chromosome 20q Amplification Regulates in Vitro Response to Kinesin-5 Inhibitor
Source: Cancer Inform. 2008 Mar 26;6:147–64. doi: 10.4137/cin.s609 (PMC2621078; doi:10.4137/cin.s609)
Supplement: Supplementary Table 2 [file cin-6-0147-s6.doc]

**Supplemental Table 2. Kinesin-5i enhancer genes from siRNA screens**

| **Gene** | **Accession** | **Chr 20q** | **Confirmed by deconvolution** | **siRNA sequence** |
| --- | --- | --- | --- | --- |
| ALS2CR2 | AF116618 |  |  | GAAACAGAGGGACUACAAATT |
|  |  |  |  | GAUAUCCAAUGGGCUAUAATT |
|  |  |  |  | CUGAUGUUUCCUUGUCAUATT |
| AMFR | NM_001144 |  |  | GAAUUCGUCGGCACAAGAATT |
|  |  |  |  | GAGGACUGCUCAUGUGAUUTT |
|  |  |  |  | CUUCGUGUGGGUUCUAGUATT |
| ARFRP1 | NM_003224 | Yes | No | GUCUUUGUGGGACAAGUAUTT |
|  |  |  |  | CCACCACCGUGGGCCUAAATT |
|  |  |  |  | CUGAUCUUGCCUUGAGGAATT |
| ARPC3 | NM_005719 |  |  | GAUAGGACCUUGAUAUAUATT |
|  |  |  |  | CCCAGCAAGUGGUGGACUUTT |
|  |  |  |  | GCAAGAGACUGGACUGAGATT |
| ATP6V1B2 | NM_001693 |  |  | GGGAGAAACGGCUCGAUUATT |
|  |  |  |  | CGGUUAAUGAAGUCUGCUATT |
|  |  |  |  | CCCUCACUAUCACGGUUAATT |
| AURKA | NM_003600 | Yes | Yes | GCACAAAAGCUUGUCUCCATT |
|  |  |  |  | CCUCCCUAUUCAGAAAGCUTT |
|  |  |  |  | CACCCAAAAGAGCAAGCAGTT |
|  |  |  |  | GCUCUUAAAGUGUUAUUUATT |
|  |  |  |  | GCACCACUUGGAACAGUUUTT |
|  |  |  |  | GGAGUUCUUUGCUAUGAAUTT |
| AURKA_7926 |  |  |  | CAGUUUAUAGAGAACUUCATT |
| AURKA_9476 |  |  |  | GAAUCAGCUAGCAAACAGUTT |
| AURKA_2471 |  |  |  | CAGCAAUUUCCUUGUCAGATT |
| CCND3 | NM_001760 |  |  | CCCUCUGUGCUACAGAUUATT |
|  |  |  |  | GCCUGGGUGCCUGCUCCAUTT |
|  |  |  |  | CUGGUCCUAGGGAAGCUCATT |
| CDC42BPG | NM_017525 |  |  | CUGCUAAGCCCGGCUCACATT |
|  |  |  |  | CAUUCGUGGGCUUCACCUATT |
|  |  |  |  | CCUUCUAUGCUGAGUCCUUTT |
| CDC45L | NM_003504 |  |  | CAGAUCAAAUUACUCAUUATT |
|  |  |  |  | GCCAGUCAAUGUCGUCAAUTT |
|  |  |  |  | CGUGCAGACUUUCAGCAUUTT |
| DDX50 | NM_024045 |  |  | CCGAUUGGAUCUUUCUAAATT |
|  |  |  |  | CUCAGAGACUGAUAGAAGATT |
|  |  |  |  | CAGAGUGGCCGAUUGGAUCTT |
| DLG7 | NM_014750 |  |  | CAGUUUCUGUGAUACCAAATT |
|  |  |  |  | CAGUCAGAACUAUUUGAUATT |
|  |  |  |  | CCGGUCCUCAGAAUACGAATT |
| DYRK2 | NM_006482 |  |  | GGGUAGAAGCGGUAUUAAATT |
|  |  |  |  | GGUGCUAUCACAUCUAUAUTT |
|  |  |  |  | GCCCAACAAUGGUGGCUAUTT |
| ESR2 | NM_001437 |  |  | AUCUGUAUGCGGAACCUCATT |
|  |  |  |  | GCUGUUGGAUGGAGGUGUUTT |
|  |  |  |  | GCCCGGCAGAGGACAGUAATT |
| FANCG | NM_004629 |  |  | CAGUUUCUCAUUGAGGUAGTT |
|  |  |  |  | CAUCAUCUCUGCUACCCAATT |
|  |  |  |  | GUCUGACUCUGAGGCGACATT |
| FLJ20035 | NM_017631 |  |  | CAGAGUCAUGGACAUGUUATT |
|  |  |  |  | CCCACAAAGGCCCUUGUUATT |
|  |  |  |  | GGGAACAUCUCCUUGUCAUTT |
| FURIN | NM_002569 |  |  | GCCAGAAGGUCUUCACCAATT |
|  |  |  |  | CCGGACUUGGCAGGCAAUUTT |
|  |  |  |  | GGACUAAACGGGACGUGUATT |
| FZD2 | NM_001466 |  |  | CGGUCUACAUGAUCAAAUATT |
|  |  |  |  | CGCUCUUCGUGUACCUGUUTT |
|  |  |  |  | CAUCCUAUCUCAGCUACAATT |
| GRK4 | NM_005307 |  |  | CUACGCUUAUGAAACCAAATT |
|  |  |  |  | GAUAGAUUCUUCAAUGAUATT |
|  |  |  |  | GAUGUUGUGACAGAAUGUATT |
| hsa_miR_101 | |  |  | UCAGUUAUCACAGUACUGAAAU |
| hsa_miR_200c | |  |  | CAUCAUUACCCGGCAGUAAUAUCAUCAUUACCCGGCAGUAAUAUBottom of Form |
| hsa_miR_345 | |  |  | Top of Form  CCUGGACUAGGAGUCAGGAAU |
| HSPCA | NM_005348 |  |  | CGGAGGAUCUCCCUCUAAATT |
|  |  |  |  | CCCAGUUGAUGUCAUUGAUTT |
|  |  |  |  | GCUUAGAAGUGAUCUAUAUTT |
| ITSN1 | NM_003024 |  |  | CACAUUGACCGCGUCUAUATT |
|  |  |  |  | GGAGUCUUCCCUUCUAACUTT |
|  |  |  |  | GGCAAUUAUUCAAUAGUCATT |
| KCNA7 | NM_031886 |  |  | CGGGUGGACUCCCAUUUCATT |
|  |  |  |  | CCGUCAUUGUCUCCAAUUUTT |
|  |  |  |  | CACUGUGGGUGGCAAGAUATT |
| KINESIN-5 | NM_004523 |  |  | CUGGAUCGUAAGAAGGCAGTT |
| KIF13B | NM_015254 |  |  | GACACACAUUCCUGUUAUATT |
|  |  |  |  | GUAUGCAGGUCAAGAUAUUTT |
|  |  |  |  | GCUUGAGAGUCUUGGAAUATT |
| KIF15 | NM_020242 |  |  | GGGACCAUCUGAAUCUGAUTT |
|  |  |  |  | GCAGUUGCGUGAAAUGGAATT |
|  |  |  |  | CGUUUGCUCUCAGAAUUAATT |
| LBR | NM_002296 |  |  | CUUGGUGUGGGUUCCCUUUTT |
|  |  |  |  | GAGUUCACUUUAUUAUGAATT |
|  |  |  |  | CGCUGAUUCUGAAGCCAUUTT |
| LMTK2 | NM_014916 |  |  | CCUCUCUGCCUAUCAGAUATT |
|  |  |  |  | GUCAUCGCCGGCUAGAGAATT |
|  |  |  |  | GGCAGACAGUGGCUACGAATT |
| LOC158301 | AI338451 |  |  | GUAUUACGAUCUGCUAGAATT |
|  |  |  |  | GACUGUCUCUGGAGUUUAUTT |
|  |  |  |  | CAUGCUCAGAGUAAACUAATT |
| MAST3 | AB011133 |  |  | GAGUUUGACCCUGAGGAAUTT |
|  |  |  |  | GCGCCACGCUCCUGAAGAATT |
|  |  |  |  | CUUGGAUAGUCCUCGGAAUTT |
| mmu_miR_380_3p | |  |  | GAUGUGGACCAUACUACAAAAU |
| MSH3 | NM_002439 |  |  | GCCAGUUUGUGAACUAGAATT |
|  |  |  |  | GCAAUAAUACCUGCUGUUATT |
|  |  |  |  | GACAGGAGUUUAUGAUAGATT |
| MYBL2 | NM_002466 | Yes | Yes | GUCUCUGGCUCUUGACAUUTT |
|  |  |  |  | GCCUCACCCUGUCAGGUAUTT |
|  |  |  |  | CCAGAAACAUGCUGCGUUUTT |
| PAWR | NM_002583 |  |  | CAGUUUCAGGCAGAUAUAATT |
|  |  |  |  | GUGCUUAGAUGAGUACGAATT |
|  |  |  |  | GAGACUGAUGCAAGAUAAATT |
| PCTK1 | NM_033018 |  |  | CCAACAUCGUUACGCUACATT |
|  |  |  |  | CAGACAACCUUGUGGCACUTT |
|  |  |  |  | CAGAGAUUGUGCACGAGGATT |
| PEPD | NM_000285 |  |  | GGUUCUGCGCUAUACCAAUTT |
|  |  |  |  | CUGCUAUGGUGUCAUCGAUTT |
|  |  |  |  | GAGGUAAUGAAGGCUGUAATT |
| PKD2L2 | NM_014386 |  |  | AAGGUUAUGUCAUCUCUAUTT |
|  |  |  |  | CACAUACUAUAAUGUACAATT |
|  |  |  |  | CCAUUUACUUCAUCACUUUTT |
| PLK4 | NM_014264 |  |  | CUCCUUCUGUGGAUUCAAATT |
|  |  |  |  | CAGACUACAUCAAACAGAATT |
|  |  |  |  | CAGUAUAAGUGGUAGUUUATT |
| POLR2F | NM_021974 |  |  | CCUGCCAGAUGGGAGCUAUTT |
|  |  |  |  | CAGGCCAACCAGAAGCGAATT |
|  |  |  |  | GCCAGGAGAAUGUCGAGAUTT |
| POLR2K | NM_005034 |  |  | CUCUUCCCAUUUCUGAUUGTT |
|  |  |  |  | CAUUUCUGAUUGUUGUAUATT |
|  |  |  |  | CCUCCAAAGCAGCAACCAATT |
| PRPF4B | NM_176800 |  |  | GAACUUACGAGAGGUGUUATT |
|  |  |  |  | GCUAUGACUAUGGUAUAGATT |
|  |  |  |  | CCUAGAUAAACGUUACAAUTT |
| PRPS2 | NM_002765 |  |  | CCUGCAUGCUUCUCAGAUATT |
|  |  |  |  | CCACCAAAGUGUAUGCUAUTT |
|  |  |  |  | CGGAUCACAUCAUCACCAUTT |
| RRM1 | NM_001033 |  |  | GACCAGCAGCUAAUCCAAUTT |
|  |  |  |  | CUAGAUACUUUGGCUGCUGTT |
|  |  |  |  | GAAGUUGGCUGAAGUCACUTT |
| SHC3 | NM_016848 |  |  | GACAGAGUCUUUGACAGUATT |
|  |  |  |  | GAGACUUCCUGGUCAGGAATT |
|  |  |  |  | GACUGACUAUGUUGCAUAUTT |
| SLK | NM_014720 |  |  | ACCAGAAUCUGAGAAUCCATT |
|  |  |  |  | GACAGUGGAUCGAUAUCUUTT |
|  |  |  |  | GGAUCGAUAUCUUUACAAGTT |
| SNF1LK2 | NM_015191 |  |  | GUCUCAACUGCAGGCCUAUTT |
|  |  |  |  | GGCAAAUCCUGUCUGCUGUTT |
|  |  |  |  | CCAAUCAACUGGUCGUGAUTT |
| STAT3 | NM_139276 |  |  | GCCACUUUGGUGUUUCAUATT |
|  |  |  |  | GAGUUGAAUUAUCAGCUUATT |
|  |  |  |  | GGAGCUGUUUAGAAACUUATT |
| STK35 | NM_080836 |  |  | GCUCUUGAAAGAUAUGUUATT |
|  |  |  |  | GGACCGGCCUGAUGCCUUUTT |
|  |  |  |  | CUUUGAACUUGAAACCAGATT |
| STK36 | NM_015690 |  |  | CCCUGAAGUUCAUCCCAAATT |
|  |  |  |  | CUGAGCCUGUGCCUAUUCATT |
|  |  |  |  | GUGAUUCUGUUGCCUUGUATT |
| SULF2 | NM_018837 | Yes | No | CGGUGAAUCGGUUUCACUUTT |
|  |  |  |  | GAGUGGGUCGGACUCCUUATT |
|  |  |  |  | GAGGCAAGCUGCUACACAATT |
| TPX2 | NM_012112 | Yes | Yes | GAGAUUAAGUCAUGGUUUATT |
|  |  |  |  | GAGACAAUGUCACAAGUUATT |
|  |  |  |  | CCGUGCCUGAGAAAGCAUATT |
| UBE2Q2 | NM_173469 |  |  | UUUAUUCAGUGGAACUCAUTT |
|  |  |  |  | GUAUGGAACUUCUCACAAATT |
|  |  |  |  | GACUCAAAGGCAAGACCAUTT |
| UBE2U | NM_152489 |  |  | CUCAUUUAGUGAUUACUACTT |
|  |  |  |  | GCCUGUAAGUGAAGAUAUGTT |
|  |  |  |  | CUGAGAAGUGGAAUACAAATT |
| WNT2 | NM_003391 |  |  | CAUUGACUAUGGGAUCAAATT |
|  |  |  |  | CCAUCCAGGUGGUCAUGAATT |
|  |  |  |  | GGUUUAAGAAGCCAACGAATT |
