# Supplementary material for: Chromosome 20q Amplification Regulates in Vitro Response to Kinesin-5 Inhibitor
Source: Cancer Inform. 2008 Mar 26;6:147–64. doi: 10.4137/cin.s609 (PMC2621078; doi:10.4137/cin.s609)
Supplement: Supplementary Table 3 [file cin-6-0147-s7.doc]

**Supplemental Table 3. Comparison of chromosomal location of chromosome 20 genes with the correlation between gene expression and Kinesin-5i responsiveness.**

| Accession | Gene Symbol | Start coordinate (Mbp) | Correlation of gene expression with log10(EC50) Kinesin-5i |
| --- | --- | --- | --- |
| Contig43129_RC | DEFB126 | 0.71198 | 0.038278016 |
| Contig16788_RC | DEFB127 | 0.8743 | -0.273213364 |
| Contig39620_RC | DEFB129 | 1.57998 | -0.100442801 |
| Contig4467_RC | ZCCHC3 | 2.27661 | -0.150111076 |
| NM_006943 | SOX12 | 2.54238 | -0.31012838 |
| NM_006462 | RBCK1 | 3.36755 | 0.330783755 |
| Contig40598 |  | 3.59975 | -0.079608233 |
| AF116909 | TBC1D20 | 3.64125 | -0.496615895 |
| Contig48114_RC | TBC1D20 | 3.67127 | -0.20058019 |
| Contig45338_RC |  | 4.09753 | -0.279742391 |
| NM_001895 | CSNK2A1 | 4.11337 | -0.263691256 |
| NM_004609 | TCF15 | 5.32638 | -0.372314898 |
| Contig63520_RC | SRXN1 | 5.75269 | -0.540492733 |
| Contig49782_RC | C20orf54 | 6.88724 | 0.184209726 |
| Contig50329_RC | C20orf55 | 7.74455 | 0.213507849 |
| NM_015985 | ANGPT4 | 8.01298 | -0.265893694 |
| NM_006814 | PSMF1 | 10.47244 | -0.054978996 |
| Contig27288_RC |  | 11.00463 | -0.141091114 |
| NM_018354 | C20orf46 | 11.09214 | -0.076769339 |
| NM_014723 | SNPH | 11.94959 | -0.267626731 |
| NM_015685 | SDCBP2 | 12.38622 | 0.100232278 |
| NM_000801 | FKBP1A | 12.97622 | 0.015149815 |
| Contig42363_RC |  | 13.06051 | 0.058414322 |
| NM_016143 | NSFL1C | 13.70812 | -0.114518393 |
| NM_018839 | NSFL1C | 13.70812 | -0.062809104 |
| Contig28050_RC | SIRPD | 14.80336 | -0.1708154 |
| NM_018556 | SIRPG | 15.57797 | 0.233249768 |
| NM_004648 | SIRPA | 18.24081 | -0.350198822 |
| Contig39959_RC | PDYN | 19.07405 | -0.005108729 |
| Contig13568_RC |  | 20.30278 | -0.132679379 |
| Contig39647_RC |  | 20.31754 | -0.240784833 |
| Contig39626_RC | STK35 | 20.76759 | 0.023533223 |
| Contig18462_RC |  | 21.36049 | 0.269447444 |
| NM_003245 | TGM3 | 22.24674 | 0.236911114 |
| NM_003091 | SNRPB | 23.9028 | -0.274837428 |
| Contig40528_RC | ZNF343 | 24.10468 | -0.446561482 |
| NM_006392 | NOL5A | 25.81253 | -0.557839625 |
| NM_006899 | IDH3B | 25.87042 | -0.339658771 |
| AB037863 | RP5-860F19.3 | 26.21523 | -0.016267189 |
| AF161343 |  | 26.25349 | -0.200236258 |
| NM_019609 | CPXM1 | 27.22715 | -0.470874191 |
| Contig48118_RC | C20orf141 | 27.43861 | -0.023672442 |
| NM_002836 | PTPRA | 27.92829 | 0.001565356 |
| Contig41232_RC |  | 29.67401 | 0.239348823 |
| NM_000915 | OXT | 30.00265 | 0.626513997 |
| NM_014948 | UBOX5 | 30.36219 | 0.093166703 |
| Contig15703_RC |  | 30.45131 | -0.452434335 |
| Contig45209_RC | FASTKD5 | 30.75166 | -0.282967939 |
| NM_014731 |  | 30.91272 | 0.129191369 |
| Contig27645_RC |  | 31.1744 | 0.033934453 |
| AF026816 | ITPA | 31.38149 | -0.093545307 |
| Contig52940_RC | C20orf194 | 31.77953 | -0.037762374 |
| AL110249 | C20orf194 | 31.79801 | -0.055924266 |
| NM_012070 | ATRN | 33.99675 | -0.1159566 |
| AL117415 | ADAM33 | 35.96611 | 0.228314141 |
| Contig53537_RC | SIGLEC1 | 36.15618 | -0.387143101 |
| Contig37198_RC | HSPA12B | 36.81291 | 0.197733546 |
| NM_017874 | C20orf27 | 36.8216 | -0.455821151 |
| NM_015417 | C20orf28 | 37.06151 | -0.166527644 |
| X05299 | CENPB | 37.12499 | 0.049597502 |
| NM_004358 | CDC25B | 37.244 | 0.135650745 |
| NM_018347 | C20orf29 | 37.49202 | -0.02607309 |
| AB033097 |  | 37.83204 | 0.193711186 |
| Contig22622_RC |  | 38.04306 | -0.319028147 |
| Contig48643_RC |  | 38.46598 | -0.252435405 |
| Contig64855_RC | PANK2 | 38.47373 | 0.031403936 |
| Contig48480_RC |  | 38.55962 | -0.215088722 |
| NM_007219 | RNF24 | 38.61729 | -0.1658038 |
| NM_019025 | SMOX | 41.00356 | -0.231537333 |
| NM_000311 | PRNP | 46.15068 | -0.1608168 |
| NM_012409 | PRND | 46.50555 | -0.37138866 |
| AL137296 | PRNT | 46.59928 | -0.116264305 |
| NM_014737 | RASSF2 | 47.08669 | -0.161619032 |
| Contig12727_RC |  | 47.62032 | -0.12357068 |
| NM_005116 | SLC23A2 | 47.81001 | 0.004617178 |
| NM_014145 | C20orf30 | 50.28488 | 0.044497543 |
| NM_002592 | PCNA | 50.43599 | -0.166566589 |
| Y16521 | CDS2 | 50.55481 | 0.192546122 |
| Contig30392_RC |  | 51.20956 | 0.220061801 |
| Contig48722_RC |  | 51.25909 | -0.167381679 |
| Contig38591_RC |  | 53.60643 | 0.154734767 |
| Contig34824_RC |  | 54.02362 | 0.428342565 |
| AL137476 |  | 54.24713 | 0.011962447 |
| AB037855 | RP5-1022P6.2 | 54.73086 | 0.129223957 |
| Contig27878_RC | C20orf196 | 57.0166 | 0.166355066 |
| NM_001819 | CHGB | 58.40167 | 0.073624512 |
| NM_015939 |  | 58.66485 | -0.262807907 |
| Contig16394_RC |  | 59.1944 | -0.52898713 |
| Contig31244_RC |  | 59.27162 | -0.328376318 |
| NM_019095 | CRLS1 | 59.34878 | -0.031648563 |
| NM_017671 | C20orf42 | 60.03492 | 0.189144561 |
| NM_001200 | BMP2 | 66.96744 | 0.327211815 |
| Contig48673 | BMP2 | 67.07873 | 0.454267203 |
| Contig21464_RC |  | 67.11014 | 0.404478429 |
| AL121739 | HAO1 | 78.1163 | 0.341285796 |
| AB032988 | TXNDC13 | 79.06023 | 0.085005316 |
| AB011153 | PLCB1 | 80.60911 | 0.196689079 |
| Contig19918_RC |  | 84.63411 | 0.205979391 |
| NM_000933 | PLCB4 | 90.24931 | 0.107970044 |
| Contig25072_RC | PLCB4 | 94.02893 | 0.0163001 |
| NM_012261 | C20orf103 | 94.4327 | -0.056632997 |
| AB033090 | PAK7 | 94.66037 | -0.103309046 |
| NM_003081 | SNAP25 | 101.47476 | -0.111801758 |
| NM_018848 | MKKS | 103.33832 | -0.118001288 |
| Contig34113_RC |  | 104.33212 | 0.037847588 |
| NM_000214 | JAG1 | 105.66333 | 0.021782875 |
| Contig27206_RC | JAG1 | 105.85124 | -0.252204802 |
| Contig40856_RC |  | 106.92874 | -0.102207432 |
| Contig21439_RC |  | 117.38635 | -0.289434373 |
| NM_014962 | BTBD3 | 118.46564 | -0.208449867 |
| Contig15953_RC |  | 128.63527 | -0.195563419 |
| Contig40691_RC |  | 128.79183 | -0.20768378 |
| NM_018327 | C20orf38 | 129.37846 | 0.354370078 |
| Contig56735_RC | C20orf38 | 130.94102 | 0.195793167 |
| Contig42882_RC | C20orf82 | 132.28458 | 0.047306646 |
| NM_017714 | TASP1 | 133.18036 | 0.079960643 |
| NM_016649 | C20orf6 | 136.42969 | -0.469261056 |
| Contig49219_RC | C20orf7 | 137.30184 | -0.179400073 |
| AL137678 | C20orf50 | 137.78051 | 0.012289997 |
| Contig23793_RC |  | 141.77796 | -0.414793232 |
| NM_013281 | FLRT3 | 142.52642 | 0.617409334 |
| Contig26947_RC |  | 156.8467 | -0.099736693 |
| Contig31913_RC | C20orf133 | 159.81415 | 0.557365234 |
| Contig46709_RC | C20orf23 | 162.00749 | -0.077756653 |
| AK000142 | C20orf23 | 163.07805 | 0.047288852 |
| Contig15101_RC |  | 165.18569 | -0.010978023 |
| NM_003092 | SNRPB2 | 166.58628 | -0.344166229 |
| NM_020157 | OTOR | 166.77002 | 0.252446851 |
| Contig34330_RC | OTOR | 166.80421 | -0.060717515 |
| NM_002594 | PCSK2 | 171.5563 | 0.054007345 |
| NM_001195 | BFSP1 | 174.2255 | 0.264494978 |
| NM_006870 | DSTN | 174.98598 | 0.340894347 |
| NM_004587 | RRBP1 | 175.42744 | 0.239881387 |
| Contig327_RC | RRBP1 | 175.47778 | -0.028375859 |
| Contig31391_RC |  | 175.72122 | 0.216451722 |
| Contig22408_RC | C20orf179 | 176.51412 | -0.284520444 |
| NM_014426 | SNX5 | 178.70245 | 0.191912013 |
| Contig43549_RC | SNX5 | 178.96341 | -0.290228796 |
| Contig53837_RC | C20orf72 | 179.19074 | 0.008434168 |
| NM_021220 | OVOL2 | 179.52795 | 0.144094356 |
| AL079276 | OVOL2 | 179.52905 | 0.21498145 |
| Contig16239_RC |  | 179.88194 | -0.39155466 |
| Contig56948_RC | CSRP2BP | 181.13267 | 0.041605985 |
| NM_003434 | ZNF133 | 182.17156 | 0.201619674 |
| Contig36307_RC |  | 183.09739 | -0.165745318 |
| NM_018152 | C20orf12 | 183.12014 | 0.045609601 |
| NM_006466 | POLR3F | 183.96032 | -0.286108354 |
| NM_006606 | RBBP9 | 184.15189 | -0.484220314 |
| Contig51485_RC | RBBP9 | 184.15189 | -0.228985242 |
| NM_006363 | SEC23B | 184.36187 | 0.089438073 |
| NM_014162 |  | 187.17307 | -0.033331894 |
| Contig45772_RC |  | 187.24239 | 0.172902739 |
| Contig29207_RC |  | 187.38383 | -0.122454543 |
| Contig50624_RC | C20orf79 | 187.42556 | -0.161495732 |
| Contig5607_RC |  | 191.37779 | 0.058740657 |
| NM_020689 | SLC24A3 | 191.41289 | -0.044401926 |
| Contig49066_RC | SLC24A3 | 196.51098 | 0.26278388 |
| Contig729_RC | RIN2 | 199.30416 | 0.376040117 |
| NM_016100 | NAT5 | 199.45936 | 0.002265154 |
| Contig29047_RC |  | 199.56481 | 0.214927008 |
| NM_016652 | CRNKL1 | 199.63012 | -0.049311861 |
| AL117439 | C20orf26 | 199.85351 | -0.417508793 |
| Contig31592_RC |  | 200.98824 | -0.136822497 |
| Contig13678_RC | C20orf26 | 202.88965 | -0.163298754 |
| NM_002196 | INSM1 | 202.96764 | 0.240622455 |
| Contig3474_RC |  | 203.18203 | 0.223937369 |
| AK002211 | C20orf74 | 203.20855 | 0.254809332 |
| Contig39664_RC | C20orf74 | 204.0008 | 0.174184327 |
| NM_018474 | C20orf19 | 210.54694 | -0.018213283 |
| Contig29613_RC |  | 210.71995 | -0.000893256 |
| AL117539 | C20orf19 | 211.60773 | 0.009453059 |
| AF035314 |  | 211.66819 | -0.357354288 |
| NM_012255 | XRN2 | 212.31941 | -0.080647977 |
| NM_002509 | NKX2-2 | 214.39663 | 0.261929331 |
| NM_006192 | PAX1 | 216.34363 | 0.068613467 |
| Contig48910_RC |  | 216.46367 | -0.167085243 |
| Contig28452_RC |  | 222.08868 | -0.207049586 |
| AB028021 | FOXA2 | 225.10113 | 0.446347775 |
| NM_001052 | SSTR4 | 229.6412 | -0.262883349 |
| NM_000361 | THBD | 229.7427 | -0.102139672 |
| Contig61938_RC | THBD | 229.7427 | -0.238918869 |
| NM_012072 | CD93 | 230.07993 | -0.22718314 |
| Contig37241_RC |  | 231.18507 | -0.139342295 |
| NM_013248 | NXT1 | 232.79372 | 0.081751019 |
| Contig12058_RC |  | 232.8029 | -0.001552566 |
| Contig49484_RC | GZF1 | 233.0038 | -0.139845194 |
| Contig1619_RC | NAPB | 233.03163 | 0.101969837 |
| Contig26208_RC | CST11 | 233.79041 | -0.288672749 |
| NM_005492 | CST8 | 234.19765 | 0.143720435 |
| AL137625 |  | 234.45521 | -0.128970852 |
| Contig47230_RC | CST9L | 234.93371 | 0.032478865 |
| Contig32509_RC |  | 235.5535 | 0.224753863 |
| NM_000099 | CST3 | 235.62293 | 0.553320203 |
| NM_001899 | CST4 | 236.14276 | 0.572743222 |
| NM_001898 | CST1 | 236.7619 | 0.318672972 |
| NM_001322 | CST2 | 237.52403 | 0.27469767 |
| NM_001900 | CST5 | 238.04571 | 0.393696304 |
| Contig40440_RC |  | 244.92839 | -0.306911443 |
| Contig54547_RC | C20orf39 | 245.94303 | -0.30271849 |
| NM_003650 | CST7 | 248.77865 | 0.058761984 |
| AF090935 | C20orf3 | 248.91578 | 0.238517856 |
| Contig56745_RC | ACSS1 | 249.34867 | 0.555320626 |
| NM_014588 | VSX1 | 250.04099 | 0.341928817 |
| NM_001247 | ENTPD6 | 251.24371 | 0.386718998 |
| NM_002862 | PYGB | 251.76705 | 0.268279791 |
| AL117442 | ABHD12 | 252.28849 | 0.566986735 |
| D80008 | GINS1 | 253.36362 | -0.123449377 |
| AB023197 | RP4-691N24.1 | 253.81461 | 0.083962589 |
| Contig44817_RC | NANP | 255.42212 | 0.151557905 |
| AL049942 | ZNF337 | 256.02851 | -0.150136356 |
| AL096727 | RP13-401N8.2 | 257.02654 | 0.341292459 |
| Contig46352_RC | C20orf91 | 260.1494 | 0.162748765 |
| Contig22720_RC |  | 261.15655 | -0.155270818 |
| Contig64392_RC | DEFB119 | 294.28628 | -0.499951034 |
| NM_014012 | REM1 | 295.26765 | -0.406741523 |
| Contig39516_RC |  | 296.21173 | 0.373837489 |
| NM_002165 | ID1 | 296.56752 | 0.059183641 |
| Contig31153 | COX4I2 | 296.9052 | -0.21674313 |
| NM_001191 | BCL2L1 | 297.15923 | 0.158837102 |
| Contig32556_RC | BCL2L1 | 297.74485 | -0.221484764 |
| AB024704 | TPX2 | 297.90791 | 0.113298517 |
| AK000652 | C20orf57 | 298.99101 | -0.043741304 |
| Contig17675_RC |  | 299.16555 | 0.020455955 |
| Contig61267_RC | PDRG1 | 299.96429 | 0.355578827 |
| Contig21409_RC | C20orf160 | 300.83242 | 0.026642703 |
| NM_002110 | HCK | 301.03717 | -0.263318332 |
| NM_014742 | TM9SF4 | 301.84475 | 0.752927004 |
| Contig31201_RC | TM9SF4 | 302.18937 | 0.360019629 |
| NM_002657 | PLAGL2 | 302.43967 | 0.230183564 |
| D80002 | POFUT1 | 302.59359 | 0.463574878 |
| NM_004798 | KIF3B | 303.29127 | 0.179655217 |
| Contig23853_RC |  | 304.14335 | 0.045825724 |
| AB023195 | ASXL1 | 304.80848 | 0.469265682 |
| AL122043 | C20orf112 | 304.94522 | 0.549355676 |
| AL080086 |  | 305.01218 | -0.352377602 |
| Contig64390 | COMMD7 | 307.54163 | 0.229311494 |
| NM_006892 | DNMT3B | 308.13851 | -0.242576445 |
| NM_012325 | MAPRE1 | 308.71434 | 0.546599862 |
| Contig55248_RC |  | 309.09392 | -0.031692384 |
| NM_016408 | CDK5RAP1 | 314.10305 | 0.060421838 |
| NM_016082 | CDK5RAP1 | 314.10305 | 0.136969624 |
| NM_003098 | SNTA1 | 314.59423 | -0.049315831 |
| NM_005093 | CBFA2T2 | 316.13831 | 0.566007073 |
| Contig51872_RC | C20orf144 | 317.13793 | 0.147245079 |
| M96577 | E2F1 | 317.27149 | 0.166592705 |
| NM_007238 | PXMP4 | 317.58175 | 0.667675736 |
| Contig11009_RC | PXMP4 | 317.69096 | -0.290080303 |
| Contig29397_RC |  | 318.38923 | 0.301193652 |
| Contig65439 | CHMP4B | 319.03584 | 0.492071002 |
| Contig45814_RC |  | 320.43979 | 0.243534402 |
| NM_016732 | RALY | 320.45392 | 0.313903524 |
| NM_007367 | RALY | 320.45392 | 0.375958576 |
| Contig25736_RC |  | 320.77388 | 0.398081037 |
| AF116649 |  | 321.00239 | -0.397536272 |
| NM_003908 | EIF2S2 | 321.39777 | -0.019185436 |
| NM_001672 | ASIP | 323.11831 | 0.173193544 |
| NM_000687 | AHCY | 323.31736 | 0.40305014 |
| AF038564 | ITCH | 324.6524 | 0.436636057 |
| Contig25546_RC | ITCH | 325.60437 | 0.387811179 |
| NM_014183 | DYNLRB1 | 325.67864 | 0.600092087 |
| Contig57721_RC | CDC91L1 | 326.12007 | 0.365030288 |
| AL137597 | TP53INP2 | 327.61963 | 0.257823631 |
| NM_014071 | NCOA6 | 327.66239 | 0.372624473 |
| NM_018677 | ACSS2 | 329.28035 | 0.215131876 |
| Contig43632_RC |  | 329.41891 | 0.308982327 |
| NM_000178 | GSS | 329.79897 | 0.099352908 |
| AB040945 | MYH7B | 330.26866 | 0.010765058 |
| AK000947 | MYH7B | 330.2913 | 0.038944329 |
| AL096738 | TRPC4AP | 330.53875 | 0.306911041 |
| NM_018217 | EDEM2 | 331.6683 | 0.547303175 |
| NM_006404 | PROCR | 332.23434 | -0.127376069 |
| NM_006690 | MMP24 | 332.78116 | 0.279224903 |
| Contig2512_RC |  | 333.27572 | -0.049393958 |
| NM_002212 | ITGB4BP | 333.30138 | 0.400370037 |
| NM_018244 | C20orf44 | 333.53796 | -0.235908122 |
| Contig53187_RC | C20orf44 | 333.95327 | 0.402024116 |
| NM_000557 | GDF5 | 334.84562 | -0.540306505 |
| NM_007186 | CEP250 | 335.06563 | -0.036526283 |
| Contig33602_RC | CEP250 | 335.68343 | 0.07452701 |
| NM_015966 | ERGIC3 | 335.93191 | 0.551262346 |
| AF262992 | SPAG4 | 336.67222 | 0.329213771 |
| NM_003915 | CPNE1 | 336.77381 | 0.541531099 |
| AB018308 | RBM12 | 337.01477 | -0.283073773 |
| NM_021100 | NFS1 | 337.20024 | 0.179798927 |
| AK001470 | NFS1 | 337.20024 | 0.1535631 |
| Contig33147_RC | C20orf52 | 337.50889 | 0.408369578 |
| NM_004902 | RBM39 | 337.54944 | 0.338583543 |
| NM_016436 | PHF20 | 338.23364 | 0.440536214 |
| Contig20889_RC | PHF20 | 338.25888 | 0.294189998 |
| AL137330 | PHF20 | 339.92327 | 0.416475811 |
| Contig49046_RC | SCAND1 | 340.05902 | 0.142917838 |
| Contig65227 |  | 340.96961 | 0.323212883 |
| AB002336 | EPB41L1 | 342.06085 | 0.545687376 |
| NM_015511 | C20orf4 | 342.8786 | 0.129582206 |
| NM_015951 | C20orf4 | 342.91155 | 0.11633785 |
| NM_014902 | DLGAP4 | 343.57716 | 0.419915537 |
| NM_006097 | MYL9 | 346.0331 | 0.176622287 |
| AF055012 | TGIF2 | 346.54004 | 0.163224146 |
| NM_018840 | C20orf24 | 346.6758 | 0.610266301 |
| AF112213 | C20orf24 | 346.67587 | 0.526207592 |
| Contig46796_RC | C20orf172 | 348.14298 | 0.218782148 |
| Contig56909_RC | C20orf117 | 348.39262 | -0.129585531 |
| Contig40385_RC | C20orf117 | 348.45598 | 0.168825539 |
| NM_015377 | C20orf117 | 348.5257 | 0.03407121 |
| NM_015474 | SAMHD1 | 349.54058 | 0.131491032 |
| NM_002895 | RBL1 | 350.59592 | -0.358682083 |
| Contig22779_RC | C20orf132 | 351.90637 | -0.055528615 |
| NM_002951 | RPN2 | 352.40887 | 0.624790237 |
| Contig875_RC | MANBAL | 353.78309 | 0.474545184 |
| NM_005417 | SRC | 354.06501 | 0.069527292 |
| NM_006698 | BLCAP | 355.79233 | 0.632867728 |
| NM_005386 | NNAT | 355.8302 | 0.154134942 |
| Contig50059_RC | CTNNBL1 | 357.94798 | 0.184511281 |
| Contig29223_RC |  | 358.50371 | 0.105180026 |
| Contig50316_RC | C20orf102 | 360.06702 | 0.197477652 |
| AB007866 | KIAA0406 | 360.44837 | 0.156152601 |
| AL117521 | C20orf77 | 361.51918 | 0.39082203 |
| NM_004613 | TGM2 | 361.90278 | 0.397732181 |
| Contig20361_RC | RP5-1054A22.3 | 362.7429 | 0.018765168 |
| NM_001725 | BPI | 363.65998 | 0.273923733 |
| AF105067 | LBP | 364.08316 | 0.144351855 |
| Contig28527_RC | C20orf198 | 365.12408 | 0.30966521 |
| AB033045 | KIAA1219 | 365.34872 | 0.310352487 |
| Contig26811 |  | 366.29664 | 0.232704651 |
| Contig17046_RC |  | 366.43293 | -0.126411625 |
| Contig20913_RC | ACTR5 | 368.33802 | 0.003530441 |
| Contig43923_RC |  | 368.41807 | 0.104269503 |
| AB020630 | PPP1R16B | 368.67761 | -0.108318051 |
| Contig56414_RC | DHX35 | 371.01028 | 0.175494344 |
| Contig16347_RC |  | 371.35867 | -0.144411221 |
| NM_005461 | MAFB | 387.47932 | 0.22072759 |
| J03250 | TOP1 | 390.90919 | -0.05926909 |
| NM_014134 |  | 390.9906 | -0.260589419 |
| Contig32995 |  | 391.09498 | 0.150909784 |
| Contig26384_RC |  | 391.34978 | 0.181431503 |
| NM_002660 | PLCG1 | 391.99574 | -0.313404488 |
| AB007855 | ZHX3 | 392.44168 | 0.073762256 |
| AL137580 | EMILIN3 | 394.22019 | -0.017775642 |
| AB037756 | CHD6 | 394.64657 | 0.478907718 |
| Contig10455_RC |  | 396.24559 | 0.362630226 |
| NM_007050 | PTPRT | 401.34806 | 0.159386964 |
| AL110214 | SFRS6 | 415.23409 | -0.048529929 |
| NM_015478 | L3MBTL | 415.76487 | 0.096108058 |
| AB014581 | L3MBTL | 415.90759 | 0.182684385 |
| NM_016276 | SGK2 | 416.2815 | 0.280475684 |
| Contig37135_RC | SGK2 | 416.4981 | 0.206751171 |
| NM_016004 | IFT52 | 416.52992 | 0.463692049 |
| NM_002466 | MYBL2 | 417.29122 | 0.325486994 |
| Contig45719_RC | C20orf100 | 421.31218 | -0.436040979 |
| NM_020433 | JPH2 | 421.7375 | 0.480169858 |
| Contig24576_RC | JPH2 | 421.73754 | 0.512891061 |
| NM_016470 | C20orf111 | 422.58549 | 0.463312572 |
| Contig46477_RC | GDAP1L1 | 423.26589 | -0.041236937 |
| Contig51235_RC | C20orf142 | 423.64894 | 0.293403598 |
| NM_000457 | HNF4A | 424.63337 | 0.263765451 |
| Contig36321_RC |  | 424.94254 | 0.334958515 |
| AA669593_RC | C20orf121 | 425.53167 | 0.446348726 |
| NM_006811 | SERINC3 | 425.61314 | 0.676325948 |
| NM_007066 | PKIG | 425.93849 | -0.148381201 |
| NM_000022 | ADA | 426.81577 | -0.541990442 |
| Contig38425_RC |  | 427.56947 | -0.01434459 |
| NM_003881 | WISP2 | 427.77298 | -0.131747209 |
| Contig29780_RC |  | 428.10278 | 0.294147022 |
| NM_003404 | YWHAB | 429.47757 | 0.567552299 |
| NM_014052 | YWHAB | 429.68601 | 0.386847619 |
| Contig9370_RC | C20orf119 | 429.98719 | -0.160771648 |
| NM_006809 | TOMM34 | 430.04184 | 0.048094521 |
| NM_006282 | STK4 | 430.28533 | -0.13273546 |
| Contig25510_RC | STK4 | 430.49429 | 0.066957168 |
| Contig50799_RC | STK4 | 431.4112 | 0.041829465 |
| NM_002251 | KCNS1 | 431.54364 | -0.303454105 |
| NM_002638 | PI3 | 432.36911 | 0.213096687 |
| NM_003007 | SEMG1 | 432.69087 | 0.080876663 |
| NM_003008 | SEMG2 | 432.83423 | -0.278803447 |
| NM_003064 | SLPI | 433.14292 | 0.096042904 |
| NM_003066 | SLPI | 433.15056 | 0.131232219 |
| NM_003833 | MATN4 | 433.555 | -0.451888206 |
| NM_014276 | RBPSUHL | 433.68904 | -0.228441013 |
| AB026048 | RBPSUHL | 433.68976 | -0.106867776 |
| NM_002999 | SDC4 | 433.87344 | 0.362898431 |
| Contig28615_RC | C20orf10 | 434.34616 | 0.164865212 |
| NM_014477 | C20orf10 | 434.35934 | 0.203850304 |
| NM_018478 | C20orf169 | 434.68274 | 0.304881293 |
| NM_015937 | PIGT | 434.78137 | 0.566649874 |
| NM_006103 | WFDC2 | 435.31807 | 0.465888259 |
| Contig58156_RC | DNTTIP1 | 438.54026 | 0.466729723 |
| NM_007019 | UBE2C | 438.74661 | 0.499221842 |
| NM_003279 | TNNC2 | 438.85262 | 0.428217997 |
| Contig30152_RC | SNX21 | 439.0332 | -0.303439252 |
| NM_005469 | ACOT8 | 439.03767 | 0.663041683 |
| Contig45320_RC | ZSWIM3 | 439.4066 | 0.628318236 |
| Contig32718_RC | C20orf165 | 439.48531 | 0.474300728 |
| NM_000308 | PPGB | 439.53612 | 0.490465829 |
| NM_006227 | PLTP | 439.60803 | 0.573388927 |
| Contig39085_RC | C20orf67 | 439.96805 | -0.288813622 |
| AL137473 | C20orf67 | 440.07054 | 0.755324012 |
| Contig47464_RC | ZNF335 | 440.10699 | 0.208250338 |
| Contig42870_RC | ZNF335 | 440.30042 | 0.119069723 |
| Contig45944_RC | ZNF335 | 440.33352 | -0.180055716 |
| NM_004994 | MMP9 | 440.70953 | -0.061288433 |
| Contig49118_RC |  | 441.50933 | -0.254796663 |
| NM_001250 | CD40 | 441.80312 | -0.393980836 |
| AF035300 | CDH22 | 442.35782 | -0.115493872 |
| NM_015945 | SLC35C2 | 444.11584 | 0.448182329 |
| NM_018102 | ZNF334 | 445.63115 | 0.173177387 |
| AF154121 | SLC13A3 | 446.2046 | 0.073962865 |
| NM_005244 | EYA2 | 449.56915 | -0.300262063 |
| AB032951 | PRKCBP1 | 452.7248 | 0.664853491 |
| NM_018634 | PRKCBP1 | 453.52764 | 0.483112603 |
| AF144233 | PRKCBP1 | 454.10287 | 0.397552595 |
| Contig38763_RC | PRKCBP1 | 454.17961 | 0.445939113 |
| NM_006534 | NCOA3 | 455.64063 | 0.469553531 |
| AB033073 | SULF2 | 457.19062 | 0.189190825 |
| Contig33236_RC |  | 460.4554 | -0.063422141 |
| Contig20816_RC |  | 464.04717 | 0.115312666 |
| AB037836 |  | 466.74199 | -0.147818954 |
| Contig21787_RC |  | 468.57532 | -0.450942858 |
| NM_006420 | ARFGEF2 | 469.71833 | 0.595169469 |
| NM_001316 | CSE1L | 470.96244 | 0.165892548 |
| NM_004602 | STAU1 | 471.63284 | 0.424705641 |
| NM_017453 | STAU1 | 471.63284 | 0.395474938 |
| NM_017454 | STAU1 | 471.63284 | 0.409910021 |
| NM_017895 | DDX27 | 472.6929 | 0.59782901 |
| AB037825 | ZNFX1 | 472.95847 | 0.284292805 |
| Contig29707 |  | 474.18964 | -0.116934163 |
| NM_004975 | KCNB1 | 474.21911 | 0.59069338 |
| D38145 | PTGIS | 475.57416 | 0.342542616 |
| NM_004776 | B4GALT5 | 476.82891 | 0.035097694 |
| AB023156 | SLC9A8 | 478.62824 | 0.306023584 |
| NM_006038 | SPATA2 | 479.53337 | 0.582964891 |
| NM_018683 | ZNF313 | 479.8632 | 0.403923263 |
| Contig15229_RC | ZNF313 | 479.89261 | 0.42285376 |
| NM_005985 | SNAI1 | 480.32933 | -0.207401167 |
| NM_003349 |  | 481.31069 | 0.109636591 |
| AF155120 |  | 481.7368 | 0.586946 |
| Contig14197_RC |  | 482.15973 | -0.084187736 |
| NM_005194 | CEBPB | 482.40782 | -0.019352368 |
| NM_002827 | PTPN1 | 485.60297 | 0.1773104 |
| Contig41614_RC | PTPN1 | 486.31755 | 0.441231934 |
| Contig52629_RC | C20orf175 | 486.36055 | 0.286329063 |
| Contig44870 | PARD6B | 488.01794 | 0.365751682 |
| Contig37562_RC | PARD6B | 488.03318 | 0.292408934 |
| NM_017843 | BCAS4 | 488.44873 | -0.281614797 |
| Contig28909_RC |  | 489.32123 | -0.295927781 |
| AB018327 | ADNP | 489.40289 | 0.186697875 |
| Contig19877_RC |  | 489.61489 | 0.065694676 |
| NM_003859 | DPM1 | 489.84811 | 0.396457666 |
| Contig16143_RC | DPM1 | 489.87011 | 0.139842289 |
| Contig26144_RC |  | 489.9464 | 0.596816705 |
| NM_014484 | MOCS3 | 490.08769 | -0.037254759 |
| NM_002237 | KCNG1 | 490.536 | -0.47481708 |
| Contig47221_RC |  | 494.36908 | 0.239672199 |
| NM_012340 | NFATC2 | 494.41172 | -0.335627345 |
| U43342 | NFATC2 | 494.4134 | -0.24078112 |
| AB014511 | ATP9A | 496.46723 | 0.724102413 |
| NM_020436 | SALL4 | 498.33991 | 0.580210111 |
| AK001666 | SALL4 | 498.34016 | #N/A |
| Contig45107_RC | ZFP64 | 501.34433 | -0.039049818 |
| NM_018197 | ZFP64 | 502.01226 | 0.47663853 |
| Contig22550_RC |  | 504.14404 | 0.330793349 |
| Contig42103_RC | TSHZ2 | 513.06171 | -0.135312794 |
| Contig10865_RC | TSHZ2 | 515.37489 | -0.013039522 |
| NM_018692 | TSHZ2 | 515.38901 | 0.174211625 |
| Contig47308_RC | TSHZ2 | 515.40513 | 0.487594737 |
| Contig45742 |  | 515.44515 | 0.172109076 |
| NM_006526 | ZNF217 | 516.17018 | 0.511540601 |
| Contig26627_RC |  | 516.37843 | 0.160057305 |
| NM_003657 | BCAS1 | 519.93923 | 0.223043242 |
| NM_000782 | CYP24A1 | 522.03394 | 0.022848069 |
| Contig13724_RC |  | 522.25121 | -0.104216025 |
| NM_002623 | PFDN4 | 522.57908 | 0.169781191 |
| NM_018431 | DOK5 | 525.25672 | -0.071466907 |
| Contig16588_RC | CBLN4 | 540.05903 | 0.152340965 |
| Contig28438_RC |  | 541.11669 | 0.090896704 |
| NM_019888 | MC3R | 542.57195 | -0.332057521 |
| Contig15196_RC |  | 543.04461 | -0.11319404 |
| Contig34755_RC | C20orf108 | 543.68742 | 0.108320515 |
| AL133555 | C20orf108 | 543.75409 | 0.076470898 |
| NM_003600 | AURKA | 543.77851 | 0.428420971 |
| NM_003158 | AURKA | 543.77872 | 0.262202476 |
| NM_001324 | CSTF1 | 544.0098 | 0.510817455 |
| NM_020356 | C20orf32 | 544.2077 | 0.016115354 |
| NM_016407 | C20orf43 | 544.77091 | 0.529772027 |
| Contig25700_RC | RP5-1153D9.3 | 545.41921 | -0.405913931 |
| NM_003222 | TFAP2C | 546.37764 | -0.080691169 |
| NM_001719 | BMP7 | 551.78961 | 0.221418535 |
| NM_012444 | SPO11 | 553.38237 | 0.095577322 |
| NM_003610 | RAE1 | 553.59551 | 0.367625492 |
| Contig25142_RC | RAE1 | 553.87266 | 0.145782407 |
| X75315 | RBM38 | 554.00125 | -0.074714736 |
| NM_002591 | PCK1 | 555.69542 | 0.176248269 |
| NM_020182 | TMEPAI | 556.56857 | 0.409398651 |
| Contig1805_RC | TMEPAI | 556.56859 | 0.380135412 |
| Contig51433_RC | C20orf85 | 561.59453 | -0.247994216 |
| Contig52380 |  | 562.37402 | 0.240801453 |
| Contig52382_RC |  | 562.38647 | 0.277155441 |
| NM_018498 | PPP4R1L | 562.4638 | 0.291171072 |
| Contig10268_RC | PPP4R1L | 562.55519 | 0.351193519 |
| NM_020673 | RAB22A | 563.18176 | 0.602526005 |
| AL049985 | RAB22A | 563.74142 | 0.248148006 |
| NM_004738 | VAPB | 563.9765 | 0.664393103 |
| Contig18780_RC |  | 564.31937 | 0.322177274 |
| Contig41692_RC | VAPB | 564.54575 | 0.046619937 |
| Contig57172_RC |  | 564.58671 | 0.205754161 |
| NM_003763 | STX16 | 566.59733 | 0.203014879 |
| Contig58430_RC | STX16 | 566.86534 | 0.505634259 |
| Contig43192_RC |  | 567.12385 | 0.001425698 |
| Contig51087_RC | NPEPL1 | 567.22521 | 0.355066219 |
| Contig30569_RC |  | 567.82887 | 0.093969678 |
| AJ251759 |  | 568.27368 | -0.036696067 |
| NM_016592 | GNAS | 568.48189 | 0.363534777 |
| Contig27587_RC |  | 568.85043 | 0.071707275 |
| NM_000516 | GNAS | 569.00129 | 0.265835286 |
| Contig44039_RC | GNAS | 569.02542 | 0.249874446 |
| Contig30072_RC | GNAS | 569.06815 | -0.037774942 |
| Contig53674_RC | GNAS | 569.16493 | 0.187857169 |
| NM_016397 | TH1L | 569.89725 | 0.304727888 |
| NM_001336 | CTSZ | 570.03635 | 0.341935033 |
| Contig56697 | TUBB1 | 570.33153 | -0.247121507 |
| NM_006886 | ATP5E | 570.37128 | 0.561785379 |
| NM_016045 | C20orf45 | 570.416 | 0.307710831 |
| Contig15081_RC | C20orf174 | 572.64116 | -0.142079681 |
| NM_000114 | EDN3 | 573.08893 | 0.187777381 |
| NM_014258 | SYCP2 | 578.72013 | 0.307434778 |
| NM_006242 | PPP1R3D | 579.45286 | 0.620320384 |
| AL137442 | C20orf177 | 579.52596 | 0.40109748 |
| Contig40177_RC | CDH26 | 580.21214 | 0.45790509 |
| Contig21187_RC |  | 580.5445 | 0.487774569 |
| Contig44954_RC |  | 580.919 | 0.07076371 |
| Contig35542_RC |  | 580.97144 | 0.379005573 |
| Contig32990_RC |  | 590.8758 | -0.368524517 |
| NM_001794 | CDH4 | 592.60953 | -0.337289714 |
| NM_003185 | TAF4 | 599.83249 | 0.4381195 |
| Contig28035_RC | LSM14B | 601.37296 | -0.16666973 |
| NM_016182 | LSM14B | 601.4313 | 0.565131251 |
| NM_014054 | LSM14B | 601.4313 | 0.595684316 |
| NM_002792 | PSMA7 | 601.45185 | 0.562922546 |
| AB014593 | SS18L1 | 601.52245 | 0.327713824 |
| AK001603 | GTPBP5 | 602.01866 | 0.483761185 |
| NM_007232 | HRH3 | 602.23421 | 0.225889556 |
| NM_014835 | OSBPL2 | 602.46974 | 0.547934924 |
| NM_007002 | ADRM1 | 603.11421 | 0.499806325 |
| AB011105 | LAMA5 | 603.17609 | 0.525458808 |
| Contig50324_RC |  | 603.94754 | 0.201456363 |
| NM_001024 | RPS21 | 603.95515 | 0.379982009 |
| X79563 | RPS21 | 603.95568 | 0.370958535 |
| Contig54187_RC | CABLES2 | 603.9708 | 0.601801567 |
| Contig32979_RC | GATA5 | 604.72554 | -0.075246215 |
| Contig61890_RC |  | 607.35601 | -0.043126049 |
| NM_016354 | SLCO4A1 | 607.44241 | 0.250624448 |
| NM_002531 | NTSR1 | 608.10633 | -0.173863629 |
| NM_018270 | C20orf20 | 608.98282 | 0.280804617 |
| Contig21434_RC |  | 609.02437 | 0.412301917 |
| Contig16724_RC |  | 609.0317 | 0.060036089 |
| NM_007346 | OGFR | 609.06621 | 0.139582973 |
| NM_001853 | COL9A3 | 609.18831 | 0.074338057 |
| NM_006602 | TCFL5 | 609.42912 | 0.342170915 |
| AL133063 | DIDO1 | 609.79541 | 0.286321559 |
| AB002331 | DIDO1 | 609.89013 | 0.344482826 |
| Contig21352_RC | DIDO1 | 610.0422 | 0.246590231 |
| AK002127 | DIDO1 | 610.06799 | 0.262468461 |
| NM_017896 | C20orf11 | 610.39885 | 0.186295347 |
| Contig45932_RC |  | 610.40952 | -0.272964424 |
| Contig1924_RC | C20orf11 | 610.49374 | 0.612502246 |
| AL117533 | C20orf51 | 611.36014 | 0.064602189 |
| NM_017798 | YTHDF1 | 612.97228 | 0.721667425 |
| NM_018209 | ARFGAP1 | 613.74609 | 0.427301757 |
| AB040943 | COL20A1 | 614.07809 | 0.409982837 |
| NM_000744 | CHRNA4 | 614.45108 | -0.321003167 |
| NM_004518 | KCNQ2 | 615.08249 | -0.410627373 |
| AL080160 |  | 615.79727 | 0.194037703 |
| NM_001958 | EEF1A2 | 615.89809 | 0.060950186 |
| NM_005975 | PTK6 | 616.30221 | 0.39338052 |
| NM_012384 | GMEB2 | 616.89398 | 0.074806529 |
| NM_015894 | STMN3 | 617.41504 | -0.034629023 |
| NM_016434 | RTEL1 | 617.6009 | -0.010296841 |
| AB029011 | RTEL1 | 617.61199 | -0.276169386 |
| NM_003823 | TNFRSF6B | 617.98464 | -0.0359076 |
| NM_003224 | ARFRP1 | 618.01252 | 0.517594635 |
| Contig50920_RC | ZGPAT | 618.37137 | 0.439319721 |
| Contig2608 | SLC2A4RG | 618.44353 | 0.396505732 |
| NM_017962 |  | 619.17392 | -0.219233097 |
| NM_003288 | TPD52L2 | 619.67033 | 0.339398146 |
| NM_017859 | UCKL1 | 620.41631 | 0.295665321 |
| AB033022 |  | 620.58501 | 0.223977887 |
| Contig51339_RC | SAMD10 | 620.75912 | 0.497514288 |
| NM_012469 | PRPF6 | 620.82874 | 0.543916628 |
| AL157500 | PRR17 | 621.37459 | -0.327529314 |
| NM_003195 | TCEA2 | 621.64453 | 0.02508796 |
| NM_005873 | RGS19 | 621.7498 | 0.011572131 |
| NM_000913 | OPRL1 | 621.81931 | 0.008529722 |
| Contig33852_RC | OPRL1 | 622.01984 | -0.18803874 |
| NM_005286 | NPBWR2 | 622.07627 | 0.144886898 |
| NM_018257 | PCMTD2 | 623.57564 | 0.223192308 |
