# Supplementary material for: Chromosome 20q Amplification Regulates in Vitro Response to Kinesin-5 Inhibitor
Source: Cancer Inform. 2008 Mar 26;6:147–64. doi: 10.4137/cin.s609 (PMC2621078; doi:10.4137/cin.s609)
Supplement: Supplementary Table 4 [file cin-6-0147-s8.doc]

**Supplemental Table 4. siRNA viability screen in the presence and absence of Kinesin-5i.** Each row represents the data for a pool of 3 siRNAs targeting the gene of interest.

| Gene Symbol | Sequence Code | %Viability no Kinesin-5i | Std Dev no Kinesin-5i | %Viability siRNA + 30nM Kinesin-5i | Std Dev siRNA + 30nM Kinesin-5i |
| --- | --- | --- | --- | --- | --- |
|  | M15077 | 99.75 | 0.57 | 96.93 | 0.59 |
| PLK1 | NM_005030 | 7.77 | 0.8 | 8.26 | 1.13 |
| TM2D2 | NM_078473 | 87.71 | 3.84 | 84.28 | 14.79 |
| KCNC4 | NM_004978 | 67.47 | 1.94 | 48.92 | 3.73 |
| ADCY6 | NM_015270 | 112.54 | 4.9 | 126.26 | 8.52 |
| ADCY5 | AF497517 | 107.22 | 16.34 | 89.47 | 15.71 |
| FBXW10 | NM_031456 | 50.02 | 28.17 | 49.71 | 15.42 |
| CDC2L6 | NM_015076 | 122.33 | 1.5 | 129.89 | 22.23 |
| HDAC10 | NM_032019 | 88.88 | 5.39 | 96.39 | 1.76 |
| EIF2AK4 | AB037759 | 104.92 | 13.58 | 132.5 | 12.13 |
| CATSPER2 | NM_172095 | 56.24 | 12.24 | 71.29 | 4.78 |
| IHH | L38517 | 73.87 | 4.32 | 90.48 | 5.94 |
| ATRX | NM_138271 | 85.94 | 0.21 | 94.8 | 2.35 |
| SLC30A5 | NM_022902 | 97.91 | 2.54 | 96.07 | 7.55 |
| MAPKAPK5 | NM_139078 | 81.77 | 12.76 | 100.04 | 28.99 |
| WHSC1 | NM_133330 | 110.22 | 2.12 | 121.16 | 12.49 |
| PTP4A2 | NM_003479 | 76.18 | 0.29 | 58.19 | 2.43 |
| ZUBR1 | NM_020765 | 73.24 | 2.75 | 69.8 | 9.67 |
| BCL2L14 | NM_138723 | 109.57 | 11.58 | 124.18 | 0.98 |
| FBXO24 | NM_033506 | 52.48 | 1 | 74.97 | 16.13 |
| ILK | AJ277481 | 111.03 | 9.95 | 125.2 | 4.88 |
| NR4A2 | NM_006186 | 84.25 | 8.23 | 90.97 | 5.64 |
| TTBK1 | AB058758 | 107.9 | 5.07 | 118.81 | 7.39 |
| SKP1A | NM_170679 | 85.56 | 5.91 | 113.49 | 6.97 |
| FBXW7 | NM_033632 | 101.01 | 9.54 | 103.05 | 3.36 |
| ICK | NM_014920 | 97.71 | 7.23 | 104.95 | 8.14 |
| DDX17 | NM_006386 | 107.24 | 4.09 | 101.05 | 1.9 |
| NR1I2 | NM_022002 | 104.78 | 14.08 | 103.23 | 0.05 |
| DNMBP | AB023227 | 101.01 | 4.64 | 108.01 | 4.6 |
| TRADD | NM_003789 | 81.51 | 9.27 | 74.97 | 3.65 |
| PPP1R8 | NM_014110 | 97.09 | 7.74 | 97.07 | 17.8 |
| CTSB | NM_147780 | 59.27 | 7.98 | 72.13 | 7.93 |
| STK19 | NM_032454 | 105.16 | 5.9 | 75.27 | 21.88 |
| DDX48 | NM_014740 | 14.2 | 1.69 | 9.54 | 0.04 |
| RAD51 | NM_002875 | 83.56 | 2.35 | 97.63 | 2.13 |
| RAD51L3 | NM_002878 | 85.38 | 4.63 | 111.91 | 10.49 |
| ACOT11 | NM_015547 | 87.71 | 1.36 | 63.73 | 2.23 |
| RAD52 | NM_002879 | 91.98 | 4.59 | 95.25 | 2.94 |
| STARD7 | NM_020151 | 99.67 | 9.26 | 93.17 | 11.28 |
| BZRP | NM_000714 | 103.8 | 3.23 | 138.05 | 11.9 |
| VDR | NM_000376 | 94.05 | 3.64 | 93 | 3.33 |
|  | XM_292638 | 73.67 | 4.87 | 48.09 | 12.52 |
| RAD1 | NM_133377 | 63.06 | 0.12 | 85.68 | 31.17 |
| ZAK | NM_016653 | 101.96 | 4.4 | 114.03 | 11.23 |
| DICER1 | NM_177438 | 95.92 | 0.35 | 118.16 | 1.27 |
| TRPV1 | NM_080706 | 103.95 | 11.64 | 75.42 | 11.19 |
| UBE2J1 | NM_016021 | 84.85 | 3 | 102.64 | 4.19 |
| DNMT3B | NM_006892 | 92.93 | 6.85 | 93.73 | 2.77 |
| ST3GAL3 | NM_174963 | 76.54 | 4.37 | 75.39 | 12.12 |
| DDX41 | NM_016222 | 48.82 | 2 | 39.26 | 2.87 |
| FBXO3 | NM_012175 | 101.62 | 3.94 | 98.42 | 20.21 |
| CAMK1D | NM_020397 | 105.5 | 9.94 | 121.17 | 1.35 |
| FBXO4 | NM_012176 | 65.86 | 2.06 | 67.57 | 3.31 |
| PLEKHG4 | AK024475 | 100.43 | 3.28 | 108.82 | 6.43 |
| UCP3 | NM_003356 | 75.54 | 5.59 | 62.78 | 6.66 |
| PTPDC1 | NM_152422 | 90.09 | 4.37 | 70.09 | 7.27 |
| RAD17 | NM_133339 | 79.59 | 2.38 | 99.54 | 5.42 |
| HSPA4 | AB023420 | 107.08 | 7.66 | 77.64 | 6.97 |
| KCND3 | NM_004980 | 31.55 | 1.93 | 29.81 | 3.48 |
| NEDD4 | D42055 | 97.44 | 2.56 | 119.5 | 22.38 |
| POLD3 | D26018 | 111.54 | 10.41 | 114 | 14.59 |
| APEX1 | NM_001641 | 84.63 | 0.82 | 87.8 | 14.44 |
| PTPN2 | NM_002828 | 111.64 | 3.41 | 118.74 | 7.08 |
| FLJ31033 | AK055595 | 50.1 | 0.41 | 60.6 | 7.38 |
| MYLK | NM_053025 | 67.48 | 2.14 | 74.67 | 3.36 |
| DDX50 | NM_024045 | 77.43 | 8.55 | 32.36 | 8.26 |
| DDX39 | NM_005804 | 85.51 | 6.46 | 67.08 | 5.7 |
| TREX1 | BC002903 | 68.25 | 8.57 | 97.26 | 12.85 |
| RAD50 | NM_005732 | 104.45 | 10.39 | 102.95 | 11.66 |
| HACE1 | NM_020771 | 107.42 | 5.06 | 125.35 | 4.5 |
| PTPRO | NM_030667 | 117.91 | 2.81 | 100.04 | 5.61 |
| PMS2L4 | D38500 | 83.1 | 9.17 | 91.75 | 13.19 |
| MRE11A | NM_005591 | 106.3 | 0.82 | 120.49 | 4.75 |
| SKIP | NM_016532 | 84.92 | 12.53 | 100.85 | 14.18 |
| ANKK1 | NM_178510 | 87.92 | 2.59 | 107.51 | 7.32 |
| MAP4K4 | NM_145686 | 103.58 | 9.57 | 127.91 | 6.86 |
| ST8SIA4 | NM_005668 | 93.16 | 6.66 | 108.67 | 14.39 |
| XRCC4 | NM_022406 | 97.75 | 2.54 | 104.95 | 16.25 |
| FBXO10 | AF176705 | 61.43 | 1.29 | 41.22 | 16.12 |
| NR2E3 | NM_014249 | 69.68 | 1.46 | 95.53 | 17.13 |
| DLX4 | NM_138281 | 54.93 | 6.7 | 83.31 | 10.23 |
| NR6A1 | NM_033334 | 71.28 | 4.04 | 103.28 | 21.73 |
| DDX47 | NM_016355 | 97.34 | 0.94 | 123.19 | 12.32 |
| FGD2 | NM_173558 | 90.02 | 6.53 | 70.51 | 9.68 |
| KIAA2002 | AB082533 | 66.12 | 4.76 | 96.75 | 4.67 |
| PMS2L11 | D38437 | 112.76 | 1.5 | 112.64 | 4.43 |
| PAK7 | NM_020341 | 77.3 | 3.08 | 97.91 | 7.24 |
| APEG1 | AB037718 | 113.25 | 8.15 | 101.21 | 7.56 |
| POLR2J2 | NM_145325 | 103.92 | 9.94 | 111.76 | 5.27 |
| TTBK2 | NM_173500 | 86.31 | 15.3 | 55.46 | 4.52 |
| TRPV6 | NM_018646 | 93.2 | 10.05 | 130.14 | 13.97 |
| HDAC4 | NM_006037 | 70.78 | 1.59 | 44.4 | 8.86 |
| STK31 | NM_031414 | 110.7 | 3.82 | 117.93 | 10.86 |
| FBXO41 | AB075820 | 110.61 | 5.97 | 115.85 | 14.4 |
| FBXO27 | NM_178820 | 108.42 | 3.92 | 104.9 | 18.27 |
| PTPRA | NM_002836 | 94.99 | 2.55 | 116.86 | 12.08 |
| PLEKHG2 | AK024429 | 90.61 | 3.74 | 68.21 | 3.93 |
| FBXO15 | NM_152676 | 66.45 | 2.52 | 73.35 | 5.46 |
| KCNH1 | NM_172362 | 83.66 | 0.96 | 95.33 | 1.68 |
| DDX46 | NM_014829 | 85.09 | 7.18 | 92.35 | 24.42 |
| OCRL | NM_000276 | 72.17 | 1.83 | 89.01 | 21.12 |
| AKAP13 | NM_006738 | 80.42 | 6.35 | 87.51 | 19.76 |
| ZAP70 | NM_001079 | 67.08 | 3.59 | 93.19 | 13.39 |
| DDX31 | NM_022779 | 99.77 | 0.68 | 123.93 | 3.02 |
| HSPA8 | NM_006597 | 67.7 | 1.45 | 87.42 | 2.49 |
| KCNT1 | AB037843 | 73.96 | 3.28 | 93.74 | 6.33 |
| KCNQ4 | NM_004700 | 100.04 | 6.76 | 91.71 | 10.28 |
| TRAF4 | NM_004295 | 110.62 | 18.8 | 135.5 | 13.88 |
| OBSCN | AB046776 | 107.49 | 11.25 | 92.06 | 3.99 |
| PTPRC | NM_002838 | 95.97 | 3.13 | 136.73 | 16.45 |
| SMG1 | NM_015092 | 86.66 | 6.7 | 82.71 | 9.14 |
| PTPRD | NM_002839 | 113.24 | 8.51 | 128.67 | 12.91 |
| BIN1 | NM_139343 | 98.71 | 2.19 | 133.64 | 13.87 |
| PMS2L11 | U38980 | 67.83 | 1.18 | 65.06 | 4.08 |
| NSD1 | NM_022455 | 109.2 | 2.73 | 116.5 | 5.81 |
| PAXIP1 | NM_007349 | 113.07 | 5.74 | 115.41 | 15.42 |
| DDX23 | NM_004818 | 103.73 | 2.82 | 111.32 | 13.73 |
| DDX49 | NM_019070 | 85.9 | 0.2 | 75.61 | 4.14 |
| MAP3K6 | NM_004672 | 117.36 | 5.18 | 112.07 | 0.28 |
| HECW2 | AB037722 | 127.89 | 6.34 | 145.16 | 3.06 |
| ITSN2 | NM_006277 | 96.14 | 8.46 | 124.77 | 9.66 |
| MAST3 | AB011133 | 38.47 | 9.48 | 23.26 | 10.21 |
| SNF1LK2 | AB018324 | 76.66 | 4.81 | 102.73 | 10.56 |
| BRD4 | NM_058243 | 62.09 | 4.17 | 77.12 | 2.4 |
| RIOK3 | NM_003831 | 88.31 | 5.42 | 96.02 | 9.7 |
| PPARD | NM_006238 | 91.48 | 6.93 | 131.85 | 31.02 |
| PTP4A3 | NM_032611 | 97.46 | 2.92 | 85.22 | 7.45 |
| FBXO22 | AK026282 | 105.24 | 7.43 | 118.08 | 13.42 |
| HDAC9 | NM_058176 | 104.17 | 2.21 | 123.57 | 12.42 |
| CAMKK1 | NM_172207 | 93.04 | 8.2 | 100.9 | 10.21 |
| SLC25A37 | NM_016612 | 99.2 | 4.65 | 136.41 | 8.11 |
| IER3 | NM_052815 | 94.67 | 1.49 | 125.69 | 10.74 |
| HCN3 | AB040968 | 65.39 | 7.95 | 77.64 | 10.71 |
| YY1AP1 | NM_139118 | 97.7 | 11.41 | 108.43 | 20.15 |
| RORA | NM_134260 | 99.62 | 0.7 | 132.75 | 16.6 |
| PTPRF | NM_002840 | 105.7 | 11.21 | 104.11 | 17.27 |
| UBE3A | NM_000462 | 71.32 | 0.76 | 102.37 | 7.57 |
| FBXO11 | NM_025133 | 99.13 | 7.82 | 106.15 | 6.18 |
| SCYL3 | NM_020423 | 45.55 | 33.93 | 49.52 | 7.08 |
| RASGRF2 | AF023130 | 81.08 | 11.32 | 96.3 | 6.05 |
| MAP3K7 | NM_145331 | 73.39 | 1.13 | 97.71 | 0.03 |
| RIOK1 | NM_031480 | 70.58 | 7.15 | 70.69 | 2.9 |
| PTPN7 | NM_080588 | 96.13 | 1.01 | 138 | 14.48 |
| FBXO22 | NM_147188 | 65.8 | 6.31 | 89.05 | 1.95 |
| KCNH7 | NM_033272 | 106.15 | 5.11 | 123.25 | 12.08 |
| RASGRF1 | NM_002891 | 62.89 | 4.42 | 60.88 | 1.13 |
| ARID4A | NM_002892 | 46.85 | 31.51 | 94.56 | 11.89 |
| XRCC6BP1 | NM_033276 | 96.22 | 0.66 | 93.09 | 2.81 |
| MSH5 | NM_025259 | 95.51 | 0.82 | 108.27 | 3.07 |
| PDE5A | NM_001083 | 84.86 | 2.95 | 82.16 | 6.6 |
| NR4A3 | NM_173200 | 96.19 | 0.72 | 105.95 | 3.74 |
| PTPRN2 | NM_002847 | 97.17 | 6.49 | 136.94 | 22.45 |
| PTPRR | NM_002849 | 105.51 | 5.4 | 109.68 | 4.61 |
| KCNH2 | NM_000238 | 103.53 | 16.19 | 94.09 | 16.99 |
| ADCK4 | BC013114 | 87.25 | 1.4 | 87.53 | 5.33 |
| TERF1 | NM_003218 | 114.76 | 13.91 | 129.24 | 16.52 |
| WHSC1L1 | NM_023034 | 102 | 12.06 | 115.92 | 1.05 |
| PDE7A | NM_002603 | 100.68 | 3.19 | 103.03 | 5.79 |
| UBE1 | NM_003334 | 104.46 | 0.36 | 147.91 | 19.56 |
| C14orf130 | NM_175748 | 93.39 | 2.14 | 114.97 | 6.94 |
| ST6GALNAC4 | NM_014403 | 85.25 | 2.96 | 116.94 | 1.49 |
| YME1L1 | NM_139312 | 104.07 | 14.21 | 112.38 | 10.2 |
| CXXC6 | AF430147 | 105.92 | 4.06 | 121.3 | 2.91 |
| TRPV4 | NM_021625 | 113.06 | 2.26 | 141 | 23.56 |
| BAT1 | NM_004640 | 95.06 | 3.94 | 96.68 | 5.12 |
| CACNA1A | NM_023035 | 94.19 | 10.28 | 109.26 | 7.17 |
| INSRR | NM_014215 | 77.97 | 2.99 | 85.99 | 2.46 |
| PLEKHG5 | NM_020631 | 97.8 | 5.86 | 90.68 | 24.21 |
| CSF3R | NM_156039 | 108.14 | 8.35 | 114.73 | 9.52 |
| ADAR | NM_001111 | 87.19 | 12.09 | 79.64 | 4.55 |
| BACE1 | NM_012104 | 59.4 | 3.46 | 76.45 | 3.68 |
| PTPRS | NM_002850 | 69.35 | 7.27 | 72.03 | 0.74 |
| PSEN1 | NM_000021 | 67.87 | 2.76 | 77.96 | 12.26 |
| PTPRZ1 | NM_002851 | 95.49 | 7.1 | 91.59 | 3.52 |
| DYRK1B | NM_004714 | 56.3 | 1.3 | 48.69 | 7.61 |
| BIRC7 | NM_139317 | 49.82 | 0.6 | 60.98 | 10.01 |
| CTDP1 | NM_004715 | 52.38 | 7.54 | 68.62 | 13.44 |
| KCNH5 | NM_139318 | 100.6 | 10.71 | 126.57 | 6.87 |
| PRPF4B | NM_003913 | 89.46 | 3.5 | 69.21 | 6.09 |
| MINK1 | NM_153827 | 79.45 | 9.09 | 84.21 | 3.86 |
| ARHGEF7 | NM_145735 | 90.39 | 0.65 | 55.85 | 0.95 |
| DDX4 | NM_024415 | 88.84 | 0.14 | 117.48 | 17.72 |
| CAMK1D | NM_153498 | 88.89 | 6.67 | 95.62 | 1.24 |
| PTPRT | NM_133170 | 100.18 | 4.45 | 104.81 | 27.22 |
| ZA20D1 | NM_020205 | 70.93 | 2.2 | 99.45 | 4.88 |
| MAST1 | AB023190 | 104.79 | 7.94 | 128.37 | 10.98 |
| MARK2 | NM_017490 | 103.32 | 3.13 | 103.58 | 2.98 |
| GLI2 | NM_030379 | 75.78 | 3.47 | 85.33 | 3.09 |
| KIAA0446 | AB007915 | 53.84 | 0.92 | 49.7 | 3.49 |
| TREX1 | NM_130384 | 93.98 | 6.8 | 69.15 | 7.84 |
| FBXW11 | NM_012300 | 81.96 | 1.23 | 91.32 | 16.88 |
| STK32C | NM_173575 | 82 | 0.02 | 64.51 | 5.23 |
| WWOX | NM_016373 | 104.22 | 10.23 | 101.17 | 0.83 |
| RAD51L1 | NM_133509 | 84.3 | 2.92 | 78.68 | 13.8 |
| PTPN22 | NM_015967 | 58.75 | 9.63 | 80.73 | 16.77 |
| RAD51C | NM_058216 | 93.51 | 1.53 | 86.11 | 2.52 |
| KCNC2 | NM_139137 | 73.54 | 0.39 | 67.75 | 0.94 |
| DDX51 | NM_175066 | 70.99 | 0.43 | 67.97 | 2.28 |
| TRAF3IP2 | AF136408 | 94.56 | 0.87 | 114.21 | 3.43 |
| FASTK | NM_006712 | 76.94 | 1.36 | 69.69 | 3.74 |
| DDX42 | NM_007372 | 68.48 | 1.08 | 67.55 | 7.49 |
| FBXL5 | NM_012161 | 100.19 | 13.48 | 112.56 | 4.96 |
| FBXL6 | NM_012162 | 93.58 | 5.57 | 105.45 | 2.71 |
| MAP2K5 | NM_145161 | 92.21 | 2.44 | 83.12 | 2.24 |
| LIG3 | NM_013975 | 81.37 | 0.6 | 90 | 5.25 |
| ST6GAL1 | NM_173216 | 100.7 | 9.21 | 138.47 | 11.52 |
| HDAC8 | NM_018486 | 93.9 | 3.96 | 121.25 | 20.14 |
| KCNG4 | NM_172347 | 94.34 | 4.36 | 103.47 | 13.88 |
| KCNG3 | NM_133329 | 114.8 | 1.1 | 123.72 | 12.83 |
| KCNH3 | AB033108 | 96.29 | 10.36 | 117.84 | 15.5 |
| WNK2 | NM_006648 | 85.05 | 5.39 | 112.78 | 14.83 |
| KCNH6 | NM_030779 | 83.89 | 7.99 | 86.64 | 8.13 |
| UBR1 | NM_174916 | 87.89 | 12.78 | 115.25 | 10.14 |
| C9orf96 | BC036504 | 68.89 | 11.71 | 97.67 | 10.08 |
| SLC25A28 | NM_031212 | 89.47 | 5.54 | 110.91 | 9.39 |
| DDX52 | NM_007010 | 97.07 | 3.67 | 104.08 | 1.34 |
| CHEK1 | NM_001274 | 104.16 | 2.76 | 75.57 | 2.61 |
| RRM1 | NM_001033 | 66.19 | 3.48 | 31.32 | 2.55 |
| BRCA1 | NM_007296 | 88.11 | 2.55 | 86.59 | 1.9 |
| SENP7 | NM_020654 | 106.07 | 0.03 | 123.71 | 14.24 |
| SLFN5 | NM_144975 | 94.27 | 9.19 | 84.76 | 8.75 |
| HSPA1A | NM_005345 | 91 | 4.41 | 111.98 | 5.76 |
| BRSK2 | NM_003957 | 48.65 | 0.72 | 73.65 | 11.89 |
| BIRC2 | NM_001166 | 92.62 | 11.76 | 84.32 | 9.31 |
| TRPA1 | NM_007332 | 93.98 | 3.43 | 76.39 | 0.68 |
| BIRC4 | NM_001167 | 92.42 | 7.74 | 87.91 | 6.68 |
| TOPORS | NM_005802 | 19.07 | 6.68 | 38.74 | 2.97 |
| HSPA1B | NM_005346 | 96.49 | 1.98 | 136.31 | 2.17 |
| RNF8 | NM_003958 | 87.45 | 4.91 | 67.21 | 11.82 |
| ERCC2 | NM_000400 | 51.1 | 3.94 | 28.34 | 1.96 |
| NR0B1 | NM_000475 | 95.35 | 5.5 | 108.95 | 11.07 |
| TPCN2 | NM_139075 | 60.92 | 7.19 | 57.72 | 6.01 |
| CIDEA | NM_001279 | 71.36 | 5.37 | 61.05 | 4.18 |
| HSPA5 | NM_005347 | 81.29 | 3.2 | 92.99 | 2.95 |
| PNPT1 | NM_033109 | 68.42 | 1.26 | 72.98 | 4.43 |
| SOCS1 | NM_003745 | 80.72 | 3.96 | 90.23 | 2.12 |
| IRAK4 | NM_016123 | 72.81 | 5.91 | 106.3 | 2.2 |
| DLX1 | NM_178120 | 104.83 | 2.46 | 117.58 | 4.1 |
| SMPD3 | NM_018667 | 75.86 | 4.96 | 78.15 | 8.04 |
| RAD23B | NM_002874 | 84.14 | 5.42 | 109.5 | 10.22 |
| DDX54 | NM_024072 | 34.53 | 2.69 | 36.8 | 2.45 |
| SMO | NM_005631 | 93.34 | 9.59 | 97.7 | 3.25 |
| PHKG2 | NM_000294 | 82.48 | 6.94 | 77.55 | 7.36 |
| POLR1A | NM_015425 | 36.27 | 1.28 | 28.26 | 0.06 |
| FLJ20433 | NM_017820 | 81.59 | 6.88 | 93.16 | 11.3 |
| RPS6KL1 | NM_031464 | 117.2 | 1.06 | 135.56 | 1.67 |
| SLC25A23 | NM_024103 | 82.81 | 6.83 | 79.77 | 10.53 |
| DDX27 | NM_017895 | 89.69 | 28.53 | 93.55 | 1.35 |
| NR1H2 | NM_007121 | 62.6 | 2.91 | 109.37 | 7.09 |
| SENP1 | NM_014554 | 108.42 | 2.36 | 101.53 | 2.3 |
| PDE11A | NM_016953 | 99.47 | 1.98 | 96.93 | 15.69 |
| APEX2 | NM_014481 | 94.61 | 3.83 | 71.76 | 1.98 |
| CGI-69 | NM_016016 | 71.66 | 10.28 | 66.6 | 8.46 |
| TRPM5 | NM_014555 | 63.38 | 14.72 | 36.65 | 1.78 |
| EIF2AK1 | NM_014413 | 91.31 | 12.04 | 73.52 | 0.03 |
| STAMBP | NM_006463 | 53.02 | 0.8 | 53.57 | 2.15 |
| MTCH1 | NM_014341 | 76.33 | 0.18 | 87.12 | 4.91 |
| HIPK3 | NM_005734 | 76.76 | 9.7 | 68.74 | 10.72 |
| GNRHR | NM_000406 | 82.12 | 6.49 | 90.95 | 1.22 |
| KIAA1706 | NM_030636 | 110.34 | 2.41 | 87.84 | 0.56 |
| SCN4A | NM_000334 | 77.2 | 0.69 | 91.08 | 1.61 |
| ARHGEF9 | NM_015185 | 63.83 | 7.56 | 59.63 | 12.83 |
| SCN5A | NM_000335 | 99.45 | 0.22 | 105.86 | 7.22 |
| PARP4 | NM_006437 | 63.34 | 1.96 | 67.43 | 4.07 |
| PTCH | NM_000264 | 84.47 | 7.13 | 102.73 | 11.6 |
| FTS | NM_022476 | 98.38 | 3.72 | 90.81 | 12.49 |
| SHH | NM_000193 | 89.35 | 9.04 | 83.46 | 1.29 |
| LATS1 | NM_004690 | 81.37 | 2.73 | 106.91 | 10.11 |
| DDX28 | NM_018380 | 84.48 | 3.55 | 103.65 | 15.74 |
| PKD2L2 | NM_014386 | 76.28 | 4.61 | 40.75 | 5.43 |
| DISP1 | NM_032890 | 58.87 | 9.13 | 80.98 | 7.3 |
| ITPR1 | NM_002222 | 90.2 | 2.07 | 78.53 | 2 |
| UBE2Q1 | NM_017582 | 72.89 | 3.1 | 87.85 | 2.3 |
| ITSN1 | NM_003024 | 96.71 | 0.59 | 70.16 | 13.16 |
| MTCH2 | NM_014342 | 112.51 | 6.82 | 79.71 | 2.55 |
| VCP | NM_007126 | 28.57 | 2.26 | 23.26 | 0.42 |
| ARHGEF10 | NM_014629 | 92.71 | 6.39 | 114.38 | 5.8 |
| BCL10 | NM_003921 | 76.83 | 1.91 | 89.7 | 4.4 |
| MOCS3 | NM_014484 | 98.47 | 9.51 | 108.09 | 5.23 |
| PASK | NM_015148 | 90.06 | 2.76 | 89.94 | 0.79 |
| POLR3F | NM_006466 | 79.46 | 4.03 | 79.95 | 7.95 |
| MCF2L | NM_024979 | 108.29 | 8.76 | 103.28 | 7.2 |
| MUSK | NM_005592 | 61.7 | 0.49 | 65.97 | 4.83 |
| HERC1 | NM_003922 | 107.09 | 0.55 | 112.52 | 2.6 |
| ATG7 | NM_006395 | 108.11 | 0.33 | 131.25 | 10.32 |
| HSPA1L | NM_005527 | 109.53 | 3.87 | 114.6 | 15.17 |
| CACNA1D | NM_000720 | 96.8 | 5.99 | 112.19 | 20.28 |
| MCF2L2 | NM_015078 | 97.79 | 6.64 | 110.69 | 1.83 |
| KCNS1 | NM_002251 | 78.15 | 2.03 | 64.88 | 1.44 |
| TSSK1 | NM_032028 | 68.74 | 6.03 | 62.64 | 0.87 |
| ITPR3 | NM_002224 | 84.13 | 9.11 | 99.14 | 2.27 |
| PIN1 | NM_006221 | 76.75 | 6.33 | 73.49 | 0.57 |
| HSPA6 | NM_002155 | 107.03 | 13.01 | 98.67 | 26.63 |
| DDX58 | NM_014314 | 58.47 | 1.95 | 68.1 | 13.06 |
| SYNJ1 | NM_003895 | 72.16 | 1.83 | 62 | 11.86 |
| TRPC6 | NM_004621 | 64.17 | 17.29 | 66.01 | 3.6 |
| UBA2 | NM_005499 | 84.56 | 17.36 | 96.8 | 0.23 |
| UBE2D4 | NM_015983 | 42.34 | 2.06 | 66.26 | 6.38 |
| UBE1C | NM_003968 | 88.31 | 4.93 | 95.64 | 7.09 |
| RNF7 | NM_014245 | 72.4 | 2.6 | 84.78 | 4.63 |
| UBE2M | NM_003969 | 71.83 | 8.91 | 86.2 | 12.08 |
| ST6GALNAC6 | NM_013443 | 96.23 | 5.69 | 117.19 | 2.69 |
| ST3GAL5 | NM_003896 | 74.87 | 5.99 | 85.1 | 1.19 |
| NR5A2 | NM_003822 | 71.2 | 0.05 | 69.32 | 6.51 |
| AMPD3 | NM_000480 | 102.37 | 7.6 | 72.03 | 5.49 |
| TRIM24 | NM_003852 | 93.95 | 0.1 | 107.3 | 7.83 |
| GUCY2F | NM_001522 | 87.72 | 8.07 | 87.51 | 17.87 |
| DDX21 | NM_004728 | 53.06 | 2.08 | 65.99 | 1.26 |
| PRSS15 | NM_004793 | 116.78 | 13.02 | 143.75 | 0.18 |
| IRAK3 | NM_007199 | 112.05 | 6.15 | 130.77 | 10.87 |
| HSPA4L | NM_014278 | 95.08 | 3.28 | 106.17 | 2.09 |
| KCNS3 | NM_002252 | 107.68 | 1.93 | 112.55 | 1.84 |
| RAD9A | NM_004584 | 106.74 | 9.29 | 82.44 | 3.72 |
| DDX1 | NM_004939 | 116.61 | 2.47 | 119.94 | 0.54 |
| PDE6A | NM_000440 | 67.77 | 13.27 | 71.15 | 9.38 |
| CACNA1E | NM_000721 | 128.73 | 7.24 | 144.49 | 1.86 |
| CSNK1G2 | NM_001319 | 86.23 | 10.87 | 73.47 | 12.54 |
| VPS4B | NM_004869 | 46.89 | 0.34 | 69.41 | 4.48 |
| TPCN1 | NM_017901 | 67.71 | 0.81 | 50.55 | 4.78 |
| MCFP | NM_018843 | 99.2 | 8.08 | 78.64 | 5.69 |
| TP53BP2 | NM_005426 | 87.01 | 0.82 | 86.19 | 5.79 |
| SYNJ2 | NM_003898 | 109.69 | 7.55 | 74.24 | 2.99 |
| PNLIPRP1 | NM_006229 | 67.17 | 6.29 | 46.8 | 0.46 |
| GEFT | NM_133483 | 65.91 | 3.41 | 70.32 | 6.57 |
| UBE2T | NM_014176 | 63.2 | 0.07 | 59.53 | 2.73 |
| CLCN7 | NM_001287 | 65 | 0.85 | 90.92 | 1.52 |
| XPC | NM_004628 | 69.77 | 0.28 | 71.99 | 0.85 |
| MGC16169 | NM_033115 | 103.45 | 7.28 | 118.11 | 12.41 |
| UBE2J2 | NM_058167 | 90.39 | 2.83 | 73.62 | 6.75 |
| PRKAG2 | NM_016203 | 100.32 | 5.91 | 102.29 | 3.6 |
| FANCG | NM_004629 | 57.48 | 1.13 | 20.9 | 0.3 |
| DPP7 | NM_013379 | 57.31 | 1.83 | 84.64 | 3.08 |
| BMSC-MCP | NM_032315 | 101.45 | 12.18 | 103.54 | 7.83 |
| CA6 | NM_001215 | 65.23 | 2.69 | 73.77 | 2.75 |
| FBXO34 | NM_017943 | 109.62 | 1.03 | 118.55 | 4.24 |
| HUWE1 | NM_031407 | 49.07 | 6.7 | 66.88 | 4.73 |
| FBXL15 | NM_024326 | 95.04 | 5.04 | 108.59 | 9.15 |
| DDX20 | NM_007204 | 114.29 | 6.7 | 136.99 | 22.48 |
| MUS81 | NM_025128 | 103.24 | 9.89 | 103.74 | 16.45 |
| PIB5PA | NM_014422 | 94.65 | 1.57 | 110.15 | 4.97 |
| SKIV2L2 | NM_015360 | 111.6 | 7.24 | 98.21 | 10.78 |
| LGP2 | NM_024119 | 98.11 | 9.13 | 127.05 | 5.12 |
| FANCE | NM_021922 | 105.01 | 1.09 | 98.23 | 11.92 |
| TRIB2 | NM_021643 | 100.5 | 0.65 | 57.26 | 50.93 |
| FANCF | NM_022725 | 105.17 | 8.26 | 82.88 | 9.03 |
| PPP3CC | NM_005605 | 98.9 | 7.6 | 82.82 | 5.09 |
| SUFU | NM_016169 | 98.92 | 10.6 | 81.2 | 1.6 |
| FBXO36 | NM_174899 | 81.59 | 11.04 | 94.41 | 6.73 |
| BCL2L13 | NM_015367 | 66.4 | 9.47 | 47.11 | 5.09 |
| POLR2D | NM_004805 | 42.84 | 5.24 | 20.71 | 10.26 |
| DNASE1L3 | NM_004944 | 121.14 | 1.14 | 138.55 | 0.57 |
| RXRA | NM_002957 | 68.88 | 0.28 | 35.74 | 2.38 |
| PIK3R4 | NM_014602 | 56.42 | 36.39 | 112.95 | 5.37 |
| NP | NM_000270 | 54.27 | 4.72 | 71.06 | 3.07 |
| TRPM8 | NM_024080 | 95.97 | 4.83 | 116.9 | 5.2 |
| STAR | NM_000349 | 56.82 | 2.38 | 45.44 | 0.29 |
| DDX19B | NM_007242 | 97.77 | 7.79 | 122.58 | 3.05 |
| TRPM6 | NM_017662 | 70.44 | 12.88 | 100.92 | 5.69 |
| FLJ20551 | NM_017875 | 95.17 | 4.73 | 103.58 | 8.24 |
| MAP3K12 | NM_006301 | 59.96 | 10.43 | 71.82 | 2.75 |
| SARS | NM_006513 | 36.56 | 18.31 | 62.89 | 4.66 |
| DDX59 | NM_031306 | 73.03 | 10.01 | 101.28 | 1.24 |
| SCN10A | NM_006514 | 114.08 | 6.09 | 149.9 | 24.14 |
| SAE1 | NM_005500 | 66.89 | 0.64 | 92.85 | 7.27 |
| TESK2 | NM_007170 | 98.89 | 2.75 | 124.5 | 8.15 |
| TSSK6 | NM_032037 | 113.12 | 1.9 | 145.77 | 3.84 |
| HERC3 | NM_014606 | 61.91 | 10.35 | 40.12 | 2.8 |
| DDX3Y | NM_004660 | 76.74 | 3.02 | 86.74 | 1.14 |
| BAG4 | NM_004874 | 84.42 | 5.44 | 79.16 | 4.64 |
| MCRS1 | NM_006337 | 91.02 | 5.62 | 98.65 | 10.84 |
| POLR1C | NM_004875 | 58.65 | 9.34 | 89.67 | 2.38 |
| RAD21 | NM_006265 | 34.59 | 4.84 | 43.03 | 3.6 |
| ACAA1 | NM_001607 | 35.9 | 2.59 | 26.91 | 2.02 |
| PDK3 | NM_005391 | 60.63 | 5.7 | 92.19 | 8.46 |
| EI24 | NM_004879 | 64.81 | 2.44 | 59.94 | 1.11 |
| INPP5A | NM_005539 | 108.15 | 6.15 | 109.73 | 10.99 |
| XRCC6 | NM_001469 | 112.61 | 2.77 | 99.3 | 2.96 |
| PMS2L1 | NM_005394 | 52.69 | 6.05 | 47.55 | 1.33 |
| POLR3K | NM_016310 | 69.22 | 0.95 | 71.71 | 4.63 |
| PMS2L3 | NM_005395 | 55.5 | 1 | 64.02 | 4.5 |
| REV1L | NM_016316 | 71.04 | 9.48 | 81.61 | 7.06 |
| MTA2 | NM_004739 | 91.15 | 7.26 | 82.68 | 1.3 |
| FANCD2 | NM_033084 | 63.9 | 6.48 | 70.92 | 4.69 |
| POLD2 | NM_006230 | 82.02 | 2.98 | 87.01 | 1.64 |
| ARHGEF6 | NM_004840 | 112.53 | 2.79 | 128.55 | 5.5 |
| HECW1 | NM_015052 | 75.9 | 3.38 | 82.85 | 14.71 |
| STARD5 | NM_030574 | 81.11 | 6.77 | 77 | 15.27 |
| BMP2K | NM_017593 | 54.29 | 4.73 | 62 | 3.31 |
| KCNB2 | NM_004770 | 99.21 | 20.03 | 100.72 | 27.93 |
| POLR2H | NM_006232 | 57.31 | 0.73 | 57.23 | 1.61 |
| DCLRE1C | NM_022487 | 147.18 | 3.79 | 163.93 | 15.26 |
| XRCC2 | NM_005431 | 47.33 | 3.92 | 54.5 | 7.82 |
| ST8SIA1 | NM_003034 | 103.93 | 7.65 | 115.14 | 14.89 |
| POLR2I | NM_006233 | 43.91 | 0.95 | 44.85 | 8 |
| KCNA3 | NM_002232 | 106.72 | 6.73 | 115.09 | 0.09 |
| TCF19 | NM_007109 | 88.78 | 0.34 | 94.77 | 3.78 |
| KCNA4 | NM_002233 | 124.17 | 0.18 | 107.16 | 5.76 |
| POLR2J | NM_006234 | 83.94 | 6.41 | 91.22 | 0.47 |
| KCNA5 | NM_002234 | 108.71 | 1.39 | 88.74 | 8.83 |
| ALPK3 | NM_020778 | 72.55 | 0.48 | 100.21 | 11.37 |
| MDC1 | NM_014641 | 61.73 | 4.68 | 26.49 | 14.29 |
| HERC2 | NM_004667 | 93.65 | 7.23 | 105.9 | 1 |
| PNLIPRP2 | NM_005396 | 97.63 | 5.84 | 109.98 | 0.12 |
| SLC25A18 | NM_031481 | 108.03 | 6.3 | 126.91 | 1.82 |
| XPA | NM_000380 | 88.44 | 2.74 | 84.07 | 4.12 |
| ITCH | NM_031483 | 115.98 | 13.09 | 108.08 | 9.65 |
| NEK8 | NM_178170 | 88.27 | 1.6 | 81.37 | 12.37 |
| SCYL2 | NM_017988 | 109.76 | 4.24 | 116.03 | 9.07 |
| HNF4A | NM_000457 | 82.83 | 2.04 | 99.32 | 8.17 |
| HSPCB | NM_007355 | 83.93 | 2.02 | 76.36 | 4.29 |
| FBXL13 | NM_145032 | 94.73 | 7.04 | 97.22 | 5.86 |
| TEK | NM_000459 | 106.16 | 9 | 115.47 | 7.23 |
| SMURF2 | NM_022739 | 94.69 | 1.76 | 47.48 | 9.04 |
| XRCC3 | NM_005432 | 99.12 | 0.51 | 112.36 | 12.11 |
| KCNA6 | NM_002235 | 92.11 | 1.64 | 109.13 | 1.79 |
| KCNF1 | NM_002236 | 63 | 2.2 | 62.77 | 3.07 |
| PTPN21 | NM_007039 | 84.07 | 1.46 | 114.5 | 1.36 |
| ESR2 | NM_001437 | 77.51 | 12.08 | 37.09 | 7.6 |
| ROR2 | NM_004560 | 74.49 | 0.92 | 96.29 | 5.24 |
| ESRRG | NM_001438 | 94.96 | 2.32 | 88.36 | 3.64 |
| ARHGEF5 | NM_005435 | 61.33 | 4.85 | 71.54 | 4.81 |
| HEL308 | NM_133636 | 126.12 | 2.34 | 119.74 | 6 |
| APPBP1 | NM_003905 | 71.65 | 0.57 | 81.49 | 9.15 |
| CNGB1 | NM_001297 | 100.25 | 2.28 | 99.74 | 3.93 |
| UBE2Z | NM_023079 | 83.13 | 13.68 | 102.39 | 29.52 |
| CNGA3 | NM_001298 | 80.48 | 4.94 | 78.14 | 12.03 |
| RDM1 | NM_145654 | 96.78 | 0.94 | 106.83 | 8.7 |
| SLC30A7 | NM_133496 | 124.37 | 11.12 | 136.44 | 2.28 |
| HERC6 | NM_017912 | 78.42 | 2.95 | 71.93 | 8.79 |
| PTK9L | NM_007284 | 78.44 | 9.6 | 79.44 | 7.87 |
| PXK | NM_017771 | 96.05 | 6.03 | 101.97 | 4.11 |
| SPHK2 | NM_020126 | 43.37 | 3 | 59.84 | 2.98 |
| FBXO30 | NM_032145 | 96.92 | 10.29 | 79.17 | 24.06 |
| TRPC4 | NM_016179 | 113.85 | 21.7 | 90.32 | 6.99 |
| ANGEL1 | NM_015305 | 101.63 | 7.52 | 107.49 | 12.97 |
| HSPB2 | NM_001541 | 56.35 | 0.12 | 52.42 | 3.31 |
| MARK4 | NM_031417 | 29.57 | 1.05 | 18.95 | 0.01 |
| FRAP1 | NM_004958 | 84.57 | 9.98 | 81.72 | 6.33 |
| RBBP4 | NM_005610 | 86.44 | 3.92 | 88.63 | 4.76 |
| MKI67 | NM_002417 | 72.44 | 6.82 | 66 | 5.97 |
| PMPCA | NM_015160 | 70.2 | 7.16 | 92.01 | 1.04 |
| NR5A1 | NM_004959 | 94.98 | 1.94 | 100.46 | 1.84 |
| POLG2 | NM_007215 | 94.55 | 10.11 | 88.19 | 21.53 |
| PARP1 | NM_001618 | 58.85 | 3.39 | 40.98 | 3.16 |
| DLC1 | NM_006094 | 71.72 | 14.41 | 84.06 | 0.65 |
| KCNV2 | NM_133497 | 110.99 | 2.34 | 99.91 | 3.8 |
| STK16 | NM_003691 | 105.95 | 7.43 | 129.87 | 8.64 |
| ARHGEF3 | NM_019555 | 122.89 | 7.1 | 107.14 | 0.29 |
| MCF2 | NM_005369 | 50.9 | 2.68 | 72.69 | 2.8 |
| PSKH2 | NM_033126 | 114.53 | 5.49 | 87.08 | 37.41 |
| SLC25A5 | NM_001152 | 87.71 | 1.16 | 110.08 | 16.17 |
| UBXD8 | NM_014613 | 62.98 | 7.55 | 89.84 | 8.73 |
| PEA15 | NM_003768 | 70.33 | 11.8 | 110.89 | 18.5 |
| WNT6 | NM_006522 | 106.27 | 3.22 | 129.17 | 9.97 |
| TCOF1 | NM_000356 | 72.86 | 3.35 | 86.7 | 3.33 |
| POLK | NM_016218 | 96.27 | 0.8 | 85.12 | 6.62 |
| PDE6B | NM_000283 | 45.5 | 3.65 | 67.56 | 4.12 |
| STK32A | NM_145001 | 100.27 | 7.1 | 92.41 | 10.44 |
| UBE2R2 | NM_017811 | 68.82 | 5.4 | 89.94 | 0.76 |
| UCP1 | NM_021833 | 76.11 | 7.41 | 47.15 | 1.11 |
| UTP11L | NM_016037 | 61.23 | 2.79 | 40.98 | 1.95 |
| CXXC4 | NM_025212 | 72.9 | 6.01 | 91.17 | 2.91 |
| NR1D1 | NM_021724 | 81.81 | 2 | 86.76 | 5.53 |
| BRSK1 | NM_032430 | 66.32 | 2.86 | 70.48 | 11.1 |
| FBXO16 | NM_172366 | 98.6 | 5.34 | 109.61 | 2.18 |
| HERC5 | NM_016323 | 92.2 | 7.94 | 87.3 | 6.44 |
| SLC30A9 | NM_006345 | 105.84 | 6.02 | 102.05 | 3.91 |
| ALPK1 | NM_025144 | 52.98 | 7.14 | 49.02 | 1.53 |
| S100B | NM_006272 | 100.79 | 6.58 | 99.64 | 2.05 |
| SLK | NM_014720 | 61.83 | 0.44 | 20.54 | 0.72 |
| EDG1 | NM_001400 | 110.7 | 15.19 | 127.54 | 5.18 |
| BIRC6 | NM_016252 | 68.3 | 15.45 | 38.61 | 36.02 |
| RPS6KA4 | NM_003942 | 115.4 | 8.15 | 116.36 | 7.45 |
| HIPK2 | NM_022740 | 98.02 | 6.2 | 99.55 | 3.62 |
| PTPN18 | NM_014369 | 60.64 | 6.39 | 89.96 | 8.56 |
| STK33 | NM_030906 | 99.42 | 1.22 | 92.97 | 0.53 |
| PEX6 | NM_000287 | 109.22 | 5.82 | 92.02 | 4.59 |
| PLB1 | NM_153021 | 99.42 | 12.69 | 81.29 | 0.57 |
| TSSK4 | NM_174944 | 88.73 | 8.86 | 114.11 | 5.35 |
| PARP9 | NM_031458 | 57.02 | 3.32 | 51.15 | 6.86 |
| SPATA13 | NM_153023 | 63.8 | 8.74 | 43.97 | 38.91 |
| KATNAL1 | NM_032116 | 92.27 | 4.19 | 106.88 | 39.1 |
| TERF2 | NM_005652 | 133.37 | 8.88 | 138.75 | 1.6 |
| TRPM7 | NM_017672 | 95.09 | 5.5 | 122.47 | 1.76 |
| NR2F1 | NM_005654 | 69.71 | 0.37 | 69.07 | 2.18 |
| FBXL18 | NM_024963 | 89.66 | 7.75 | 116.32 | 2.22 |
| ST6GALNAC2 | NM_006456 | 86.57 | 0.9 | 84.85 | 2.02 |
| PREX1 | NM_020820 | 78.45 | 0.44 | 104.07 | 1.64 |
| NEDD4L | NM_015277 | 98.8 | 7.01 | 63.55 | 7.89 |
| FGD4 | NM_139241 | 102.02 | 0.2 | 109.59 | 0.32 |
| ZFP36L1 | NM_004926 | 104.79 | 1 | 100.48 | 2.95 |
| KCNA10 | NM_005549 | 118.34 | 6.83 | 142.3 | 0.84 |
| NR2F2 | NM_021005 | 112.81 | 5.65 | 128.24 | 9.67 |
| ST3GAL4 | NM_006278 | 94.59 | 6.34 | 104.94 | 5.06 |
| STARD8 | NM_014725 | 83.91 | 2.41 | 109.52 | 1.38 |
| NOL3 | NM_003946 | 86.39 | 0.59 | 105.94 | 2.74 |
| CNOT6 | NM_015455 | 10.13 | 0.8 | 6.41 | 0.78 |
| HCN4 | NM_005477 | 78.67 | 0.6 | 87.95 | 7.46 |
| SCN2A2 | NM_021007 | 52.26 | 1.63 | 48.41 | 2.58 |
| KALRN | NM_003947 | 110.25 | 3.74 | 105.69 | 1.35 |
| MLL4 | NM_014727 | 69.59 | 0.06 | 69.9 | 9.44 |
| SOCS2 | NM_003877 | 110.33 | 5.73 | 89.34 | 7.65 |
| MAK | NM_005906 | 93.33 | 12.47 | 86.94 | 1.1 |
| THRB | NM_000461 | 95.63 | 8.47 | 107.95 | 4.73 |
| TRIM28 | NM_005762 | 56.04 | 10.29 | 74.18 | 20.31 |
| CFLAR | NM_003879 | 56.5 | 6.93 | 58.22 | 4.13 |
| TRPM1 | NM_002420 | 101.2 | 8.03 | 86.99 | 49.97 |
| KATNA1 | NM_007044 | 109.66 | 6.88 | 136.56 | 3.38 |
| TRAF1 | NM_005658 | 79.69 | 0.4 | 102.85 | 7.47 |
| TRIO | NM_007118 | 116.68 | 7.8 | 107.01 | 1.72 |
| SPG7 | NM_003119 | 74.73 | 5.56 | 100.07 | 5.39 |
| SENP2 | NM_021627 | 87.98 | 8.39 | 70.84 | 2.51 |
| HYOU1 | NM_006389 | 70.27 | 5.2 | 87.67 | 0.23 |
| STARD6 | NM_139171 | 101.55 | 6.95 | 102.43 | 3.35 |
| TNK1 | NM_003985 | 86.87 | 7.1 | 85.73 | 0.51 |
| SCN8A | NM_014191 | 94.44 | 0.08 | 86.47 | 1.84 |
| KCNK3 | NM_002246 | 103.12 | 4.3 | 138.26 | 13.97 |
| SCYL1 | NM_020680 | 90.11 | 13.07 | 106.28 | 0.87 |
| NRBP | NM_013392 | 97.59 | 1.17 | 83.36 | 6.28 |
| KCNMA1 | NM_002247 | 104.28 | 3.6 | 120.98 | 9.4 |
| ADCY4 | NM_139247 | 117.25 | 3.57 | 124.88 | 0.1 |
| CACNA1B | NM_000718 | 69.55 | 2.85 | 28.82 | 2.02 |
| HCN2 | NM_001194 | 95.38 | 2.83 | 107.68 | 11.29 |
| SLC25A29 | NM_152333 | 84.73 | 2.75 | 80.05 | 7.14 |
| BARD1 | NM_000465 | 88.36 | 1.68 | 104.48 | 3.28 |
| CCRN4L | NM_012118 | 78.33 | 6.78 | 57.97 | 10.66 |
| PEX1 | NM_000466 | 102.11 | 10.94 | 104.92 | 2.93 |
| NR1H3 | NM_005693 | 95.71 | 2.72 | 57.67 | 7.33 |
| PKD2L1 | NM_016112 | 121.68 | 6.98 | 110.7 | 3.62 |
| FARP1 | NM_005766 | 121.72 | 9.77 | 102.11 | 8.18 |
| TRPV2 | NM_016113 | 86.15 | 2.01 | 107.22 | 1.63 |
| SMPD2 | NM_003080 | 39 | 5.6 | 39.36 | 4.56 |
| ARHGEF12 | NM_015313 | 98.9 | 6.72 | 106.85 | 9.96 |
| CDKL5 | NM_003159 | 77.83 | 6.75 | 101.72 | 10.42 |
| SPATA5L1 | NM_024063 | 111.91 | 1.91 | 133.38 | 6.03 |
| SLC30A2 | NM_032513 | 22.83 | 3.3 | 14.07 | 0.43 |
| ARHGEF18 | NM_015318 | 97.62 | 1.21 | 129.96 | 11.67 |
| BOK | NM_032515 | 81.35 | 7.77 | 94.89 | 2.57 |
| LIPH | NM_139248 | 100.41 | 3.93 | 100.88 | 12.74 |
| DNASE1L2 | NM_001374 | 56.18 | 8.48 | 69.34 | 2.93 |
| ACYP2 | NM_138448 | 96.39 | 6.51 | 92.35 | 6.53 |
| CACNA1C | NM_000719 | 76.99 | 9.62 | 71.55 | 1.29 |
| NSF | NM_006178 | 66.48 | 2.8 | 73.24 | 2.93 |
| DNMT1 | NM_001379 | 87.96 | 14.3 | 71.15 | 12.75 |
| DYRK4 | NM_003845 | 50.96 | 10.36 | 51.14 | 2.01 |
| ATCAY | NM_033064 | 78.33 | 19.58 | 73.98 | 5.91 |
| LOC221955 | NM_139179 | 103.35 | 8.74 | 122.57 | 2.32 |
| TAOK2 | NM_016151 | 91.77 | 4.87 | 116.51 | 5.33 |
| SCN7A | NM_002976 | 88.31 | 10.68 | 97.53 | 5.82 |
| SNF1LK | NM_173354 | 61.77 | 2.84 | 64.93 | 9.93 |
| BIRC3 | NM_001165 | 106.44 | 8.4 | 121.45 | 7.52 |
| SLC30A6 | NM_017964 | 113.47 | 6.49 | 100.91 | 7.53 |
| SCN9A | NM_002977 | 81.33 | 2.28 | 99.65 | 2.74 |
| PINK1 | NM_032409 | 97.07 | 3.14 | 91.38 | 4.09 |
| STK23 | NM_014370 | 89.71 | 5.8 | 101.29 | 9.65 |
| PDE7B | NM_018945 | 78.29 | 6.73 | 73.39 | 0.8 |
| STK3 | NM_006281 | 109.62 | 2.69 | 109.33 | 1.62 |
| RFC5 | NM_007370 | 76.59 | 4.29 | 64.34 | 14.34 |
| FBXO28 | NM_015176 | 106.94 | 0.33 | 111.55 | 6.34 |
| BRD3 | NM_007371 | 111.52 | 6.35 | 98 | 0.33 |
| ARHGEF16 | NM_014448 | 89.48 | 6.95 | 83.71 | 12.06 |
| FARP2 | NM_014808 | 84.16 | 4.7 | 78.98 | 6.95 |
| SLC25A17 | NM_006358 | 88.36 | 4.68 | 62.32 | 6.35 |
| HSPH1 | NM_006644 | 80.73 | 0.85 | 106.57 | 6.07 |
| KCNV1 | NM_014379 | 78.85 | 1.96 | 83.72 | 8.28 |
| GEMIN5 | NM_015465 | 95.59 | 7.8 | 109.82 | 17.69 |
| RYR1 | NM_000540 | 112.2 | 3.48 | 141.02 | 0.46 |
| UBE2Q2 | NM_173469 | 74.3 | 0.72 | 43.16 | 9.58 |
| EIF4A1 | NM_001416 | 66.31 | 1.68 | 78.99 | 3.19 |
| PTPN23 | NM_015466 | 85.78 | 4.01 | 92.69 | 11.27 |
| PLK1 | NM_005030 | 11.09 | 0.76 | 10.56 | 1.02 |
| AURKA | NM_003600 | 45.68 | 2.61 | 6.67 | 0.7 |
| MSH3 | NM_002439 | 108.93 | 0.37 | 81.94 | 5.09 |
| UBE2D1 | NM_003338 | 104.08 | 1.45 | 117.97 | 2.58 |
| AOAH | NM_001637 | 100.97 | 12.72 | 107.2 | 4.38 |
| UBE2D2 | NM_003339 | 96.77 | 3.99 | 71.95 | 0.87 |
| INPPL1 | NM_001567 | 104.61 | 9.19 | 93.36 | 5.47 |
| MYBL2 | NM_002466 | 85.89 | 2.63 | 49.03 | 2.15 |
| SVEP1 | NM_024500 | 93.28 | 5.62 | 98.16 | 1.07 |
| NR2E1 | NM_003269 | 104.16 | 5.49 | 120.14 | 1.26 |
| SLC25A26 | NM_173471 | 96.64 | 7.01 | 113.36 | 8.86 |
| PNLIP | NM_000936 | 110.07 | 0.82 | 135.24 | 1.61 |
| FBXO44 | NM_033182 | 96.05 | 8.84 | 117.04 | 1.28 |
| NGEF | NM_019850 | 95.47 | 6.01 | 115.98 | 17.34 |
| KIAA0999 | NM_025164 | 111.64 | 17.73 | 140.6 | 8.79 |
| ALPK2 | NM_052947 | 100.47 | 6.55 | 130.31 | 3.28 |
| SGEF | NM_015595 | 100.67 | 4.48 | 81.57 | 5.65 |
| POLRMT | NM_005035 | 92.53 | 2.28 | 91.9 | 10.93 |
| FBXO8 | NM_012180 | 123.36 | 10.96 | 132.02 | 6.68 |
| PPARA | NM_005036 | 107.18 | 5.19 | 122.18 | 2.21 |
| FKBP8 | NM_012181 | 85.73 | 0.32 | 68.5 | 1.61 |
| XRN2 | NM_012255 | 111.41 | 2.69 | 134 | 9.88 |
| CLCN4 | NM_001830 | 96.09 | 0.53 | 104.09 | 5.98 |
| KIAA0470 | NM_014812 | 113.13 | 9.86 | 91.63 | 0.45 |
| HECTD3 | NM_024602 | 90.35 | 1.04 | 117.08 | 2.27 |
| ST6GAL2 | NM_032528 | 108.23 | 5.01 | 128.99 | 17.13 |
| SLFN12 | NM_018042 | 102.5 | 9.27 | 109.6 | 11.28 |
| SLC25A32 | NM_030780 | 112.24 | 8.24 | 131.41 | 6.89 |
| VRK3 | NM_016440 | 107.88 | 6.29 | 124.43 | 8.29 |
| MCL1 | NM_021960 | 114.42 | 1.33 | 112.57 | 8.57 |
| AGGF1 | NM_018046 | 96.29 | 9.66 | 123.22 | 0.67 |
| UBE3C | NM_014671 | 93.16 | 6.2 | 117.35 | 14.27 |
| NUAK1 | NM_014840 | 104.74 | 10.33 | 121.04 | 0.86 |
| CSNK1A1L | NM_145203 | 49.05 | 2.34 | 57.68 | 2.29 |
| NUAK2 | NM_030952 | 85.1 | 1.49 | 89.1 | 8.28 |
| SENP8 | NM_145204 | 110.76 | 11.1 | 113.6 | 12.38 |
| SENP6 | NM_015571 | 67.9 | 5 | 80.04 | 12.25 |
| SPATA5 | NM_145207 | 117.48 | 7.66 | 102.43 | 9.8 |
| ROR1 | NM_005012 | 108.59 | 5.13 | 125.89 | 5.94 |
| PPID | NM_005038 | 119.39 | 2.38 | 118.53 | 3.17 |
| CNNM1 | NM_020348 | 106.65 | 11.02 | 96.22 | 0.7 |
| TRIP13 | NM_004237 | 99.02 | 0.72 | 99.18 | 4.49 |
| ACVRL1 | NM_000020 | 86.02 | 3.17 | 80.64 | 4.46 |
| FBXW8 | NM_153348 | 105.7 | 4.1 | 104.25 | 2.61 |
| DCAMKL2 | NM_152619 | 117.01 | 3.34 | 132.49 | 1.91 |
| TRIP12 | NM_004238 | 98.02 | 9.67 | 123.94 | 9.06 |
| PPP1CA | NM_002708 | 102.78 | 8.42 | 108.58 | 20.23 |
| MSH4 | NM_002440 | 113.25 | 15.01 | 113.99 | 1.48 |
| LMTK2 | NM_014916 | 100.17 | 7.9 | 80.84 | 3.08 |
| NR0B2 | NM_021969 | 105.39 | 14.52 | 105.17 | 20.13 |
| FBXL7 | NM_012304 | 105.19 | 5.92 | 101.27 | 0.87 |
| EXOSC5 | NM_020158 | 95.58 | 9.05 | 109.65 | 2.75 |
| LOC203427 | NM_145305 | 96.33 | 4.64 | 104.67 | 16.09 |
| SUV39H1 | NM_003173 | 61.49 | 7.35 | 69.07 | 6.63 |
| FAIM2 | NM_012306 | 94.84 | 2.11 | 110.76 | 0.81 |
| BCL2A1 | NM_004049 | 104.8 | 5.04 | 108.26 | 3.15 |
| FBXL4 | NM_012160 | 84.25 | 1.85 | 74.76 | 4.51 |
| TRPV3 | NM_145068 | 98.16 | 2.19 | 107.56 | 17.36 |
| ASCC3 | NM_006828 | 28.8 | 7.05 | 33.52 | 10.39 |
| STARD13 | NM_052851 | 83.59 | 7.05 | 115.99 | 3.37 |
| POLR2L | NM_021128 | 54.62 | 4.6 | 65.82 | 7.06 |
| TPP2 | NM_003291 | 109.5 | 9.04 | 124.01 | 11.42 |
| EIF4EBP1 | NM_004095 | 103.22 | 7.29 | 121.79 | 6.6 |
| ACVR1C | NM_145259 | 115.07 | 13.6 | 95.67 | 8.01 |
| CTSD | NM_001909 | 109.64 | 0.51 | 107.77 | 5 |
| NR2C1 | NM_003297 | 108.67 | 9.1 | 96.78 | 10.96 |
| NR2C2 | NM_003298 | 101.92 | 8.18 | 115.16 | 7.92 |
| SNIP1 | NM_024700 | 100.95 | 10.02 | 110.78 | 7.94 |
| MARK3 | NM_002376 | 99.34 | 6.7 | 107.32 | 2.24 |
| BCKDK | NM_005881 | 92.87 | 1.66 | 104.91 | 6.75 |
| KIAA0317 | NM_014821 | 118.49 | 10.27 | 69.3 | 1.19 |
| UBE2D3 | NM_003340 | 76.58 | 3.33 | 56.98 | 10.38 |
| DNASE1L1 | NM_006730 | 96.33 | 0.56 | 96.31 | 10.58 |
| PDK4 | NM_002612 | 108.34 | 5.21 | 109.27 | 11.99 |
| ERN2 | NM_033266 | 97.62 | 6.04 | 116.1 | 16.31 |
| CLCNKA | NM_004070 | 97.46 | 1.62 | 87.6 | 4.65 |
| STARD3 | NM_006804 | 98.84 | 0.77 | 100.51 | 1.43 |
| UBE2E1 | NM_003341 | 92.96 | 5.03 | 104.15 | 5.02 |
| CACNA1G | NM_018896 | 70.76 | 7.64 | 79.15 | 12.98 |
| UBE2G1 | NM_003342 | 86.54 | 5.73 | 108.76 | 2.91 |
| CLPX | NM_006660 | 104.93 | 6.89 | 113.85 | 17.11 |
| PDE1A | NM_005019 | 78.1 | 1.44 | 93.94 | 9.01 |
| FBXO39 | NM_153230 | 111.08 | 8.33 | 123.49 | 5.8 |
| PDPK1 | NM_002613 | 48.35 | 1.43 | 63.61 | 8.14 |
| ATAD3B | NM_031921 | 98.28 | 14.56 | 96.18 | 0.51 |
| TTRAP | NM_016614 | 98.61 | 14.83 | 103.32 | 5.13 |
| IKBKE | NM_014002 | 118.72 | 11.26 | 113.8 | 3.76 |
| SCN1A | NM_006920 | 67.23 | 1.27 | 48.91 | 6.31 |
| FAIM | NM_018147 | 74.78 | 0.9 | 101.05 | 15.64 |
| SCN3A | NM_006922 | 96.27 | 7.55 | 99.18 | 9.18 |
| SENP3 | NM_015670 | 107.02 | 9.99 | 131.47 | 9.95 |
| ADCK1 | NM_020421 | 92.15 | 6.95 | 120.67 | 8.71 |
| PDE10A | NM_006661 | 92.66 | 1.61 | 99.83 | 1.07 |
| UBE2G2 | NM_003343 | 86.84 | 7.94 | 96.13 | 10.21 |
| MLL | NM_005933 | 114.18 | 8.58 | 105.52 | 9.1 |
| FBXW2 | NM_012164 | 109.9 | 4.72 | 109.05 | 3.34 |
| SPHK1 | NM_021972 | 81.38 | 1.14 | 89.88 | 6.2 |
| UBE2H | NM_003344 | 108.83 | 6.99 | 95.43 | 3.88 |
| ULK2 | NM_014683 | 106.77 | 12.35 | 90.59 | 0.75 |
| CNNM4 | NM_020184 | 75.03 | 7.04 | 68.07 | 8.94 |
| POLR2F | NM_021974 | 41.58 | 1.5 | 24.78 | 0.68 |
| UBE2I | NM_003345 | 73.22 | 3.28 | 75.96 | 5.92 |
| NET1 | NM_005863 | 98.39 | 8.32 | 122.58 | 14.49 |
| FBXO2 | NM_012168 | 85.16 | 16.98 | 115.94 | 9.98 |
| MLLT4 | NM_005936 | 101.98 | 8.91 | 133.21 | 6.72 |
| UBE2N | NM_003348 | 106.72 | 9.66 | 120.13 | 11.19 |
| RAD18 | NM_020165 | 82.77 | 5.23 | 112.19 | 13.83 |
| APPL | NM_012096 | 93.97 | 10.67 | 119 | 8.22 |
| ST3GAL2 | NM_006927 | 107.04 | 13.44 | 115.97 | 4.81 |
| SPAST | NM_014946 | 91.69 | 7 | 97.5 | 8.38 |
| FBXW5 | NM_018998 | 91.59 | 0.59 | 84 | 1.02 |
| TRPM2 | NM_003307 | 100.85 | 3.35 | 98.64 | 11.04 |
| CARD8 | NM_014959 | 97.12 | 3.62 | 108.28 | 7.61 |
| ITPR2 | NM_002223 | 99.4 | 0.83 | 119.22 | 3.25 |
| UBE2F | NM_080678 | 111.56 | 6.01 | 123.5 | 1.71 |
| ASAH1 | NM_004315 | 83.74 | 1.92 | 85.96 | 1.66 |
| POLR3D | NM_001722 | 95.2 | 1.32 | 91.12 | 2.42 |
| PPP3CA | NM_000944 | 98.5 | 11.96 | 112.28 | 3.26 |
| HSPA2 | NM_021979 | 89.15 | 10.99 | 98.43 | 5.77 |
| UBE3B | NM_130466 | 106.67 | 7.45 | 110.93 | 3.42 |
| PDE3A | NM_000921 | 94.51 | 4.86 | 99.48 | 6.18 |
| EPHA6 | NM_173655 | 104.24 | 6.89 | 113.43 | 11.94 |
| GUCY1B2 | NM_004129 | 98.22 | 3.05 | 102.6 | 3.09 |
| JDP2 | NM_130469 | 104.14 | 6.78 | 85.66 | 2.32 |
| PDE3B | NM_000922 | 58.14 | 2.46 | 64.3 | 6.59 |
| PDE1C | NM_005020 | 93.68 | 2.37 | 105.67 | 2.02 |
| PDE4C | NM_000923 | 98.11 | 8.42 | 75.03 | 9.06 |
| PRKD2 | NM_016457 | 85.6 | 5.5 | 95.41 | 0.92 |
| PDE1B | NM_000924 | 83.72 | 0.66 | 75.94 | 0.53 |
| ST6GALNAC5 | NM_030965 | 82.03 | 0.88 | 120.05 | 1.67 |
| MALT1 | NM_006785 | 102.49 | 12.56 | 110.35 | 13.78 |
| SMURF1 | NM_020429 | 102.75 | 10.77 | 90.31 | 5.01 |
| SLC25A25 | NM_052901 | 108.5 | 4.61 | 112.35 | 8.2 |
| KIAA1639 | AB046859 | 88.85 | 3.41 | 56.08 | 1.52 |
| CD38 | NM_001775 | 87.22 | 2.24 | 104.82 | 3.53 |
| BCL2L11 | NM_138621 | 98.8 | 8.96 | 100.92 | 3.08 |
| DHX36 | NM_020865 | 80.49 | 4.4 | 89.63 | 3.24 |
| BRDT | NM_001726 | 98.66 | 2.7 | 111.79 | 3.14 |
| POLDIP2 | NM_015584 | 102.59 | 7.6 | 101.9 | 8.44 |
| GUCY1A2 | NM_000855 | 107.7 | 4.32 | 113.47 | 8.24 |
| UBE2L6 | NM_004223 | 88.6 | 8.56 | 109.85 | 9.87 |
| GUCY1A3 | NM_000856 | 97.45 | 3.9 | 108.53 | 8.01 |
| ARHGEF11 | NM_014784 | 95.26 | 8.59 | 94.39 | 1.37 |
| GUCY1B3 | NM_000857 | 18.07 | 4.39 | 2.82 | 0.51 |
| METAP2 | NM_006838 | 102.8 | 1.95 | 102.64 | 9.83 |
| TRPV5 | NM_019841 | 68.7 | 7.57 | 70.57 | 4.23 |
| LYK5 | NM_153335 | 90.72 | 0.58 | 107.77 | 16.91 |
| MCART1 | NM_033412 | 65.29 | 11.46 | 88.58 | 6.89 |
| UBE2V2 | NM_003350 | 67.05 | 0.65 | 82.84 | 9.87 |
| KCNQ5 | NM_019842 | 68.78 | 8.42 | 78.54 | 3.91 |
| ARHGEF17 | NM_014786 | 97.08 | 0.15 | 128.82 | 22.48 |
| VGCNL1 | NM_052867 | 107.15 | 13.16 | 135.9 | 16.81 |
| ORC1L | NM_004153 | 87.46 | 1.4 | 117.7 | 13.15 |
| CECR1 | NM_017424 | 112.23 | 5.56 | 124.51 | 8.94 |
| WNT8A | NM_031933 | 105.32 | 8.4 | 107.58 | 2.51 |
| FBXL10 | NM_032590 | 84.89 | 4.28 | 92.77 | 1.53 |
| EZH2 | NM_152998 | 93.53 | 3.57 | 85.11 | 11.57 |
| KSR | XM_290793 | 90.6 | 4.81 | 124.62 | 24.84 |
| RAD54B | NM_012415 | 76.2 | 6.89 | 106.91 | 0.73 |
| ACSL4 | NM_022977 | 114.25 | 7.35 | 149.62 | 21.92 |
| TANK | NM_004180 | 73.49 | 7.21 | 81.34 | 1.26 |
| BIRC8 | NM_033341 | 104.71 | 14.48 | 137.58 | 18.94 |
| FBXO25 | NM_012173 | 98.31 | 7.49 | 100.01 | 11.29 |
| MGC34725 | NM_173637 | 82.82 | 8.38 | 61.36 | 4.09 |
| FGD5 | NM_152536 | 98.05 | 5.38 | 100.73 | 6.24 |
| WNK1 | NM_018979 | 65.89 | 8.59 | 79.62 | 9.4 |
| UCP2 | NM_003355 | 109.68 | 10.53 | 118.27 | 2.93 |
| PSKH1 | NM_006742 | 47.76 | 1.46 | 52.81 | 8.11 |
| UGCG | NM_003358 | 115.24 | 6.28 | 123.76 | 3.71 |
| DHCR24 | NM_014762 | 86.3 | 8.73 | 75.78 | 3.94 |
| FBXO7 | NM_012179 | 71.65 | 4.26 | 85.76 | 7.01 |
| DHH | NM_021044 | 82.57 | 14.41 | 104.7 | 17.84 |
| PTAFR | NM_000952 | 80.52 | 5.68 | 91.4 | 6.39 |
| PDE4B | NM_002600 | 103.16 | 11.32 | 126.3 | 18.96 |
| DNTT | NM_004088 | 83.2 | 6.13 | 109.24 | 14.06 |
| TXNRD1 | NM_003330 | 88.81 | 4.17 | 84.55 | 9.34 |
| CLCN3 | NM_001829 | 98.5 | 5.01 | 134.9 | 9.91 |
| FBXO9 | NM_012347 | 104.8 | 6.21 | 130.17 | 8.39 |
| ARHGEF4 | NM_015320 | 63.37 | 6.2 | 68.62 | 11.14 |
| UHMK1 | NM_175866 | 88.14 | 5.09 | 94.41 | 9.11 |
| DDX43 | NM_018665 | 106.45 | 4.84 | 92.7 | 1.59 |
| PSMC4 | NM_006503 | 41.92 | 1.44 | 50.63 | 6.64 |
| PTPRE | NM_006504 | 103.66 | 11.32 | 133.25 | 4.8 |
| PTPRU | NM_005704 | 84.5 | 10.55 | 102.73 | 9.04 |
| UBR2 | NM_015255 | 63.85 | 5.17 | 77.23 | 0.21 |
| FBXL14 | NM_152441 | 106.5 | 10.39 | 122.27 | 3.98 |
| CBR1 | NM_001757 | 80.31 | 3.9 | 107.6 | 14.04 |
| HNF4G | NM_004133 | 126.77 | 16.04 | 147.1 | 17.83 |
| DISP2 | NM_033510 | 103.33 | 11.4 | 99.74 | 5.19 |
| YWHAH | NM_003405 | 109.66 | 10.15 | 125.04 | 9.19 |
| KCNK9 | NM_016601 | 101.66 | 12.21 | 110.52 | 19.52 |
| FBXO40 | NM_016298 | 112.18 | 12.69 | 102.57 | 17.4 |
| RBBP6 | NM_006910 | 63.71 | 0.66 | 82.67 | 2.85 |
| HSPA9B | NM_004134 | 51.23 | 7.44 | 61.44 | 5.56 |
| CXXC5 | NM_016463 | 68.32 | 2.8 | 64.39 | 1.7 |
| HSPA14 | NM_016299 | 78.29 | 2.54 | 91.67 | 12.74 |
| PCTP | NM_021213 | 102.99 | 19.86 | 124.57 | 10.91 |
| PDE8A | NM_002605 | 73.28 | 2.18 | 89.53 | 10.15 |
| RORB | NM_006914 | 91.13 | 12.74 | 109.05 | 3.97 |
| MAP3K9 | AF251442 | 93.43 | 5.71 | 111.3 | 1.37 |
| CDC42BPG | Y12337 | 70.38 | 3.83 | 77.19 | 1.24 |
| LIMK2 | NM_005569 | 98.58 | 0.27 | 108.81 | 10.41 |
| TRIM33 | NM_015906 | 94.13 | 11.06 | 95.2 | 9.18 |
| ADARB1 | NM_015833 | 99.19 | 8.41 | 95.41 | 4.05 |
| NOS2A | NM_000625 | 94.58 | 7.58 | 125.63 | 13.76 |
| DDX3X | NM_001356 | 78.33 | 2.19 | 105.87 | 18.11 |
| NVL | NM_002533 | 102.28 | 12.53 | 122.24 | 4.3 |
| XRCC5 | NM_021141 | 99.71 | 14.28 | 70.6 | 3.27 |
| UBE1L | NM_003335 | 80.59 | 8.08 | 84.55 | 3.23 |
| BRD2 | NM_005104 | 116.17 | 9.43 | 115.87 | 8.49 |
| PDE9A | NM_002606 | 111 | 3.94 | 120.74 | 6.72 |
| EDG5 | NM_004230 | 83.9 | 7.19 | 86.45 | 4.8 |
| UBE2A | NM_003336 | 105.05 | 2.57 | 72.26 | 10.83 |
| RXRG | NM_006917 | 100.22 | 8.87 | 116.28 | 9.59 |
| FBXL2 | NM_012157 | 100.5 | 10.99 | 128.48 | 11.23 |
| DDX24 | NM_020414 | 56.9 | 0.97 | 74.83 | 2.13 |
| UBE2B | NM_003337 | 103.95 | 6 | 123.17 | 4.09 |
| MELK | NM_014791 | 84.59 | 1.55 | 105.78 | 7.32 |
| FBXL3 | NM_012158 | 103.06 | 5.77 | 122.2 | 8.27 |
| POLR2K | NM_005034 | 48.47 | 3.62 | 37.46 | 1.93 |
| TRAIP | NM_005879 | 93.65 | 7.79 | 131.2 | 22.71 |
| DDX18 | NM_006773 | 44.24 | 2.49 | 59.86 | 1.5 |
| ULK3 | AL117482 | 112.03 | 12.74 | 120.3 | 9.44 |
| CLCN6 | NM_001286 | 113.12 | 5.41 | 119.18 | 28.91 |
| PSMB9 | NM_002800 | 89.61 | 9.86 | 109.63 | 12.23 |
| UHMK1 | NM_144624 | 104.81 | 11.5 | 102.62 | 8.92 |
| CDC42BPA | NM_003607 | 94.15 | 8.66 | 80.46 | 10.24 |
| HSPA5BP1 | NM_017870 | 100.77 | 6.88 | 124.19 | 12.54 |
| PTPN6 | NM_080549 | 95.32 | 5.34 | 96.13 | 4.04 |
| DCAMKL3 | AB051552 | 117.44 | 17.24 | 143.34 | 28.22 |
| STARD10 | NM_006645 | 74.12 | 1.57 | 78.86 | 10.5 |
| TRPC1 | NM_003304 | 114.61 | 0.15 | 118.23 | 8.86 |
| CXXC1 | NM_014593 | 76.04 | 9.86 | 85.36 | 3.13 |
| TRPC3 | NM_003305 | 102.54 | 8.8 | 97.22 | 3.94 |
| SLFN11 | NM_152270 | 44.29 | 6.9 | 48.29 | 10.1 |
| LOC91137 | NM_138773 | 73.77 | 8.9 | 75.78 | 5.25 |
| TFRC | NM_003234 | 85.69 | 12.17 | 93.38 | 3.2 |
| ADCY3 | NM_004036 | 97.35 | 8.13 | 114.4 | 11.45 |
| KCNA2 | NM_004974 | 76.81 | 1.58 | 77.95 | 3.92 |
| AMPD2 | NM_004037 | 84.76 | 2.86 | 93.95 | 1.81 |
| KCNB1 | NM_004975 | 91.49 | 7.02 | 99.37 | 15.3 |
| KCNC1 | NM_004976 | 94.93 | 5.24 | 90.86 | 0.6 |
| KCNC3 | NM_004977 | 80.29 | 6.65 | 60.47 | 11.13 |
| SLC25A6 | NM_001636 | 87.71 | 0.27 | 96.48 | 14.39 |
| TSSK3 | NM_052841 | 113.89 | 13.09 | 133.71 | 9.4 |
| KCND1 | NM_004979 | 103 | 2.25 | 96.72 | 3.38 |
| COL4A3BP | NM_005713 | 130.33 | 1.7 | 135.92 | 4.69 |
| CDH26 | NM_177980 | 100.81 | 12.13 | 100.59 | 11.46 |
| CIT | NM_007174 | 61.34 | 2.64 | 64.87 | 2 |
| TRPM3 | NM_020952 | 101.93 | 12.13 | 118.69 | 1.9 |
| KCNS2 | AB032970 | 61.64 | 2.58 | 67.33 | 1.51 |
| JUP | NM_002230 | 105.95 | 0.72 | 119.85 | 2.39 |
| FBXO21 | NM_033624 | 100.86 | 13.78 | 112.35 | 2.57 |
| ST3GAL1 | NM_003033 | 94.23 | 3.59 | 91.89 | 6.52 |
| BCL2L1 | NM_138578 | 110.76 | 12.59 | 83.07 | 5.92 |
| KCNG1 | NM_002237 | 103.52 | 6.81 | 115.06 | 4.72 |
| KCNQ2 | NM_172107 | 84.39 | 0.95 | 54.63 | 7.48 |
| EPHB2 | NM_017449 | 69.69 | 2.14 | 67.13 | 5.58 |
| MBD1 | NM_015846 | 98.65 | 7.97 | 111.42 | 3.64 |
| DNMT2 | NM_004412 | 112.71 | 8.5 | 137.26 | 7.21 |
| EXOSC8 | NM_181503 | 80.21 | 3.13 | 83.96 | 5.51 |
| EDG2 | NM_057159 | 106.7 | 9.81 | 105.29 | 11.82 |
| IKBKB | AF080158 | 102.15 | 14.7 | 95.02 | 8.56 |
| DKFZp761P0423 | AL833872 | 94.13 | 8.16 | 88.11 | 7.04 |
| SLC30A4 | NM_013309 | 72.73 | 2.19 | 94.51 | 5.33 |
| KCNH8 | NM_144633 | 80.23 | 3.2 | 79.61 | 2.49 |
| LPL | NM_000237 | 101.3 | 2.19 | 94.09 | 16.27 |
| GLI3 | NM_000168 | 94.64 | 2.55 | 65.04 | 5.53 |
| UBE2E2 | NM_152653 | 101.71 | 5.86 | 116.99 | 18.49 |
| TRPC7 | NM_020389 | 83.81 | 4.94 | 109.61 | 0.6 |
| BTK | NM_000061 | 88.55 | 5.86 | 80.07 | 1.26 |
| FANCA | NM_000135 | 90.19 | 11.02 | 95 | 0.6 |
| FANCC | NM_000136 | 73.57 | 6.86 | 49.58 | 1.37 |
| CACNA1S | NM_000069 | 76.82 | 6.22 | 79.4 | 9.43 |
| SLC30A8 | NM_173851 | 61.99 | 12.94 | 77.8 | 9.01 |
| SUV39H2 | NM_024670 | 78.91 | 0.31 | 106.84 | 15.83 |
| STYK1 | NM_018423 | 42.18 | 1.85 | 44.87 | 3.39 |
| PRKX | NM_005044 | 90.72 | 1.51 | 108.99 | 0.8 |
| AMID | NM_032797 | 110.93 | 0.41 | 120.15 | 9.38 |
| SKIV2L | NM_006929 | 70.61 | 0.5 | 78.38 | 2.92 |
| CHTF18 | NM_022092 | 81.71 | 0.42 | 72.32 | 11.28 |
| CDC14C | NM_152627 | 103.04 | 2.94 | 119.97 | 11.72 |
| ESRRA | NM_004451 | 64.26 | 3.23 | 81.22 | 3.78 |
| PAFAH1B2 | NM_002572 | 98.43 | 6.15 | 102.11 | 4.31 |
| ESRRB | NM_004452 | 77.57 | 0.33 | 85.08 | 6.57 |
| PAFAH1B3 | NM_002573 | 104.61 | 7.98 | 132.56 | 14.35 |
| TRPC5 | NM_012471 | 92.11 | 6.67 | 99.64 | 5.33 |
| UBE2U | NM_152489 | 82.51 | 6.72 | 22.23 | 3.91 |
| IFIH1 | NM_022168 | 74.83 | 10.9 | 84.85 | 5.51 |
| AMPD1 | NM_000036 | 103.41 | 6.56 | 101.59 | 2.03 |
| GAK | NM_005255 | 64.66 | 2.42 | 52.58 | 7.39 |
| UBE1DC1 | NM_024818 | 83.33 | 2.17 | 116.98 | 8.73 |
| GSG2 | NM_031965 | 49.08 | 5.11 | 70.2 | 3.91 |
| ATAD3A | NM_018188 | 86.26 | 3.31 | 90.93 | 12.3 |
| TAX1BP1 | NM_006024 | 86.68 | 0.53 | 70.98 | 10.27 |
| DNASE1 | NM_005223 | 94.07 | 8.34 | 99.29 | 1.31 |
| PPM1D | NM_003620 | 85.23 | 4.75 | 71.72 | 1.21 |
| EDG3 | NM_005226 | 77.62 | 5.61 | 97.99 | 2.09 |
| VPS4A | NM_013245 | 95.02 | 9.14 | 123.9 | 4.85 |
| ENTPD1 | NM_001776 | 95.54 | 4.39 | 122.23 | 17.32 |
| CACNA1F | NM_005183 | 49.41 | 2.2 | 52.03 | 2.7 |
| PAK3 | NM_002578 | 103.99 | 0.9 | 120.75 | 8.01 |
| PDIK1L | NM_152835 | 68.44 | 3.28 | 69.46 | 1.88 |
| 2'-PDE | NM_177966 | 87.2 | 6.67 | 109.84 | 9.79 |
| PPT1 | NM_000310 | 54.49 | 7.76 | 50.4 | 3.53 |
| CHFR | NM_018223 | 74.18 | 0.25 | 92.26 | 2.01 |
| MLLT3 | NM_004529 | 95.47 | 3.13 | 118.96 | 7.46 |
| UBE1L2 | NM_018227 | 96.9 | 5.01 | 140.04 | 12.67 |
| ADCY7 | NM_001114 | 86.36 | 2.33 | 103.83 | 5.66 |
| FBXO18 | NM_032807 | 97.7 | 2.83 | 118.77 | 11.55 |
| CSNK1G3 | NM_004384 | 68.21 | 2.09 | 55.4 | 7.87 |
| SLC25A36 | NM_018155 | 104.03 | 4.61 | 110.56 | 3.82 |
| CAMK1 | NM_003656 | 77.15 | 7.24 | 73.33 | 7.26 |
| POLE4 | NM_019896 | 100.86 | 4.22 | 118.58 | 5.89 |
| ADCY8 | NM_001115 | 84.25 | 10.73 | 97.4 | 8.41 |
| UBE2O | NM_022066 | 84.39 | 3.51 | 97.63 | 4.3 |
| PPM1M | NM_144641 | 104.05 | 2.89 | 69.52 | 1.75 |
| PTK9 | NM_002822 | 73.33 | 0.45 | 95.92 | 0.58 |
| SLC30A1 | NM_021194 | 93.99 | 7.51 | 107.55 | 7.87 |
| CNOT6L | NM_144571 | 68.69 | 6.11 | 88.64 | 0.71 |
| RAB3D | NM_004283 | 105.38 | 8.86 | 127.34 | 12.35 |
| BCL2L10 | NM_020396 | 66.85 | 6.18 | 78.33 | 4.33 |
| PTPN1 | NM_002827 | 75.3 | 8.94 | 93.13 | 7.23 |
| SLC4A1AP | NM_018158 | 84.6 | 0.42 | 85.33 | 1.07 |
| ADCY9 | NM_001116 | 77.95 | 14.21 | 59.12 | 4.16 |
| FIGN | NM_018086 | 92.96 | 3.07 | 91.4 | 7.39 |
| HIPK1 | NM_152696 | 84.79 | 13.94 | 70.06 | 3.56 |
| ASCC3L1 | NM_014014 | 38.27 | 5.33 | 45.73 | 1.64 |
| ERCC1 | NM_001983 | 71.89 | 6.39 | 92.04 | 10.37 |
| MGC26597 | NM_152700 | 91.43 | 0.62 | 108.49 | 9.89 |
| CATSPER1 | NM_053054 | 73.82 | 0.09 | 90.65 | 1.77 |
| NR1I3 | NM_005122 | 95.66 | 4.38 | 133.61 | 10.23 |
| SENP5 | NM_152699 | 101.89 | 5.9 | 91.44 | 3.63 |
| FLJ25006 | NM_144610 | 80.97 | 1.44 | 88.17 | 0.94 |
| FBXL12 | NM_017703 | 75.16 | 1.35 | 83.3 | 8.98 |
| NR1H4 | NM_005123 | 84.26 | 0.52 | 86.33 | 7.15 |
| FLJ20035 | NM_017631 | 7.36 | 2.19 | 2.74 | 1.63 |
| MSH6 | NM_000179 | 88.33 | 3.51 | 98.29 | 4.31 |
| PIP5K1A | NM_003557 | 93.18 | 0.75 | 135.59 | 26.79 |
| PTPN3 | NM_002829 | 80.51 | 0.57 | 96.56 | 3.95 |
| EXDL1 | NM_152596 | 80.92 | 1.15 | 86.56 | 10.75 |
| CBS | NM_000071 | 62.42 | 6.4 | 73.78 | 1.01 |
| KCNA1 | NM_000217 | 102.62 | 9.37 | 111.9 | 1.61 |
| KCNQ1 | NM_000218 | 71.76 | 3.73 | 72.73 | 0.07 |
| POLA2 | NM_002689 | 88.63 | 0.43 | 94.88 | 7.99 |
| DDX19A | NM_018332 | 68.75 | 2.36 | 51.7 | 23.81 |
| RAD23A | NM_005053 | 98.6 | 5.29 | 88.02 | 3.35 |
| FBXO6 | NM_018438 | 78.88 | 0.03 | 94.71 | 8.74 |
| NR1D2 | NM_005126 | 97.44 | 4.93 | 102.55 | 2.99 |
| TRPM4 | NM_017636 | 83.34 | 5.8 | 112.43 | 6.56 |
| MGC42105 | NM_153361 | 74.49 | 1.18 | 95.06 | 8.71 |
| PDE4A | NM_006202 | 58.51 | 1.41 | 72.57 | 5.73 |
| FBXW4 | NM_022039 | 20.2 | 1.62 | 23.51 | 1.75 |
| PDE4D | NM_006203 | 84.52 | 7.54 | 103.93 | 7.24 |
| JARID1A | NM_005056 | 106.15 | 10.65 | 140.31 | 3.45 |
| PTPN14 | NM_005401 | 72.17 | 5.39 | 74.8 | 12.92 |
| RBBP5 | NM_005057 | 80.62 | 3.94 | 98.34 | 6.82 |
| FBXL20 | NM_032875 | 101.89 | 0.43 | 95.89 | 9.25 |
| CAMK1G | NM_020439 | 71.75 | 2.09 | 101.18 | 0.71 |
| PDE6C | NM_006204 | 85.46 | 1.25 | 79 | 1.29 |
| BCS1L | NM_004328 | 56.24 | 9.91 | 78.61 | 6.38 |
| CNGB3 | NM_019098 | 72.16 | 0.2 | 56.27 | 10.88 |
| ST3GAL6 | NM_006100 | 86.34 | 3.17 | 114.78 | 21.17 |
| LIPG | NM_006033 | 87.09 | 1.2 | 87.32 | 8.43 |
| EPHA3 | NM_005233 | 91.54 | 0.82 | 87.98 | 4.72 |
| C14orf114 | NM_018199 | 99.55 | 2.84 | 112.85 | 16.41 |
| NR2F6 | NM_005234 | 81.64 | 3.44 | 87.19 | 9.48 |
| GRK5 | NM_005308 | 65.74 | 5.27 | 86.6 | 0.18 |
| TNFRSF13B | NM_012452 | 63.37 | 3.62 | 70.96 | 12.39 |
| HUS1 | NM_004507 | 76.74 | 3.9 | 101.5 | 8.97 |
| AFG3L2 | NM_006796 | 71.91 | 14.24 | 99.77 | 2.4 |
| C11orf11 | NM_006133 | 64.74 | 5.9 | 69.59 | 4.76 |
| CACNA1I | NM_021096 | 56.57 | 5.4 | 45.83 | 8.95 |
| MCOLN3 | NM_018298 | 97.39 | 11.92 | 93.69 | 1.65 |
| CACNA1H | NM_021098 | 70.5 | 6.96 | 82.9 | 2.72 |
| UBE2W | NM_018299 | 64.58 | 6.81 | 89.24 | 0.31 |
| PAWR | NM_002583 | 87.8 | 11.31 | 40.22 | 3.42 |
| ENPP2 | NM_006209 | 64.98 | 2.52 | 101.13 | 4.96 |
| SLC30A3 | NM_003459 | 77.62 | 2.27 | 92.46 | 17.5 |
| HCFC1 | NM_005334 | 93.4 | 15.76 | 87.65 | 25.31 |
| ATAD1 | NM_032810 | 95.72 | 1.36 | 114.37 | 1.02 |
| GRK7 | NM_139209 | 64.76 | 1.06 | 76.16 | 0.59 |
| PRKAG3 | NM_017431 | 59.77 | 0.55 | 58.87 | 4.16 |
| CRADD | NM_003805 | 51.75 | 0.61 | 31.66 | 32.11 |
| PLA1A | NM_015900 | 104.25 | 5.35 | 122.01 | 20.75 |
| EXOSC6 | NM_058219 | 94.46 | 1.23 | 80.57 | 2.21 |
| PTPN4 | NM_002830 | 80.23 | 2.52 | 96.32 | 9.25 |
| ENDOG | NM_004435 | 89.52 | 5.73 | 88.64 | 6.47 |
| PLA2G6 | NM_003560 | 74.48 | 1.58 | 68.12 | 0 |
| ADCY2 | NM_020546 | 70.35 | 14.38 | 72.85 | 22.97 |
| TIAM2 | NM_012454 | 86.33 | 9.52 | 67.67 | 0.48 |
| AMHR2 | NM_020547 | 61.33 | 0.39 | 86.41 | 0.17 |
| PTPN9 | NM_002833 | 79.99 | 9.14 | 114.87 | 4.69 |
| CLCN2 | NM_004366 | 52.29 | 2.15 | 28.82 | 3.2 |
| EXOSC4 | NM_019037 | 62.52 | 4.05 | 76.56 | 7.32 |
| FGD1 | NM_004463 | 106.96 | 3.8 | 141.46 | 6.9 |
| EDD1 | NM_015902 | 85.71 | 2.88 | 77.88 | 11.9 |
| POLM | NM_013284 | 66.51 | 0.44 | 81.37 | 4.45 |
| CLSPN | NM_022111 | 87.25 | 4.27 | 89.31 | 1.91 |
| BIRC1 | NM_004536 | 73.67 | 5.2 | 96.19 | 2.35 |
| MULK | NM_018238 | 80.11 | 3.39 | 121.07 | 17.11 |
| HIP2 | NM_005339 | 107.41 | 1.31 | 111.67 | 10.42 |
| C9orf100 | NM_032818 | 51 | 4.19 | 63.01 | 0.13 |
| PTCH2 | NM_003738 | 78.67 | 5.11 | 109.81 | 15.38 |
| BFAR | NM_016561 | 80.5 | 5.4 | 62.06 | 5.16 |
| SSTR3 | NM_001051 | 52.66 | 2.67 | 55.85 | 3.8 |
| FIGNL1 | NM_022116 | 98.09 | 7.55 | 107.08 | 7.96 |
| GLI1 | NM_005269 | 101 | 2.41 | 97.85 | 17.07 |
| SLC25A2 | NM_031947 | 64.38 | 4.06 | 48.57 | 4.58 |
| PTPN12 | NM_002835 | 64.93 | 1.01 | 60.48 | 1.27 |
| POLB | NM_002690 | 71.67 | 1.28 | 100.68 | 1.2 |
| PTPRB | NM_002837 | 72.71 | 4.76 | 81.91 | 0.79 |
| POLE2 | NM_002692 | 102.29 | 10.56 | 117.78 | 9.08 |
| POLG | NM_002693 | 78.93 | 9.7 | 73.51 | 17.65 |
| TRRAP | NM_003496 | 81.93 | 7.91 | 81.12 | 6.15 |
| POLR2E | NM_002695 | 45.71 | 4.31 | 46.03 | 5.82 |
| EIF4A2 | NM_001967 | 77.41 | 6.27 | 79.6 | 6.6 |
| STK36 | NM_015690 | 92.34 | 7.21 | 39.6 | 2.53 |
| DFFB | NM_004402 | 82.58 | 3.03 | 117.92 | 15.72 |
| THOC1 | NM_005131 | 79.84 | 7.18 | 106.67 | 14.52 |
| BNIP2 | NM_004330 | 86.15 | 1.09 | 114.19 | 1.51 |
| RORC | NM_005060 | 91.09 | 3.29 | 109.92 | 2.56 |
| CSNK1G1 | NM_022048 | 85.68 | 5.39 | 84.07 | 0.09 |
| POLD4 | NM_021173 | 73.31 | 3.99 | 89.55 | 13.51 |
| ERCC8 | NM_000082 | 105.94 | 8.48 | 104.77 | 12.66 |
| POLR2G | NM_002696 | 40.65 | 3.76 | 51.15 | 6.69 |
| CLCN1 | NM_000083 | 88.7 | 12.19 | 106.76 | 11.2 |
| CATSPER3 | NM_178019 | 91.61 | 3.13 | 93.09 | 0.81 |
| CLCN5 | NM_000084 | 92.39 | 10.56 | 89.64 | 0.88 |
| CLCNKB | NM_000085 | 101.58 | 11.81 | 129.55 | 10.2 |
| ST6GALNAC1 | NM_018414 | 86.68 | 7.89 | 121.49 | 12.95 |
| PSMC1 | NM_002802 | 56.72 | 0.02 | 52.53 | 6.5 |
| PSMC2 | NM_002803 | 49.31 | 3.76 | 55.73 | 9.38 |
| PSMC3 | NM_002804 | 54.48 | 2.56 | 59.49 | 3.27 |
| KCND2 | NM_012281 | 89.86 | 1.76 | 81.04 | 3.04 |
| LIPF | NM_004190 | 90.17 | 0.9 | 121.35 | 0.22 |
| PRKACG | NM_002732 | 113.23 | 10.49 | 115.76 | 17.37 |
| PSMC5 | NM_002805 | 36.24 | 1.95 | 44.72 | 9.91 |
| LOC153328 | NM_145282 | 103.38 | 11.69 | 105.42 | 8.82 |
| CNGA1 | NM_000087 | 85.39 | 9.28 | 85.86 | 2.68 |
| RIOK2 | NM_018343 | 79.99 | 8.17 | 86.28 | 18.23 |
| EXOSC7 | NM_015004 | 74.83 | 9.31 | 72.08 | 2.89 |
| FOXK2 | NM_004514 | 83.39 | 4.2 | 123.24 | 2.47 |
| SCN11A | NM_014139 | 91.29 | 6.15 | 114.09 | 7.19 |
| DDX25 | NM_013264 | 76.86 | 10.65 | 117.65 | 15.26 |
| ST8SIA3 | NM_015879 | 84.8 | 2.86 | 109.31 | 12.73 |
| RFC2 | NM_002914 | 112.29 | 4.13 | 106.99 | 5.74 |
| KCNG2 | NM_012283 | 48.69 | 27.17 | 89.24 | 15.04 |
| PSMC6 | NM_002806 | 50.15 | 6.88 | 61.01 | 16.59 |
| PRKAG1 | NM_002733 | 51.87 | 1.49 | 64.86 | 1.92 |
| PTP4A1 | NM_003463 | 54.28 | 2.75 | 40.67 | 8.5 |
| KCNH4 | NM_012285 | 93.03 | 7.57 | 96.42 | 7.2 |
| MLKL | NM_152649 | 97.06 | 5.76 | 89.87 | 7.35 |
| ERCC5 | NM_000123 | 78.81 | 2.58 | 89.35 | 4.68 |
| COX5B | NM_001862 | 90.31 | 3.17 | 102 | 5.23 |
| HIPK4 | NM_144685 | 88.85 | 12.6 | 94.92 | 8.54 |
| PTPRG | NM_002841 | 82.57 | 0.08 | 77.65 | 14.32 |
| PTPRH | NM_002842 | 89.35 | 6.26 | 70.6 | 0.67 |
| KCNQ3 | NM_004519 | 77.07 | 10.21 | 66.7 | 2.76 |
| PTPRJ | NM_002843 | 106.3 | 12.46 | 100.79 | 2.01 |
| ACVR1 | NM_001105 | 89.41 | 4.45 | 93.3 | 9.57 |
| PTPRK | NM_002844 | 56.29 | 5.13 | 60.99 | 3.49 |
| PDE8B | NM_003719 | 101.73 | 4.27 | 101.55 | 9 |
| PTPRM | NM_002845 | 104.74 | 10.94 | 106.48 | 11.09 |
| TSSK2 | NM_053006 | 87.5 | 3.11 | 111.46 | 14.41 |
| DDX5 | NM_004396 | 91.8 | 4.02 | 117.56 | 7.54 |
| CDH2 | NM_001792 | 88.89 | 12.49 | 123.22 | 9.82 |
| EZH1 | NM_001991 | 98.81 | 4.82 | 127.91 | 15.37 |
| PCTK2 | NM_002595 | 91.19 | 6.06 | 99.86 | 13.76 |
| DDX10 | NM_004398 | 46.73 | 2.54 | 51.6 | 2.47 |
| CDH5 | NM_001795 | 24.66 | 1.32 | 26.19 | 3.84 |
| FBXO38 | NM_024862 | 104.39 | 6.28 | 101.32 | 4.49 |
| PDE2A | NM_002599 | 59.85 | 3.12 | 84.03 | 8.93 |
| MKNK2 | NM_017572 | 103.56 | 8.09 | 83.4 | 6.36 |
| TP53RK | NM_033550 | 80.89 | 0.36 | 99.46 | 7.16 |
| WWP1 | NM_007013 | 67.75 | 6.38 | 78.46 | 0.07 |
| POLE3 | NM_017443 | 113.95 | 9.21 | 154.77 | 8.77 |
| PES1 | NM_014303 | 70.25 | 5.18 | 72.64 | 1.49 |
| PLEKHG6 | NM_018173 | 76.23 | 10.48 | 85.29 | 2.29 |
| WWP2 | NM_007014 | 129.27 | 7.72 | 128.38 | 22.8 |
| PTPRN | NM_002846 | 21.39 | 0.23 | 15.7 | 1.38 |
| STK24 | NM_003576 | 82.72 | 2.12 | 77.53 | 7.95 |
| RYR2 | NM_001035 | 87.91 | 6.34 | 97.59 | 5.73 |
| RYR3 | NM_001036 | 88.75 | 4.23 | 105.5 | 0.18 |
| LIPA | NM_000235 | 74.95 | 10.74 | 103.51 | 10.19 |
| RAD54L | NM_003579 | 71.49 | 2.74 | 104.32 | 0.19 |
| LIPC | NM_000236 | 64.1 | 5.7 | 58.74 | 10.19 |
| ST8SIA2 | NM_006011 | 55.83 | 0.78 | 66.78 | 1.06 |
| PHKG1 | NM_006213 | 22.2 | 4.69 | 27.46 | 4.07 |
| EEF2K | NM_013302 | 105.4 | 6.1 | 118.44 | 4.14 |
| CNNM2 | NM_017649 | 98.25 | 5.09 | 88.85 | 9.12 |
| ST8SIA5 | NM_013305 | 103.74 | 6.96 | 141.66 | 7.63 |
| FBXL8 | NM_018378 | 117.71 | 2.34 | 104.34 | 5.96 |
| KCNK13 | NM_022054 | 99.22 | 4.18 | 137.65 | 0.36 |
| POLR1D | NM_015972 | 55.44 | 6.46 | 46.94 | 4.27 |
| STK39 | NM_013233 | 96.14 | 8.22 | 98.46 | 1.67 |
| TGM2 | NM_004613 | 64.32 | 31.37 | 82.69 | 5.05 |
| SETDB1 | NM_012432 | 65.12 | 6.3 | 54.22 | 5.2 |
| SRMS | NM_080823 | 55.62 | 3.59 | 58.85 | 2.09 |
| KCNA7 | NM_031886 | 98.02 | 0.31 | 47.71 | 3.59 |
| PKDREJ | NM_006071 | 97.09 | 2.55 | 122.95 | 0.92 |
| AIFL | NM_144704 | 96.16 | 3.88 | 66.55 | 3.29 |
| UBE2C | NM_007019 | 47.78 | 9.33 | 61.47 | 13.71 |
| MAST2 | NM_015112 | 122 | 5.06 | 124.97 | 4.47 |
| CLK3 | NM_003992 | 117.06 | 0.88 | 136.37 | 5.23 |
| MAST4 | AB002301 | 82.16 | 0.1 | 90.78 | 7.84 |
| CCNE2 | NM_057749 | 112.24 | 3.33 | 88.76 | 1.06 |
| FGFR4 | NM_002011 | 74.3 | 3.78 | 78.86 | 1.9 |
| KDR | NM_002253 | 97.4 | 3.35 | 94.69 | 10.74 |
| JUND | NM_005354 | 69.58 | 3.4 | 78.74 | 17.45 |
| CLK2 | NM_003993 | 75.6 | 0.2 | 71.71 | 12.55 |
| BUB1B | NM_001211 | 89.37 | 0.17 | 74.47 | 14.37 |
| KIF3C | NM_002254 | 59.19 | 2.36 | 47.86 | 18.2 |
| LCK | NM_005356 | 72.62 | 3.82 | 83.65 | 3.02 |
| NPR2 | NM_003995 | 72.06 | 1.27 | 63.98 | 3.84 |
| RPN2 | NM_002951 | 89.94 | 0.39 | 83.72 | 0.38 |
| KIF3B | NM_004798 | 88.63 | 3.09 | 83.85 | 2.84 |
| TACC3 | NM_006342 | 15.85 | 4.06 | 13.62 | 0.73 |
| KIF15 | NM_020242 | 73.81 | 0.07 | 17.74 | 9.43 |
| INPP5D | NM_005541 | 85.69 | 9.71 | 74.77 | 6.55 |
| CCNG1 | NM_004060 | 119.3 | 4.53 | 110.39 | 3.05 |
| MERTK | NM_006343 | 94.79 | 10.36 | 95.26 | 5.47 |
| YWHAB | NM_003404 | 88.88 | 4.27 | 90.77 | 3.85 |
| IRS1 | NM_005544 | 59.2 | 0.27 | 67.81 | 11.61 |
| CDKN1B | NM_004064 | 78.43 | 0.35 | 90.18 | 0.38 |
| WASL | NM_003941 | 66.3 | 4.49 | 58.56 | 1.73 |
| CTNNBIP1 | NM_020248 | 69.03 | 9.11 | 87.2 | 15.52 |
| ITK | NM_005546 | 98.48 | 0.41 | 110.1 | 7.56 |
| PDGFB | NM_002608 | 99.34 | 4.4 | 100.13 | 5.37 |
| ITGA5 | NM_002205 | 88.49 | 2.29 | 81.89 | 2.36 |
| PDGFRB | NM_002609 | 80.83 | 2.66 | 80.01 | 1.17 |
| ALS2CR7 | NM_139158 | 100.23 | 2.09 | 77.14 | 4.64 |
| NFKB1 | NM_003998 | 100.72 | 8.09 | 77.59 | 11.34 |
| FOXO1A | NM_002015 | 107.66 | 1.25 | 65.47 | 6.56 |
| C20orf24 | NM_018840 | 111.88 | 8.19 | 97.22 | 8.37 |
| SMAD4 | NM_005359 | 115.96 | 0.53 | 75.5 | 20.24 |
| MGC11266 | NM_024322 | 77.07 | 2.45 | 73.94 | 6.57 |
| RPS6KA1 | NM_002953 | 127.77 | 5.77 | 134.73 | 5.88 |
| MYL9 | NM_181526 | 108.54 | 14 | 95.18 | 0.36 |
| NOTCH4 | NM_004557 | 66.53 | 2.85 | 53.01 | 6.49 |
| OGFR | NM_007346 | 55.16 | 3.26 | 59.85 | 5.93 |
| TUBG1 | NM_001070 | 39.42 | 0.07 | 26.1 | 0.17 |
| DHX8 | NM_004941 | 69.58 | 0.54 | 59.19 | 13.2 |
| RREB1 | NM_002955 | 113.2 | 5.62 | 90.53 | 1.69 |
| MAP3K3 | NM_002401 | 98.33 | 1.2 | 95.8 | 0.4 |
| FLT1 | NM_002019 | 108.24 | 3.67 | 106.75 | 0.73 |
| CAMKK2 | NM_006549 | 93.74 | 1.03 | 101.86 | 0.17 |
| CDK8 | NM_001260 | 99.67 | 7.63 | 108.62 | 2.36 |
| CENPA | NM_001809 | 83.91 | 6.6 | 75.81 | 1.35 |
| FGFR1 | NM_000604 | 87.37 | 3.32 | 80.51 | 9.34 |
| TAOK1 | AB037782 | 103.2 | 1.84 | 113.62 | 2.27 |
| CDKL2 | NM_003948 | 67.64 | 7.03 | 66.5 | 4.96 |
| RHOA | NM_001664 | 114.03 | 5.32 | 127.94 | 7.23 |
| CDK9 | NM_001261 | 99.98 | 1.25 | 87.89 | 6.55 |
| MYC | NM_002467 | 83.33 | 3.06 | 86.73 | 3.69 |
| STK40 | AK024504 | 43.82 | 2.08 | 46.82 | 4.96 |
| AAK1 | NM_014911 | 76.44 | 0.57 | 61.04 | 0.73 |
| MARK1 | NM_018650 | 104.67 | 3.67 | 110.54 | 2.11 |
| TRIB1 | NM_025195 | 81.42 | 10.17 | 94.74 | 5.68 |
| LMTK3 | AB067470 | 86.5 | 6.92 | 103.48 | 6.16 |
| KIF2B | NM_032559 | 108 | 3.92 | 90.89 | 16.51 |
| UCKL1 | NM_017859 | 83.69 | 3.56 | 98.52 | 6 |
| KIFC1 | BC000712 | 83.39 | 4.81 | 104.52 | 4.83 |
| TYMS | NM_001071 | 88.33 | 1.08 | 91.92 | 7.77 |
| SLPI | NM_003064 | 106.62 | 4.98 | 119.48 | 3.04 |
| RYK | NM_002958 | 78.16 | 2.19 | 79.5 | 2.54 |
| FZD2 | NM_001466 | 89.14 | 4.74 | 47.03 | 7.75 |
| NPC1 | NM_000271 | 84.01 | 5.14 | 91.77 | 2.9 |
| C20orf4 | NM_015511 | 87.69 | 3.64 | 89.84 | 0.67 |
| VCAM1 | NM_001078 | 110.7 | 0.03 | 96.56 | 4.49 |
| DHX35 | NM_021931 | 89.15 | 7.3 | 70.9 | 5.86 |
| ORC6L | NM_014321 | 99.2 | 9.37 | 94.16 | 8.96 |
| LATS2 | NM_014572 | 103.09 | 4.11 | 49.51 | 9.09 |
| KIF9 | NM_022342 | 127.39 | 4.28 | 127.21 | 6.55 |
| TEC | NM_003215 | 91.06 | 5.04 | 87.5 | 9.76 |
| CCNB2 | NM_004701 | 103.15 | 7.19 | 112.92 | 4.38 |
| TERT | NM_003219 | 103.25 | 2.2 | 93.42 | 1.73 |
| KIFC3 | NM_005550 | 85.36 | 6.85 | 84.14 | 7.14 |
| PDK1 | NM_002610 | 79.03 | 2.47 | 83.28 | 2.02 |
| MAP2K4 | NM_003010 | 93.01 | 8.56 | 85.85 | 8.07 |
| PDK2 | NM_002611 | 99.86 | 2.34 | 99.28 | 2.28 |
| RPS6KB2 | NM_003952 | 67.14 | 6.36 | 56.56 | 4.43 |
| CLK1 | NM_004071 | 94.95 | 3.04 | 84.82 | 5.38 |
| SFRS2 | NM_003016 | 95.41 | 4.66 | 84.91 | 12.05 |
| AURKB | NM_004217 | 35.59 | 1.21 | 31.24 | 5.32 |
| RPS6KA5 | NM_004755 | 98.65 | 0.05 | 77.85 | 1.22 |
| CENPC1 | NM_001812 | 97.05 | 6.62 | 96.81 | 1.06 |
| CD44 | NM_000610 | 111.44 | 6.82 | 119.87 | 0.35 |
| MAPK1 | NM_138957 | 75.71 | 4.71 | 84.76 | 7.27 |
| MAP3K14 | NM_003954 | 96.93 | 9.53 | 59.15 | 11.87 |
| PLK3 | NM_004073 | 83.71 | 3.93 | 103.53 | 0.14 |
| DAPK2 | NM_014326 | 94.8 | 3.48 | 75.15 | 8.02 |
| ACVR2A | NM_001616 | 91.3 | 5.6 | 83.05 | 6.08 |
| CDC16 | NM_003903 | 64.31 | 2 | 72.83 | 3.83 |
| MAP3K11 | NM_002419 | 108.04 | 11.43 | 103.21 | 4.31 |
| FLT4 | NM_002020 | 95.47 | 7.08 | 102.06 | 6.48 |
| ADRBK1 | NM_001619 | 80.97 | 4.2 | 71.85 | 2.69 |
| STARD4 | NM_139164 | 102.83 | 2.59 | 114.62 | 1.53 |
| GART | NM_000819 | 86.56 | 7.54 | 69.39 | 7.49 |
| CAMK2B | NM_001220 | 89.04 | 1.18 | 81.04 | 1.7 |
| SMAD2 | NM_005901 | 81.14 | 6.74 | 89.09 | 5.49 |
| EIF2S2 | NM_003908 | 45.85 | 6.73 | 41.8 | 4.78 |
| WNT4 | NM_030761 | 68.07 | 3.87 | 80.47 | 2.85 |
| CAMK2D | NM_001221 | 84.26 | 3.29 | 67.18 | 5.62 |
| RASA3 | NM_007368 | 95.23 | 1.19 | 89.19 | 7.31 |
| STX16 | NM_003763 | 67.22 | 3.05 | 66.27 | 3.5 |
| ARFRP1 | NM_003224 | 93.12 | 4.27 | 53.45 | 6.08 |
| CHEK1 | NM_001274 | 83.83 | 3.98 | 64.36 | 4.53 |
| CENPE | NM_001813 | 24.45 | 1.44 | 44.66 | 4.15 |
| CHUK | NM_001278 | 87.58 | 11.6 | 85.26 | 5.79 |
| IGF1R | NM_000875 | 90.05 | 5.58 | 54.38 | 10.03 |
| RASA2 | NM_006506 | 116 | 16.82 | 122.31 | 3.28 |
| PIK3CD | NM_005026 | 81.44 | 4.2 | 76.78 | 2.09 |
| RNPC2 | NM_004902 | 55.73 | 0.6 | 48.24 | 3.12 |
| PIK3R2 | NM_005027 | 78.35 | 7.78 | 68.5 | 8.51 |
| LAMA5 | NM_005560 | 109.7 | 3.1 | 120.16 | 6.02 |
| STK17B | NM_004226 | 124.5 | 6.81 | 110.76 | 1.14 |
| STK17A | NM_004760 | 73.33 | 2.24 | 78.11 | 8.68 |
| CKS2 | NM_001827 | 74.81 | 5.05 | 76.48 | 10.03 |
| STMN1 | NM_005563 | 88.69 | 11.82 | 79.5 | 16.14 |
| TOP1 | NM_003286 | 66.19 | 2.73 | 68.44 | 3.32 |
| NEK1 | AB067488 | 97.9 | 5.41 | 111.18 | 1.77 |
| TPD52L2 | NM_003288 | 101.13 | 4.45 | 111.34 | 5.71 |
| MAPK12 | NM_002969 | 84.43 | 7.03 | 84.99 | 0.4 |
| HUNK | NM_014586 | 105.43 | 2.4 | 115.14 | 16.09 |
| C20orf35 | NM_018478 | 85.81 | 1.68 | 98.68 | 6.22 |
| KIF19 | NM_153209 | 89.84 | 0.64 | 102.86 | 1.96 |
| CCNA1 | NM_003914 | 90.4 | 5.22 | 86.72 | 0.62 |
| GUCY2C | NM_004963 | 103.93 | 0.72 | 107.31 | 2.96 |
| MOS | NM_005372 | 102.53 | 1.39 | 90.23 | 10.3 |
| HDAC1 | NM_004964 | 90.44 | 3.88 | 65.01 | 10.32 |
| KCNK15 | NM_022358 | 86.87 | 16.55 | 107.23 | 11.3 |
| AKT2 | NM_001626 | 49.63 | 7.53 | 65.5 | 4.57 |
| FRK | NM_002031 | 76.93 | 13.91 | 88.8 | 0.23 |
| CDC14A | NM_033312 | 100.37 | 6 | 94.91 | 1.67 |
| MYB | NM_005375 | 95.36 | 5.7 | 113.08 | 9.44 |
| AHCY | NM_000687 | 99.01 | 7.57 | 100.37 | 4.31 |
| MYCL1 | NM_005376 | 92.2 | 0.03 | 113.89 | 0.73 |
| DBF4 | NM_006716 | 83.32 | 0.45 | 75.99 | 2.57 |
| RGL2 | NM_004761 | 102.78 | 13.91 | 92.43 | 0.29 |
| IMPDH1 | NM_000883 | 77.08 | 6.26 | 81.65 | 2.03 |
| SHC1 | NM_003029 | 106.26 | 2.87 | 112.03 | 3.05 |
| ATP5B | NM_001686 | 102.01 | 5.21 | 47.95 | 13.15 |
| JAK1 | NM_002227 | 70.69 | 0.39 | 72.46 | 4.45 |
| IMPDH2 | NM_000884 | 69.75 | 15.44 | 78.1 | 7.79 |
| GRK6 | NM_002082 | 79.39 | 1.71 | 76.69 | 9.45 |
| C10orf3 | NM_018131 | 50 | 3.42 | 38.59 | 11.22 |
| JUN | NM_002228 | 87.54 | 0.43 | 92.54 | 3.01 |
| ASPM | NM_018136 | 79.31 | 8.72 | 47.71 | 9.55 |
| JUNB | NM_002229 | 75.06 | 7.42 | 100.65 | 1.44 |
| PLK1 | NM_005030 | 3.6 | 0.86 | 3.13 | 0.46 |
| CHMP4B | NM_176812 | 100.45 | 3.16 | 115.02 | 5.11 |
| C20orf77 | NM_021215 | 98.06 | 4.44 | 108.69 | 7.8 |
| GRB2 | NM_002086 | 88.54 | 0.31 | 106.2 | 2.5 |
| PTK6 | NM_005975 | 70.98 | 4.09 | 75.49 | 2.94 |
| MYCN | NM_005378 | 97.95 | 2.47 | 89.88 | 2.48 |
| MCM6 | NM_005915 | 98.77 | 2.33 | 98.7 | 6.55 |
| FYN | NM_002037 | 82.26 | 0.32 | 89.51 | 12.85 |
| TNIK | AF172264 | 104.11 | 4.01 | 97.31 | 4.97 |
| MAP4K2 | NM_004579 | 81.62 | 6.2 | 81.89 | 8.1 |
| MPHOSPH1 | NM_016195 | 104.04 | 9.58 | 69.79 | 22.43 |
| GAB1 | NM_002039 | 99.1 | 14.12 | 106.61 | 5.56 |
| JAK2 | NM_004972 | 85.76 | 7.66 | 70.07 | 8.63 |
| CCNA2 | NM_001237 | 39.26 | 2.68 | 49.79 | 0.09 |
| SNTA1 | NM_003098 | 113.25 | 7.33 | 114.73 | 3.92 |
| NOTCH3 | NM_000435 | 85.6 | 13.21 | 98.29 | 2.84 |
| RAPGEF6 | NM_016340 | 103.74 | 13.2 | 108.03 | 0.1 |
| CCNE1 | NM_001238 | 90.08 | 1.26 | 76.26 | 7.6 |
| TUBB6 | NM_032525 | 85.27 | 8.66 | 83.49 | 9.59 |
| CDC42 | NM_044472 | 96.49 | 6.71 | 65.86 | 0.39 |
| DDR1 | NM_013994 | 99.89 | 8.35 | 71.86 | 5.49 |
| RALGDS | U14417 | 112.65 | 13.77 | 103.45 | 13.93 |
| S100A2 | NM_005978 | 123.47 | 17.38 | 107.6 | 10.77 |
| NEK9 | NM_033116 | 92.36 | 3.29 | 90.96 | 4.11 |
| EIF4EBP2 | NM_004096 | 102.57 | 5.22 | 92.89 | 6.78 |
| MYLK2 | NM_033118 | 78.27 | 1.33 | 96.41 | 3.43 |
| NEK2 | NM_002497 | 100.93 | 8.53 | 83.89 | 16.47 |
| ABL1 | NM_007313 | 80.02 | 0.47 | 93.49 | 3.03 |
| GSK3A | NM_019884 | 68.92 | 0.03 | 77.4 | 4.48 |
| ABL2 | NM_007314 | 85.31 | 7.37 | 103.52 | 2.08 |
| MASK | NM_016542 | 104.28 | 9.37 | 97.65 | 1.47 |
| STAT1 | NM_007315 | 94.68 | 5.72 | 115 | 3.07 |
| HES7 | NM_032580 | 69.61 | 28.01 | 105.86 | 1.73 |
| CCNH | NM_001239 | 100.37 | 4.15 | 83.44 | 10.78 |
| CENPF | NM_016343 | 100.62 | 0.86 | 97.8 | 5.27 |
| PKD2 | NM_000297 | 96.05 | 2.8 | 113.6 | 10.83 |
| DNCH1 | U53530 | 63.34 | 0.26 | 64.54 | 1.88 |
| MAP3K13 | NM_004721 | 83.67 | 1.83 | 63.54 | 10.28 |
| TM9SF4 | NM_014742 | 104.12 | 6.6 | 96.69 | 1.42 |
| NTRK2 | NM_006180 | 76.52 | 3.1 | 93.21 | 9.78 |
| MAP3K4 | NM_005922 | 86.12 | 1.33 | 96.03 | 1.19 |
| CAMK2G | BC021269 | 63.7 | 1.2 | 83.32 | 6.8 |
| MAP3K5 | NM_005923 | 95.84 | 9.15 | 100.64 | 6.69 |
| ARHGEF2 | NM_004723 | 73.43 | 7.81 | 97.33 | 6.87 |
| CENPH | NM_022909 | 89.74 | 3.85 | 81.61 | 3.12 |
| DDR2 | NM_006182 | 87.35 | 7.42 | 74.77 | 11.99 |
| WNK4 | NM_032387 | 120.23 | 15.49 | 103.15 | 0.57 |
| ZW10 | NM_004724 | 95.15 | 4.57 | 97.39 | 6.71 |
| TNK2 | NM_005781 | 77.5 | 2.25 | 84.81 | 2.1 |
| KIF22 | NM_007317 | 74.69 | 3.58 | 88.89 | 0.92 |
| SFRS1 | NM_006924 | 67.05 | 4.7 | 64.13 | 1.23 |
| ASXL1 | NM_015338 | 113.72 | 0.55 | 126.94 | 9.62 |
| MAP2K7 | NM_005043 | 114.64 | 8.05 | 99.24 | 2.47 |
| ADNP | NM_015339 | 89.5 | 5.87 | 68.49 | 1.83 |
| GHRH | NM_021081 | 67.24 | 9.48 | 69.3 | 0.54 |
| LMNA | NM_005572 | 60.76 | 2.49 | 74.31 | 7.63 |
| PRKCBP1 | NM_012408 | 75.35 | 5.93 | 74.79 | 8.46 |
| STK25 | NM_006374 | 30.52 | 3.07 | 33.78 | 4.18 |
| MAPK10 | NM_138982 | 99 | 2.48 | 100.88 | 1.08 |
| ARPC3 | NM_005719 | 30.03 | 3.18 | 9.78 | 5.59 |
| SKP2 | NM_005983 | 40.33 | 2 | 46.36 | 2.82 |
| LMNB1 | NM_005573 | 103.43 | 0.82 | 124.69 | 4.59 |
| RALBP1 | NM_006788 | 46.72 | 1.54 | 45.91 | 5.78 |
| NEK6 | NM_014397 | 106.01 | 7.61 | 113.01 | 9.04 |
| PIK3C2A | NM_002645 | 97.73 | 15.95 | 109.58 | 13.96 |
| BUB3 | NM_004725 | 95.46 | 5.74 | 108.81 | 4.62 |
| TGFBR2 | NM_003242 | 113.29 | 3.17 | 96.66 | 10.46 |
| NUMA1 | NM_006185 | 79.23 | 2.76 | 73.39 | 4.16 |
| KIF5A | NM_004984 | 115.36 | 5.99 | 68.28 | 32.18 |
| CCNT1 | NM_001240 | 110.42 | 1.64 | 108.53 | 5.5 |
| B2M | NM_004048 | 111.94 | 1.3 | 85.27 | 10.38 |
| RPS6KA3 | NM_004586 | 106.3 | 1.58 | 99.81 | 5.52 |
| APLP2 | NM_001642 | 104.76 | 4.97 | 94.94 | 1.55 |
| CSE1L | NM_177436 | 66.72 | 4.12 | 65.68 | 3.24 |
| KRAS | NM_004985 | 49.98 | 25.47 | 79.42 | 3.7 |
| CCNK | BC015935 | 75.6 | 10.84 | 72.06 | 6.38 |
| MAP3K10 | NM_002446 | 87.74 | 10.53 | 56.83 | 1.54 |
| PBK | NM_018492 | 81.28 | 8.8 | 89 | 0.31 |
| MST1R | NM_002447 | 82.39 | 2.2 | 88.49 | 6.06 |
| DCAMKL1 | NM_004734 | 68.6 | 1.91 | 82.78 | 4.56 |
| CDC14B | NM_033331 | 105.14 | 9.93 | 107.11 | 0.75 |
| ERN1 | NM_001433 | 97.65 | 1.22 | 76.13 | 2.07 |
| PIK3C3 | NM_002647 | 75.09 | 0.9 | 82.49 | 6.61 |
| BCL2 | NM_000633 | 97.03 | 6.41 | 89.51 | 3.09 |
| PIM1 | NM_002648 | 111.45 | 11.55 | 59.01 | 1.33 |
| GSK3B | NM_002093 | 82.88 | 2.79 | 77.37 | 2.04 |
| PIK3CG | NM_002649 | 119.83 | 3.93 | 104 | 9.77 |
| NEK7 | NM_133494 | 104.59 | 2.65 | 91.94 | 1.8 |
| FGFR2 | NM_022972 | 108.93 | 0.25 | 88.13 | 1.23 |
| ZNF313 | NM_018683 | 80 | 4.59 | 73.87 | 2.68 |
| SCAND1 | NM_016558 | 79.08 | 1.93 | 64.24 | 0.99 |
| ANLN | NM_018685 | 77.24 | 3.53 | 66.47 | 3.5 |
| STK10 | NM_005990 | 95.98 | 24.47 | 58.13 | 0.8 |
| ACTR3 | NM_005721 | 90.39 | 1.28 | 105.25 | 1.28 |
| MYO3B | NM_138995 | 80.17 | 1.05 | 93.23 | 5.18 |
| ULK4 | NM_017886 | 81.69 | 2.23 | 94.86 | 1.7 |
| COL9A3 | NM_001853 | 79.15 | 3.28 | 68.66 | 2.05 |
| KIFC2 | NM_145754 | 93.68 | 4.32 | 96.14 | 3.06 |
| DIAPH3 | NM_030932 | 101.1 | 3.47 | 119.8 | 0.73 |
| TOP3B | NM_003935 | 95.6 | 1.8 | 98.29 | 7.34 |
| SLCO4A1 | NM_016354 | 81.58 | 9.74 | 84.33 | 6.49 |
| CDK5R2 | NM_003936 | 103.92 | 4.92 | 104.2 | 6.34 |
| DLG7 | NM_014750 | 79.62 | 1.27 | 26.86 | 3.49 |
| VAPB | NM_004738 | 105.46 | 6.35 | 93.45 | 4.09 |
| NOTCH1 | AF308602 | 118.57 | 2.35 | 92.72 | 10.5 |
| CDC5L | NM_001253 | 68.16 | 0.79 | 70.08 | 2.51 |
| RAP2A | NM_021033 | 103.56 | 4.2 | 100.18 | 1.1 |
| CDC6 | NM_001254 | 76.69 | 7.27 | 82.99 | 0.72 |
| THRA | NM_003250 | 91.53 | 7.9 | 106.66 | 8.25 |
| CDC20 | NM_001255 | 91.25 | 4.79 | 94.41 | 14.16 |
| C20orf23 | AK000142 | 88.64 | 3.18 | 104.74 | 13.94 |
| ACTR2 | NM_005722 | 97.15 | 1.54 | 67.27 | 0.09 |
| PLAGL2 | NM_002657 | 102.5 | 4.52 | 106.43 | 12.09 |
| MAP4K1 | NM_007181 | 113.54 | 3.13 | 103.75 | 6.31 |
| PLAU | NM_002658 | 108.87 | 7.34 | 98.53 | 2.65 |
| ZNF217 | NM_006526 | 91.63 | 5.23 | 102.96 | 2.52 |
| TUBA1 | NM_006000 | 13.94 | 2.01 | 18.34 | 5.94 |
| CEP2 | NM_007186 | 77.73 | 5.21 | 90 | 5.4 |
| MAP3K8 | NM_005204 | 89.44 | 1.95 | 104.87 | 2.98 |
| PRC1 | NM_003981 | 53.73 | 2.11 | 74.05 | 4.05 |
| RPS6KC1 | NM_012424 | 94.98 | 0.91 | 107.91 | 2.34 |
| NCOA5 | NM_020967 | 109.14 | 3.16 | 122.26 | 4.93 |
| CRKL | NM_005207 | 96.51 | 2.77 | 99.65 | 17.07 |
| POFUT1 | NM_015352 | 103.47 | 9.89 | 102.23 | 13.31 |
| DMPK | NM_004409 | 99.86 | 4.46 | 95.84 | 2.81 |
| CDC27 | NM_001256 | 36.36 | 8.47 | 40.82 | 1.64 |
| ARAF | NM_001654 | 101.14 | 8.76 | 87.82 | 0.69 |
| CTNNAL1 | NM_003798 | 105.06 | 7.82 | 85.39 | 1.43 |
| TK1 | NM_003258 | 96.81 | 9.6 | 96.43 | 7.75 |
| STK11 | NM_000455 | 82.26 | 2.43 | 90.18 | 0.59 |
| CDCA8 | NM_018101 | 86.04 | 11.33 | 87.82 | 2 |
| CDK3 | NM_001258 | 99.61 | 3.17 | 113.1 | 1.43 |
| GUK1 | NM_000858 | 97.93 | 11.02 | 93.89 | 5.98 |
| CDK6 | NM_001259 | 105.35 | 5.44 | 110.79 | 14.53 |
| HMGCR | NM_000859 | 84.93 | 0.49 | 60.05 | 45.07 |
| SDC4 | NM_002999 | 93.29 | 4.43 | 73.84 | 8.92 |
| DLGAP4 | NM_014902 | 73.62 | 0.11 | 82.42 | 3.9 |
| HES6 | NM_018645 | 100.64 | 4.81 | 88.81 | 9.11 |
| CRKRS | NM_016507 | 78.31 | 6.87 | 100.38 | 5.54 |
| MAD2L2 | NM_006341 | 82.17 | 4.93 | 101.25 | 9.77 |
| CDKL3 | NM_016508 | 111.57 | 6.18 | 100.12 | 10 |
| AXL | NM_021913 | 96.53 | 10.28 | 87.05 | 1.55 |
| PLCG1 | NM_002660 | 92.86 | 4.96 | 75.87 | 1.65 |
| C20orf11 | NM_017896 | 82.77 | 1.68 | 75.01 | 3.18 |
| PLCG2 | NM_002661 | 77.86 | 13.25 | 65.32 | 0.5 |
| KIF20A | NM_005733 | 77.11 | 2.95 | 77.18 | 3.51 |
| PLD1 | NM_002662 | 105.29 | 9.66 | 87.96 | 5.37 |
| CHEK2 | NM_007194 | 72.56 | 1.74 | 68.42 | 8.64 |
| CDC91L1 | NM_080476 | 96.66 | 3.7 | 101.02 | 0.92 |
| CDK5 | NM_004935 | 90.76 | 6.59 | 94.81 | 11.32 |
| ESR1 | NM_000125 | 104.83 | 9.37 | 92.54 | 7.82 |
| CDKN2B | NM_004936 | 104.63 | 1.46 | 92.26 | 7.54E-03 |
| CSF1R | NM_005211 | 82.67 | 6.45 | 72.69 | 0.31 |
| RASGRP1 | NM_005739 | 92.04 | 0.29 | 116.33 | 6.43 |
| SAMD10 | NM_080621 | 77.54 | 1.75 | 88.09 | 1.91 |
| DAPK1 | NM_004938 | 91.65 | 14.07 | 99.54 | 9.09 |
| RAE1 | NM_003610 | 79 | 2.97 | 68.25 | 11.39 |
| NDRG3 | NM_032013 | 136.35 | 15.38 | 105.84 | 2.07 |
| SNRK | NM_017719 | 95.29 | 1.78 | 106.2 | 14.67 |
| KIF18A | NM_031217 | 86.01 | 6.39 | 104.03 | 4.14 |
| SHMT2 | NM_005412 | 118.72 | 4.84 | 121.8 | 7.89 |
| C20orf108 | NM_080821 | 108.52 | 9.7 | 70.54 | 5.53 |
| TGFBR1 | NM_004612 | 104.62 | 1.19 | 119.31 | 0.77 |
| PIK3CA | NM_006218 | 63.99 | 0.62 | 73.66 | 16.97 |
| CAMK4 | NM_001744 | 100.67 | 2.32 | 113.1 | 2.91 |
| DNTTIP1 | NM_052951 | 87.08 | 2.56 | 107.54 | 1.08 |
| PRIM1 | NM_000946 | 101.51 | 8.63 | 95 | 14.93 |
| PRIM2A | NM_000947 | 100.21 | 1.41 | 92.14 | 3.45 |
| RNASEL | NM_021133 | 99.56 | 8.28 | 86.44 | 5.12 |
| CCNI | NM_006835 | 92.07 | 1.87 | 96.45 | 1.86 |
| RPS6KA2 | NM_021135 | 94.31 | 4.99 | 90.63 | 9.39 |
| TK2 | NM_004614 | 116.28 | 8.72 | 91.87 | 0.01 |
| PIK3CB | NM_006219 | 99.86 | 14.63 | 114.2 | 0.02 |
| SRC | NM_005417 | 131.11 | 3.92 | 111.52 | 6.4 |
| ST5 | NM_005418 | 102.9 | 0.64 | 95.16 | 11.37 |
| TNNI3K | NM_015978 | 104.83 | 6.6 | 98.12 | 0.51 |
| STAT2 | NM_005419 | 92.17 | 11.19 | 70.7 | 2.69 |
| TOP3A | NM_004618 | 111.41 | 1.71 | 116.29 | 6.55 |
| GPSM2 | NM_013296 | 105.07 | 2.56 | 118.77 | 6.17 |
| STK38L | BC028603 | 98.3 | 5.79 | 103.81 | 15.8 |
| PPP2CB | NM_004156 | 106.75 | 2.12 | 105.21 | 10.76 |
| BLCAP | NM_006698 | 71.14 | 0.05 | 67.35 | 5.29 |
| FBXO5 | NM_012177 | 34.23 | 2.1 | 36.57 | 2.2 |
| TOP1MT | NM_052963 | 81.72 | 0.28 | 90.67 | 11.62 |
| CCND2 | NM_001759 | 72.55 | 3.53 | 69.84 | 14.48 |
| CKAP2 | NM_018204 | 119.28 | 2.92 | 136.4 | 1.8 |
| TCF3 | M31523 | 91.88 | 5.23 | 100.15 | 2.9 |
| CDK10 | NM_003674 | 113.49 | 1.56 | 95.65 | 1.04 |
| HPRT1 | NM_000194 | 115.87 | 2.02 | 94.55 | 7.1 |
| MAPKAPK2 | NM_032960 | 93.19 | 9.65 | 86.59 | 9.11 |
| CAMK2A | NM_015981 | 69.28 | 5.08 | 59.89 | 0.23 |
| TOPBP1 | NM_007027 | 102.27 | 1.83 | 93.12 | 9.03 |
| TIE1 | NM_005424 | 92.49 | 6.1 | 92.15 | 12.92 |
| KIF12 | NM_138424 | 113.02 | 1.15 | 91.04 | 12.17 |
| ACVR1B | NM_004302 | 83.81 | 4.89 | 79.86 | 0.61 |
| KIF2C | NM_006845 | 58.96 | 0.04 | 30.7 | 8.32 |
| ALK | NM_004304 | 110.38 | 15.21 | 102.58 | 1.45 |
| CDC7 | NM_003503 | 87.84 | 13.2 | 80.17 | 9.26 |
| MAPRE1 | NM_012325 | 93.39 | 0.56 | 76.23 | 9.25 |
| CDC45L | NM_003504 | 34.07 | 5.74 | 17.35 | 8.35 |
| MAPRE3 | NM_012326 | 31.63 | 11.67 | 30.84 | 10.12 |
| RIPK2 | NM_003821 | 88.79 | 4.54 | 87.99 | 1.8 |
| RBX1 | NM_014248 | 76.78 | 7.07 | 60.04 | 1.35 |
| STK35 | NM_080836 | 115.74 | 6.65 | 31.21 | 3.99 |
| VAV1 | NM_005428 | 75.6 | 1.26 | 48.39 | 2.82 |
| WNT7B | NM_058238 | 97.2 | 9.81 | 96.34 | 3.6 |
| CLK4 | NM_020666 | 90.39 | 5.17 | 82.84 | 10.07 |
| TUBB4 | NM_006087 | 64.82 | 0.04 | 46.7 | 2.33 |
| RAF1 | NM_002880 | 62.84 | 4.64 | 84.57 | 12.63 |
| OXSR1 | NM_005109 | 96.17 | 8.9 | 110.82 | 2.93 |
| FZD1 | NM_003505 | 102.96 | 4.1 | 123.96 | 7.41 |
| CTNNA1 | NM_001903 | 102.23 | 11.04 | 80.04 | 6.78 |
| FZD9 | NM_003508 | 80.4 | 7.46 | 77.84 | 1.85 |
| RRAD | NM_004165 | 84.14 | 5.8 | 101.56 | 0.34 |
| ADA | NM_000022 | 105.49 | 1.92 | 120.84 | 1.92 |
| CCND3 | NM_001760 | 96.48 | 2.62 | 42.02 | 2.52 |
| RALB | NM_002881 | 74.69 | 2.79 | 59.71 | 5.03 |
| MKNK1 | NM_003684 | 105.57 | 1.73 | 73.2 | 14.5 |
| RAP1A | NM_002884 | 105.96 | 8.45 | 94.61 | 5.96 |
| CASK | NM_003688 | 104.6 | 0.64 | 95.08 | 4.3 |
| AATK | AB014541 | 81.63 | 6.56 | 92.27 | 8.52 |
| APBA2BP | NM_031231 | 62.75 | 3.46 | 68.51 | 0.95 |
| POLE | NM_006231 | 93.83 | 5.92 | 92.61 | 1.35 |
| WNT1 | NM_005430 | 105.09 | 3.33 | 111.11 | 3.34 |
| CCNF | NM_001761 | 53.31 | 7.08 | 55.49 | 7.78 |
| SHMT1 | NM_004169 | 95.58 | 12.88 | 86.21 | 11.49 |
| AGA | NM_000027 | 100.2 | 4 | 97.81 | 10.41 |
| RARA | NM_000964 | 67.88 | 8.44 | 67.37 | 0.77 |
| RARB | NM_000965 | 95.48 | 10.94 | 103.7 | 6.13 |
| DNCL2A | NM_177953 | 59.08 | 0.51 | 68.29 | 20.54 |
| FAM64A | NM_019013 | 67.16 | 3.66 | 93.75 | 10.76 |
| FZD3 | NM_017412 | 80.77 | 2.7 | 102.37 | 9.81 |
| CCNT2 | NM_058241 | 105.56 | 3.29 | 109.62 | 7.64 |
| YES1 | NM_005433 | 49.87 | 0.61 | 48.22 | 4.18 |
| RAB22A | NM_020673 | 117.65 | 6.14 | 112.25 | 6.1 |
| MAPKAPK3 | NM_004635 | 90.54 | 1.91 | 78.36 | 2.91 |
| RASA1 | NM_002890 | 64.13 | 33.84 | 125.86 | 8.53 |
| RBL1 | NM_002895 | 120.74 | 10.69 | 105.66 | 8.36 |
| IFT52 | NM_016004 | 88.01 | 6.48 | 100.33 | 11.45 |
| RIPK5 | AB007941 | 93.73 | 1.68 | 95.52 | 3.17 |
| C20orf31 | NM_018217 | 98.31 | 2.55 | 99.59 | 1.65 |
| C20orf111 | NM_016470 | 77.07 | 4.18 | 85.5 | 4.02 |
| NEK3 | Z29067 | 93.09 | 4.19 | 88.83 | 2.5 |
| TLK2 | NM_006852 | 60.12 | 14.09 | 52.32 | 2.28 |
| TTN | NM_133378 | 65.26 | 2.12 | 46.81 | 4.79 |
|  | XM_066649 | 83.69 | 3.89 | 87.44 | 5.3 |
| PLK4 | NM_014264 | 82.78 | 5.59 | 36.1 | 7.03 |
| BAD | NM_032989 | 106.8 | 12.55 | 82.23 | 0.73 |
| CDK2AP1 | NM_004642 | 38.79 | 2.27 | 38.8 | 0.56 |
| MAPRE2 | NM_014268 | 98.53 | 6.76 | 32.14 | 5.16 |
| BIRC5 | NM_001168 | 47.6 | 3.76 | 48.69 | 2.47 |
| PRKAA2 | NM_006252 | 100.08 | 0.6 | 86.75 | 1.86 |
| KIF3A | NM_007054 | 127.51 | 3.38 | 95.46 | 9.19 |
| KIF1B | NM_015074 | 97.64 | 8.4 | 101.84 | 9.2 |
|  | M15077 | 113.92 | 7.98 | 103.16 | 1.33 |
|  | AF188479 | 88.61 | 5.13 | 77.27 | 13.68 |
| CDCA2 | NM_152562 | 79.99 | 0.12 | 61.19 | 2.46 |
|  | AI278633 | 76.26 | 3.41 | 62.82 | 2.31 |
| ELMO2 | NM_133171 | 88.49 | 0.32 | 98.76 | 4.9 |
| LOC158301 | AI338451 | 17.55 | 0.47 | 7.89 | 2.58 |
| NBN | NM_002485 | 92.84 | 0.66 | 110.13 | 2.84 |
| BRIP1 | NM_032043 | 99.84 | 9.84 | 123.02 | 13.81 |
| HCK | NM_002110 | 109.75 | 12.63 | 98.9 | 7.99 |
| PRKCD | NM_006254 | 72.73 | 5.21 | 82.13 | 4.53 |
| PRKCH | NM_006255 | 120.24 | 7.23 | 104.81 | 0.9 |
| PKN2 | NM_006256 | 118.45 | 0.82 | 88.65 | 21.5 |
| PRKCQ | NM_006257 | 116.33 | 8.6 | 99.02 | 9.12 |
| PRKG1 | NM_006258 | 104.24 | 4.31 | 106.35 | 7.06 |
| PRKG2 | NM_006259 | 76.15 | 0.84 | 68.49 | 5.41 |
| AXIN2 | NM_004655 | 98 | 7.06 | 114.68 | 5.18 |
| SHFM1 | NM_006304 | 113.39 | 6.01 | 91.48 | 5.36 |
| PCTK3 | BC011526 | 98.02 | 3.81 | 89.99 | 5.18 |
| DNMT3A | NM_175629 | 101.82 | 8.17 | 95.43 | 0.89 |
| ANAPC7 | NM_016238 | 95 | 1.86 | 89.94 | 2.1 |
| C20orf177 | NM_022106 | 85.65 | 1.21 | 81.14 | 0.09 |
| CPNE1 | NM_152925 | 61.61 | 2.12 | 56.38 | 1.65 |
| KIF1C | AB014606 | 81.36 | 3.02 | 98.38 | 5.78 |
| KIF17 | AB037826 | 99.69 | 4.58 | 119.93 | 6.57 |
| MAPK14 | NM_001315 | 82.5 | 3.42 | 88.37 | 15.32 |
| DPM1 | NM_003859 | 113.23 | 1.06 | 111.05 | 4.02 |
| PROCR | NM_006404 | 92.24 | 2 | 101.52 | 2.87 |
| CAMKV | NM_024046 | 122.08 | 2.34 | 119.86 | 12.07 |
| MGC5566 | NM_024049 | 77.69 | 6.57 | 89.55 | 7.09 |
| KIAA1333 | NM_017769 | 106.03 | 13.48 | 119.05 | 2.09 |
| KALRN | NM_007064 | 82.14 | 1.78 | 93.66 | 3.61 |
| HES2 | BC012091 | 103.99 | 5.97 | 96.89 | 3.62 |
| WFDC8 | NM_181510 | 111.33 | 14.83 | 113.81 | 0.71 |
| BXDC1P | NM_178472 | 101.11 | 9.6 | 100.44 | 6.82 |
| HM13 | NM_178580 | 80.55 | 26.93 | 108.88 | 1.64 |
| SULF2 | NM_018837 | 60.8 | 12.15 | 27.09 | 16.41 |
| MYT1 | NM_004535 | 103.39 | 9.67 | 114.71 | 7.86 |
| PHACTR3 | NM_080672 | 90.53 | 8.09 | 104.49 | 0.87 |
| CDC37 | NM_007065 | 105.67 | 3.79 | 101.95 | 4.03 |
| PTK2 | NM_005607 | 83.28 | 6.87 | 72.85 | 13.01 |
| CDC23 | NM_004661 | 83.47 | 7.28 | 99.62 | 1.28 |
| AKT3 | NM_005465 | 102.64 | 2.53 | 96.59 | 9.04 |
| CSTF1 | NM_001324 | 105.45 | 4.39 | 113.94 | 6.44 |
| ACOT8 | NM_005469 | 76.86 | 3.53 | 73.4 | 5.37 |
| CTBP1 | NM_001328 | 107.49 | 4.85 | 104.11 | 0.37 |
| LDLR | NM_000527 | 99.81 | 8.8 | 119.42 | 6.06 |
| KIF21B | AL137395 | 100.41 | 3.31 | 110.33 | 4.25 |
| C9orf48 | NM_194313 | 104.01 | 6.88 | 99.05 | 5.8 |
| PIWIL1 | NM_004764 | 76.37 | 9.69 | 85.71 | 4 |
| KIF26B | BC035896 | 60.55 | 3.44 | 49.8 | 1.59 |
| KIAA0406 | NM_014657 | 88.33 | 6.09 | 99.34 | 2.52 |
| ZNFX1 | NM_021035 | 92.65 | 5.32 | 108.04 | 9.22 |
| C20orf152 | NM_080834 | 96.61 | 8.32 | 101.9 | 7.54 |
| PRIC285 | NM_033405 | 87.81 | 5.8 | 93.98 | 0.49 |
| KIF14 | NM_014875 | 61.18 | 0.1 | 49.77 | 4.27 |
| TRIB3 | NM_021158 | 85.13 | 2.88 | 117.78 | 10.82 |
| VAV2 | NM_003371 | 110.2 | 4.86 | 99.54 | 18.77 |
| PPP2CA | NM_002715 | 28.32 | 0.22 | 30.39 | 0.18 |
| RAP1GDS1 | NM_021159 | 110.17 | 5.01 | 83.14 | 3.39 |
| MAP2K6 | NM_002758 | 102.62 | 2.25 | 75.45 | 5.83 |
| EIF2AK2 | NM_002759 | 110.92 | 0.03 | 105.05 | 1.81 |
| STK32B | NM_018401 | 87.7 | 7.09 | 110.45 | 15.18 |
| CDK4 | NM_000075 | 72.09 | 7.53 | 57.45 | 11.86 |
| CDKN1C | NM_000076 | 87.37 | 6.33 | 105.02 | 6.62 |
| CDKN2A | NM_000077 | 90.35 | 7.65 | 100.14 | 1.22 |
| MASTL | NM_032844 | 96.73 | 7.23 | 86.97 | 3.43 |
| FZD4 | NM_012193 | 96.47 | 4.2 | 103.98 | 9.26 |
| PAK1 | NM_002576 | 109.62 | 9.69 | 111.23 | 8.09 |
| PAK2 | NM_002577 | 70.74 | 0.44 | 79.73 | 11.48 |
| APC | NM_000038 | 71.88 | 4.89 | 75.05 | 6.06 |
| C20orf67 | NM_022104 | 80.32 | 6.7 | 83.3 | 6.98 |
| DATF1 | NM_022105 | 86.14 | 6.77 | 89.39 | 4.38 |
| KIF1A | NM_004321 | 65.75 | 5.78 | 63.11 | 7.19 |
| NUP153 | NM_005124 | 58.97 | 7.25 | 63.97 | 8.73 |
| KNTC2 | NM_006101 | 10.1 | 1.43 | 12.39 | 0.39 |
| HDAC11 | NM_024827 | 68.46 | 16.28 | 33.87 | 0.17 |
| ADRBK2 | NM_005160 | 104.42 | 0.79 | 83.82 | 7.49 |
| GRK4 | NM_005307 | 94.21 | 6.97 | 41.76 | 0.73 |
| CDH1 | NM_004360 | 105.92 | 0.37 | 102.64 | 1.11 |
| PPARG | NM_015869 | 98.38 | 7.29 | 122.68 | 16.07 |
| AKT1 | NM_005163 | 78.41 | 5.04 | 58.23 | 6.24 |
| PRKY | NM_002760 | 79.37 | 1.41 | 91.47 | 5.86 |
| ANAPC10 | NM_014885 | 73.67 | 6.48 | 83.85 | 6.35 |
| BCR | NM_004327 | 77.01 | 6.31 | 88.46 | 2.69 |
| BMPR1A | NM_004329 | 113.03 | 6.64 | 106.8 | 1.19 |
| VRK1 | NM_003384 | 75.26 | 2.06 | 82.63 | 2.81 |
| AR | NM_000044 | 106.97 | 0.33 | 100.66 | 9.75 |
| CDC2 | NM_001786 | 41.09 | 4.43 | 58.52 | 4.92 |
| CDC25A | NM_001789 | 87.57 | 4.83 | 119.69 | 4.93 |
| GMEB2 | NM_012384 | 42.69 | 2.13 | 25.4 | 3.43 |
| REL | NM_002908 | 91.35 | 2.68 | 78.36 | 7.29 |
| ULK1 | NM_003565 | 124.28 | 8.86 | 80.24 | 7.34 |
| RPS21 | NM_001024 | 35.34 | 2.69 | 27.52 | 5.95 |
| KIT | NM_000222 | 96.89 | 6 | 104.54 | 6.19 |
| EGR1 | NM_001964 | 63.43 | 5.41 | 68.88 | 5.31 |
| DKFZp762E1312 | NM_018410 | 90.94 | 4.99 | 114.84 | 6.07 |
| MYO3A | NM_017433 | 77.88 | 1.24 | 88.39 | 3.64 |
| KIF13A | NM_022113 | 88.67 | 1.22 | 78.1 | 2.54 |
| ECT2 | NM_018098 | 26.63 | 1.68 | 15.84 | 3.51 |
| HES4 | NM_021170 | 88.82 | 6.48 | 77.2 | 11.36 |
| KIAA1285 | NM_015694 | 103.45 | 10.6 | 118.38 | 7.43 |
| BRAF | NM_004333 | 73.04 | 4.93 | 94.51 | 7.67 |
| PRKACA | NM_002730 | 90.76 | 2.79 | 91.6 | 7.03 |
| PIM2 | NM_006875 | 94.41 | 1.48 | 94.49 | 3.12 |
| C20orf20 | NM_018270 | 76.49 | 3.96 | 98.89 | 6.57 |
| VAV3 | NM_006113 | 86.03 | 4.4 | 98.42 | 5.3 |
| MAP3K7IP1 | NM_006116 | 87.81 | 4.19 | 52.63 | 1.55 |
| ILK | NM_004517 | 107.61 | 7.88 | 115.03 | 12.1 |
| CDC2L5 | NM_003718 | 119.82 | 10.19 | 113.56 | 12.89 |
| RFC4 | NM_002916 | 74.09 | 1.84 | 74.29 | 2.65 |
| RRM1 | NM_001033 | 53.72 | 0.14 | 71.04 | 16.59 |
| PFTK1 | NM_012395 | 109.41 | 3.1 | 110.96 | 5.48 |
| PRKACB | NM_002731 | 107.36 | 9.45 | 113.99 | 6.25 |
| BUB1 | NM_004336 | 86.07 | 4.84 | 62.88 | 3.03 |
| WEE1 | NM_003390 | 4.74 | 1.36 | 2.45 | 0.38 |
| WNT2 | NM_003391 | 73.12 | 5.76 | 24.82 | 1.95 |
| PRKCA | NM_002737 | 65.08 | 3.45 | 60.03 | 2.33 |
| CDC25C | NM_001790 | 93.75 | 0.09 | 93.22 | 2.25 |
| PRKCB1 | NM_002738 | 72.22 | 3.55 | 61.9 | 9.98 |
| PCNA | NM_002592 | 66.95 | 5.93 | 84.33 | 10.01 |
| RRM2 | NM_001034 | 5.78 | 0.07 | 6.5 | 0.5 |
| LIG1 | NM_000234 | 26.2 | 26.09 | 46.88 | 6.33 |
| KIF2 | NM_004520 | 86.39 | 1.84 | 80.14 | 11.27 |
| KIF5B | NM_004521 | 83.01 | 3.83 | 60.08 | 2.05 |
| KIF5C | NM_004522 | 87.8 | 8.24 | 100.68 | 2.68 |
| WFDC2 | NM_080733 | 95.52 | 3.94 | 113.16 | 8.64 |
| KIF11 | NM_004523 | 5.77 | 1.8 | 3.17 | 0.86 |
| CREBBP | NM_004380 | 67.26 | 10.23 | 65.84 | 6.09 |
| ATM | NM_000051 | 90.1 | 0.4 | 98.33 | 12.58 |
| CDKL1 | NM_004196 | 94.81 | 5.8 | 42.58 | 3.99 |
| PRKCG | NM_002739 | 100.16 | 1.59 | 102.37 | 0.42 |
| BLM | NM_000057 | 91.55 | 3.6 | 69.38 | 9.48 |
| NEK11 | NM_024800 | 98.36 | 1.65 | 81.68 | 7.33 |
| CDK2 | NM_001798 | 89.22 | 3.3 | 89.82 | 9.02 |
| BRCA2 | NM_000059 | 74.37 | 4.09 | 82.01 | 2.61 |
| CSK | NM_004383 | 82.78 | 9.47 | 57.09 | 0.7 |
| DYRK3 | NM_003582 | 90.15 | 5.57 | 76.86 | 0.1 |
| GRK1 | NM_002929 | 87.57 | 0.4 | 116.78 | 6 |
| ERBB3 | NM_001982 | 91.98 | 2.57 | 74.09 | 6.73 |
| CTNNA2 | NM_004389 | 86.72 | 4.58 | 85.44 | 15.41 |
| CUL4B | NM_003588 | 71.56 | 1.62 | 41.34 | 0.3 |
| CUL4A | NM_003589 | 76.47 | 7.66 | 85.94 | 3.89 |
| CDK7 | NM_001799 | 82.4 | 1.08 | 35 | 7.24 |
| C20orf44 | NM_018244 | 107.19 | 5.34 | 105.2 | 4.45 |
| NPEPL1 | NM_024663 | 107.1 | 12.47 | 99.24 | 21.14 |
| CDK5RAP2 | NM_018249 | 82.88 | 4.41 | 68.64 | 4.36 |
| ATAD2 | NM_014109 | 110.54 | 4.52 | 100.99 | 1.16 |
| FAM61B | NM_144703 | 94.16 | 0.75 | 90.49 | 10.76 |
| EPHA8 | NM_020526 | 116.46 | 1.41 | 133.23 | 6.96 |
| PRKCI | NM_002740 | 59.82 | 33.07 | 53.27 | 42.19 |
| CCND1 | NM_053056 | 82.74 | 2.42 | 102.54 | 4.11 |
| MLH1 | NM_000249 | 101.48 | 0.96 | 113.04 | 18.13 |
| CDKN1A | NM_078467 | 120.56 | 15.77 | 124.27 | 16.23 |
| CCNC | NM_005190 | 78.91 | 7.88 | 75.9 | 7.33 |
| CDKN3 | NM_005192 | 76.8 | 3.56 | 88.04 | 16.44 |
| C20orf52 | NM_080748 | 80.85 | 2.74 | 84.64 | 7.14 |
| CUL3 | NM_003590 | 60.28 | 2.4 | 58.93 | 2.5 |
| CUL2 | NM_003591 | 96.58 | 8.93 | 89.44 | 4.33 |
| PKN1 | NM_002741 | 87.48 | 8.75 | 82.21 | 13.18 |
| ICAM1 | NM_000201 | 92.4 | 1.16 | 60.81 | 23.14 |
| ATP5E | NM_006886 | 93.65 | 9.2 | 62.08 | 1.07 |
| PRKD1 | NM_002742 | 80.72 | 8.88 | 104.36 | 7.73 |
| PRKCZ | NM_002744 | 86.29 | 5.56 | 91.68 | 1.89 |
| HBEGF | NM_001945 | 89.1 | 7.35 | 79.18 | 1.27 |
| MAPK4 | NM_002747 | 57.07 | 19.97 | 78.4 | 14.3 |
| MAPK6 | NM_002748 | 103.58 | 1.89 | 97.38 | 1.89 |
| CUL1 | NM_003592 | 95.17 | 2.62 | 111.85 | 9.96 |
| PSMA7 | NM_002792 | 74.08 | 3.26 | 75.17 | 4.4 |
| MSH2 | NM_000251 | 76.16 | 2.98 | 85.77 | 13.75 |
| DDX6 | NM_004397 | 91.61 | 6.78 | 96.34 | 15.19 |
| SHC3 | NM_016848 | 116.94 | 2.26 | 132.88 | 4.15 |
| HRAS | NM_005343 | 55.76 | 8.56 | 73.42 | 5.48 |
| ANAPC2 | NM_013366 | 76.33 | 7.25 | 78.53 | 1.48 |
| FES | NM_002005 | 80.08 | 12.06 | 84.97 | 13.28 |
| INSR | NM_000208 | 85.39 | 1.55 | 51.46 | 13.51 |
| STAU | NM_017453 | 105.65 | 6.7 | 89.8 | 8.35 |
| BTRC | NM_033637 | 120.45 | 8.66 | 141.81 | 6.05 |
| PCMTD2 | NM_018257 | 86.71 | 1.35 | 84.44 | 1.61 |
| SH3KBP1 | NM_031892 | 92.47 | 1.33 | 90.36 | 8.4 |
| MAD1L1 | NM_003550 | 89.79 | 6.42 | 118.13 | 14.01 |
| MAPK8 | NM_002750 | 108.81 | 4.44 | 105.1 | 7.22 |
| HSPCA | NM_005348 | 33.69 | 8.68 | 12.94 | 4.1 |
| BMPR1B | NM_001203 | 62.63 | 2.67 | 73.64 | 14.93 |
| ANAPC4 | NM_013367 | 83.16 | 10.31 | 109.71 | 19.43 |
| FGF2 | NM_002006 | 78.78 | 2.74 | 76.17 | 3.64 |
| BMPR2 | NM_001204 | 108.68 | 2.03 | 133.97 | 11.14 |
| ROS1 | NM_002944 | 91.4 | 7.19 | 140.87 | 11.48 |
| IRS2 | NM_003749 | 77.93 | 1.14 | 81.62 | 4.69 |
| CCNG2 | NM_004354 | 104.08 | 3.72 | 57.35 | 3.1 |
| MAPK11 | NM_002751 | 73.03 | 0.74 | 66.71 | 1.75 |
| MAPK9 | NM_002752 | 116.55 | 8.62 | 135.83 | 19.4 |
| CD81 | NM_004356 | 70.77 | 5.33 | 104.04 | 12.28 |
| MAPK13 | NM_002754 | 48.67 | 4.18 | 57.14 | 4.62 |
| MAP2K1 | NM_002755 | 71.74 | 3.94 | 70.79 | 3.25 |
| CDC34 | NM_004359 | 89.49 | 2.58 | 122.28 | 25.93 |
| JAK3 | NM_000215 | 108.69 | 3.55 | 98.5 | 0.71 |
| TOP2A | NM_001067 | 114.89 | 1.79 | 146.5 | 16.95 |
| CDKN2D | NM_079421 | 67.06 | 36.59 | 116.54 | 13.73 |
| TOP2B | NM_001068 | 97.36 | 6.62 | 72.31 | 5.47 |
| NF1 | NM_000267 | 103.62 | 3.64 | 124.89 | 10.82 |
| NF2 | NM_000268 | 102.89 | 8.53 | 135.01 | 27.45 |
| CENPJ | NM_018451 | 101.46 | 6.66 | 108.31 | 15.33 |
| CDKN2C | NM_078626 | 99.31 | 3.8 | 114.05 | 4.47 |
| NUSAP1 | NM_018454 | 115.17 | 12.41 | 81.11 | 3.49 |
| DCC | NM_005215 | 60.05 | 2.32 | 47.15 | 1.23 |
| DTX1 | NM_004416 | 108.4 | 11.41 | 113.28 | 0.81 |
| TLK1 | NM_012290 | 96.83 | 6.66 | 102.61 | 10.23 |
| MAP4K3 | NM_003618 | 119.69 | 5.81 | 80.05 | 3.04 |
| TLE1 | NM_005077 | 119.11 | 2.96 | 119.75 | 3.54 |
| OSBPL2 | NM_144498 | 103.89 | 3.48 | 101.66 | 4.49 |
| MAPK15 | NM_139021 | 83.71 | 0.03 | 96.21 | 1.13 |
| CCNB1 | NM_031966 | 105.2 | 0.66 | 100.8 | 3.59 |
| DVL1 | NM_004421 | 97.5 | 7.74 | 73.04 | 0.8 |
| DVL2 | NM_004422 | 72.75 | 9.75 | 87.21 | 8.12 |
| DVL3 | NM_004423 | 133.96 | 1.29 | 104.84 | 6.9 |
| E2F1 | NM_005225 | 85.69 | 6.67 | 45.73 | 11.95 |
| PTK7 | NM_002821 | 80.79 | 4.36 | 88.5 | 7.42 |
| EGFR | NM_005228 | 87.82 | 5.87 | 82.47 | 6.51 |
| ELK1 | NM_005229 | 82.44 | 0.28 | 102.5 | 10.03 |
| STAT5B | NM_012448 | 99.66 | 1.54 | 88.31 | 3.71 |
| FGFR3 | NM_000142 | 93.44 | 1.46 | 84.36 | 7.84 |
| RALY | NM_016732 | 96.39 | 5.8 | 107.81 | 3.65 |
| GNAS | NM_016592 | 100.13 | 5.16 | 89.81 | 2.57 |
| PIGT | NM_015937 | 75.23 | 1.89 | 71.47 | 2.18 |
| EPHA1 | NM_005232 | 96.87 | 1.67 | 89.96 | 8.03 |
| MAPK7 | NM_139034 | 97.52 | 8.08 | 89.94 | 3.54 |
| EPHA2 | NM_004431 | 77.52 | 1.61 | 74.15 | 2.45 |
| CDC42BPB | NM_006035 | 85.85 | 0.64 | 86.19 | 1.5 |
| TBK1 | NM_013254 | 105.12 | 11.62 | 113.3 | 5.31 |
| ERBB4 | NM_005235 | 90.5 | 3.27 | 91.95 | 8.74 |
| SPATA2 | NM_006038 | 76.87 | 0.81 | 84.01 | 3.5 |
| MANBAL | NM_022077 | 89.75 | 3.19 | 93.61 | 0.82 |
| SGK3 | NM_013257 | 87.19 | 12.16 | 106.67 | 9.85 |
| ETS1 | NM_005238 | 111.93 | 0.84 | 118.23 | 1.87 |
| CBFA2T2 | NM_005093 | 123.13 | 11.89 | 117.9 | 9.7 |
| EPHA4 | NM_004438 | 108.44 | 10.3 | 102.75 | 0.99 |
| POLD1 | NM_002691 | 111.37 | 6.16 | 104.18 | 10.56 |
| CSNK1A1 | NM_001892 | 82 | 3.05 | 86.46 | 4.58 |
| CSNK1D | NM_001893 | 72.72 | 4.27 | 73.55 | 10.43 |
| CSNK1E | NM_001894 | 80.04 | 4.16 | 63.49 | 4.84 |
| CSNK2A1 | NM_001895 | 104.44 | 0.03 | 78.19 | 3.05 |
| CSNK2A2 | NM_001896 | 86.97 | 1.15 | 74.78 | 1.11 |
| EPHA7 | NM_004440 | 95.87 | 1.33 | 70.57 | 2.36 |
| HDAC6 | NM_006044 | 90.94 | 5.88 | 77.23 | 5.95 |
| EPHB1 | NM_004441 | 76.56 | 4.34 | 74.97 | 0.4 |
| EPHB3 | NM_004443 | 88.87 | 6.58 | 84.05 | 0.04 |
| RBM12 | NM_006047 | 119.94 | 2.42 | 124.3 | 16.77 |
| EPHB4 | NM_004444 | 92.02 | 8.84 | 117.23 | 2.27 |
| FER | NM_005246 | 113.89 | 1.71 | 109.62 | 12.47 |
| EPHB6 | NM_004445 | 91.66 | 1.42 | 90.35 | 4.57 |
| FGF3 | NM_005247 | 91.63 | 8.85 | 77.09 | 7.46 |
| FGR | NM_005248 | 81.13 | 3.76 | 57.13 | 11.28 |
| EPS8 | NM_004447 | 96.6 | 0.15 | 63.91 | 9.53 |
| ERBB2 | NM_004448 | 108.79 | 5.06 | 132.76 | 21.9 |
| ACVR2B | NM_001106 | 74.31 | 3.43 | 72.73 | 2.82 |
| C20orf14 | NM_012469 | 61.95 | 4.15 | 76.32 | 6.6 |
| PPGB | NM_000308 | 79.4 | 5.19 | 89.91 | 3.07 |
| C20orf172 | NM_024918 | 99.28 | 2.74 | 92.22 | 1.04 |
| NCOA6 | NM_014071 | 88.63 | 19.57 | 91.15 | 12.54 |
| FOS | NM_005252 | 111.99 | 6.6 | 89.46 | 0.93 |
| ZNF335 | NM_022095 | 109.28 | 9.36 | 83.12 | 4.31 |
| TXN2 | NM_012473 | 103.39 | 8.24 | 62.65 | 5.96 |
| RIPK4 | NM_020639 | 77.86 | 1.06 | 69.26 | 8.26 |
| POLS | NM_006999 | 93.41 | 9.19 | 105.25 | 5.68 |
| PTEN | NM_000314 | 108.3 | 2.38 | 76.35 | 7.94 |
| NR3C1 | NM_000176 | 93.77 | 5.65 | 76.43 | 0.13 |
| GSS | NM_000178 | 89.42 | 2.57 | 99.44 | 0.44 |
| ADRM1 | NM_007002 | 90.08 | 5.06 | 105.1 | 6.9 |
| PRKCE | NM_005400 | 101.46 | 2.3 | 85.29 | 0.57 |
| RALA | NM_005402 | 97.95 | 5.56 | 82.95 | 1.58 |
| PDGFRA | NM_006206 | 88.16 | 12.85 | 61 | 6.9 |
| RAGE | NM_014226 | 106.6 | 2.15 | 108.9 | 2.18 |
| ROCK1 | NM_005406 | 98.67 | 5.45 | 87.88 | 11.25 |
| RIPK1 | NM_003804 | 91.21 | 3.74 | 101.9 | 0.91 |
| HMMR | NM_012484 | 80.14 | 8.37 | 99.74 | 3.22 |
| RB1 | NM_000321 | 114.88 | 6.48 | 96.56 | 3.69 |
| RET | NM_000323 | 101.42 | 1.29 | 77.04 | 2.01 |
| RAB2 | NM_002865 | 110.22 | 12.48 | 111.16 | 14.67 |
| RAB3A | NM_002866 | 96.4 | 6.34 | 110 | 0.45 |
| GUCY2D | NM_000180 | 56.07 | 4.43 | 70.97 | 6.96 |
| ATR | NM_001184 | 82.2 | 2.96 | 63.46 | 9.5 |
| ALS2CR2 | NM_018571 | 119.7 | 0.36 | 57.31 | 29.32 |
| RRM2B | AL137348 | 96.69 | 7.87 | 93.9 | 11.79 |
| PCTK1 | NM_033018 | 101.28 | 0.14 | 109.6 | 8.33 |
| KIF25 | NM_030615 | 83.97 | 25.4 | 71.65 | 12.35 |
| GRAP2 | NM_004810 | 127.94 | 3.88 | 118.86 | 16.2 |
| RHEB | NM_005614 | 89.44 | 6.15 | 75.21 | 26.43 |
| PLK2 | NM_006622 | 111.25 | 5.5 | 114.41 | 3.4 |
| ITGB4BP | NM_181467 | 90.89 | 0.18 | 103.59 | 1.55 |
| ANAPC1 | NM_022662 | 73.74 | 2.25 | 77 | 2.14 |
| RASGRP2 | NM_005825 | 53.8 | 43.87 | 102.86 | 9.6 |
| DYRK2 | NM_006482 | 114.9 | 4.92 | 56.47 | 5.23 |
| LTK | NM_002344 | 70.99 | 5.47 | 70.92 | 5.96 |
| MAP2K3 | NM_145110 | 86.63 | 3.65 | 83.04 | 6.21 |
| DEPDC1 | NM_017779 | 118.58 | 0.56 | 100.88 | 0.76 |
| SFRS6 | NM_006275 | 127.11 | 4.14 | 106.97 | 5.13 |
| HDAC5 | NM_005474 | 76.29 | 0.35 | 62.21 | 1.77 |
| STAT3 | NM_139276 | 137.92 | 8.44 | 72.04 | 6.2 |
| CTNND1 | NM_001331 | 121.87 | 7.62 | 96.27 | 7.79 |
| DIRAS3 | NM_004675 | 77.59 | 3.61 | 74.27 | 0.09 |
| CTNND2 | NM_001332 | 74.6 | 11.95 | 91.49 | 30.04 |
| FRAT1 | NM_005479 | 96.47 | 8.23 | 95.13 | 6.64 |
| TPX2 | NM_012112 | 11.83 | 2.29 | 3.25 | 0.88 |
| CDC25B | NM_021874 | 115.83 | 3.73 | 98.52 | 1.93 |
| BRCA1 | NM_007296 | 85.38 | 1.71 | 55.52 | 3.06 |
| STAT4 | NM_003151 | 100.61 | 1.73 | 80.94 | 4.37 |
| CCRK | NM_012119 | 108.35 | 5.21 | 106.71 | 2.75 |
| LYN | NM_002350 | 88.4 | 4.69 | 103.72 | 6.74 |
| STAT5A | NM_003152 | 61.98 | 6.69 | 62.37 | 9.5 |
| STAT6 | NM_003153 | 101.31 | 3.77 | 99.86 | 4.38 |
| PMS1 | NM_000534 | 115.84 | 5.64 | 100.8 | 5.61 |
| CTSZ | NM_001336 | 89.48 | 2.55 | 52.54 | 3.34 |
| PMS2 | NM_000535 | 88.82 | 0.6 | 69.48 | 4.5 |
| CHD6 | NM_032221 | 94.57 | 9.47 | 78.8 | 6.57 |
| EGR2 | NM_000399 | 98.11 | 1.53 | 85.02 | 1.48 |
| ARFGEF2 | NM_006420 | 89.97 | 0.86 | 79.31 | 17.63 |
| C20orf45 | NM_016045 | 112.76 | 9.14 | 126.46 | 11.71 |
| TACSTD1 | NM_002354 | 79.79 | 0.06 | 47.16 | 12.55 |
| NEK4 | NM_003157 | 93.04 | 0.11 | 97.89 | 6.06 |
| TUBA8 | NM_018943 | 49.29 | 4.02 | 52.94 | 14.32 |
| WT1 | NM_024426 | 100.72 | 10.55 | 93.71 | 0.88 |
| C20orf43 | NM_016407 | 85.82 | 2.59 | 76.6 | 12.25 |
| ZGPAT | NM_181484 | 59.07 | 2.55 | 68.17 | 0.3 |
| FZR1 | NM_016263 | 105.2 | 1.99 | 101.96 | 2.14 |
| TGIF2 | NM_021809 | 121.16 | 3.1 | 70.71 | 1.18 |
| STK4 | NM_006282 | 81.54 | 5.56 | 77.11 | 0.59 |
| CCT7 | NM_006429 | 77.96 | 6.5 | 53.82 | 2.96 |
| SGK | NM_005627 | 118.16 | 9.13 | 104.85 | 9.19 |
| TESK1 | NM_006285 | 81.48 | 0.66 | 57.24 | 2.03 |
| WISP2 | NM_003881 | 81.17 | 3.05 | 82.44 | 1.83 |
| WISP1 | NM_003882 | 91.81 | 0.03 | 105.34 | 2.62 |
| HDAC3 | NM_003883 | 85.47 | 2.06 | 83.45 | 2.07 |
| PTK2B | NM_004103 | 88.83 | 10.9 | 85.21 | 0.76 |
| LEF1 | NM_016269 | 94.2 | 1.94 | 85.62 | 4.79 |
| NFKB2 | NM_002502 | 102.45 | 5.35 | 88.57 | 6.39 |
| AURKC | NM_003160 | 82.99 | 1.38 | 59.28 | 0.28 |
| NR3C2 | NM_000901 | 72.85 | 2.07 | 38.52 | 1.56 |
| RPS6KB1 | NM_003161 | 92.33 | 1.89 | 97.79 | 9.17 |
| KIF21A | AK000059 | 97.93 | 5.89 | 72.73 | 4.96 |
| IRAK1 | NM_001569 | 99.32 | 4.3 | 48.45 | 7.88 |
| TCF1 | NM_000545 | 92.52 | 2.62 | 84.99 | 7.28 |
| TP53 | NM_000546 | 122 | 7.85 | 121.55 | 6.08 |
| DAPK3 | NM_001348 | 80.82 | 6.07 | 76.87 | 2.1 |
| CCNB3 | NM_033031 | 106.75 | 2.9 | 74.66 | 8.31 |
| CCT4 | NM_006430 | 46.59 | 6.97 | 49.68 | 4.19 |
| CCT2 | NM_006431 | 47.28 | 0.87 | 50.57 | 1.18 |
| KIF13B | NM_015254 | 111.83 | 5.73 | 47.9 | 1.39 |
| SOS1 | NM_005633 | 92.84 | 0.75 | 91.85 | 7.65 |
| SGK2 | NM_016276 | 94.46 | 4.27 | 81.49 | 0.9 |
| FLT3 | NM_004119 | 108.01 | 7.92 | 97.66 | 3.17 |
| TTK | NM_003318 | 85.39 | 0.86 | 67.92 | 6.81 |
| BLK | NM_001715 | 50.24 | 0.64 | 51.94 | 6.8 |
| IRAK2 | NM_001570 | 102.63 | 4.55 | 93.7 | 4.13 |
| SYK | NM_003177 | 85.32 | 2.74 | 77.18 | 1.22 |
| BMP7 | NM_001719 | 100.09 | 6.45 | 107.86 | 1.19 |
| MATK | NM_002378 | 91.81 | 15.33 | 93.36 | 4.86 |
| YTHDF1 | NM_017798 | 108.13 | 9.81 | 108.75 | 1.53 |
| TSG101 | NM_006292 | 120.59 | 6.24 | 94.36 | 3.14 |
| TYRO3 | NM_006293 | 75.74 | 1.76 | 69.84 | 3.7 |
| SFRS10 | U61267 | 32.66 | 0.04 | 37.96 | 1.98 |
| EIF2AK3 | NM_004836 | 124.86 | 15.06 | 130.38 | 4.38 |
| CDCA3 | NM_031299 | 84.15 | 8.31 | 82.81 | 0.2 |
| VRK2 | NM_006296 | 92.46 | 1.63 | 97.45 | 2.9 |
| NFS1 | NM_021100 | 111.78 | 9.26 | 102.04 | 8.63 |
| MAP3K1 | AF042838 | 111.38 | 3.34 | 104.84 | 11.85 |
| COX4I2 | NM_032609 | 108.68 | 9.23 | 71.12 | 4.55 |
| GTSE1 | NM_016426 | 73.34 | 0.71 | 67.21 | 9.41 |
| TAOK3 | NM_016281 | 103.5 | 6.6 | 89.86 | 1.51 |
| AXIN1 | AF009674 | 105.14 | 13.91 | 85.3 | 12.62 |
| TOMM34 | NM_006809 | 86.27 | 8.8 | 104.6 | 6.05 |
| SMC4L1 | NM_005496 | 110.16 | 5.63 | 87.87 | 4.1 |
| VHL | NM_000551 | 122.25 | 2.53 | 86.21 | 5.6 |
| GDF5 | NM_000557 | 111.24 | 8.97 | 113.01 | 7.78E-03 |
| HDAC7A | NM_015401 | 70.35 | 3.31 | 42.23 | 8.76 |
| RASAL2 | NM_004841 | 93.23 | 7.1 | 84.33 | 0.47 |
| ID1 | NM_002165 | 85.78 | 2.12 | 69.83 | 2.48 |
| ROCK2 | NM_004850 | 106.29 | 3.33 | 110.23 | 12.56 |
| LIMK1 | NM_002314 | 99.2 | 5.7 | 105.17 | 3.06 |
| DTYMK | NM_012145 | 21.32 | 3.57 | 21.05 | 9.84 |
| BMX | NM_001721 | 95.64 | 8.36 | 95.74 | 13.02 |
| NRAS | NM_002524 | 103.19 | 11.01 | 70.51 | 5.75 |
| WNK3 | AJ409088 | 101.97 | 6.57 | 94.2 | 6.46 |
| PAK6 | NM_020168 | 83.01 | 18.94 | 84.23 | 9.54 |
| TXK | NM_003328 | 81.76 | 6.57 | 99.19 | 0.89 |
| NTRK1 | NM_002529 | 83.68 | 4.56 | 94.16 | 5.81 |
| TAF4 | NM_003185 | 83.44 | 6.43 | 85.54 | 9.73 |
| SPAG4 | NM_003116 | 103.66 | 1.49 | 84.52 | 27.21 |
| KIF23 | NM_004856 | 66.74 | 6.77 | 56.77 | 6.32 |
| TCFL5 | NM_006602 | 83.79 | 4.86 | 76.83 | 11.36 |
| MAP2K2 | NM_030662 | 104.48 | 7.14 | 109.26 | 16.77 |
| CDK5RAP1 | NM_016082 | 100.26 | 5.63 | 96.33 | 2.78 |
| SPAG5 | NM_006461 | 34.09 | 0.89 | 38.65 | 1.15 |
| RASD1 | NM_016084 | 101.22 | 2.6 | 121.78 | 4.05 |
| C20orf158 | NM_152302 | 68.58 | 2.31 | 62.57 | 1.51 |
| PGR | NM_000926 | 92.69 | 0.87 | 96.7 | 7.3 |
| MCC | NM_002387 | 117.94 | 17.11 | 105.9 | 6.67 |
| MCM3 | NM_002388 | 111.34 | 0.1 | 81.68 | 5.87 |
| DCK | NM_000788 | 106.28 | 2.12 | 97.82 | 14.88 |
| TDE1 | NM_006811 | 107.99 | 3.35 | 94.51 | 4.68 |
| PHF20 | NM_016436 | 85.33 | 4.02 | 64.95 | 1.14 |
| KIAA1804 | AJ311798 | 94.8 | 7.01 | 102.68 | 8.87 |
| TUBG2 | NM_016437 | 105.41 | 12.09 | 80.82 | 6.47 |
| MAP3K2 | NM_006609 | 91.93 | 5.76 | 87.85 | 7.8 |
| HDAC2 | NM_001527 | 98.59 | 5.59 | 82.42 | 1.74 |
| YSK4 | NM_025052 | 106.66 | 15.63 | 122 | 6.04 |
| NLK | NM_016231 | 107.39 | 4.1 | 100.57 | 2.93 |
| PDRG1 | NM_030815 | 115.24 | 8.69 | 95.16 | 5.92 |
| GRAP | NM_006613 | 63.67 | 3.6 | 54.08 | 18.17 |
| ANAPC5 | NM_016237 | 106.02 | 6.31 | 74.99 | 11.72 |
| NCOA3 | NM_181659 | 90.73 | 0.99 | 95.44 | 5.1 |
| CTNNBL1 | NM_030877 | 106.43 | 4.01 | 91.2 | 4.79 |
| TYK2 | NM_003331 | 71.86 | 0.24 | 80.18 | 2.67 |
| NTRK3 | NM_002530 | 119.18 | 11.82 | 104.15 | 1.51 |
| RGS19 | NM_005873 | 132.04 | 1.37 | 121.99 | 6.03 |
| EIF2C2 | NM_012154 | 121.25 | 13.29 | 117.01 | 1.01 |
| MDM2 | NM_002392 | 93.35 | 7.32 | 105.28 | 1.83 |
| STK38 | NM_007271 | 118.44 | 4.69 | 124.96 | 18.41 |
| PRKD3 | NM_005813 | 110.41 | 11.61 | 101.11 | 6.33 |
| RPS6KA6 | NM_014496 | 124.93 | 6.06 | 120.92 | 5.47 |
| HIF1A | NM_001530 | 109.35 | 1.56 | 67.1 | 11.42 |
| SRPK1 | NM_003137 | 121.55 | 8.78 | 112.02 | 2.45 |
| DYRK1A | NM_001396 | 102.78 | 1.95 | 105.11 | 1.02 |
| CTBP2 | NM_022802 | 89.59 | 4.94 | 112.89 | 3.14 |
| NOTCH2 | NM_024408 | 107.45 | 18.96 | 99.99 | 6.3 |
| TBP | NM_003194 | 84.43 | 4.79 | 73.19 | 0.56 |
| POLR2A | NM_000937 | 9.77 | 2.3 | 9.27 | 4.4 |
| POLR2B | NM_000938 | 56.4 | 0.29 | 56.35 | 1.39 |
| C20orf161 | NM_033421 | 80.7 | 2.53 | 78.63 | 2.34 |
| PKIG | NM_181804 | 109.52 | 0.57 | 122.35 | 15.17 |
| SGOL2 | NM_152524 | 80.94 | 2.61 | 91.67 | 12.09 |
| TMEPAI | NM_020182 | 99.75 | 0.26 | 91.6 | 9.69 |
| PAK4 | NM_005884 | 75.63 | 9.36 | 76.9 | 6.93 |
| TCEB1 | NM_005648 | 84.61 | 1.18 | 100.78 | 6.45 |
| UBE2L3 | NM_003347 | 81.27 | 7.88 | 91.25 | 8.32 |
| KIF11 | NM_004523 | 17.43 | 6.63 | 14.33 | 6.41 |
| PSENEN | NM_172341 | 77.43 | 1.48 | 89.74 | 4.07 |
| FUCA1 | NM_000147 | 99.95 | 1.83 | 104 | 4.42 |
| GAA | NM_000152 | 93.1 | 0.82 | 95.48 | 2.17 |
| GALK1 | NM_000154 | 83.71 | 0.26 | 51.79 | 9.08 |
| GALT | NM_000155 | 94.29 | 5.86 | 96.77 | 10.67 |
| GBA | NM_000157 | 67.99 | 2.98 | 67.68 | 6.85 |
| GBE1 | NM_000158 | 96.53 | 2.54 | 117.58 | 1.84 |
| GCDH | NM_000159 | 106.1 | 9.26 | 118.5 | 10.72 |
| COX6A1 | AF020589 | 75.7 | 2.52 | 62.03 | 10.25 |
| PCCB | NM_000532 | 99.85 | 3.15 | 97.08 | 8.67 |
| ZNF265 | AF065391 | 97.56 | 6.4 | 84.91 | 5.42 |
| SMPD1 | NM_000543 | 103.04 | 5.72 | 90.35 | 14.24 |
| PRO0650 | AF090941 | 107.58 | 8.85 | 117.25 | 0.66 |
| TAP2 | NM_000544 | 85.14 | 2.07 | 97.57 | 7.24 |
| IBTK | AF235049 | 98.92 | 17.44 | 107.32 | 8.77 |
| TSC2 | NM_000548 | 111.23 | 3.33 | 117.3 | 10.14 |
| MTHFS | AF249277 | 103.15 | 0.13 | 97.23 | 4.76 |
| WRN | NM_000553 | 118.82 | 5.51 | 109.01 | 0.57 |
| THOC2 | AF441770 | 84.53 | 6.25 | 80.52 | 0.67 |
| GSTM1 | NM_000561 | 105.34 | 2.81 | 102.32 | 6.28 |
| TXNDC4 | AJ344330 | 101.98 | 6.51 | 112.32 | 0.24 |
| DAF | NM_000574 | 91.07 | 2.21 | 99.19 | 0.53 |
| GLA | NM_000169 | 112.16 | 5.49 | 108.54 | 7.32 |
| GPI | NM_000175 | 109.08 | 3.55 | 106.92 | 0.56 |
| GUSB | NM_000181 | 110.51 | 4.04 | 128.14 | 0.19 |
| HADHA | NM_000182 | 128.14 | 1.02 | 125.7 | 15.29 |
| HK2 | NM_000189 | 95.96 | 14.36 | 100.6 | 9.63 |
| HMBS | NM_000190 | 111.53 | 0.81 | 110.78 | 8.19 |
| HPS1 | NM_000195 | 139.66 | 4.05 | 130.68 | 2.57 |
| ITGA6 | NM_000210 | 100.92 | 5.42 | 89.78 | 1.71 |
| C14orf125 | AK000343 | 112.36 | 5.48 | 69.79 | 15.95 |
| TAP1 | NM_000593 | 93.66 | 1.83 | 35.87 | 4.5 |
| FNBP3 | AK000592 | 47.06 | 5.17 | 50.49 | 3.6 |
| IL10RB | NM_000628 | 110.99 | 5.3 | 104.19 | 0.75 |
| FAM62B | AK001181 | 111.76 | 2.07 | 101.55 | 7.39 |
| IFNAR1 | NM_000629 | 97.92 | 3.56 | 103.96 | 1.41 |
| FLJ12649 | AK022463 | 87.59 | 4.72 | 80.29 | 2.61 |
| SOD2 | NM_000636 | 89.78 | 5.92 | 83.95 | 3.79 |
| UNC84A | AK022469 | 97.47 | 7.56 | 105.58 | 5.24 |
| ACY1 | NM_000666 | 100.72 | 6.26 | 62.88 | 2.98 |
| ZDHHC5 | AK023130 | 99.76 | 6.7 | 96.32 | 4.61 |
| ALDH1B1 | NM_000692 | 63.23 | 4.92 | 81.15 | 2.01 |
| ATP11A | AK024264 | 94.43 | 7.28 | 93.54 | 2.82 |
| ALDH3B1 | NM_000694 | 107.47 | 0.71 | 119.59 | 2.48 |
| MAK10 | AK025266 | 105.93 | 7.08 | 112.17 | 0.05 |
| ALDH9A1 | NM_000696 | 76.75 | 8.41 | 93.4 | 0.06 |
| LAMA3 | NM_000227 | 108.36 | 4.8 | 115.71 | 7.84 |
| SGCB | NM_000232 | 125.39 | 7.79 | 103.95 | 2.79 |
| MEN1 | NM_000244 | 74.31 | 3.86 | 57.67 | 12.08 |
| MET | NM_000245 | 101.86 | 11.29 | 88.37 | 10.93 |
| MICA | NM_000247 | 105.91 | 4.69 | 107.36 | 6.83 |
| MTM1 | NM_000252 | 86.68 | 2.67 | 72.74 | 3.59 |
| MTR | NM_000254 | 112.35 | 13.71 | 75.27 | 9.6 |
| RAB6IP1 | AK025499 | 99.31 | 2.6 | 94.18 | 3.74 |
| ATP1A1 | NM_000701 | 84.37 | 2.63 | 72.24 | 2.05 |
| C17orf27 | AK025676 | 112.01 | 5.96 | 90.11 | 4.83 |
| BCKDHA | NM_000709 | 99.33 | 13.14 | 100.77 | 4.14 |
| NUP160 | AK026236 | 78.14 | 2.17 | 84.92 | 1.29 |
| BLVRA | NM_000712 | 84.62 | 16.22 | 75.9 | 4.36 |
| KIAA1815 | AK026962 | 47.9 | 1.93 | 52.71 | 1.7 |
| CACNB3 | NM_000725 | 109.05 | 15.21 | 87.24 | 0.48 |
| KIAA1794 | AK027564 | 101.08 | 13.28 | 92.75 | 8.28 |
| CHRNA5 | NM_000745 | 76.27 | 4.29 | 75.78 | 0.2 |
| KIAA1715 | AK056532 | 75.21 | 1.05 | 85.88 | 1.41 |
| COMT | NM_000754 | 85.73 | 0.69 | 103.81 | 3.59 |
| TIMP2 | AK057217 | 94.59 | 0.81 | 108.2 | 1.6 |
| CYP51A1 | NM_000786 | 95.72 | 3.1 | 81.6 | 1.49 |
| MUT | NM_000255 | 60.75 | 8.69 | 73.67 | 1.39 |
| MYO5A | NM_000259 | 74.37 | 2.36 | 70.94 | 10.83 |
| NAGLU | NM_000263 | 57.74 | 1.59 | 58.09 | 2.51 |
| NME1 | NM_000269 | 95.01 | 3.46 | 80.62 | 4.12 |
| OAT | NM_000274 | 81.63 | 2.93 | 56.5 | 1.36 |
| PCBD1 | NM_000281 | 53.22 | 3.09 | 61.37 | 5.61 |
| PDHA1 | NM_000284 | 101.75 | 0.43 | 104.85 | 12.96 |
| PEPD | NM_000285 | 80.65 | 5.19 | 41.01 | 8.48 |
| TMEM76 | AK057293 | 83.96 | 3.39 | 94.12 | 1.92 |
| FOLR1 | NM_000802 | 99.89 | 5.56 | 108.23 | 7.25 |
| SLC39A10 | AK074996 | 83.43 | 2.08 | 76.96 | 6.42 |
| GGCX | NM_000821 | 81.8 | 10.2 | 58.88 | 12.68 |
| ARID2 | AK075385 | 104.14 | 7.97 | 130.72 | 5.62 |
| GSTM3 | NM_000849 | 29.48 | 12.53 | 24.52 | 10.59 |
| PSMB7 | AK090539 | 75.81 | 7.96 | 77.54 | 2.09 |
| GSTM4 | NM_000850 | 100.35 | 7.33 | 89.09 | 6.62 |
| KIRREL | AK090554 | 69.68 | 4.02 | 74.12 | 4.09 |
| GSTM5 | NM_000851 | 110.37 | 4.64 | 96.56 | 2.55 |
| KIAA0241 | AK090619 | 100.72 | 11.26 | 110.51 | 7.84 |
| GSTT2 | NM_000854 | 92.31 | 2 | 112.09 | 0.13 |
|  | AK098160 | 101.81 | 7.87 | 118 | 7.03 |
| IGF2R | NM_000876 | 95.02 | 15.34 | 103.82 | 6.01 |
| HEATR1 | AK098212 | 82.64 | 3.84 | 84.85 | 3.9 |
| LTA4H | NM_000895 | 101.14 | 2.78 | 91.38 | 4.03 |
| PEX12 | NM_000286 | 109.87 | 4.82 | 109.14 | 1 |
| PFKM | NM_000289 | 54.06 | 27.85 | 83.39 | 3.73 |
| PGK1 | NM_000291 | 88.24 | 9.65 | 190.05 | 98.42 |
| PHKA2 | NM_000292 | 87.14 | 7.53 | 52.86 | 13.81 |
| PHKB | NM_000293 | 88.76 | 1.25 | 99.11 | 5.95 |
| PLOD1 | NM_000302 | 79.18 | 2.72 | 75.83 | 9.56 |
| PMM2 | NM_000303 | 99.63 | 1.44 | 98.3 | 5.66 |
| PMP22 | NM_000304 | 76.99 | 2.7 | 76.84 | 0.41 |
| NBPF14 | AL117237 | 86.3 | 0.7 | 60.91 | 1.82 |
| MAOB | NM_000898 | 98.16 | 2.31 | 99.64 | 6.16 |
| KIAA1219 | AL132998 | 102.82 | 11.38 | 70.48 | 8 |
| NQO1 | NM_000903 | 64.16 | 1.28 | 65.8 | 2.58 |
| XPO6 | AY026388 | 95.96 | 5.97 | 103.29 | 2.8 |
| NQO2 | NM_000904 | 54.03 | 0.13 | 63.4 | 1.32 |
| KIAA0152 | BC000371 | 100.27 | 3.01 | 99.85 | 2.14 |
| NPR1 | NM_000906 | 72.14 | 17.64 | 85.93 | 1.33 |
| PRNPIP | BC001072 | 82.87 | 7.35 | 75.35 | 3.46 |
| P4HA1 | NM_000917 | 99.63 | 9.36 | 100.22 | 12.66 |
| KIAA0494 | BC002525 | 101.13 | 6.95 | 106.89 | 3.63 |
| P4HB | NM_000918 | 74.93 | 1.26 | 96.97 | 1.21 |
| TOMM70A | BC003633 | 77.77 | 8.73 | 101.97 | 5.26 |
| PAM | NM_000919 | 92.67 | 2.67 | 105.9 | 12.58 |
| PLXNB2 | BC004542 | 89 | 12.43 | 107.99 | 2.46 |
| PC | NM_000920 | 72.26 | 5.89 | 70.7 | 0.69 |
| PON2 | NM_000305 | 84.84 | 4.18 | 71.71 | 15.54 |
| PPOX | NM_000309 | 123.61 | 7.53 | 118.97 | 7.84 |
| PRNP | NM_000311 | 114.42 | 8.6 | 101.19 | 7.41 |
| PTS | NM_000317 | 99.33 | 2.36 | 85.09 | 0.71 |
| PXMP3 | NM_000318 | 71.81 | 1.26 | 85.7 | 2.77 |
| QDPR | NM_000320 | 102.21 | 2.52 | 97.31 | 0.2 |
| SLC12A3 | NM_000339 | 87.08 | 3.3 | 90.53 | 1.16 |
| MGC3413 | BC004969 | 94.42 | 1.75 | 98.73 | 0.88 |
| PDHB | NM_000925 | 110.47 | 2.08 | 112.68 | 8.01 |
| hCAP-D3 | BC011408 | 85.75 | 6.49 | 85.09 | 3.46 |
| PLCB3 | NM_000932 | 83.56 | 8.36 | 80.01 | 4.73 |
| ZNF262 | BC012093 | 82.28 | 5.03 | 67.92 | 7.94 |
| POR | NM_000941 | 96.64 | 7.07 | 70.28 | 8.5 |
| RSPRY1 | BC013173 | 73.75 | 3.73 | 102.26 | 2.93 |
| PPIB | NM_000942 | 67.6 | 1.68 | 77.59 | 3.22 |
| PREPL | BC013193 | 100.34 | 5.2 | 125.94 | 4.24 |
| PPIC | NM_000943 | 99.43 | 10.3 | 87.88 | 0.22 |
| GAPVD1 | BC021119 | 107.28 | 2.68 | 125.81 | 1.75 |
| PRRG1 | NM_000950 | 79.75 | 0.57 | 70.07 | 8.37 |
| KIAA0368 | BC021127 | 59.89 | 16.26 | 78.13 | 0.39 |
| PTGIR | NM_000960 | 67.5 | 5.43 | 78.55 | 1.92 |
| STS | NM_000351 | 94.42 | 10.98 | 105.41 | 3.01 |
| TPMT | NM_000367 | 93.84 | 8.52 | 81.01 | 7.64 |
| TSC1 | NM_000368 | 95.62 | 4.06 | 74.71 | 9.27 |
| UMPS | NM_000373 | 83.01 | 4.36 | 96.22 | 0.86 |
| UROD | NM_000374 | 103.14 | 2.58 | 98.22 | 3.63 |
| UROS | NM_000375 | 57.89 | 8.22 | 57.69 | 3.29 |
| MID1 | NM_000381 | 102.5 | 6.51 | 85.96 | 4.27 |
| BLMH | NM_000386 | 92.48 | 11.88 | 94.8 | 12.02 |
| C4orf9 | BC026035 | 80.73 | 3.39 | 80.6 | 5.21 |
| PTGS2 | NM_000963 | 94.14 | 1.15 | 90.9 | 0.13 |
| SLC7A6 | BC028216 | 108.34 | 2.98 | 120.46 | 2.92 |
| RARG | NM_000966 | 78.99 | 3.92 | 97.06 | 11.82 |
| KIAA0256 | BC033001 | 119.1 | 6.76 | 105.93 | 12.34 |
| SLC12A2 | NM_001046 | 93.62 | 8.92 | 97.9 | 2.17 |
| GDA | BC033310 | 84.31 | 1.8 | 103.4 | 1.09 |
| SRD5A1 | NM_001047 | 88.32 | 16.45 | 90.9 | 8.33 |
| NXPH4 | BC036679 | 85.67 | 18.41 | 82.84 | 9.92 |
| SSTR5 | NM_001053 | 98.35 | 1.61 | 114.34 | 0.74 |
| PTENP1 | BC038293 | 111.29 | 9.85 | 102.19 | 1.21 |
| SULT1A2 | NM_001054 | 76.71 | 11.51 | 76.58 | 4.45 |
| C17orf49 | BC040036 | 75.29 | 4.79 | 82.69 | 0.08 |
| SULT1A1 | NM_001055 | 106.39 | 0.53 | 90.25 | 6.31 |
| OAZ1 | D78361 | 84.7 | 16.37 | 80.92 | 2.74 |
| TKT | NM_001064 | 59.29 | 2.04 | 44.89 | 0.74 |
| CHM | NM_000390 | 97.61 | 0.53 | 106.77 | 5.6 |
| ABCC2 | NM_000392 | 84.35 | 4.11 | 99.06 | 4.42 |
| CYB5R3 | NM_000398 | 90.74 | 10.31 | 82.7 | 1.46 |
| G6PD | NM_000402 | 85.14 | 8.67 | 96.61 | 0.75 |
| GALE | NM_000403 | 87.82 | 10.42 | 96.23 | 2.48 |
| GLB1 | NM_000404 | 88.86 | 7.39 | 90.93 | 11.53 |
| GPD2 | NM_000408 | 56.21 | 5.18 | 68.99 | 7.36 |
| HLCS | NM_000411 | 103.66 | 2.91 | 118.41 | 3.82 |
| ACADVL | NM_000018 | 57.61 | 2.61 | 58.3 | 10.45 |
| TNFRSF1A | NM_001065 | 87.19 | 1.91 | 92.26 | 5.89 |
| ACAT1 | NM_000019 | 121.15 | 17.24 | 120.9 | 3.95 |
| TUBB2A | NM_001069 | 113 | 14.77 | 123.24 | 0.17 |
| ADSL | NM_000026 | 73.37 | 3.19 | 91.13 | 4.09 |
| PLOD3 | NM_001084 | 59.78 | 8.2 | 50.26 | 6.53 |
| ALDOA | NM_000034 | 91.67 | 2.17 | 84.28 | 1.43 |
| ACO2 | NM_001098 | 102.16 | 0.77 | 99.43 | 5.46 |
| FAS | NM_000043 | 93.57 | 10.45 | 103.46 | 5.41 |
| ACTN3 | NM_001104 | 116.53 | 12.86 | 123.6 | 7.01 |
| ASL | NM_000048 | 95.65 | 7.57 | 79.33 | 3.21 |
| ADAM10 | NM_001110 | 99 | 8.48 | 96.21 | 10.39 |
| ASS | NM_000050 | 73.21 | 9.97 | 67.36 | 7.4 |
| TETRAN | NM_001120 | 88.48 | 3.4 | 91.74 | 1.38 |
| BCKDHB | NM_000056 | 88.33 | 3.35 | 106.23 | 11.08 |
| ADM | NM_001124 | 83.25 | 8.05 | 46.31 | 13.34 |
| HSD17B4 | NM_000414 | 84.33 | 0.21 | 83.3 | 0.67 |
| IFNGR1 | NM_000416 | 104.7 | 1.47 | 90.4 | 3.28 |
| IL4R | NM_000418 | 113.78 | 9.55 | 124.85 | 0.02 |
| L1CAM | NM_000425 | 110.93 | 5.81 | 109.4 | 0.46 |
| NEU1 | NM_000434 | 101.99 | 3.99 | 109.75 | 4.19 |
| OXCT1 | NM_000436 | 85.7 | 11.52 | 67.59 | 3.46 |
| SOD1 | NM_000454 | 74.2 | 4.44 | 91.15 | 7.79 |
| BTD | NM_000060 | 71.38 | 2.56 | 79.22 | 1.15 |
| ADSS | NM_001126 | 103.12 | 10.65 | 92.34 | 0.73 |
| CLN3 | NM_000086 | 103.48 | 10.07 | 96.59 | 6.63 |
| AP1B1 | NM_001127 | 92.81 | 6.99 | 41.86 | 1.69 |
| CPOX | NM_000097 | 105.27 | 13.78 | 108.29 | 9.67 |
| AP1G1 | NM_001128 | 97.47 | 8.46 | 115.03 | 7.42 |
| CST3 | NM_000099 | 107.16 | 1.53 | 115.22 | 6.58 |
| AMFR | NM_001144 | 97.28 | 2.48 | 58.66 | 2.62 |
| DDB2 | NM_000107 | 80.49 | 10.18 | 93.75 | 4.13 |
| ARHGAP5 | NM_001173 | 106.49 | 4.71 | 103.04 | 2.32 |
| DLD | NM_000108 | 117.3 | 0.52 | 132.63 | 0.89 |
| ARL1 | NM_001177 | 75.38 | 12.04 | 91.35 | 9.47 |
| TOR1A | NM_000113 | 93.92 | 11.26 | 109.88 | 12.46 |
| ALDH7A1 | NM_001182 | 100.22 | 5.63 | 103.34 | 9.53 |
| SUOX | NM_000456 | 86.22 | 3.91 | 71.25 | 3.94 |
| AK1 | NM_000476 | 82.95 | 4.72 | 89.05 | 11.49 |
| APP | NM_000484 | 76.9 | 7.61 | 80.75 | 5.72 |
| APRT | NM_000485 | 111.99 | 2.88 | 92 | 9.11 |
| GALNS | NM_000512 | 86.87 | 5.3 | 102.15 | 4.68 |
| HEXA | NM_000520 | 103.8 | 14.82 | 118.52 | 9.84 |
| HEXB | NM_000521 | 88.04 | 6.05 | 89.34 | 13.75 |
| HOXA13 | NM_000522 | 78.29 | 2.57 | 84.32 | 4.35 |
| TAZ | NM_000116 | 61.52 | 0.46 | 38.55 | 0.12 |
| ATP6AP1 | NM_001183 | 64.48 | 3.24 | 73.43 | 1.96 |
| EMD | NM_000117 | 102.64 | 0.05 | 101.44 | 6.41 |
| BCAT2 | NM_001190 | 119.52 | 2.01 | 123.35 | 16.26 |
| EPHX1 | NM_000120 | 119.49 | 0.95 | 121.57 | 3.35 |
| C1QBP | NM_001212 | 95.37 | 10.42 | 112.14 | 0.85 |
| EPOR | NM_000121 | 105.74 | 4.58 | 109.17 | 1.4 |
| CA9 | NM_001216 | 99.04 | 5.62 | 110.5 | 7.89 |
| ERCC3 | NM_000122 | 90.76 | 2.7 | 92.29 | 1.88 |
| CA11 | NM_001217 | 89.63 | 2.09 | 98.04 | 4.53 |
| ERCC6 | NM_000124 | 61.54 | 2.52 | 65.34 | 6.09 |
| CASP4 | NM_001225 | 110.66 | 10.9 | 101.59 | 18.85 |
| EXT1 | NM_000127 | 106.59 | 11.08 | 120.82 | 13.53 |
| CASP6 | NM_001226 | 103.77 | 10.33 | 108.24 | 8.03 |
| FH | NM_000143 | 82.64 | 0.25 | 95.34 | 0.08 |
| CASP9 | NM_001229 | 91.37 | 8.88 | 100.41 | 0.45 |
| CAV2 | NM_001233 | 107.4 | 4.46 | 110.72 | 1.23 |
| GOT2 | NM_002080 | 108.35 | 3.04 | 121.13 | 1.65 |
| SERPINH1 | NM_001235 | 108.08 | 3.33 | 119.86 | 0.61 |
| GPX4 | NM_002085 | 118.49 | 1.87 | 122.09 | 1.66 |
| CD68 | NM_001251 | 82.1 | 12.09 | 57.97 | 10.33 |
| GRN | NM_002087 | 109.31 | 8.11 | 112.14 | 0.28 |
| CHD1 | NM_001270 | 92.61 | 7.89 | 88.04 | 0.4 |
| GYS1 | NM_002103 | 103.92 | 3.96 | 110.39 | 1.95 |
| CHD2 | NM_001271 | 101.4 | 2.96 | 94.92 | 9.02 |
| HARS | NM_002109 | 118.61 | 0.66 | 75.38 | 2.68 |
| CHKA | NM_001277 | 62.95 | 6.57 | 72.59 | 0.51 |
| HD | NM_002111 | 97.44 | 5.1 | 96.12 | 0.02 |
| AP1S1 | NM_001283 | 88.11 | 8.03 | 78.16 | 9.59 |
| HLA-A | NM_002116 | 103.09 | 5.35 | 97.48 | 15.47 |
| ATP5F1 | NM_001688 | 69.83 | 1.97 | 70.57 | 18.67 |
| PIGF | NM_002643 | 116.82 | 3.22 | 99.09 | 3.95 |
| ATP5G3 | NM_001689 | 119.61 | 6.83 | 112.63 | 11.59 |
| PIK3C2B | NM_002646 | 92.27 | 6.32 | 57.98 | 13.49 |
| ATP6V1A | NM_001690 | 95.85 | 3.93 | 99.48 | 1.18 |
| PIK4CB | NM_002651 | 93.24 | 1.01 | 111.95 | 7.96 |
| ATP6V1B2 | NM_001693 | 96.33 | 7.61 | 33.4 | 13.85 |
| PLAUR | NM_002659 | 110.7 | 3.51 | 99.09 | 9.43 |
| ATP6V1C1 | NM_001695 | 75.14 | 0.03 | 79.98 | 6.66 |
| PLP2 | NM_002668 | 94.86 | 1.27 | 117 | 13.56 |
| ATP6V1E1 | NM_001696 | 70.52 | 7.97 | 71.65 | 4.91 |
| PLS1 | NM_002670 | 90.63 | 2.74 | 98.07 | 1.67 |
| ATP5O | NM_001697 | 93.46 | 3.41 | 121.93 | 2.92 |
| PLXNB1 | NM_002673 | 105.55 | 5.45 | 86.29 | 1.19 |
| CLIC1 | NM_001288 | 107.25 | 7.28 | 99.2 | 6.96 |
| HLA-DOA | NM_002119 | 90.58 | 8.96 | 103.22 | 5.76 |
| CLNS1A | NM_001293 | 104.01 | 6.69 | 104.85 | 1.11 |
| HMOX2 | NM_002134 | 97.19 | 3.6 | 94.28 | 2.82 |
| COX10 | NM_001303 | 121.77 | 6.26 | 118.63 | 0.39 |
| NR4A1 | NM_002135 | 108.31 | 8.97 | 98.07 | 8.7 |
| CPD | NM_001304 | 115.93 | 7.51 | 113.57 | 3.67 |
| HPD | NM_002150 | 107.57 | 2.26 | 110.95 | 0.42 |
| CLDN4 | NM_001305 | 91.11 | 9.08 | 95.06 | 0.51 |
| IDH2 | NM_002168 | 108.66 | 0.49 | 95.82 | 7.88 |
| CLDN7 | NM_001307 | 87.27 | 7.87 | 90.72 | 11.8 |
| IGHMBP2 | NM_002180 | 91.87 | 6.71 | 75.72 | 1.99 |
| CSNK2B | NM_001320 | 87.43 | 4.32 | 78.62 | 6.19 |
| IL6ST | NM_002184 | 107.41 | 7.02 | 124.06 | 1.2 |
| CXADR | NM_001338 | 106.85 | 3.18 | 130.1 | 1.73 |
| INPP1 | NM_002194 | 103.69 | 2.86 | 106.79 | 1.62 |
| AUH | NM_001698 | 76.04 | 0.86 | 60.75 | 8.56 |
| POLR2C | NM_002694 | 60.64 | 1.23 | 55.76 | 1.33 |
| BF | NM_001710 | 112.32 | 3.55 | 102.73 | 10.87 |
| PPAT | NM_002703 | 95.2 | 0.47 | 85.63 | 2.71 |
| C1S | NM_001734 | 136.05 | 1.65 | 129.48 | 16.51 |
| PPM1B | NM_002706 | 88.84 | 2.59 | 101.8 | 0.73 |
| CAMLG | NM_001745 | 87.16 | 1.41 | 79.94 | 1.63 |
| PPM1G | NM_002707 | 128.33 | 6.24 | 109.68 | 0.94 |
| CANX | NM_001746 | 105.25 | 0.34 | 82.54 | 0.25 |
| PPP1CB | NM_002709 | 47.21 | 3.95 | 52.26 | 1.19 |
| CAPN2 | NM_001748 | 90.78 | 7.9 | 89.15 | 8.49 |
| PPP1CC | NM_002710 | 78.87 | 3.67 | 100.7 | 8.73 |
| CARS | NM_001751 | 102.96 | 0.83 | 117.64 | 8.69 |
| PPP2R2A | NM_002717 | 103.28 | 5 | 111.59 | 7.2 |
| CAT | NM_001752 | 108.24 | 0.27 | 124.95 | 5.94 |
| PPP2R5C | NM_002719 | 91.55 | 3.34 | 104.59 | 0.67 |
| DAB2 | NM_001343 | 111.68 | 6.26 | 115.09 | 2.06 |
| ACO1 | NM_002197 | 79.17 | 2.4 | 88.41 | 0.57 |
| DAD1 | NM_001344 | 113.75 | 7.6 | 122.53 | 5.97 |
| ITGAV | NM_002210 | 57.54 | 1.82 | 46.2 | 6.58 |
| DHX15 | NM_001358 | 77.76 | 0.43 | 58.87 | 8.68 |
| ITGB5 | NM_002213 | 66.95 | 1 | 81.88 | 8.71 |
| DECR1 | NM_001359 | 83.55 | 5.54 | 82.57 | 12 |
| IVD | NM_002225 | 120.4 | 9.65 | 118.1 | 13.64 |
| DHCR7 | NM_001360 | 103.49 | 3.17 | 112.63 | 10.81 |
| LAMP2 | NM_002294 | 102.51 | 4.33 | 115.47 | 10.8 |
| DKC1 | NM_001363 | 90.94 | 6.48 | 93.77 | 1.73 |
| LBR | NM_002296 | 76.62 | 3.24 | 50.85 | 16.34 |
| DNASE2 | NM_001375 | 96.66 | 6.35 | 99.52 | 10.38 |
| LDHB | NM_002300 | 110.28 | 3.76 | 121.61 | 3.89 |
| CAV1 | NM_001753 | 106.46 | 3.44 | 96.7 | 12.36 |
| PPP4C | NM_002720 | 135.69 | 2.36 | 135.36 | 9.89 |
| CD9 | NM_001769 | 63.79 | 0.83 | 83.34 | 4.97 |
| PPP6C | NM_002721 | 109.22 | 7.75 | 99.93 | 9.1 |
| CD33 | NM_001772 | 91.2 | 0.23 | 105.93 | 5.35 |
| PREP | NM_002726 | 116.28 | 2.81 | 132.05 | 5.18 |
| CD47 | NM_001777 | 76.21 | 4.71 | 62.13 | 2.35 |
| PRKAR1A | NM_002734 | 86.9 | 2.33 | 85.51 | 7.01 |
| CD58 | NM_001779 | 84.68 | 3.16 | 63.43 | 11.42 |
| PRPS1 | NM_002764 | 96.68 | 3.56 | 93.12 | 3.52 |
| CD63 | NM_001780 | 100.91 | 13.32 | 94.17 | 8.38 |
| PRPS2 | NM_002765 | 84.93 | 10.35 | 52.23 | 3.43 |
| CTSC | NM_001814 | 73.75 | 6.39 | 98.08 | 2.65 |
| PRPSAP1 | NM_002766 | 113.23 | 3.79 | 126.92 | 5.65 |
| DOCK1 | NM_001380 | 93.26 | 1.49 | 94.12 | 4.2 |
| LIFR | NM_002310 | 91.69 | 10.25 | 86.67 | 2.17 |
| DPH2 | NM_001384 | 105.68 | 12.22 | 107.45 | 2.15 |
| LOX | NM_002317 | 115.04 | 6.3 | 113.34 | 1.47 |
| DPYSL2 | NM_001386 | 109.85 | 4.81 | 101.27 | 4.99 |
| LOXL2 | NM_002318 | 91.58 | 2.71 | 100.24 | 7.56 |
| ECE1 | NM_001397 | 107.8 | 1.82 | 102.61 | 7.9 |
| LRCH4 | NM_002319 | 111.31 | 0.94 | 90.41 | 0.73 |
| EIF4G2 | NM_001418 | 108.69 | 4.28 | 116.8 | 3.31 |
| LRP3 | NM_002333 | 97.94 | 5.66 | 76.57 | 4.85 |
| EMP2 | NM_001424 | 101.74 | 2.02 | 102.54 | 4.11 |
| LRP5 | NM_002335 | 100.99 | 0.13 | 117.99 | 7.19 |
| EMP3 | NM_001425 | 86.12 | 2.59 | 106.06 | 2.12 |
| LRP6 | NM_002336 | 130.72 | 3.43 | 125.8 | 2.61 |
| EPB41L2 | NM_001431 | 67.21 | 3.96 | 87.76 | 7.09 |
| LRPAP1 | NM_002337 | 91 | 7.56 | 102.56 | 6.73 |
| CEACAM4 | NM_001817 | 87.96 | 4.53 | 96.92 | 3.04 |
| PRPSAP2 | NM_002767 | 110.63 | 1.74 | 116.87 | 6.41 |
| CHML | NM_001821 | 111.84 | 0.75 | 120.43 | 5.2 |
| PCOLN3 | NM_002768 | 94.33 | 2.14 | 100.61 | 3.98 |
| CHN1 | NM_001822 | 59.48 | 4.12 | 45.58 | 8.16 |
| HTRA1 | NM_002775 | 95.93 | 2.76 | 95.11 | 3.12 |
| CKB | NM_001823 | 104.45 | 4.77 | 109.34 | 12.17 |
| PSMA2 | NM_002787 | 74.81 | 0.52 | 83.43 | 5.68 |
| CLU | NM_001831 | 86 | 7.92 | 77.24 | 3.41 |
| PSMA3 | NM_002788 | 88.73 | 5.7 | 83.21 | 0.84 |
| SLC31A1 | NM_001859 | 50.26 | 6.77 | 55.88 | 14.08 |
| PSMA4 | NM_002789 | 83.11 | 0.41 | 91.49 | 12.44 |
| COX4I1 | NM_001861 | 104.89 | 14.64 | 113.47 | 2.93 |
| PSMA5 | NM_002790 | 116.39 | 2.28 | 125.46 | 3.64 |
| COX6B1 | NM_001863 | 117.88 | 2.66 | 123.43 | 9.78 |
| PSMB1 | NM_002793 | 67.77 | 7.9 | 51.4 | 29.33 |
| EXTL2 | NM_001439 | 82.36 | 1.3 | 74.04 | 0.71 |
| LSS | NM_002340 | 90.33 | 3.16 | 75.97 | 9.48 |
| EXTL3 | NM_001440 | 100.89 | 4.47 | 83.01 | 0.49 |
| LTBR | NM_002342 | 97.23 | 4.11 | 43.04 | 6.55 |
| FLNA | NM_001456 | 80.87 | 2.22 | 85.78 | 3.3 |
| LY6E | NM_002346 | 119.28 | 11.53 | 102.32 | 0.36 |
| FLNB | NM_001457 | 47.95 | 3.72 | 62.98 | 4.39 |
| M6PR | NM_002355 | 103.16 | 5.93 | 95.63 | 5.84 |
| GDI1 | NM_001493 | 62.27 | 10.53 | 58.2 | 4.92 |
| MARCKS | NM_002356 | 61.61 | 19.55 | 78.71 | 6.14 |
| GDI2 | NM_001494 | 103.51 | 5.64 | 92.27 | 24.1 |
| MAD2L1 | NM_002358 | 54.06 | 0.18 | 43.92 | 2.78 |
| GMDS | NM_001500 | 80.65 | 7.38 | 102.45 | 2.98 |
| MAZ | NM_002383 | 77.25 | 2.31 | 95.79 | 3.69 |
| GSTA4 | NM_001512 | 83.63 | 7.16 | 101.6 | 4.51 |
| SLC3A2 | NM_002394 | 92.98 | 2.36 | 96.88 | 10.85 |
| COX7A2 | NM_001865 | 86.95 | 6.19 | 96.49 | 2.5 |
| PSMB2 | NM_002794 | 72.85 | 1.26 | 75.24 | 3.81 |
| COX7B | NM_001866 | 93.68 | 2 | 70.69 | 1.76 |
| PSMB3 | NM_002795 | 72.93 | 4.09 | 70.8 | 1.25 |
| CPM | NM_001874 | 120.59 | 2.27 | 129.08 | 3.95 |
| PSMB4 | NM_002796 | 78.49 | 0.12 | 69.66 | 4.8 |
| CPS1 | NM_001875 | 84.4 | 3.25 | 87.97 | 6.4 |
| PSMB5 | NM_002797 | 81.31 | 2.83 | 79.06 | 5.81 |
| CPT1A | NM_001876 | 87.53 | 1.52 | 98.12 | 1.77 |
| PSMB6 | NM_002798 | 69.02 | 3.55 | 77.22 | 2.98 |
| CRYZ | NM_001889 | 84.33 | 11.33 | 108.15 | 4.54 |
| PSMB7 | NM_002799 | 47.53 | 5.75 | 55.73 | 8.75 |
| CTNNB1 | NM_001904 | 95.43 | 2.68 | 115.31 | 10.06 |
| PSMB10 | NM_002801 | 89.79 | 5.93 | 102.36 | 5.17 |
| CTPS | NM_001905 | 103.22 | 1.41 | 118.71 | 6.37 |
| PSMD2 | NM_002808 | 55.81 | 1.3 | 56.16 | 3.13 |
| GTF2H2 | NM_001515 | 100.19 | 1.29 | 71.22 | 13.01 |
| ME1 | NM_002395 | 104.22 | 9.45 | 76.39 | 14.49 |
| GTF2H3 | NM_001516 | 106.73 | 8.05 | 105.48 | 4.42 |
| ME2 | NM_002396 | 109.46 | 3.88 | 89.95 | 11.28 |
| GTF3C1 | NM_001520 | 83.42 | 10.49 | 78.2 | 0.17 |
| MEST | NM_002402 | 82.18 | 4.9 | 95.59 | 7.3 |
| MR1 | NM_001531 | 83.47 | 1.06 | 94.95 | 16.22 |
| MGAT5 | NM_002410 | 77.44 | 2.66 | 75.85 | 1.23 |
| HRMT1L1 | NM_001535 | 90.1 | 14.57 | 86.89 | 3.24 |
| MGST2 | NM_002413 | 74.91 | 3.95 | 83.24 | 0.53 |
| HRMT1L2 | NM_001536 | 99.53 | 0.48 | 104.17 | 10.69 |
| CD99 | NM_002414 | 50.22 | 10.95 | 45.49 | 4.9 |
| HSPB1 | NM_001540 | 55.1 | 6.32 | 62.22 | 0.8 |
| MMP15 | NM_002428 | 90.72 | 7.76 | 82.64 | 12.41 |
| CTSL | NM_001912 | 78.66 | 3.59 | 90.2 | 6.28 |
| PSMD7 | NM_002811 | 42.86 | 4.93 | 35.58 | 2.45 |
| CYC1 | NM_001916 | 109.78 | 9.32 | 97.97 | 13.47 |
| PSMD8 | NM_002812 | 21.37 | 1.11 | 9.19 | 3.04 |
| DBT | NM_001918 | 44.46 | 5.52 | 45.7 | 1.31 |
| QSCN6 | NM_002826 | 101.7 | 11.07 | 100 | 10.1 |
| DCI | NM_001919 | 85.58 | 1.43 | 92.69 | 7.24 |
| PTPN11 | NM_002834 | 95 | 2.59 | 107.69 | 3.11 |
| DCTD | NM_001921 | 100.68 | 1.36 | 118.6 | 4.21 |
| PEX19 | NM_002857 | 91.88 | 0.51 | 91.52 | 3.94 |
| DHPS | NM_001930 | 106.55 | 0.25 | 89.32 | 6.62 |
| ABCD3 | NM_002858 | 95.96 | 1.09 | 76.2 | 7.43 |
| DLAT | NM_001931 | 85.65 | 3.45 | 110.21 | 3.31 |
| ALDH18A1 | NM_002860 | 49.05 | 6 | 66.8 | 3.16 |
| NDST1 | NM_001543 | 87.13 | 4.3 | 88.37 | 1.32 |
| MNAT1 | NM_002431 | 96.63 | 1.64 | 93.67 | 2.09 |
| ICT1 | NM_001545 | 111.35 | 0.67 | 124.15 | 0.87 |
| MPI | NM_002435 | 111.04 | 5.03 | 111.97 | 18.39 |
| IFRD1 | NM_001550 | 111.5 | 3.15 | 117.65 | 8.33 |
| MPV17 | NM_002437 | 114.94 | 9.68 | 126.74 | 17.99 |
| IGFBP7 | NM_001553 | 98.04 | 2.74 | 97.08 | 6.12 |
| MSN | NM_002444 | 88.68 | 8.2 | 67.12 | 8.42 |
| CYR61 | NM_001554 | 94.07 | 1.24 | 72.74 | 2.4 |
| MTAP | NM_002451 | 96.21 | 2.47 | 84.1 | 2.92 |
| IL13RA1 | NM_001560 | 114.14 | 5.08 | 121.66 | 5.57 |
| MTX1 | NM_002455 | 93.4 | 4.02 | 100.22 | 4.36 |
| AARS | NM_001605 | 109.87 | 0.58 | 118.22 | 3.73 |
| MYD88 | NM_002468 | 90.49 | 2.82 | 102.02 | 9.48 |
| ABCA2 | NM_001606 | 96.63 | 0.83 | 88.94 | 2.56 |
| NUBP1 | NM_002484 | 108.46 | 7.26 | 113.4 | 5.12 |
| MPP3 | NM_001932 | 89.89 | 0.12 | 76.04 | 7.17 |
| PCYT2 | NM_002861 | 105.35 | 8.76 | 118.83 | 7.96 |
| DLST | NM_001933 | 77.58 | 11.23 | 89.62 | 1.68 |
| PYGL | NM_002863 | 107.81 | 0.23 | 92.63 | 2.15 |
| DR1 | NM_001938 | 105.98 | 3.13 | 112.39 | 16.83 |
| RAB5B | NM_002868 | 118.67 | 8.81 | 101.14 | 2.65 |
| DSG2 | NM_001943 | 38.38 | 37.89 | 83.17 | 6.05 |
| RAB6A | NM_002869 | 114.8 | 2.04 | 116 | 4.13 |
| EEF2 | NM_001961 | 81.7 | 0.2 | 101.8 | 3.88 |
| RAC2 | NM_002872 | 97.75 | 0.77 | 102.93 | 0.08 |
| EFNA5 | NM_001962 | 89.29 | 8.38 | 76.29 | 2.62 |
| RANGAP1 | NM_002883 | 77.24 | 3.54 | 85.79 | 1.03 |
| EHHADH | NM_001966 | 107.41 | 6.96 | 119.34 | 4.39 |
| RAP1GA1 | NM_002885 | 87.62 | 5.35 | 86.66 | 0.29 |
| ENO2 | NM_001975 | 108.28 | 6.62 | 119.11 | 4.25 |
| RAP2B | NM_002886 | 66.4 | 6.76 | 77.71 | 0.43 |
| ACADSB | NM_001609 | 75.63 | 0.1 | 56.96 | 5.22 |
| NDUFB3 | NM_002491 | 93.58 | 2.07 | 98.38 | 7.98 |
| ACP2 | NM_001610 | 60.62 | 8.14 | 65.73 | 4.45 |
| NDUFB5 | NM_002492 | 106.06 | 3.65 | 116.01 | 8.88 |
| AHR | NM_001621 | 112.63 | 5.74 | 92.34 | 12.62 |
| NDUFB6 | NM_002493 | 119.46 | 7.63 | 110.82 | 7.73 |
| AK2 | NM_001625 | 107.46 | 10.23 | 94.65 | 3.16 |
| NDUFC1 | NM_002494 | 120.03 | 5 | 61.75 | 23.12 |
| ALCAM | NM_001627 | 77.23 | 1.22 | 68.55 | 6.81 |
| NFX1 | NM_002504 | 109.8 | 0.55 | 92.87 | 4.53 |
| AKR1B1 | NM_001628 | 95.58 | 7.44 | 106.49 | 18.92 |
| NRD1 | NM_002525 | 81.93 | 6.36 | 91.78 | 11.37 |
| AMD1 | NM_001634 | 60.41 | 5.4 | 83.62 | 4.68 |
| NT5E | NM_002526 | 78.16 | 1.11 | 86.51 | 1.94 |
| APEH | NM_001640 | 105.48 | 5.55 | 138.29 | 14.26 |
| NUP88 | NM_002532 | 94.57 | 1.6 | 107 | 1.69 |
| ENO3 | NM_001976 | 100.13 | 1.73 | 95.53 | 0.05 |
| RARS | NM_002887 | 106.17 | 1.6 | 108.06 | 0.27 |
| EPB49 | NM_001978 | 85.39 | 1.64 | 67.59 | 1.14 |
| RDX | NM_002906 | 120.05 | 2.42 | 115.41 | 8.51 |
| ESD | NM_001984 | 97.95 | 2.78 | 114.22 | 0.4 |
| RENT1 | NM_002911 | 86.38 | 1.41 | 88.24 | 0.08 |
| EYA3 | NM_001990 | 110.7 | 11.04 | 137 | 19.15 |
| RFC3 | NM_002915 | 104.56 | 4.7 | 50.38 | 15.56 |
| FDPS | NM_002004 | 69.53 | 0.42 | 46.06 | 8.36 |
| RNASEH1 | NM_002936 | 127.85 | 3.75 | 111.71 | 0 |
| FKBP3 | NM_002013 | 114.85 | 8.92 | 109.2 | 8.74 |
| RNH1 | NM_002939 | 83.4 | 5.25 | 95.55 | 8.78 |
| FKBP4 | NM_002014 | 120.39 | 9.31 | 147.15 | 11.38 |
| ABCE1 | NM_002940 | 42.83 | 7.77 | 44.92 | 0.65 |
| FNTA | NM_002027 | 104.47 | 7.42 | 123.78 | 13.86 |
| RPN1 | NM_002950 | 74.33 | 4.6 | 88.65 | 2.01 |
| ARCN1 | NM_001655 | 27.19 | 0.11 | 24.26 | 1.07 |
| ODC1 | NM_002539 | 55.67 | 1.03 | 74.13 | 0.33 |
| TRIM23 | NM_001656 | 110.44 | 9.46 | 101.61 | 4.06 |
| OLR1 | NM_002543 | 97.82 | 6.63 | 81.82 | 3.58 |
| ARF1 | NM_001658 | 117.66 | 11.43 | 117.79 | 15.86 |
| ORC4L | NM_002552 | 146.71 | 11.07 | 150.88 | 12.51 |
| ARF3 | NM_001659 | 100.31 | 0.56 | 89.3 | 1.28 |
| FURIN | NM_002569 | 83.43 | 10.68 | 50.91 | 12.83 |
| ARL4D | NM_001661 | 88.45 | 3.76 | 83.52 | 1.17 |
| PRDX1 | NM_002574 | 99.26 | 0.68 | 110.73 | 6.25 |
| ARF5 | NM_001662 | 79.55 | 0.71 | 97.33 | 3.79 |
| PARN | NM_002582 | 108.96 | 11.1 | 126.32 | 12.21 |
| ARF6 | NM_001663 | 111.45 | 0.08 | 127.3 | 5.94 |
| PDGFA | NM_002607 | 102.71 | 4.16 | 112.51 | 8.44 |
| FNTB | NM_002028 | 86.59 | 8.08 | 77.98 | 1.16 |
| SORT1 | NM_002959 | 119.1 | 2.44 | 127.26 | 3.58 |
| FVT1 | NM_002035 | 76.98 | 2.07 | 98.92 | 1.51 |
| S100A10 | NM_002966 | 89.71 | 6.26 | 88.31 | 4.72 |
| GAS1 | NM_002048 | 93.67 | 5.88 | 103.49 | 1.16 |
| SCP2 | NM_002979 | 90.2 | 2.46 | 100.32 | 6.82 |
| GBP1 | NM_002053 | 94.6 | 1.85 | 91.22 | 2.06 |
| SDHB | NM_003000 | 106.57 | 4.56 | 116.97 | 9.53 |
| GFPT1 | NM_002056 | 80.53 | 2.62 | 73.8 | 5.97 |
| SEC14L1 | NM_003003 | 93.26 | 0.89 | 103.44 | 3.23 |
| GCLM | NM_002061 | 77.97 | 7.68 | 89.61 | 7.59 |
| SLC1A4 | NM_003038 | 95.19 | 6.02 | 114.27 | 6.95 |
| GLRX | NM_002064 | 102.19 | 3.63 | 105.61 | 1.45 |
| SLC4A2 | NM_003040 | 87.15 | 2.9 | 97.81 | 9.08 |
| RHOG | NM_001665 | 84.59 | 0.97 | 105.59 | 10.57 |
| PEX13 | NM_002618 | 110.87 | 2.01 | 112.36 | 11.45 |
| ARL2 | NM_001667 | 110.19 | 14.91 | 91.65 | 0.88 |
| PFKL | NM_002626 | 107.59 | 2.34 | 99.03 | 52.99 |
| ASNS | NM_001673 | 63.27 | 1.71 | 64.34 | 0.42 |
| PFKP | NM_002627 | 68.49 | 3.56 | 81.17 | 3.3 |
| ATP1B1 | NM_001677 | 58.28 | 5.02 | 65.63 | 4.11 |
| PGD | NM_002631 | 57.71 | 1.01 | 66.53 | 7.6 |
| ATP1B3 | NM_001679 | 89.28 | 0.56 | 105.83 | 1.84 |
| PGM1 | NM_002633 | 107.12 | 0.34 | 99.4 | 5.28 |
| ATP2B1 | NM_001682 | 107.78 | 0.75 | 106.24 | 17.77 |
| PHKA1 | NM_002637 | 99.91 | 1.97 | 99.76 | 17.6 |
| ATP5J | NM_001685 | 116.04 | 0.08 | 128.31 | 9.67 |
| SERPINB8 | NM_002640 | 117.96 | 1.75 | 129.13 | 23.4 |
| ATP5D | NM_001687 | 105.38 | 0.93 | 86.53 | 12.36 |
| PIGA | NM_002641 | 96.72 | 0.25 | 108.76 | 0.43 |
| GLUL | NM_002065 | 94.1 | 0.34 | 96.94 | 12.8 |
| SLC7A1 | NM_003045 | 98.31 | 4.76 | 105.84 | 5.01 |
| GNAI1 | NM_002069 | 77.33 | 2.76 | 74.81 | 0.02 |
| SLC16A1 | NM_003051 | 92.84 | 5.81 | 97.96 | 3.42 |
| GNAI2 | NM_002070 | 102.58 | 5.57 | 105.95 | 14.84 |
| SLC22A5 | NM_003060 | 106.65 | 1.34 | 46.96 | 15.93 |
| GNAL | NM_002071 | 100.63 | 2.31 | 120.4 | 15.73 |
| SMARCA2 | NM_003070 | 85.88 | 3.9 | 99.39 | 7.74 |
| GNAQ | NM_002072 | 123.17 | 2.68 | 126.16 | 10.01 |
| SMARCA3 | NM_003071 | 111.51 | 9.44 | 117.33 | 22.61 |
| GNB1 | NM_002074 | 79.84 | 7.57 | 79.12 | 2.91 |
| SMARCA4 | NM_003072 | 98.88 | 4.21 | 100.89 | 20.97 |
| GNS | NM_002076 | 105.47 | 5.47 | 119.17 | 6.04 |
| SOAT1 | NM_003101 | 83.21 | 0.7 | 113.3 | 6.77 |
| GOT1 | NM_002079 | 94.15 | 4.54 | 99.82 | 13.12 |
| SORD | NM_003104 | 95.07 | 7.63 | 89.71 | 8.62 |
| RTEL1 | NM_032957 | 93.04 | 3.14 | 92.02 | 3.63 |
| B4GALT5 | NM_004776 | 95.32 | 2.61 | 98.99 | 6.4 |
| AURKA | NM_003600 | 15.2 | 0.23 | 3.2 | 1.01 |
| EPB41L1 | NM_012156 | 110.43 | 3.84 | 126.94 | 0.52 |
| TCEA2 | NM_003195 | 71.97 | 5.68 | 82.34 | 0.15 |
| SBK1 | XM_370948 | 72.14 | 2.69 | 72.44 | 5.14 |
| LOC340156 | AK122581 | 61.65 | 3.78 | 78.28 | 3.61 |
| PKMYT1 | NM_004203 | 77.92 | 3.72 | 80.68 | 2.34 |
| C9orf96 | BC036504 | 63.7 | 6.33 | 82.09 | 1.08 |
| EMILIN3 | NM_052846 | 75.74 | 3.03 | 92.66 | 5.69 |
| EVI5L | NM_145245 | 78.9 | 3.35 | 75.31 | 4.64 |
| ADCK2 | NM_052853 | 93.58 | 13.11 | 86.53 | 3.85 |
| MARVELD3 | NM_052858 | 79.04 | 7.71 | 91.59 | 2.93 |
| AKT3 | NM_005465 | 70.58 | 15.2 | 108.23 | 3.79 |
| SRC | NM_005417 | 68.98 | 9.87 | 81.25 | 0.1 |
| TNNI3K | NM_015978 | 60.1 | 6.51 | 51.74 | 1.18 |
| HCK | NM_002110 | 77.67 | 11.01 | 79.3 | 11.61 |
| PDIK1L | NM_152835 | 75.24 | 2.66 | 91.49 | 9.52 |
| CAMK2A | NM_015981 | 59.35 | 9.35 | 74.58 | 0.43 |
| TIE1 | NM_005424 | 70.07 | 1.74 | 79.34 | 4.23 |
| PRKCE | NM_005400 | 61.41 | 9.8 | 62.22 | 2.2 |
| YSK4 | NM_025052 | 66.67 | 3.15 | 63.85 | 6.09 |
| RNASEL | NM_021133 | 73.9 | 6.37 | 82.49 | 5.02 |
| RAB32 | NM_006834 | 68.88 | 2.41 | 89.92 | 9.24 |
| RPS6KA2 | NM_021135 | 77.49 | 5.79 | 100.75 | 8.17 |
| FLT3 | NM_004119 | 84.41 | 2.17 | 101.1 | 6.52 |
| IRAK2 | NM_001570 | 104.84 | 4.59 | 89.63 | 3.2 |
| GPR125 | BC035645 | 96.11 | 13.71 | 117.44 | 8.02 |
| ULK2 | NM_014683 | 60.85 | 2.16 | 71 | 13.28 |
| PTGIS | NM_000961 | 87.28 | 5.84 | 98.69 | 5.76 |
| RIPK5 | AB007941 | 82.02 | 4.77 | 101.96 | 0.39 |
| LBP | NM_004139 | 74.06 | 3.13 | 114.36 | 1.74 |
| GUCY2F | NM_001522 | 91.11 | 2.41 | 115.66 | 12.27 |
| CDKL4 | XM_293029 | 83.55 | 4.11 | 94.35 | 4.68 |
| OPRL1 | NM_000913 | 99.9 | 8.33 | 119.97 | 2.41 |
| COL20A1 | NM_020882 | 75.47 | 4.09 | 81.1 | 5.69 |
| MYH7B | AB040945 | 75.33 | 6.29 | 49.5 | 8.18 |
| NTSR1 | NM_002531 | 110.3 | 14 | 127.77 | 3.58 |
| APEG1 | NM_005876 | 98.21 | 2.65 | 105.19 | 1.36 |
| RAPGEF3 | NM_006105 | 86.65 | 5.77 | 87.06 | 8.79 |
| GSG2 | NM_031965 | 73.48 | 13.64 | 78.5 | 5.43 |
| ACVR1C | NM_145259 | 94.44 | 1.17 | 88.09 | 9.26 |
| FLJ25006 | NM_144610 | 88.08 | 7.7 | 87.72 | 0.78 |
| NEK3 | NM_002498 | 77.37 | 4.96 | 78.23 | 10.15 |
| MYO3B | NM_138995 | 73.39 | 0.8 | 81.63 | 12.26 |
| SMAF1 | NM_174906 | 63.85 | 2.37 | 57.29 | 0.21 |
| EPHA1 | NM_005232 | 73.89 | 13.43 | 77.9 | 10.63 |
| EPHA3 | NM_005233 | 90.94 | 5.9 | 90.96 | 4.21 |
| ERBB4 | NM_005235 | 78.69 | 3.79 | 79.33 | 0.8 |
| TSSK1 | NM_032028 | 64.19 | 6.03 | 68.88 | 0.59 |
| MAP3K8 | NM_005204 | 83.09 | 7.85 | 96.52 | 9.59 |
| MAP4K4 | AK000380 | 65.44 | 1.67 | 50.27 | 1.82 |
| MLKL | NM_152649 | 106.62 | 1.6 | 78.53 | 2.08 |
| STK38L | NM_015000 | 86.44 | 1.99 | 115.62 | 10.29 |
| CSF1R | NM_005211 | 90.92 | 3.46 | 103.85 | 10.39 |
| STK40 | NM_032017 | 69.2 | 0.05 | 89.21 | 5.88 |
| CAMKK1 | NM_172207 | 98.82 | 13.92 | 107.39 | 12.76 |
| ZHX3 | NM_015035 | 108.86 | 2.53 | 101.37 | 1.75 |
| PI4K2B | NM_018323 | 78.21 | 5.45 | 94.75 | 12.51 |
| FGR | NM_005248 | 95.4 | 10.51 | 86.2 | 0.74 |
| PDZK3 | NM_178140 | 119.71 | 10.59 | 124.48 | 7.51 |
| TSSK4 | NM_174944 | 76.49 | 6.47 | 100.37 | 7.73 |
| STK31 | NM_031414 | 110.7 | 16.15 | 128.99 | 6.69 |
| EIF2AK1 | NM_014413 | 79.13 | 5.45 | 96.32 | 13.42 |
| TRIB2 | NM_021643 | 67.72 | 2.04 | 90.27 | 6.67 |
| HERC2 | NM_004667 | 96.55 | 9.69 | 114.87 | 8.31 |
| NEK8 | NM_178170 | 91.85 | 10.94 | 101.01 | 3.08 |
| PXMP4 | NM_007238 | 91.41 | 0.85 | 85.92 | 1.06 |
| RPS6KA6 | NM_014496 | 69.21 | 11.63 | 90.8 | 5.07 |
| RAB38 | NM_022337 | 72.34 | 1.57 | 86.04 | 5.01 |
| LCK | NM_005356 | 71.57 | 8.81 | 67.64 | 1.46 |
| GRK4 | NM_005307 | 63.15 | 3.65 | 45.52 | 0.42 |
| GRK5 | NM_005308 | 55.14 | 2.6 | 72.49 | 1.29 |
| FLT1 | NM_002019 | 103.7 | 2.1 | 109.15 | 3.58 |
| MAP3K13 | NM_004721 | 52.6 | 4.67 | 59.69 | 5.2 |
| PASK | NM_015148 | 114.38 | 5.97 | 101.87 | 2.93 |
| DCAMKL1 | NM_004734 | 104.67 | 9.52 | 111.21 | 5.43 |
| C20orf100 | NM_032883 | 78.4 | 6.86 | 80.86 | 4.56 |
| COMMD7 | NM_053041 | 112.82 | 9.36 | 98.69 | 4.8 |
| FRK | NM_002031 | 94.57 | 0.41 | 106.49 | 1.57 |
| STYK1 | NM_018423 | 81.66 | 4.67 | 95.91 | 3.81 |
| PRKAG2 | NM_016203 | 88.1 | 7.74 | 84.84 | 7.08 |
| STC1 | NM_003155 | 89.59 | 7.14 | 91.4 | 0.31 |
| NEK4 | NM_003157 | 42.04 | 2.92 | 46.77 | 5.81 |
| AURKC | NM_003160 | 70.92 | 14.08 | 75.3 | 2.18 |
| DYRK2 | NM_006482 | 83.98 | 15.07 | 75.92 | 6.7 |
| MAP3K5 | NM_005923 | 78.37 | 16.04 | 90.01 | 4.38 |
| PDGFRB | NM_002609 | 72.42 | 13.2 | 88.95 | 13.18 |
| TGFBR2 | NM_003242 | 79.47 | 2.63 | 93.55 | 9.81 |
| KSR2 | NM_173598 | 93.91 | 7.15 | 103.67 | 11.28 |
| PDK2 | NM_002611 | 61.08 | 2.75 | 80.32 | 5.97 |
| CDC14B | NM_033331 | 74.5 | 5.13 | 91.13 | 0.86 |
| MAPK10 | NM_138982 | 98 | 2.9 | 123.96 | 9.32 |
| PIK3CG | NM_002649 | 74.85 | 3.6 | 70.05 | 1.77 |
| FGFR2 | NM_022972 | 77.95 | 7.1 | 91.88 | 0.54 |
| PRKAR2B | NM_002736 | 72.84 | 5.8 | 80.37 | 7.76 |
| DCAMKL3 | AB051552 | 98.43 | 0.51 | 95.19 | 3.26 |
| ATM | NM_000051 | 86.67 | 5.71 | 83.66 | 10.42 |
| TSSK2 | NM_053006 | 94.98 | 8.08 | 92.4 | 4.47 |
| TXK | NM_003328 | 74.57 | 2.27 | 90.67 | 10.18 |
| EPHA6 | NM_173655 | 63.23 | 0.55 | 83.01 | 0.02 |
| MGC42105 | NM_153361 | 72.09 | 2.6 | 86.3 | 1.12 |
| PRKD1 | NM_002742 | 77.17 | 7.87 | 85.38 | 7.07 |
| MAPK4 | NM_002747 | 85.29 | 2.29 | 103.37 | 19.74 |
| BTK | NM_000061 | 53.59 | 0.08 | 58.52 | 4.73 |
| PAK7 | NM_020341 | 72.54 | 1.8 | 64.61 | 12.33 |
| MAPK13 | NM_002754 | 88.07 | 0.29 | 103.88 | 0.33 |
| EPHA10 | NM_173641 | 54.71 | 5.89 | 64.71 | 3.28 |
| MYLK | NM_053025 | 81.05 | 1.05 | 101.2 | 3.22 |
| PRKACG | NM_002732 | 93.43 | 8.89 | 101.06 | 0.06 |
| STK32B | NM_018401 | 51.5 | 0.37 | 63.61 | 2.54 |
| C20orf144 | NM_080825 | 108.47 | 6.29 | 106.34 | 2.11 |
| ADCK5 | NM_174922 | 67.93 | 7.09 | 79.2 | 10.32 |
| LOC400301 | XM_375150 | 76.16 | 2.15 | 75.32 | 3.02 |
| CDK6 | BC027989 | 79.84 | 3.33 | 86.8 | 0.66 |
| RIPK4 | NM_020639 | 45.4 | 5.04 | 40.68 | 0.43 |
| CAMK1 | NM_003656 | 60.06 | 0.49 | 82.64 | 6.42 |
| PRKCH | NM_006255 | 43.52 | 5.29 | 63.07 | 15.48 |
| PRKCQ | NM_006257 | 86.76 | 0.31 | 116.82 | 5.46 |
| PRKG1 | NM_006258 | 93.98 | 3.51 | 100.14 | 7.58 |
| PRKG2 | NM_006259 | 74.43 | 4.55 | 102.84 | 3.35 |
| STK35 | NM_080836 | 52.97 | 6.5 | 71.45 | 5.03 |
| PDGFRA | NM_006206 | 85.88 | 0.89 | 87.05 | 8.45 |
| GRK7 | NM_139209 | 78.75 | 0.06 | 92.55 | 5.25 |
| STK16 | NM_003691 | 97.57 | 4.43 | 99.79 | 11.12 |
| RET | NM_000323 | 75.22 | 12.36 | 77.94 | 1.93 |
| PHKG1 | NM_006213 | 55.99 | 7.29 | 55.73 | 5.12 |
| LOC91461 | BC007901 | 106.51 | 2.56 | 108.35 | 3.13 |
| GPR8 | NM_005286 | 90.9 | 2.65 | 74.79 | 1.11 |
| NRK | NM_198465 | 77.86 | 1.63 | 87.65 | 0.47 |
| HRH3 | NM_007232 | 74 | 5.44 | 75.82 | 2.01 |
| FLJ40852 | NM_173677 | 51.25 | 0.89 | 51.43 | 2.48 |
| MMP24 | NM_006690 | 116.57 | 9.73 | 107.21 | 3.68 |
| CHRNA4 | NM_000744 | 88.51 | 0.87 | 90.63 | 6.04 |
| KIAA1755 | AB051542 | 113.28 | 10.08 | 108.57 | 5.6 |
| NEK1 | AB067488 | 71.24 | 1.86 | 91.59 | 1.02 |
| LIMK2 | NM_005569 | 82.4 | 5.13 | 87.63 | 5.16 |
| ITK | NM_005546 | 73.91 | 2.08 | 108.12 | 7.65 |
| MUSK | NM_005592 | 79.72 | 7.86 | 80.99 | 5.82 |
| KDR | NM_002253 | 63.11 | 8.66 | 47.51 | 2.02 |
| MARK1 | NM_018650 | 77.22 | 1.16 | 74.84 | 14.99 |
| PAN3 | NM_175854 | 90.95 | 1.39 | 85.02 | 0.16 |
| NTRK2 | NM_006180 | 76.99 | 3.65 | 87.31 | 3.68 |
| DDR2 | NM_006182 | 57.52 | 3.8 | 74.9 | 6.19 |
| STK32A | NM_145001 | 75.37 | 3.14 | 62.1 | 4.02 |
| ACVR1B | NM_004302 | 71.31 | 1.48 | 76.38 | 9.19 |
| PRKAG3 | NM_017431 | 80.26 | 2.88 | 75.48 | 4.68 |
| MYO3A | NM_017433 | 59.63 | 6.11 | 76.5 | 3.2 |
| ALK | NM_004304 | 55.72 | 3.83 | 64.7 | 1.79 |
| LRRK1 | NM_024652 | 86.81 | 4.35 | 88.59 | 2.28 |
| NUAK1 | NM_014840 | 77.84 | 1.98 | 86.73 | 5.53 |
| CAMK4 | NM_001744 | 87.83 | 8.48 | 104.59 | 7.95 |
| MLCK | NM_182493 | 90.77 | 1.86 | 96.03 | 15.46 |
| EPHB2 | NM_017449 | 88.53 | 3.26 | 97.83 | 10.74 |
| PIK3C2G | NM_004570 | 94.21 | 8.82 | 110.31 | 9.39 |
| CDK3 | NM_001258 | 78.21 | 0.85 | 106.06 | 11.25 |
| NPR2 | NM_003995 | 108.74 | 8.75 | 107.09 | 6.16 |
| TTBK2 | NM_173500 | 78.62 | 8.21 | 84.3 | 4.91 |
| TESK2 | NM_007170 | 68.07 | 5.61 | 73.87 | 3.63 |
| CAMKK2 | NM_006549 | 70.03 | 3.11 | 70.27 | 4.46 |
| FGFR1 | NM_000604 | 63.26 | 4.32 | 84.67 | 6.76 |
| CDKL2 | NM_003948 | 61.44 | 0.94 | 77.35 | 1.77 |
| NEK6 | NM_014397 | 108.78 | 1.53 | 114.97 | 7.73 |
| TEC | NM_003215 | 60.99 | 3.74 | 71.2 | 4.24 |
| MAP4K1 | NM_007181 | 81.17 | 4.26 | 88.4 | 4.49 |
| IRAK3 | NM_007199 | 90.53 | 1.27 | 104.24 | 2.56 |
| PKD2L2 | NM_014386 | 101.06 | 7.92 | 105.2 | 4.47 |
| MAK | NM_005906 | 103.14 | 6.64 | 125.78 | 0.45 |
| ROR1 | NM_005012 | 79.06 | 7.93 | 80.14 | 5.94 |
| TSSK3 | NM_052841 | 83.63 | 0.3 | 92.67 | 6.22 |
| HIPK2 | NM_022740 | 56.3 | 5.4 | 61.14 | 0.08 |
| PPP1R16B | NM_015568 | 89.07 | 4.77 | 82.68 | 1.95 |
| PIK3CD | NM_005026 | 88.74 | 3.21 | 129.45 | 7.24 |
| C20orf117 | NM_199181 | 76.93 | 4.27 | 88 | 5.22 |
| PRKAR1B | NM_002735 | 103.95 | 4.11 | 116.12 | 15.63 |
| GM632 | NM_020713 | 88.47 | 4.41 | 71.06 | 1 |
| RNPC1 | NM_017495 | 118.37 | 0.47 | 117.86 | 2.15 |
| CDH4 | NM_001794 | 86.64 | 1.33 | 53.4 | 0.65 |
| LRRK2 | AL834529 | 82.47 | 9.73 | 76.35 | 2.17 |
|  | XM_292638 | 60.98 | 0.56 | 75.03 | 1.92 |
| LOC442075 | AK092352 | 113.75 | 2.12 | 122.37 | 4.26 |
| PRO0628 | AF090938 | 95.18 | 2.47 | 79.59 | 1.07 |
| FLJ30698 | XM_375602 | 68.62 | 4.37 | 77.91 | 0.64 |
| C20orf165 | NM_080608 | 80.19 | 7.58 | 92.47 | 9.79 |
| LOC200230 | AK090440 | 112.18 | 2.99 | 79.58 | 4.94 |
|  | AK128670 | 102.18 | 5.39 | 112.27 | 7.57 |
| LOC390975 | XM_372749 | 86.31 | 1.67 | 82.46 | 13.5 |
| C20orf160 | NM_080625 | 88.14 | 4.36 | 61.69 | 2.73 |
| JPH2 | NM_020433 | 110.08 | 1.12 | 94.98 | 7.88 |
| C20orf102 | NM_080607 | 90.83 | 0.83 | 86.91 | 2.21 |
| NUAK2 | NM_030952 | 67.59 | 0.83 | 69.17 | 2.97 |
| CAMK1G | NM_020439 | 69.25 | 6.99E-03 | 84.55 | 1.09 |
| IGF1R | NM_000875 | 71.8 | 2.19 | 84.97 | 7.71 |
| SGK3 | NM_013257 | 91.09 | 10.35 | 79.57 | 5.66 |
| STK39 | NM_013233 | 100.1 | 5.26 | 122.75 | 9.56 |
| C20orf121 | NM_024331 | 106.78 | 0.76 | 107.9 | 3.5 |
| ULK4 | NM_017886 | 86.18 | 4.12 | 95 | 7.31 |
| RPS6KA5 | NM_004755 | 63.81 | 2.76 | 71.29 | 5.62 |
| STK33 | NM_030906 | 89.39 | 1.26 | 77.47 | 2.21 |
| AKAP13 | NM_006738 | 87.67 | 3.21 | 93.29 | 6.71 |
| OXSM | NM_017897 | 75.79 | 4.38 | 97.53 | 3.89 |
| HUNK | NM_014586 | 54.26 | 4.47 | 80.01 | 3.32 |
| KSR | U43586 | 102.33 | 9.61 | 93.14 | 10.16 |
| CDKL3 | NM_016508 | 94.33 | 10.06 | 94.26 | 3.16 |
| PSKH1 | NM_006742 | 89.76 | 9.37 | 102.42 | 1.26 |
| TNIK | AF172264 | 100.4 | 8.98 | 103.43 | 0.08 |
| ERN1 | NM_001433 | 84.15 | 1.48 | 83.56 | 1.32 |
| NAALADL1 | NM_005468 | 69.93 | 6.7 | 80.05 | 0.87 |
| WEE2 | AK131218 | 95.22 | 5.9 | 66.77 | 6.29 |
| NFATC2 | NM_173091 | 86.3 | 8.39 | 94.86 | 7.09 |
| TNFRSF6B | NM_003823 | 105.74 | 4.84 | 79.91 | 8.22 |
| MAP3K15 | NM_001001671 | 92.04 | 8.59 | 91.25 | 10.56 |
| TCEB3C | NM_145653 | 75.51 | 3.11 | 63.15 | 15.29 |
| RIPK3 | NM_006871 | 56.4 | 4.95 | 74.71 | 5.18 |
| CDKL1 | NM_004196 | 90.93 | 10.98 | 94.09 | 1.21 |
| AMHR2 | NM_020547 | 105.35 | 2.77 | 95.51 | 14.32 |
| KIT | NM_000222 | 103.29 | 12.57 | 107.81 | 1.95 |
| ROS1 | NM_002944 | 61.96 | 2.2 | 60.85 | 9.47 |
| INSR | NM_000208 | 55.74 | 8.3 | 73.33 | 1.21 |
| ZAK | NM_016653 | 81.54 | 2.67 | 92.22 | 7.53 |
| ALS2CR7 | NM_139158 | 83.59 | 4.74 | 90.77 | 3.12 |
| DYRK3 | NM_003582 | 81.22 | 2.7 | 94.05 | 4.96 |
| PARD6B | AB044555 | 73.79 | 1.94 | 86.39 | 4.34 |
| BMPR1B | NM_001203 | 72.76 | 4.71 | 70.26 | 2.37 |
| EYA2 | NM_172113 | 116.24 | 4.59 | 85.27 | 4.96 |
| STK36 | NM_015690 | 76.57 | 5.89 | 65.23 | 3.25 |
| CAMK2G | NM_172171 | 93.56 | 3.61 | 105.14 | 7.61 |
| MAST4 | AB002301 | 67.83 | 7.69 | 74.48 | 5.72 |
| NEK11 | NM_024800 | 86.3 | 7.94 | 93.06 | 8.21 |
| ERBB3 | NM_001982 | 45.13 | 2.11 | 53.54 | 2.99 |
| TRPM6 | NM_017662 | 82.71 | 0.13 | 106.49 | 5.85 |
| ACTR5 | NM_024855 | 80.08 | 4.62 | 85.8 | 1.32 |
| DAPK2 | NM_014326 | 82.92 | 1.51 | 92.29 | 2.57 |
| ROR2 | NM_004560 | 64.44 | 2.28 | 76.99 | 2.16 |
| ADRBK2 | NM_005160 | 89.53 | 12.76 | 97.52 | 2.84 |
| CAMK2D | NM_001221 | 114.49 | 4.42 | 108.39 | 3.63 |
| KIAA1804 | NM_032435 | 52.1 | 5.25 | 58.22 | 7.93 |
| STK19 | NM_032454 | 77.91 | 1.03 | 99.15 | 0.71 |
| L3MBTL | NM_015478 | 50.17 | 1.75 | 63.91 | 2.72 |
| CCRK | NM_012119 | 108.27 | 4.32 | 124.4 | 1.33 |
| BLK | NM_001715 | 76.21 | 4.22 | 67.32 | 0.85 |
| NRBP2 | NM_178564 | 85.27 | 2.62 | 90.85 | 11.65 |
| MAP3K1 | AF042838 | 73.19 | 4.69 | 89.35 | 11.32 |
| BMX | NM_001721 | 82.62 | 2.79 | 73.11 | 8.69 |
| BRDT | NM_001726 | 93.92 | 2.64 | 71.29 | 8.97 |
| SGK2 | NM_016276 | 118.93 | 10.53 | 115.72 | 0.79 |
| SYK | NM_003177 | 83.19 | 1.54 | 98.72 | 5.98 |
| NTRK3 | NM_002530 | 86.49 | 7.03 | 118.48 | 6.56 |
| ALPK2 | NM_052947 | 60.9 | 1.97 | 51.5 | 9.72 |
| NEK10 | NM_152534 | 91.6 | 12.72 | 88.51 | 5.87 |
| ZNF218 | NM_173485 | 81.08 | 4.08 | 93.44 | 2.09 |
| C20orf132 | NM_152503 | 100.2 | 3.62 | 108.02 | 5.35 |
| PAK6 | NM_020168 | 76.08 | 8.29 | 71.89 | 2.37 |
| PAK3 | NM_002578 | 86.9 | 2.97 | 99.32 | 1.35 |
| PKD2L1 | NM_016112 | 99.33 | 4.32 | 101.2 | 2.01 |
| PSKH2 | NM_033126 | 93.65 | 2.96 | 96.54 | 2.31 |
| MAP3K12 | NM_006301 | 94.05 | 7.51 | 124.2 | 16.05 |
| MYLK2 | NM_033118 | 78.18 | 2.9 | 80.17 | 3.37 |
| TEK | NM_000459 | 80 | 4.06 | 106.62 | 10.93 |
| MERTK | NM_006343 | 75.54 | 11.36 | 87.55 | 4.52 |
| RAPGEF4 | NM_007023 | 92.92 | 5.23 | 106.05 | 5.69 |
| GDAP1L1 | NM_024034 | 86.38 | 3.87 | 91.44 | 1.89 |
| EPHA4 | NM_004438 | 56.77 | 4.98 | 66.1 | 4.38 |
| EPHA5 | NM_004439 | 106.17 | 4.19 | 107.59 | 0.05 |
| RAGE | NM_014226 | 74.7 | 5.93 | 75.33 | 8.23 |
| CSNK2A1 | NM_001895 | 95.28 | 5.38 | 116.47 | 12.64 |
| TRIM24 | NM_003852 | 83.25 | 6.72 | 89.11 | 3.12 |
| EPHA7 | NM_004440 | 101.85 | 0.85 | 101.57 | 9.43 |
| EPHB1 | NM_004441 | 60.54 | 13.15 | 68.46 | 6.19 |
| CAMKV | NM_024046 | 118.53 | 18.19 | 119.61 | 0.89 |
| ACVR1 | NM_001105 | 86.56 | 7.22 | 85.08 | 3.09 |
| ACVR2B | NM_001106 | 76.95 | 13.75 | 87.99 | 3.79 |
| KALRN | NM_007064 | 89.19 | 6.86 | 101.93 | 2.35 |
| ATR | NM_001184 | 85.3 | 1.96 | 88.34 | 6.2 |
| TEX14 | NM_031272 | 70.79 | 3.01 | 90.32 | 6.44 |
| ACVR2A | NM_001616 | 71.37 | 2.63 | 73.69 | 8.49 |
| KIAA0889 | NM_152257 | 96.61 | 4.77 | 103.17 | 5.36 |
| ALPK1 | NM_025144 | 90.1 | 13.96 | 82.91 | 4.56 |
| TRIB1 | NM_025195 | 104.27 | 2.41 | 128.04 | 10.45 |
| GUCY2C | NM_004963 | 78.68 | 3.2 | 83.92 | 1.08 |
| MIP | NM_012064 | 64.33 | 2.08 | 63.05 | 4.7 |
| CDC2L1 | BC033069 | 60.23 | 2.36 | 66.36 | 3.72 |
| JAK2 | NM_004972 | 63.75 | 5.09 | 61.8 | 4.28 |
| BRSK1 | NM_032430 | 85.16 | 12.39 | 82.75 | 22.92 |
| TYK2 | NM_003331 | 105.96 | 3.31 | 102.84 | 0.89 |
| VRK3 | NM_016440 | 125.07 | 1.13 | 98.83 | 4.67 |
| PIM3 | NM_001001852 | 71.18 | 1.19 | 81.35 | 12.93 |
| NEK5 | BC063885 | 91.85 | 3.52 | 93.37 | 1.88 |
| PNCK | NM_198452 | 87.15 | 3.95 | 109.41 | 1 |
| WNK2 | NM_006648 | 82.75 | 10.22 | 83.16 | 5.6 |
| PAK4 | NM_005884 | 79.59 | 1.54 | 72.58 | 3.09 |
| PRKD2 | NM_016457 | 93.24 | 2.93 | 105 | 9.72 |
| ERN2 | NM_033266 | 79.02 | 3.2 | 92.82 | 6.94 |
| PRKAR2A | NM_004157 | 99.51 | 3.48 | 96.21 | 13.42 |
| NTRK1 | NM_002529 | 77.12 | 7.42 | 76.51 | 1.67 |
|  | hsa_miR_125a | 120.97 | 12.45 | 148.11 | 4.52 |
|  | hsa_miR_28 | 52.27 | 2.79 | 64.91 | 6.35 |
|  | hsa_miR_126star | 116.01 | 7.45 | 137.36 | 9.75 |
|  | hsa_miR_30a_5p | 123.55 | 7.22 | 138.48 | 15.47 |
|  | hsa_miR_126 | 65.87 | 9.24 | 67.5 | 2.73 |
|  | hsa_miR_31 | 92.12 | 11.01 | 118.8 | 6.42 |
|  | hsa_miR_127 | 53.99 | 3.6 | 55.82 | 2.12 |
|  | hsa_miR_32 | 73 | 2.5 | 81.02 | 3.62 |
|  | hsa_miR_134 | 25.05 | 0.6 | 30.14 | 2.91 |
|  | hsa_miR_33 | 88.92 | 7.29 | 64.33 | 22.54 |
|  | hsa_miR_136 | 103.08 | 12.6 | 124.04 | 1.32 |
|  | hsa_miR_95 | 115.75 | 3.15 | 120.58 | 10.26 |
|  | hsa_miR_149 | 100.66 | 4.09 | 100.99 | 8.83 |
|  | hsa_miR_96 | 70.63 | 7.3 | 82.35 | 11.51 |
|  | hsa_miR_20 | 90.75 | 2.96 | 103.2 | 0.58 |
|  | hsa_miR_30a_3p | 68.8 | 4.67 | 73.43 | 6.01 |
|  | hsa_miR_92 | 79.54 | 2.79 | 104.26 | 9.76 |
|  | hsa_miR_93 | 83.23 | 5.52 | 79.31 | 0.18 |
|  | hsa_miR_106a | 58.81 | 1.75 | 64.16 | 1.34 |
|  | hsa_miR_181b | 107.96 | 14.21 | 112.41 | 1.18 |
|  | hsa_miR_212 | 123.3 | 1.13 | 113.79 | 5.5 |
|  | hsa_miR_150 | 91.81 | 1.39 | 92.04 | 4.1 |
|  | hsa_miR_98 | 71.49 | 3.73 | 90.94 | 15.34 |
|  | hsa_miR_154star | 104.41 | 6.08 | 83.69 | 1.29 |
|  | hsa_miR_99a | 79.42 | 4.12 | 72.53 | 9.61 |
|  | hsa_miR_188 | 66.17 | 4.43 | 55.15 | 7.85 |
|  | hsa_miR_100 | 74.63 | 7.7 | 80.18 | 4.1 |
|  | hsa_miR_190 | 110.18 | 10.78 | 95.16 | 10.17 |
|  | hsa_miR_101 | 91.55 | 0.62 | 51.7 | 3.69 |
|  | hsa_miR_194 | 117.69 | 1.47 | 104.56 | 0.29 |
|  | hsa_miR_29b | 91.66 | 1.07 | 103.55 | 5.89 |
|  | hsa_miR_195 | 71.55 | 3.8 | 71.59 | 2.22 |
|  | hsa_miR_103 | 90.53 | 5.65 | 102.09 | 3.92 |
|  | hsa_miR_200c | 94.3 | 0.87 | 72.22 | 6.11 |
|  | hsa_miR_105 | 93.38 | 3.34 | 128.65 | 12.31 |
|  | hsa_miR_128b | 78.68 | 0.39 | 88.51 | 7.19 |
|  | hsa_miR_107 | 84.89 | 0.22 | 97.02 | 2.41 |
|  | hsa_miR_124a | 46.03 | 0.58 | 48.42 | 3.56 |
|  | hsa_miR_137 | 78.96 | 0.95 | 89.19 | 14.41 |
|  | hsa_miR_186 | 85.8 | 9.59 | 106.54 | 18.32 |
|  | hsa_miR_193 | 58.04 | 3.5 | 57.24 | 4.81 |
|  | hsa_miR_320 | 64.18 | 1.07 | 55.24 | 0.61 |
|  | hsa_miR_155 | 60.36 | 3.84 | 62.98 | 2.17 |
|  | hsa_miR_106b | 84.99 | 2.61 | 102.84 | 10.73 |
|  | hsa_miR_302astar | 106.42 | 1.99 | 124.86 | 3.96 |
|  | hsa_miR_200a | 68.02 | 0.62 | 90.36 | 1.95 |
|  | hsa_miR_192 | 60.52 | 0.62 | 84.26 | 7.34 |
|  | hsa_miR_34b | 27.94 | 2.71 | 32.8 | 7.67 |
|  | hsa_miR_196a | 70.57 | 2.05 | 94.57 | 11.91 |
|  | hsa_miR_34c | 26.33 | 1.16 | 23.43 | 1.21 |
|  | hsa_miR_197 | 69.02 | 0.63 | 69.81 | 1.91 |
|  | hsa_miR_299 | 73.65 | 4.17 | 77.93 | 0.09 |
|  | hsa_miR_198 | 84.12 | 2.06 | 80.39 | 0.61 |
|  | hsa_miR_301 | 95.33 | 3.59 | 103.4 | 0.74 |
|  | hsa_miR_199a | 69.35 | 2.32 | 80.35 | 9.35 |
|  | hsa_miR_99b | 67.74 | 6.81 | 84.13 | 9.52 |
|  | hsa_miR_199astar | 55.21 | 4.24 | 66.71 | 16.21 |
|  | hsa_miR_30e | 104.61 | 2.96 | 106.04 | 13.91 |
|  | hsa_miR_208 | 61.21 | 0.47 | 85.37 | 4.09 |
|  | hsa_miR_302a | 83.84 | 2.8 | 92 | 2.74 |
|  | hsa_miR_130b | 116.36 | 5.94 | 145.71 | 12.26 |
|  | hsa_miR_302cstar | 102.52 | 0.91 | 121.84 | 5.77 |
|  | hsa_miR_367 | 98.09 | 3.54 | 107.63 | 4.84 |
|  | hsa_miR_372 | 74.37 | 1.13 | 70.02 | 2.15 |
|  | hsa_miR_373 | 85.49 | 3.26 | 93.72 | 10.75 |
|  | hsa_miR_108 | 80.54 | 4.58 | 91.97 | 5.94 |
|  | hsa_miR_302bstar | 105.22 | 11.15 | 119.12 | 11.18 |
|  | hsa_miR_129 | 55.75 | 5.88 | 73.42 | 10.05 |
|  | hsa_miR_302b | 72.7 | 1.29 | 80.08 | 5.18 |
|  | hsa_miR_148a | 85.26 | 3.85 | 73.24 | 12.51 |
|  | hsa_miR_368 | 86.7 | 1.37 | 86.41 | 9 |
|  | hsa_miR_30c | 85.95 | 8.95 | 71.42 | 2.62 |
|  | hsa_miR_369 | 92.77 | 1.61 | 93.5 | 6.61 |
|  | hsa_miR_139 | 108.32 | 4.88 | 98.27 | 3.09 |
|  | hsa_miR_370 | 67.98 | 3.62 | 70.43 | 10.77 |
|  | hsa_miR_147 | 60.38 | 0.53 | 63.77 | 5.2 |
|  | hsa_miR_371 | 48.94 | 0.48 | 52.44 | 2.38 |
|  | hsa_miR_7 | 79.75 | 1.8 | 50.66 | 1.16 |
|  | hsa_miR_373star | 106.23 | 4.98 | 118.06 | 7.75 |
|  | hsa_miR_10a | 96.73 | 3.99 | 111.19 | 9.4 |
|  | hsa_miR_374 | 80.38 | 3.69 | 89.81 | 2.34 |
|  | hsa_miR_10b | 88.4 | 6.05 | 87.16 | 13.31 |
|  | stoffel | 64.56 | 5.89 | 84.66 | 2.33 |
|  | mmu_miR_409 | 63.48 | 0.21 | 83.24 | 7.89 |
|  | mmu_miR_410 | 86.62 | 12.94 | 108.07 | 18.03 |
|  | mmu_miR_412 | 74.38 | 6.91 | 74.52 | 6.14 |
|  | hsa_let_7b | 58.08 | 0.12 | 76.79 | 9.71 |
|  | hsa_let_7c | 60.09 | 0.58 | 81.85 | 1.71 |
|  | hsa_miR_18 | 85.76 | 1.51 | 104.39 | 9.38 |
|  | hsa_miR_29a | 74.56 | 3.26 | 90.65 | 8.52 |
|  | hsa_miR_340 | 75.5 | 4.11 | 95.16 | 2.25 |
|  | hsa_miR_34a | 51.78 | 5.68 | 61.33 | 1.91 |
|  | hsa_miR_330 | 104.64 | 5.49 | 115.24 | 8.34 |
|  | hsa_miR_181a | 95.94 | 3.53 | 72.64 | 3.72 |
|  | hsa_miR_328 | 73.89 | 0.04 | 80.12 | 7.75 |
|  | hsa_miR_181c | 83.2 | 5.03 | 80.66 | 7.83 |
|  | hsa_miR_342 | 84.63 | 0.51 | 92.46 | 3.29 |
|  | hsa_miR_182star | 65.55 | 3.32 | 66.54 | 9.57 |
|  | hsa_miR_337 | 66.31 | 1.15 | 60.42 | 3.17 |
|  | hsa_miR_187 | 111.63 | 1.02 | 120.7 | 4.27 |
|  | hsa_miR_323 | 93.54 | 11.99 | 111 | 8.21 |
|  | hsa_miR_199b | 90.2 | 5.37 | 97.84 | 18.61 |
|  | hsa_miR_326 | 95.71 | 7.77 | 113.11 | 11.68 |
|  | hsa_miR_203 | 76.65 | 3.78 | 102.51 | 0.46 |
|  | hsa_miR_151 | 82.14 | 4.55 | 86.74 | 11.75 |
|  | hsa_miR_204 | 80.14 | 1.03 | 91.96 | 9.91 |
|  | hsa_miR_30d | 94.13 | 2.57 | 97.06 | 2.24 |
|  | hsa_miR_182 | 79 | 1.96 | 69.89 | 0.14 |
|  | hsa_miR_183 | 90.66 | 24.6 | 69.03 | 2.89 |
|  | hsa_miR_210 | 58.55 | 0.15 | 77.32 | 3.15 |
|  | hsa_miR_214 | 65.42 | 6.61 | 72.37 | 12.84 |
|  | hsa_miR_133a | 55.3 | 6.18 | 69.59 | 7.67 |
|  | hsa_miR_140 | 73.99 | 0.76 | 95 | 1.07 |
|  | hsa_miR_143 | 87.05 | 4.99 | 124.01 | 13.75 |
|  | hsa_miR_135b | 84.77 | 1.68 | 99.46 | 9.66 |
|  | hsa_miR_205 | 79.32 | 10.37 | 87.51 | 3.96 |
|  | hsa_miR_148b | 104.35 | 8.24 | 77.71 | 0.13 |
|  | hsa_miR_211 | 90.91 | 0.98 | 87.17 | 8.03 |
|  | hsa_miR_331 | 86.98 | 0.78 | 100.85 | 1.04 |
|  | hsa_miR_213 | 106.86 | 2.12 | 117 | 6.09 |
|  | hsa_miR_324_5p | 68.86 | 2.7 | 84.58 | 6.18 |
|  | hsa_miR_215 | 41.14 | 0.74 | 58.46 | 9.8 |
|  | hsa_miR_324_3p | 67.53 | 4.22 | 72.49 | 8.43 |
|  | hsa_miR_216 | 81.44 | 3.56 | 88.23 | 2.93E-03 |
|  | hsa_miR_338 | 111.84 | 2.59 | 134.26 | 10 |
|  | hsa_miR_217 | 87.3 | 11.91 | 98.71 | 6.57 |
|  | hsa_miR_339 | 79.2 | 2.91 | 105.41 | 19.22 |
|  | hsa_miR_218 | 89.43 | 0.33 | 120.69 | 0 |
|  | hsa_miR_146 | 90.53 | 3.17 | 98 | 0.5 |
|  | hsa_miR_154 | 97.17 | 3.52 | 104.16 | 8.27 |
|  | hsa_miR_184 | 89.38 | 3.34 | 100.07 | 4.39 |
|  | hsa_miR_185 | 82.87 | 17.05 | 91.16 | 4.03 |
|  | hsa_miR_206 | 80.95 | 4.73 | 74.19 | 8.7 |
|  | hsa_miR_29c | 75.41 | 3.14 | 101.34 | 9.41 |
|  | hsa_miR_296 | 81.45 | 1.56 | 95.53 | 5.03 |
|  | hsa_miR_335 | 107.98 | 5.81 | 120.92 | 8.78 |
|  | hsa_miR_219 | 99.01 | 1.92 | 138.56 | 24.21 |
|  | hsa_miR_325 | 97.1 | 3.13 | 108.72 | 4.97 |
|  | hsa_miR_220 | 88.2 | 5.03 | 59.21 | 54.44 |
|  | hsa_miR_345 | 73.91 | 3.46 | 39.83 | 0.56 |
|  | hsa_miR_221 | 97.59 | 8.03 | 97.64 | 2.6 |
|  | hsa_miR_196b | 82.37 | 3.87 | 84.9 | 18.68 |
|  | hsa_miR_222 | 84.92 | 1.13 | 90.81 | 2.3 |
|  | mmu_miR_201 | 80.52 | 8.37 | 75.93 | 12.23 |
|  | hsa_miR_223 | 73.68 | 2.74 | 101.68 | 14.63 |
|  | mmu_miR_290 | 82.62 | 6.7 | 75.37 | 7.43 |
|  | hsa_miR_224 | 100.17 | 8.87 | 113.09 | 9.97 |
|  | mmu_miR_291_5p | 70.76 | 3.31 | 81.14 | 0.74 |
|  | hsa_miR_200b | 84.73 | 3.28 | 99.45 | 2.81 |
|  | mmu_miR_292_5p | 88.6 | 8.05 | 94.49 | 6.31 |
|  | hsa_let_7g | 78.02 | 0.23 | 97.59 | 5.88 |
|  | hsa_miR_302c | 79.58 | 5.91 | 94.98 | 12.12 |
|  | hsa_miR_302d | 81.73 | 4.15 | 87.47 | 13.39 |
|  | hsa_miR_133b | 61.31 | 4.4 | 88.9 | 6.13 |
|  | hsa_miR_346 | 53.63 | 0.45 | 47.22 | 5.7 |
|  | mmu_miR_202 | 82.95 | 1.72 | 100.11 | 7.75 |
|  | mmu_miR_207 | 77.75 | 2.85 | 93.96 | 13.65 |
|  | mmu_miR_291_3p | 81.05 | 6.48 | 89.39 | 5.83 |
|  | mmu_miR_292_3p | 81.32 | 3.05 | 103.69 | 12.69 |
|  | mmu_miR_293 | 88.55 | 2.3 | 99.29 | 10.37 |
|  | hsa_let_7i | 95.18 | 1.78 | 119.64 | 12.88 |
|  | mmu_miR_298 | 74.59 | 3.35 | 74.22 | 16.14 |
|  | hsa_miR_1 | 74.72 | 5.61 | 84.6 | 16.95 |
|  | mmu_miR_300 | 95.89 | 2.13 | 90.43 | 15.89 |
|  | hsa_miR_15b | 73.97 | 7.28 | 74.11 | 3.62 |
|  | mmu_let_7dstar | 84.25 | 0.4 | 103.45 | 15.54 |
|  | hsa_miR_23b | 75.67 | 4.08 | 95 | 9.72 |
|  | mmu_miR_322 | 119.01 | 10.58 | 127.29 | 9.84 |
|  | hsa_miR_27b | 66.88 | 6.82 | 71.8 | 11.99 |
|  | mmu_miR_329 | 103.86 | 8.33 | 88.72 | 6.33 |
|  | hsa_miR_30b | 94.4 | 3.06 | 93.5 | 3.38 |
|  | mmu_miR_341 | 95.69 | 0.48 | 57.21 | 0.26 |
|  | hsa_miR_122a | 71.98 | 4.65 | 63.82 | 10.59 |
|  | mmu_miR_350 | 105.88 | 1.92 | 101.81 | 4.76 |
|  | hsa_miR_125b | 99.21 | 1.75 | 122.98 | 4.41 |
|  | mmu_miR_294 | 85.95 | 9.53 | 87.49 | 9.78 |
|  | mmu_miR_295 | 81.53 | 3.72 | 82.76 | 8.75 |
|  | mmu_miR_297 | 64.36 | 3.63 | 86.4 | 16.78 |
|  | mmu_miR_344 | 61.19 | 2.39 | 77.19 | 16.18 |
|  | hsa_let_7a | 45.83 | 1.57 | 68.86 | 5.73 |
|  | hsa_let_7d | 78.68 | 6.17 | 89.75 | 17.05 |
|  | hsa_let_7e | 82.96 | 4.18 | 98.39 | 11.32 |
|  | hsa_let_7f | 62.59 | 3.65 | 71.71 | 11.81 |
|  | mmu_miR_351 | 100.2 | 9.13 | 126.03 | 3.31 |
|  | hsa_miR_128a | 72.27 | 11.09 | 94.08 | 15.79 |
|  | mmu_miR_101b | 101.2 | 3.12 | 72.3 | 11.93 |
|  | hsa_miR_130a | 129.96 | 7.48 | 156.25 | 15.51 |
|  | mmu_miR_7b | 76.6 | 1 | 70.19 | 13.62 |
|  | hsa_miR_132 | 67.39 | 4.89 | 65.37 | 10.49 |
|  | mmu_miR_376a | 90.56 | 1.79 | 113.43 | 6.92 |
|  | hsa_miR_135a | 83.66 | 3.8 | 99.63 | 15.64 |
|  | mmu_miR_380_3p | 83.94 | 9.28 | 45.02 | 4.3 |
|  | hsa_miR_138 | 97.3 | 3.18 | 132.55 | 13.34 |
|  | mmu_miR_376b | 78.13 | 0.32 | 68.03 | 3.85 |
|  | hsa_miR_141 | 60.64 | 6.6 | 76.79 | 14.69 |
|  | mmu_miR_411 | 104.24 | 7 | 117.74 | 14.62 |
|  | hsa_miR_142_5p | 94.62 | 1.05 | 107.25 | 6.54 |
|  | hsa_miR_15a | 67.59 | 2.34 | 102.94 | 10.62 |
|  | hsa_miR_16 | 82.56 | 3.5 | 93 | 2.94 |
|  | hsa_miR_17_5p | 79.5 | 1.4 | 97.87 | 5.1 |
|  | hsa_miR_17_3p | 98.71 | 4.66 | 118.95 | 18.18 |
|  | hsa_miR_19a | 112.55 | 3.97 | 119.34 | 3.4 |
|  | hsa_miR_19b | 117.68 | 0.59 | 116.33 | 17.4 |
|  | hsa_miR_21 | 91.21 | 4.96 | 108.27 | 14.88 |
|  | hsa_miR_142_3p | 69.75 | 3.44 | 68.98 | 1.08 |
|  | hsa_miR_144 | 89.41 | 3.39 | 96.75 | 10.79 |
|  | hsa_miR_145 | 75.15 | 0.2 | 96.48 | 11.23 |
|  | hsa_miR_152 | 100.7 | 1.93 | 125.44 | 12.37 |
|  | hsa_miR_153 | 82.39 | 1.87 | 107.47 | 13.27 |
|  | hsa_miR_191 | 80.17 | 1.28 | 72.27 | 8.85 |
|  | hsa_miR_9 | 103.25 | 2.47 | 118.79 | 11.39 |
|  | hsa_miR_9star | 87.37 | 2.28 | 100.51 | 10.95 |
|  | hsa_miR_22 | 85.96 | 7.29 | 111.81 | 7.17 |
|  | hsa_miR_23a | 93.6 | 6.89 | 99.23 | 13.82 |
|  | hsa_miR_189 | 70.81 | 3.25 | 48.37 | 7.35 |
|  | hsa_miR_24 | 66.77 | 1.46 | 91.35 | 8.74 |
|  | hsa_miR_25 | 76.8 | 1.25 | 81.79 | 1.25 |
|  | hsa_miR_26a | 65.86 | 6.19 | 93.7 | 16.92 |
|  | hsa_miR_26b | 81.42 | 2 | 107.39 | 11.88 |
|  | hsa_miR_27a | 68.83 | 1.47 | 97.6 | 8.25 |
| LOC128439 | NM_139016 | 68.43 | 6.05 | 86.05 | 0.85 |
| MAPK12 | NM_002969 | 69.58 | 0.73 | 71.35 | 5.36 |
| PIM1 | NM_002648 | 65.23 | 1.41 | 69.27 | 0.6 |
| TRPM7 | NM_017672 | 101.76 | 3.23 | 106.82 | 5.57 |
| ACVRL1 | NM_000020 | 87.39 | 7.29 | 93.76 | 14.25 |
| PIK4CA | NM_058004 | 97.17 | 11.33 | 99.84 | 0.86 |
| TRIO | NM_007118 | 87.84 | 2.26 | 89.75 | 3.14 |
| DYRK1B | NM_004714 | 85.92 | 3.17 | 78.93 | 3.33 |
| MOS | NM_005372 | 90.95 | 2.36 | 100.44 | 2.09 |
| WNK4 | NM_032387 | 62.76 | 3.37 | 79.24 | 16.12 |
| TNK2 | NM_005781 | 87 | 1.47 | 94.09 | 8.75 |
| RPS6KC1 | NM_012424 | 75.46 | 2.22 | 69.26 | 0.45 |
| PHKG2 | NM_000294 | 89.01 | 5.46 | 62.68 | 2.41 |
| DMPK | NM_004409 | 80.34 | 0.25 | 86.75 | 4.05 |
| MAP3K10 | NM_002446 | 46.13 | 1.07 | 43.92 | 7.69 |
| MST1R | NM_002447 | 64.03 | 2.73 | 72.06 | 2.37 |
| ALPK3 | NM_020778 | 75.83 | 8.28 | 72.59 | 16.13 |
| PRKAB2 | NM_005399 | 92.61 | 2.21 | 92.95 | 7.64 |
| STK11 | NM_000455 | 88.11 | 6.04 | 95.88 | 11.97 |
| MAP4K3 | NM_003618 | 74.91 | 1.55 | 94.8 | 5.34 |
| MAPK15 | NM_139021 | 69.64 | 1.09 | 76.27 | 0.31 |
| TP53INP2 | NM_021202 | 84.34 | 24.4 | 84.24 | 19.64 |
| CDK9 | NM_001261 | 80.2 | 1.06 | 83.63 | 1.16 |
| TRIB3 | NM_021158 | 79.17 | 0.78 | 70.88 | 8.95 |
| LMTK3 | AB067470 | 85.57 | 0.58 | 90.9 | 9.41 |
| STK23 | NM_014370 | 64.88 | 1.24 | 86.66 | 3.34 |
| LOC390777 | XM_372663 | 89.51 | 8.55 | 107.97 | 6.31 |
| P143 | AF334792 | 89.33 | 4.62 | 99.51 | 3.5 |
| LOC126520 | AK054808 | 67.29 | 5.47 | 89.81 | 9.97 |
|  | XM_210341 | 85.1 | 0.5 | 92.2 | 2.55 |
|  | XM_166676 | 106.76 | 2.8 | 112.34 | 1.9 |
| ALS2CR2 | AF116618 | 70.96 | 3.09 | 33.47 | 3.98 |
| PAK1 | NM_002576 | 109.38 | 0.49 | 111.06 | 2.24 |
| PAK2 | NM_002577 | 92.29 | 2.94 | 85.67 | 24.96 |
| LOC387934 | XM_062966 | 83.84 | 6.35 | 94.1 | 6.86 |
| AKT1 | NM_005163 | 77.65 | 5.43 | 65.99 | 5.2 |
| PRKY | NM_002760 | 94.28 | 2.94 | 106.51 | 2.57 |
| MAP3K14 | NM_003954 | 85.58 | 9.98 | 92.11 | 14.62 |
| BCR | NM_004327 | 89.69 | 4.81 | 66.04 | 3.76 |
| BRSK2 | NM_003957 | 103.34 | 10.42 | 118.89 | 19.82 |
| BMPR1A | NM_004329 | 70.67 | 2.32 | 89.41 | 5.49 |
| CDC2 | NM_001786 | 58.92 | 1.09 | 73.52 | 10.67 |
| STK17A | NM_004760 | 90.41 | 8.63 | 93.25 | 7.17 |
| BRAF | NM_004333 | 44.5 | 9.87 | 42.95 | 3.93 |
| BUB1 | NM_004336 | 79.3 | 0.97 | 86.68 | 10.77 |
| WEE1 | NM_003390 | 25.49 | 1.1 | 26.54 | 7.69 |
| PFTK1 | NM_012395 | 94.1 | 7.34 | 108.6 | 14.79 |
| PRKCG | NM_002739 | 92.78 | 0.02 | 92.73 | 8.49 |
| NEK9 | NM_033116 | 87.84 | 1.57 | 86.17 | 7.73 |
| EPHA8 | NM_020526 | 66.13 | 0.55 | 37.12 | 34.22 |
| PKN1 | NM_002741 | 88.66 | 1.39 | 103.82 | 1.73 |
| STK25 | NM_006374 | 86.68 | 2.48 | 104.42 | 4.24 |
| PRKCZ | NM_002744 | 92.99 | 1.02 | 104.69 | 0.17 |
| GRK1 | NM_002929 | 52.75 | 4.54 | 40.16 | 7.65 |
| MAPK11 | NM_002751 | 88.9 | 0.36 | 83.32 | 3.4 |
| MAP2K1 | NM_002755 | 114.17 | 0.27 | 105.12 | 1.1 |
| JAK3 | NM_000215 | 40.09 | 17.77 | 54.11 | 5.22 |
| MAP2K6 | NM_002758 | 83.09 | 3.92 | 70.71 | 3.45 |
| EIF2AK2 | NM_002759 | 71.21 | 2.48 | 77.8 | 0.26 |
| RALB | NM_002881 | 85.09 | 1.2 | 76.03 | 16.19 |
| MKNK1 | NM_003684 | 84.39 | 7.81 | 67.02 | 11.81 |
| PCTK1 | NM_033018 | 80.18 | 12.12 | 29.77 | 4.05 |
| PXK | NM_017771 | 101.15 | 8.62 | 99.13 | 4.63 |
| MARK4 | NM_031417 | 93.34 | 7.21 | 110.65 | 1.52 |
| TSSK6 | NM_032037 | 84.73 | 11.19 | 115.49 | 11.14 |
| LIMK1 | NM_002314 | 68.55 | 9.01 | 73.96 | 1.04 |
| RIPK1 | NM_003804 | 69.02 | 7.82 | 77.58 | 1.82 |
| MAPKAPK3 | NM_004635 | 86.78 | 1.61 | 97.06 | 6.06 |
| HIPK4 | NM_144685 | 80.06 | 3.4 | 58 | 7.47 |
| GUCY2D | NM_000180 | 81.1 | 3.79 | 96.32 | 1.91 |
| PINK1 | NM_032409 | 82.1 | 7.44 | 90.61 | 0.79 |
| RPS6KL1 | NM_031464 | 75.02 | 2.21 | 66.54 | 9.29 |
| SGK | NM_005627 | 93.1 | 3.3 | 97.77 | 4.53 |
| TESK1 | NM_006285 | 91.38 | 3.29 | 84 | 23.43 |
| ANKK1 | NM_178510 | 64.98 | 7.76 | 54 | 10.2 |
| SRMS | NM_080823 | 53.4 | 31.41 | 88.28 | 0.49 |
| NLK | NM_016231 | 99.09 | 4.99 | 89.91 | 2.44 |
| LATS1 | NM_004690 | 76.45 | 0.14 | 66.9 | 3.64 |
| PRKD3 | NM_005813 | 80.76 | 1.01 | 83.65 | 19.24 |
| PRKAA1 | NM_006251 | 55.57 | 7.99 | 50.35 | 4.68 |
| VRK2 | NM_006296 | 92.26 | 0.98 | 58.74 | 1.45 |
| PIN1 | NM_006221 | 91.31 | 2.06 | 63.17 | 16.03 |
| PKN2 | NM_006256 | 79.04 | 5.28 | 62.88 | 8.17 |
| SKP1A | NM_170679 | 95.86 | 0.06 | 110.11 | 16.93 |
| CSNK1G2 | NM_001319 | 98.45 | 9.28 | 72.88 | 13.77 |
| SCYL2 | NM_017988 | 99.42 | 6.63 | 101.91 | 15.6 |
| RAF1 | NM_002880 | 93.66 | 6.93 | 99.77 | 8.9 |
| LTK | NM_002344 | 57.69 | 1.74 | 68.88 | 16.21 |
| YES1 | AF119914 | 62.44 | 3.98 | 72.55 | 7.83 |
|  | XM_210659 | 53.9 | 7.13 | 58.06 | 1.31 |
| EPHA10 | XM_496357 | 58.5 | 10.53 | 68.86 | 11.84 |
|  | XM_291786 | 69.05 | 1.42 | 75.85 | 5.46 |
| LOC441777 | BC052334 | 113.64 | 13.66 | 113.52 | 4.04 |
| PRKXP1 | XM_497470 | 61.61 | 3.1 | 70.16 | 2.62 |
| NEK1 | Z25431 | 81.6 | 5.1 | 72.88 | 11.98 |
| EEF1A2 | NM_001958 | 94.55 | 1.35 | 96.08 | 2.56 |
| STMN3 | NM_015894 | 90.91 | 1.52 | 95.6 | 4.72 |
| PPP4R1L | AK057583 | 87.89 | 2.77 | 79.92 | 2.08 |
| TAOK1 | AB037782 | 127.1 | 6.42 | 123.03 | 5.7 |
| STK32C | NM_173575 | 87.37 | 7.86 | 83.18 | 7.41 |
| TNK1 | NM_003985 | 80.19 | 1.28 | 73.99 | 2.57 |
| PTK7 | NM_002821 | 93.4 | 2.26 | 104.63 | 4.36 |
| PDK1 | NM_002610 | 92.02 | 3.76 | 108.78 | 6.03 |
| TAOK2 | NM_016151 | 94.16 | 3.43 | 106.94 | 0.49 |
| FGFR3 | NM_000142 | 98.6 | 5.78 | 98.94 | 12.19 |
| MAPK1 | NM_138957 | 61.87 | 1.4 | 41.3 | 1.69 |
| PLK3 | NM_004073 | 88.69 | 6.02 | 52.51 | 3.25 |
| FES | NM_002005 | 77.68 | 4.82 | 77.12 | 4.29 |
| DAPK1 | NM_004938 | 72.99 | 1.43 | 64.18 | 14.4 |
| MAPK7 | NM_139034 | 89.32 | 5.59 | 103.4 | 23.01 |
| STK17B | NM_004226 | 97.85 | 7.31 | 103.36 | 4.71 |
| CDC42BPB | NM_006035 | 96.04 | 0.21 | 93.17 | 3.56 |
| MAP3K3 | NM_002401 | 101.25 | 4.92 | 112.26 | 8.88 |
| PLK1 | NM_005030 | 6.81 | 0.55 | 5.13 | 0.56 |
| RPS6KA1 | NM_002953 | 84.35 | 0.98 | 81.7 | 7.43 |
| CSNK1A1 | NM_001892 | 87.89 | 2.23 | 100.61 | 6.08 |
| ZAP70 | NM_001079 | 90.6 | 1.27 | 98.05 | 0.87 |
| PTK6 | NM_005975 | 98.51 | 6.7 | 111.26 | 15.68 |
| EPHB3 | NM_004443 | 49.27 | 1.19 | 37.52 | 5.66 |
| EPHB6 | NM_004445 | 60.51 | 4.61 | 63.76 | 1.63 |
| MAP3K11 | NM_002419 | 107.05 | 5.3 | 116.34 | 20.02 |
| MAST3 | AB011133 | 99.48 | 0.3 | 70.15 | 2.74 |
| MASK | NM_016542 | 80.22 | 1.6 | 60.27 | 9.08 |
| FLT4 | NM_002020 | 102.52 | 7.01 | 78.66 | 18.17 |
| INSRR | NM_014215 | 89.41 | 13.5 | 104.56 | 5.64 |
| GAK | NM_005255 | 108.97 | 2.25 | 96.73 | 0.19 |
| MAPK3 | NM_002746 | 71.77 | 16.53 | 79.53 | 3.21 |
| POLK | NM_016218 | 89.07 | 1.36 | 90.73 | 8.67 |
| MAPK6 | NM_002748 | 91.53 | 1.7 | 102.4 | 1.63 |
| ROCK2 | NM_004850 | 103.42 | 14.21 | 102.88 | 3.63 |
| CSNK1E | NM_152221 | 86.15 | 3.94 | 85.1 | 3.46 |
| PTK2B | NM_173176 | 86.45 | 4.96 | 88.58 | 2.78 |
| BAIAP2 | NM_017451 | 68.79 | 3.02 | 71.48 | 3.48 |
| EIF2AK4 | AK027011 | 100.61 | 7.67 | 106.12 | 5.9 |
| PTK9 | NM_002822 | 110.59 | 2.85 | 100.57 | 4.73 |
| EGFR | NM_005228 | 73.51 | 2.3 | 78.26 | 0.19 |
| MAPK8 | NM_002750 | 55.03 | 1.06 | 62.71 | 0.62 |
| TTN | NM_003319 | 68.52 | 7.42 | 69.69 | 3.17 |
| POLR2K | NM_005034 | 60.29 | 2.38 | 39.9 | 3.29 |
| WNK3 | NM_001002838 | 70.82 | 12.48 | 85.73 | 5.05 |
| AATK | AK131395 | 73.47 | 0.41 | 87.04 | 0.76 |
| LYK5 | NM_001003787 | 41.08 | 2.12 | 47.39 | 9.18 |
| PRKCD | NM_212539 | 62.84 | 4.99 | 80.64 | 0.46 |
| CDC42BPG | NM_017525 | 70.5 | 5.43 | 42.16 | 2.99 |
| FGFR4 | NM_213647 | 93.19 | 10.18 | 66.43 | 40.68 |
| MAP3K9 | NM_033141 | 59.31 | 1.45 | 79.48 | 7.56 |
| DCAMKL2 | NM_152619 | 114.29 | 1.07 | 90.54 | 7.44 |
| CAMK2B | NM_172078 | 68.56 | 2.57 | 67.92 | 14.55 |
| CSK | NM_004383 | 66.72 | 10.7 | 55.54 | 3.27 |
| MAP2K7 | NM_145185 | 115.13 | 8.11 | 122.2 | 4.53 |
| CSNK1G3 | NM_004384 | 104.26 | 1.96 | 124.94 | 8.18 |
| SLK | NM_014720 | 66.84 | 8.86 | 63.8 | 16.98 |
| ABCC1 | NM_004996 | 93.47 | 3.3 | 95.96 | 9.49 |
| PRKX | NM_005044 | 91.52 | 6.63 | 86.8 | 15.47 |
| DAPK3 | NM_001348 | 131.76 | 4.61 | 108.37 | 8.84 |
| ABL1 | NM_007313 | 108.03 | 5.2 | 133.33 | 11.39 |
| MAPK9 | NM_002752 | 71.79 | 5.99 | 105.16 | 16.27 |
| ABL2 | NM_005158 | 53.47 | 0.4 | 55.28 | 4.51 |
| ARAF | NM_001654 | 85.73 | 11.03 | 73.39 | 10.5 |
| PIK3R3 | NM_003629 | 65.13 | 6.54 | 76.57 | 1.42 |
| YES1 | NM_005433 | 80.97 | 2.58 | 56.93 | 0.44 |
| NEK2 | NM_002497 | 90.1 | 3.45 | 59.48 | 7.42 |
| SMG1 | NM_015092 | 109.67 | 16.3 | 103.77 | 0.39 |
| MET | NM_000245 | 86.46 | 2.4 | 86.85 | 1.19 |
| HSPB8 | NM_014365 | 98.7 | 10.65 | 84.58 | 0.53 |
| TRIM28 | NM_005762 | 25.97 | 4.44 | 20.16 | 3.93 |
| RPS6KA4 | NM_003942 | 101.2 | 1.87 | 90.29 | 9.84 |
| LYN | NM_002350 | 73.98 | 0.14 | 67.98 | 3.3 |
| AXL | NM_001699 | 104.39 | 3.98 | 101.24 | 6.01 |
| RPS6KB2 | NM_003952 | 93.13 | 0.15 | 59.24 | 31.07 |
| MAP3K6 | NM_004672 | 100.46 | 5.32 | 125.96 | 11.28 |
| MAP4K5 | NM_198794 | 98.06 | 5.64 | 74.39 | 18.44 |
| IRAK4 | NM_016123 | 103.62 | 0.03 | 104.82 | 10.61 |
| MARK2 | NM_017490 | 84.91 | 9.15 | 97.77 | 2.01 |
| KIAA1639 | XM_290923 | 98.84 | 1.78 | 102.05 | 1.95 |
| AKT2 | NM_001626 | 92.87 | 7.11 | 96.85 | 6.58 |
| RIOK3 | NM_003831 | 106.71 | 2.83 | 109.09 | 1.64 |
| SNF1LK | NM_173354 | 51.21 | 0.1 | 66.46 | 4.09 |
| CAMK1D | NM_020397 | 80.27 | 0.59 | 87.55 | 5.11 |
| DDR1 | NM_001954 | 85.64 | 13.28 | 76.94 | 10.21 |
| CRKRS | NM_016507 | 96.9 | 2.69 | 88.63 | 4.26 |
| WNK1 | NM_018979 | 59.58 | 1.92 | 54.35 | 6.66 |
| MAP2K5 | NM_002757 | 92.97 | 2.53 | 101.68 | 7.17 |
| GSK3A | NM_019884 | 97.11 | 1.12 | 94.45 | 2.27 |
| CDKL5 | NM_003159 | 89.83 | 5.88 | 85.85 | 10.1 |
| IKBKB | NM_001556 | 106.93 | 2.32 | 92.46 | 18.28 |
| ROCK1 | NM_005406 | 89.65 | 0.98 | 91.78 | 4.79 |
| CDK8 | NM_001260 | 98.9 | 5.02 | 98.21 | 1.44 |
| BRD4 | NM_014299 | 61.92 | 3.75 | 42.48 | 13.32 |
| PKN3 | NM_013355 | 99.34 | 9.92 | 81.36 | 8.89 |
| AURKA | NM_198437 | 18.04 | 3.65 | 3.57 | 0.35 |
| ADCK1 | NM_020421 | 99.35 | 6.33 | 100.86 | 6.82 |
| FASTK | NM_006712 | 83.77 | 5.24 | 81.37 | 6.71 |
| CABC1 | NM_020247 | 89.77 | 1.04 | 77.14 | 0.42 |
| MAP2K2 | NM_030662 | 74.62 | 1.33 | 52.86 | 3.84 |
| PLK4 | NM_014264 | 75.31 | 1.85 | 63.81 | 3.53 |
| MAP3K2 | NM_006609 | 82.8 | 17.26 | 69.18 | 10.45 |
| CDK4 | NM_000075 | 89.15 | 3.98 | 78.44 | 4.44 |
| SCYL1 | NM_020680 | 67.49 | 3.81 | 66.7 | 3.66 |
| HIPK3 | NM_005734 | 99.05 | 11.38 | 103.57 | 8.02 |
| CHEK2 | NM_007194 | 30.52 | 0.38 | 46.92 | 0.74 |
| TLK2 | NM_006852 | 88.21 | 4.18 | 44.33 | 21.67 |
| SCYL3 | NM_020423 | 106.79 | 1.23 | 115.54 | 9.32 |
| UHMK1 | NM_175866 | 80.05 | 2.65 | 81.59 | 8.25 |
| MAP3K7 | NM_145331 | 106.58 | 2.54 | 59.39 | 31.8 |
| MAPKAPK5 | NM_003668 | 66.7 | 1.11 | 71.76 | 3.4 |
| VRK1 | NM_003384 | 72.46 | 4.89 | 74.34 | 0.67 |
| FLJ23356 | NM_032237 | 71.16 | 3.58 | 81.07 | 5.4 |
| BRD3 | NM_007371 | 50.22 | 4.04 | 37.74 | 2.71 |
| CCT2 | NM_006431 | 55.39 | 2.99 | 66.33 | 5.25 |
| PCTK3 | NM_212503 | 83.02 | 3.02 | 86.45 | 11.03 |
| RPS6KB1 | NM_003161 | 74.47 | 3.84 | 88.9 | 10.1 |
| NRBP | NM_013392 | 69.66 | 0.33 | 80.75 | 3.17 |
| MAP2K4 | NM_003010 | 78.96 | 11.69 | 52.41 | 6.21 |
| CHUK | NM_001278 | 109.38 | 5.78 | 107.69 | 5.25 |
| CDK5 | NM_004935 | 94.28 | 7.98 | 105.13 | 2.28 |
| DYRK4 | NM_003845 | 110.87 | 2.73 | 125.04 | 16.39 |
| AAK1 | NM_014911 | 89.97 | 10.13 | 103.32 | 9.84 |
| MASTL | NM_032844 | 76.64 | 4.28 | 70.62 | 12.79 |
| FYN | NM_002037 | 71.41 | 8.18 | 55.96 | 5.1 |
| CLK3 | NM_003992 | 85.11 | 5.02 | 79.87 | 7.16 |
| MINK1 | NM_015716 | 66.4 | 7.98 | 71.49 | 5.71 |
| CLK2 | NM_003993 | 71.24 | 4.08 | 65.03 | 2.48 |
| MAP4K2 | NM_004579 | 61.44 | 13.12 | 74.53 | 10.19 |
| MKNK2 | NM_017572 | 80.76 | 2.88 | 82.8 | 3.12 |
| CDC2L1 | NM_001787 | 62 | 6.59 | 62.85 | 4.87 |
| SNRK | NM_017719 | 47.45 | 2.63 | 48.48 | 8.33 |
| NPR1 | NM_000906 | 69.57 | 1.62 | 41.61 | 34.31 |
| TYRO3 | NM_006293 | 89.73 | 4.69 | 94.1 | 2.79 |
| EIF2AK3 | NM_004836 | 55.76 | 8.84 | 66.74 | 8.03 |
| TGFBR1 | NM_004612 | 87.55 | 0.04 | 70.71 | 8.55 |
| JAK1 | NM_002227 | 74.65 | 6.15 | 83.87 | 1.16 |
| PIK3CA | NM_006218 | 70.41 | 1.77 | 64.9 | 8.33 |
| EPHA2 | NM_004431 | 71.1 | 2.94 | 71.93 | 8.97 |
| LMTK2 | NM_014916 | 103.4 | 5.06 | 95.8 | 0.9 |
| TBK1 | NM_013254 | 70.06 | 2.08 | 67.55 | 11.37 |
| STK3 | NM_006281 | 38.56 | 0.15 | 35.4 | 0.12 |
| STK10 | NM_005990 | 108.77 | 4.53 | 107.65 | 12.02 |
| ULK1 | NM_003565 | 92.66 | 8.07 | 84.6 | 18.49 |
| BCKDK | NM_005881 | 77.47 | 2.66 | 70.85 | 1.21 |
| HIPK1 | NM_198268 | 95.53 | 10.89 | 91.34 | 10.71 |
| KIAA2002 | XM_370878 | 85.79 | 8.42 | 77.92 | 5.35 |
| GRK6 | NM_002082 | 83.83 | 3.84 | 97.77 | 11.02 |
| IRAK1 | NM_001569 | 82.81 | 5.41 | 73.75 | 12.04 |
| TRIM33 | NM_015906 | 86.03 | 4.4 | 71.76 | 6.48 |
| PIK3CB | NM_006219 | 63.51 | 8.62 | 54.19 | 6.82 |
| CRKL | NM_005207 | 66.79 | 1.87 | 66.09 | 6.66 |
| BMPR2 | NM_001204 | 78.87 | 0.47 | 74.55 | 8.35 |
| CSNK1G1 | NM_022048 | 53.42 | 4.35 | 46.82 | 1.8 |
| CDK10 | NM_003674 | 60.52 | 3.82 | 35.45 | 0.64 |
| PIK3C2B | NM_002646 | 111.05 | 0.95 | 72.48 | 1.88 |
| KIAA0999 | NM_025164 | 95.9 | 0.83 | 89.18 | 3.54 |
| MAP3K4 | NM_005922 | 105.39 | 4.33 | 91.51 | 3.63 |
| PDK4 | NM_002612 | 99.94 | 1.83 | 111.3 | 6.41 |
| TRRAP | NM_003496 | 71.28 | 2.98 | 82.33 | 8.1 |
| AURKB | NM_004217 | 45.17 | 4.28 | 25.95 | 1.19 |
| CSNK1D | NM_001893 | 81.37 | 6.16 | 98.43 | 10.07 |
| CLK1 | NM_004071 | 56.52 | 1.92 | 59.44 | 3.3 |
| PDPK1 | NM_002613 | 90.05 | 1.45 | 93.02 | 4.6 |
| STK38 | NM_007271 | 86.56 | 4.64 | 84.16 | 15.23 |
| DKFZp761P0423 | XM_291277 | 66.09 | 0.68 | 44.09 | 2.13 |
| STK4 | NM_006282 | 94.68 | 3.07 | 97.67 | 8.46 |
| CSNK2A2 | NM_001896 | 76.59 | 0.92 | 87.95 | 14.88 |
| PRKACA | NM_002730 | 97.77 | 1.15 | 89.46 | 2.27 |
| PIM2 | NM_006875 | 93.72 | 0.13 | 95.1 | 10.88 |
| PRKACB | NM_002731 | 85.24 | 0.31 | 85.75 | 11.58 |
| TAF1 | NM_138923 | 81.58 | 4.28 | 86.5 | 25.43 |
| CDC42BPA | NM_003607 | 73.65 | 0.57 | 77.52 | 2.01 |
| CSNK1A1L | NM_145203 | 77.58 | 10.25 | 96.06 | 8.43 |
| MAPKAPK2 | NM_032960 | 90.98 | 2.18 | 91.66 | 6.03 |
| MGC16169 | NM_033115 | 71.17 | 1.01 | 78.38 | 10.62 |
| TTK | NM_003318 | 77.18 | 1.49 | 52.55 | 5.36 |
| PRKAG1 | NM_002733 | 97.56 | 2.27 | 105.07 | 1.08 |
| MAST2 | NM_015112 | 94.43 | 1.97 | 60.33 | 40.34 |
| TANK | NM_004180 | 91.6 | 5.42 | 49.05 | 3.22 |
| RIOK2 | NM_018343 | 75.97 | 1.17 | 78.05 | 15.57 |
| PRKAA2 | NM_006252 | 95.06 | 3.51 | 74.29 | 3.83 |
| SRPK2 | NM_182691 | 75.73 | 7.77 | 86.21 | 14.48 |
| CDC2L6 | NM_015076 | 56.32 | 2.79 | 47.8 | 3.47 |
| SRPK1 | NM_003137 | 68.44 | 7.64 | 92.31 | 19.18 |
| POLR3K | NM_016310 | 70.02 | 7.17 | 80.87 | 10.63 |
| MAPK14 | NM_001315 | 60.1 | 5.59 | 69.91 | 15.32 |
| ILK | NM_004517 | 87.08 | 3.76 | 83.31 | 13.92 |
| ICK | NM_014920 | 53.9 | 2.85 | 54.04 | 1.27 |
| EPHB4 | NM_004444 | 88.65 | 1.85 | 80.54 | 4.56 |
| PIK3C3 | NM_002647 | 81.25 | 2.62 | 86.48 | 11.99 |
| PRKAR1A | NM_002734 | 93.36 | 1.15 | 92.13 | 13.1 |
| PRPF4B | NM_176800 | 25 | 2.95 | 7.22 | 1.4 |
| PIK3R4 | NM_014602 | 95.81 | 6.15 | 91.27 | 2.22 |
| MARK3 | NM_002376 | 103 | 1.45 | 98.91 | 4.16 |
| PRKCA | NM_002737 | 97.35 | 3.41 | 92.16 | 2.06 |
| ADCK4 | NM_024876 | 79.62 | 0.64 | 69.69 | 14.9 |
| PRKCB1 | NM_002738 | 65.44 | 2.42 | 85.62 | 7.31 |
| RIPK2 | NM_003821 | 89.97 | 6.67 | 92.53 | 7.73 |
| BUB1B | NM_001211 | 91.92 | 1.84E-03 | 90.59 | 0.35 |
| RPS6KA3 | NM_004586 | 91.25 | 6.92 | 95.74 | 7.8 |
| FER | NM_005246 | 82.27 | 6.43 | 70.52 | 8.34 |
| PRKDC | NM_006904 | 90.44 | 11.2 | 98.04 | 10.03 |
| CDC2L5 | NM_003718 | 76.06 | 3.23 | 61.71 | 7.5 |
| ERBB2 | NM_004448 | 96.29 | 7.78 | 97.82 | 9.99 |
| PIK3R2 | NM_005027 | 81.24 | 9.71 | 71.05 | 3.05 |
| RIOK1 | NM_031480 | 69.74 | 2.64 | 76.64 | 1.66 |
| STK24 | NM_003576 | 99.22 | 2.64 | 105.82 | 7.29 |
| PCTK2 | NM_002595 | 107.9 | 1.22 | 92.75 | 10.3 |
| TAF1L | NM_153809 | 91.61 | 7.22 | 110.71 | 16.09 |
| SNF1LK2 | NM_015191 | 76.52 | 1.31 | 28.43 | 2.61 |
| DYRK1A | NM_130436 | 99.7 | 3.93 | 103.18 | 10.74 |
| CIT | NM_007174 | 76.67 | 4.78 | 91.31 | 12.46 |
| CLK4 | NM_020666 | 106.76 | 1.79 | 109.87 | 1.02 |
| TTBK1 | XM_166453 | 89.71 | 6.27 | 97.08 | 7.12 |
| CDK2 | NM_001798 | 74.39 | 2.16 | 83.57 | 10.36 |
| CHEK1 | NM_001274 | 68.71 | 5.19 | 77.64 | 14.86 |
| CDK7 | NM_001799 | 103.78 | 2.01 | 116.82 | 2.33 |
| PBK | NM_018492 | 74.88 | 3.79 | 85.13 | 0.59 |
| PLK2 | NM_006622 | 92.08 | 8.56 | 69.5 | 13.39 |
| PTK2 | NM_005607 | 98.5 | 9.57 | 69.31 | 2.61 |
| ULK3 | NM_015518 | 107.75 | 11.36 | 92.12 | 28.89 |
| PDK3 | NM_005391 | 96.48 | 8.05 | 108.49 | 11.46 |
| LATS2 | NM_014572 | 78.97 | 1.17 | 86.31 | 18.55 |
| MATK | NM_139355 | 103.68 | 0.85 | 112.9 | 10.49 |
| PTK9L | NM_007284 | 77.7 | 1.5 | 85.18 | 13.46 |
| PIK4CB | NM_002651 | 63.78 | 4.24 | 74.54 | 4.03 |
| PI4KII | NM_018425 | 92.31 | 7.59 | 97.36 | 7.27 |
| TP53RK | NM_033550 | 69.14 | 5.17 | 65.79 | 21.09 |
| EEF2K | NM_013302 | 115.65 | 9.69 | 108.88 | 3.34 |
| GSK3B | NM_002093 | 92.01 | 3.31 | 106.83 | 7.95 |
| TAOK3 | NM_016281 | 92.96 | 1.82 | 103.1 | 3.04 |
| MAST1 | NM_014975 | 98.19 | 3.72 | 100.62 | 15.93 |
| RYK | NM_002958 | 72.7 | 0.03 | 65.35 | 13.57 |
| CASK | NM_003688 | 90.87 | 5.16 | 79.31 | 0.3 |
| NEK7 | NM_133494 | 98.53 | 5.07 | 111.94 | 0.09 |
| PRKCI | NM_002740 | 92.32 | 0.05 | 112.7 | 17.75 |
| RAD18 | NM_020165 | 101.91 | 1.6 | 89.69 | 7.33 |
| TLK1 | NM_012290 | 93.98 | 1.83 | 96.42 | 9.58 |
| BMP2K | NM_198892 | 48.27 | 5.04 | 55.65 | 6.59 |
| PIK3C2A | NM_002645 | 94.41 | 3.15 | 92.04 | 19.12 |
| MAP2K3 | NM_145109 | 85.02 | 7.74 | 95.79 | 1.4 |
| BRD2 | NM_005104 | 99.4 | 5.34 | 88.07 | 9.87 |
| FRAP1 | NM_004958 | 67.44 | 2.75 | 56.96 | 9.49 |
| MELK | NM_014791 | 86.62 | 9.6 | 87.71 | 8.82 |
| CDC7 | NM_003503 | 103.75 | 8.77 | 108.52 | 3.61 |
| OXSR1 | NM_005109 | 98.89 | 12.68 | 115.63 | 10.37 |
| ADRBK1 | NM_001619 | 96.2 | 1.51 | 88.1 | 19.02 |
| ALS2CR2 | NM_018571 | 55.83 | 6.36 | 51.3 | 8.31 |
| IKBKE | XM_375834 | 88.34 | 5.79 | 111.63 | 13.64 |
